# Supplementary material for: Dearomative 1,4-difunctionalization of naphthalenes via palladium-catalyzed tandem Heck/Suzuki coupling reaction
Source: Nat Commun. 2020 Sep 1;11:4380. doi: 10.1038/s41467-020-18137-w (PMC7463262; doi:10.1038/s41467-020-18137-w)
Supplement: Supplementary file 1 — Supplementary Information [file 41467_2020_18137_MOESM1_ESM.pdf]

Supplementary Information

Dearomative 1,4-Difunctionalization of Naphthalenes via Palladium-Catalyzed Tandem Heck/Suzuki Coupling Reaction

Zhou et al.

Supplementary Methods

General information

Reactions and manipulations involving organometallic or moisture sensitive compounds were carried out under dry nitrogen and glassware dried by heating gun for 5 min prior to use. ^{1}H and ^{13}C spectra were recorded on Bruker AVANCE III 400, 500 or 600 MHz using $CDCl_3$ as solvent with TMS as internal standard. Anhydrous toluene was freshly distilled over Na and benzophenone. Melting points were measured on a Büchi Melting Point B-545 apparatus and uncorrected. Commercial reagents were used as received without further purification unless otherwise noticed. HRMS were recorded on Agilent 6210 TOF LC/MS mass spectrometer. Column chromatography was carried out using silica gel (200-300 mesh). Ligands **L1-L5** were purchased or prepared according to the literature procedures[1] and used directly as received.

Typical procedure for the synthesis of Substrates 1

Substrates **1** were prepared by the condensation of 1-naphthoyl chloride with N-substituted-2-bromoanilines. Iodo-substrate **1a’** is known and prepared according to the literature method.[2]

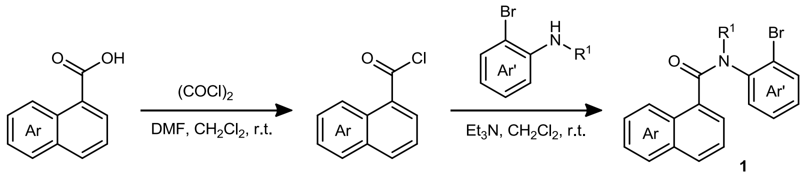

To a stirred solution of 1-naphthoic acid (5.0 mmol, 1.0 equiv.) in CH_2Cl_2 (15 mL) were added a catalytic amount of DMF (10 μL) and $(COCl)_2$ (7.5 mmol, 1.5 equiv.), and the mixture was stirred for 3 h at room temperature. The mixture was then concentrated under reduced pressure. To a stirred solution of this residue in CH_2Cl_2 (5.0 mL) was added a mixture of *N*-substituted-2-bromoanilines (7.5 mmol, 1.5 equiv.)

and Et_3N (10.0 mmol, 2.0 equiv.) in CH_2Cl_2 (10 mL). The mixture was stirred at room temperature overnight and then concentrated under vacuum. The residue was diluted with EtOAc, washed with brine, and dried over Na_2SO_4. After filtration, the solvent was concentrated under vacuum and the residue was purified by flash chromatography on silica gel, eluting with ethyl acetate/petroleum ether (*v*/*v*) to afford the substrates.

N-(2-bromophenyl)-*N*-methyl-1-naphthamide (**1a**)

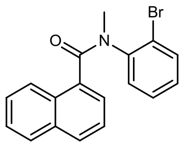

Purified by chromatography on silica gel, eluting with ethyl acetate/petroleum ether 1:10 (*v*/*v*); white solid, Mp = 98-100 ^{o}C, 74% yield; ^{1}H NMR (500 MHz, $CDCl_3$): δ 8.20 (d, *J* = 8.5 Hz, 0.86H), 7.96-7.92 (m, 0.37H), 7.78-7.75 (m, 0.98H), 7.67 (d, *J* = 8.0 Hz, 0.96H), 7.63-7.47 (m, 3.40H), 7.41-7.38 (m, 0.80H), 7.32-7.28 (m, 0.45H), 7.21-7.17 (m, 0.80H), 6.99-6.86 (m, 2.39H), 3.54 (s, 2.42H), 3.11 (s, 0.55H). ^{13}C NMR (125 MHz, $CDCl_3$): δ 170.4, 142.8, 141.9, 134.0, 133.7, 133.5, 133.2, 130.2, 129.7, 129.6, 129.43, 129.42, 129.2, 129.0, 128.8, 128.4, 128.1, 128.0, 127.0, 126.7, 126.4, 126.0, 125.5, 125.0, 124.2, 124.18, 124.14, 122.4, 39.5, 36.3. HRMS *m*/*z* (ESI+): Calculated for $C_{18}H_{15}{}^{79}BrNO$ ($[M+H]^+$): 340.0332, found 340.0332.

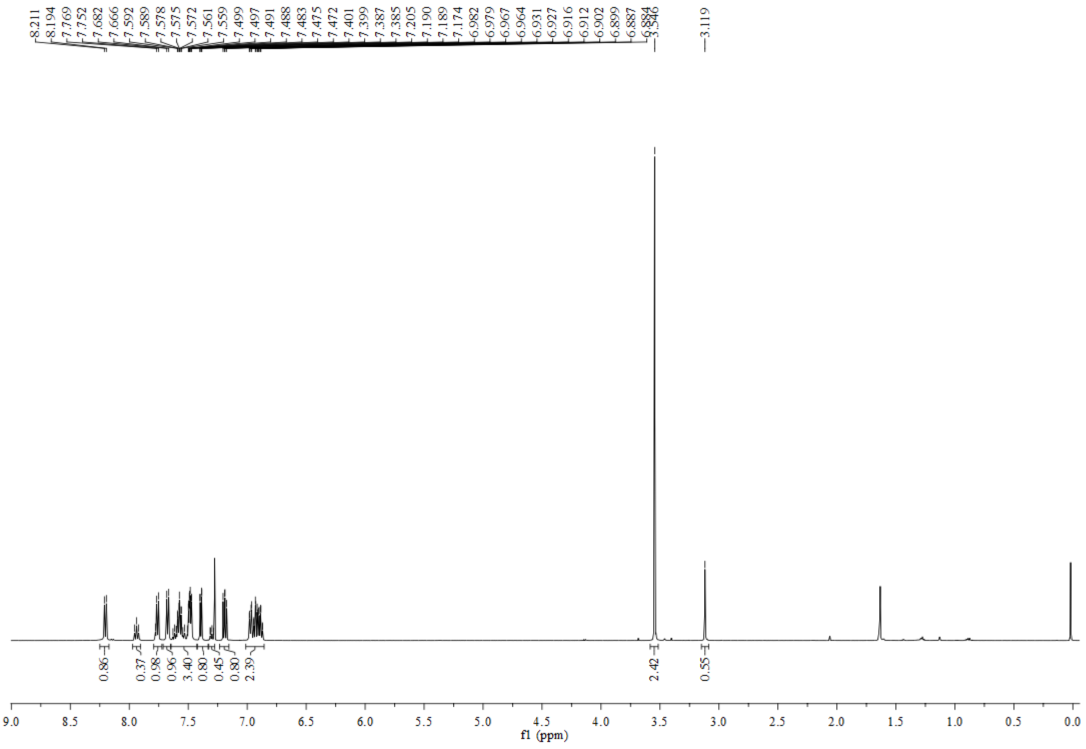

Supplementary Figure 1. ^{1}H NMR Spectra of compound **1a**.

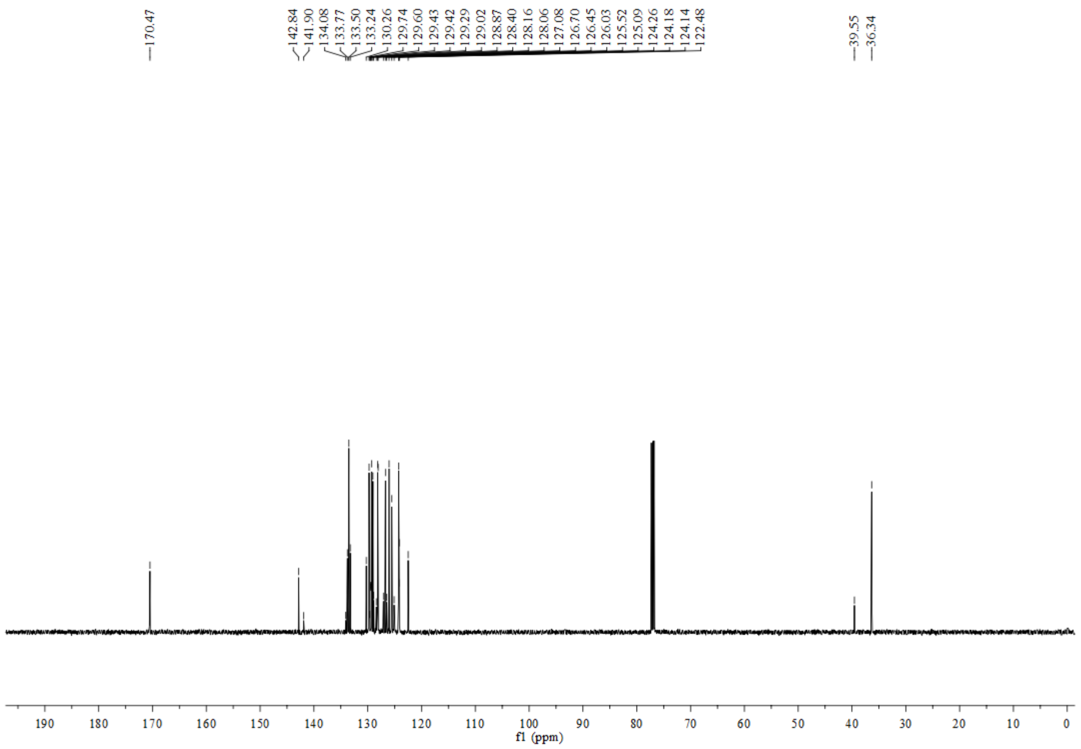

Supplementary Figure 2. ^{13}C NMR Spectra of compound **1a**.

N-(2-chlorophenyl)-*N*-methyl-1-naphthamide (**1a''**)

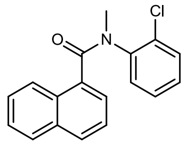

Purified by chromatography on silica gel, eluting with ethyl acetate/petroleum ether 1:5 (*v*/*v*); white solid, Mp = 88-90 °C, 83% yield; ^{1}H NMR (600 MHz, $CDCl_3$): δ 8.21 (d, *J* = 8.4 Hz, 0.93H), 7.95-7.91 (m, 0.35H), 7.75 (d, *J* = 7.8 Hz, 0.83H), 7.66 (d, *J* = 7.8 Hz, 0.86H), 7.63-7.52 (m, 1.76H), 7.49-7.41 (m, 1.08H), 7.36 (d, *J* = 7.2 Hz, 1.00H), 7.29-7.26 (m, 0.77H), 7.20-7.17 (m, 0.83H), 6.98 (d, *J* = 7.8 Hz, 1.64H), 6.82 (s, 0.83H), 3.55 (s, 2.50H), 3.10 (s, 0.50H). ^{13}C NMR (150 MHz, $CDCl_3$): δ 170.7, 170.6, 141.3, 140.3, 134.1, 133.8, 133.5, 133.3, 132.4, 132.1, 130.6, 130.33, 130.30, 129.7, 129.6, 129.4, 129.3, 129.2, 128.9, 128.4, 128.2, 127.4, 127.2, 126.7, 126.5, 126.1, 125.5, 125.1, 124.3, 124.27, 124.24, 39.5, 36.2. HRMS *m*/*z* (ESI+): Calculated for $C_{18}H_{15}{}^{35}ClNO$ ($[M+H]^+$): 296.0837, found 296.0834.

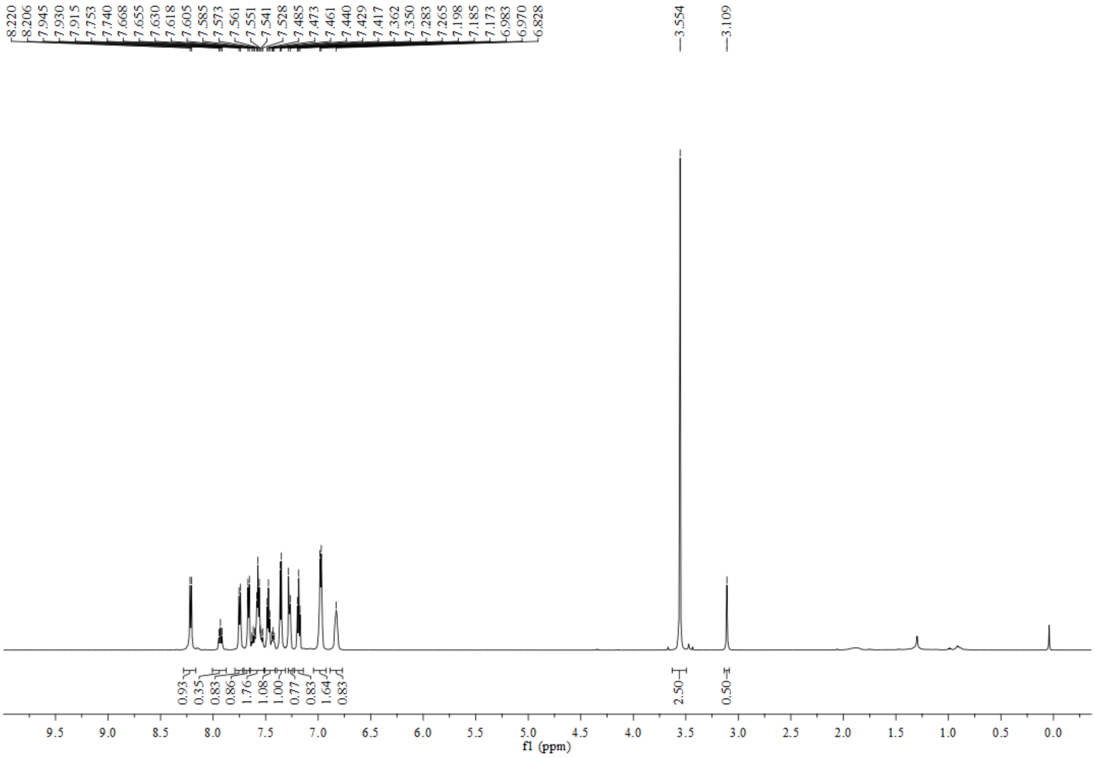

Supplementary Figure 3. ^{1}H NMR Spectra of compound **1a''**.

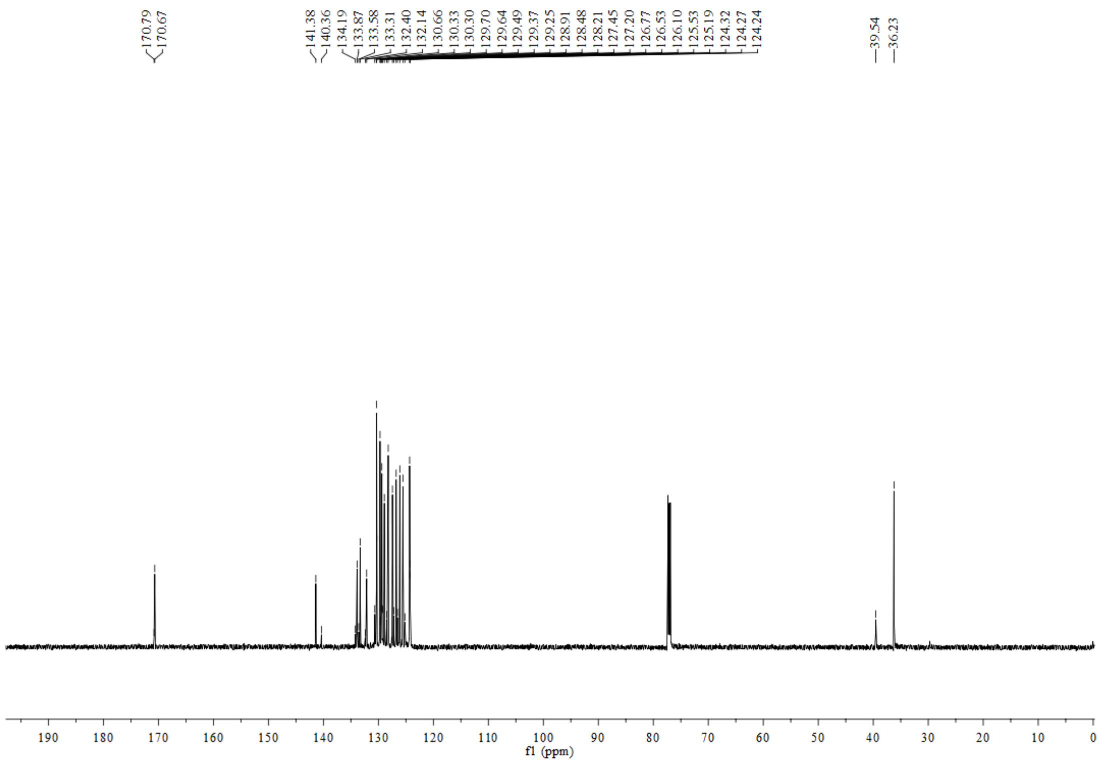

Supplementary Figure 4. ^{13}C NMR Spectra of compound **1a”**.

N-(2-bromo-3-methylphenyl)-*N*-methyl-1-naphthamide (**1b**)

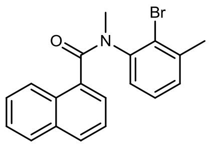

Purified by chromatography on silica gel, eluting with ethyl acetate/petroleum ether 1:10 (*v*/*v*); white solid, Mp = 134-136 ^{o}C, 75% yield; ^{1}H NMR (500 MHz, $CDCl_3$): δ 8.21 (d, *J* = 8.5 Hz, 0.99H), 7.95-7.91 (m, 0.45H), 7.76 (d, *J* = 8.0 Hz, 0.77H), 7.67 (d, *J* = 8.0 Hz, 0.95H), 7.63-7.55 (m, 1.45H), 7.50-7.46 (m, 0.83H), 7.41-7.35 (m, 1.16H), 7.31-7.29 (m, 0.26H), 7.20-7.16 (m, 0.76H), 6.95-6.92 (m, 0.77H), 6.83-6.75 (m, 1.52H), 3.53 (s, 2.30H), 3.10 (s, 0.64H), 2.54 (s, 0.64H), 2.35 (s, 2.35H). ^{13}C NMR (125 MHz, $CDCl_3$): δ 170.4, 143.1, 142.1, 140.2, 139.9, 134.2, 133.9, 133.5, 133.2, 130.3, 130.2, 129.8, 129.6, 129.3, 129.2, 128.3, 128.17, 128.13, 127.2, 127.0, 126.8, 126.6, 126.4, 125.9, 125.6, 125.08, 125.04, 124.2, 124.1, 123.9, 39.5, 36.4, 23.6, 23.5. HRMS *m*/*z* (ESI+): Calculated for $C_{19}H_{17}{}^{79}BrNO$ ($[M+H]^+$): 354.0488, found 354.0488.

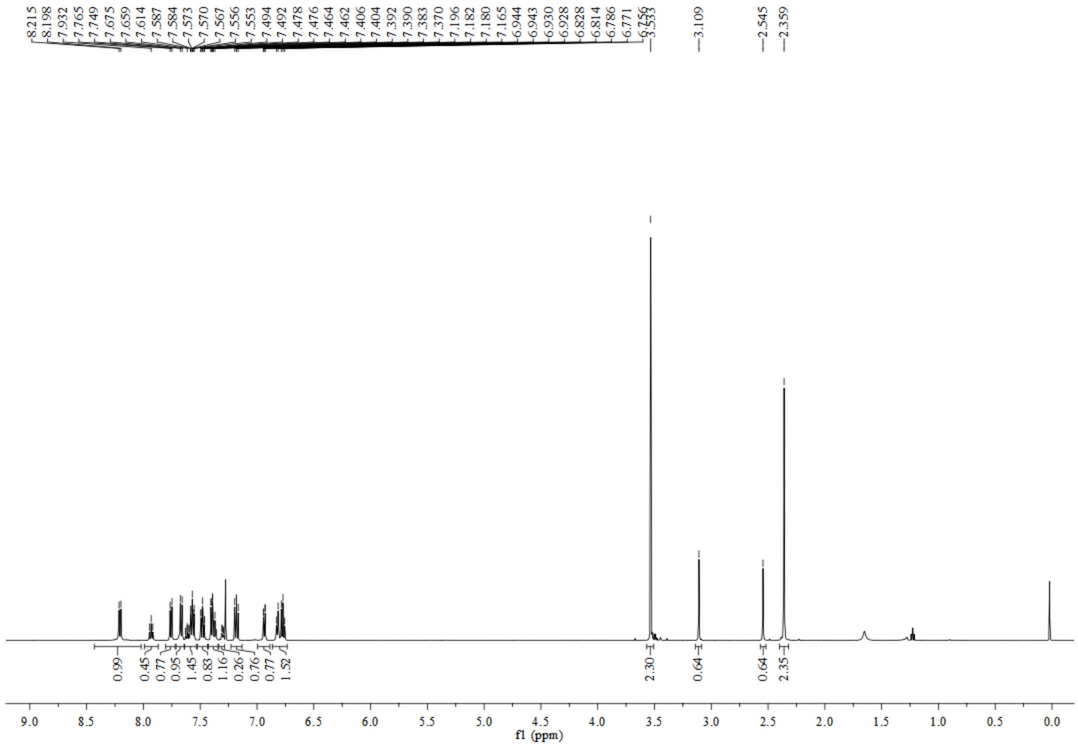

Supplementary Figure 5. ^{1}H NMR Spectra of compound **1b**.

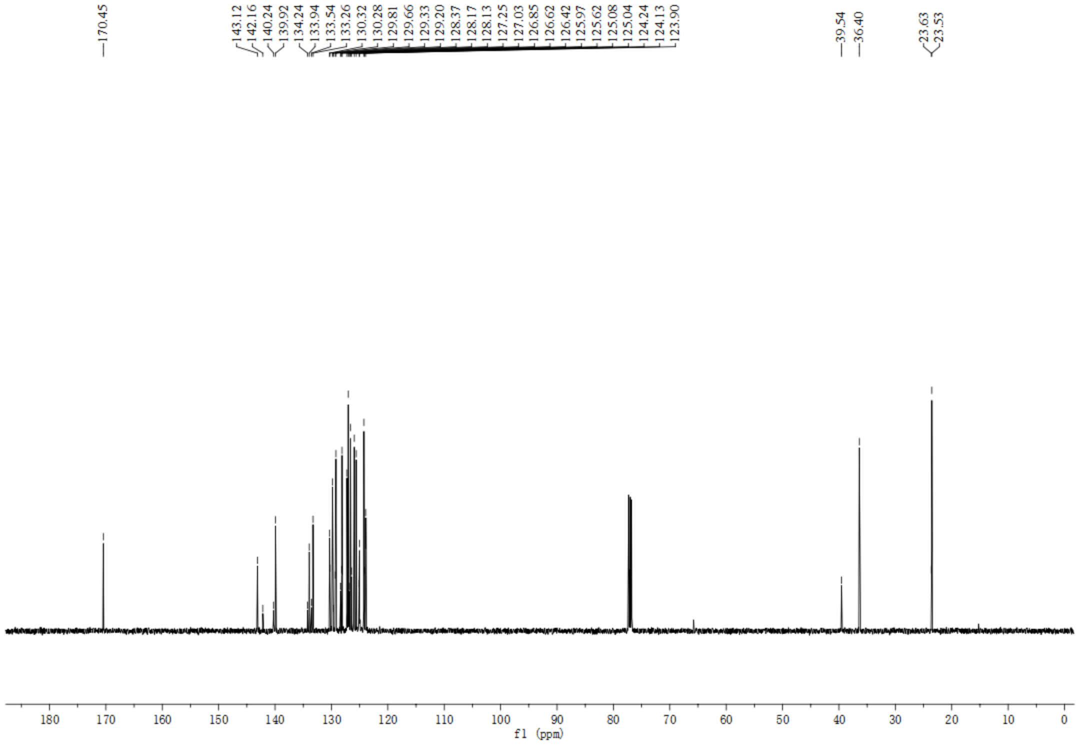

Supplementary Figure 6. ^{13}C NMR Spectra of compound **1b**.

N-(2-bromo-3-methoxyphenyl)-*N*-methyl-1-naphthamide (**1c**)

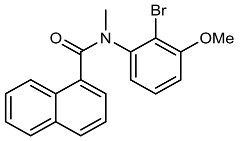

Purified by chromatography on silica gel, eluting with ethyl acetate/petroleum ether 1:10 (*v*/*v*); white solid, Mp = 184-186 ^{o}C, 61% yield; ^{1}H NMR (500 MHz, $CDCl_3$): δ 8.21 (d, *J* = 8.5 Hz, 0.89H), 7.95-7.91 (m, 0.36H), 7.75 (d, *J* = 8.0 Hz, 0.81H), 7.67 (d, *J* = 8.5 Hz, 0.95H), 7.63-7.55 (m, 1.38H), 7.50-7.38 (m, 1.81H), 7.20-7.15 (m, 1.00H), 6.96 (d, *J* = 7.5 Hz, 0.19H), 6.86-6.82 (m, 0.82H), 6.61-6.59 (m, 1.62H), 3.96 (s, 0.52H), 3.81 (s, 2.48H), 3.53 (s, 2.45H), 3.10 (s, 0.51H). ^{13}C NMR (125 MHz, $CDCl_3$): δ 170.3, 157.4, 156.8, 144.2, 143.3, 134.1, 133.8, 133.5, 133.2, 130.3, 129.6, 129.3, 129.2, 128.9, 128.3, 128.11, 128.10, 127.0, 126.6, 126.4, 125.9, 125.5, 125.0, 124.2, 124.1, 123.9, 121.6, 121.3, 112.1, 111.3, 110.6, 56.5, 56.3, 39.4, 36.3. HRMS *m*/*z* (ESI+): Calculated for $C_{19}H_{17}{}^{79}BrNO_2$ ($[M+H]^+$): 370.0437, found 370.0445.

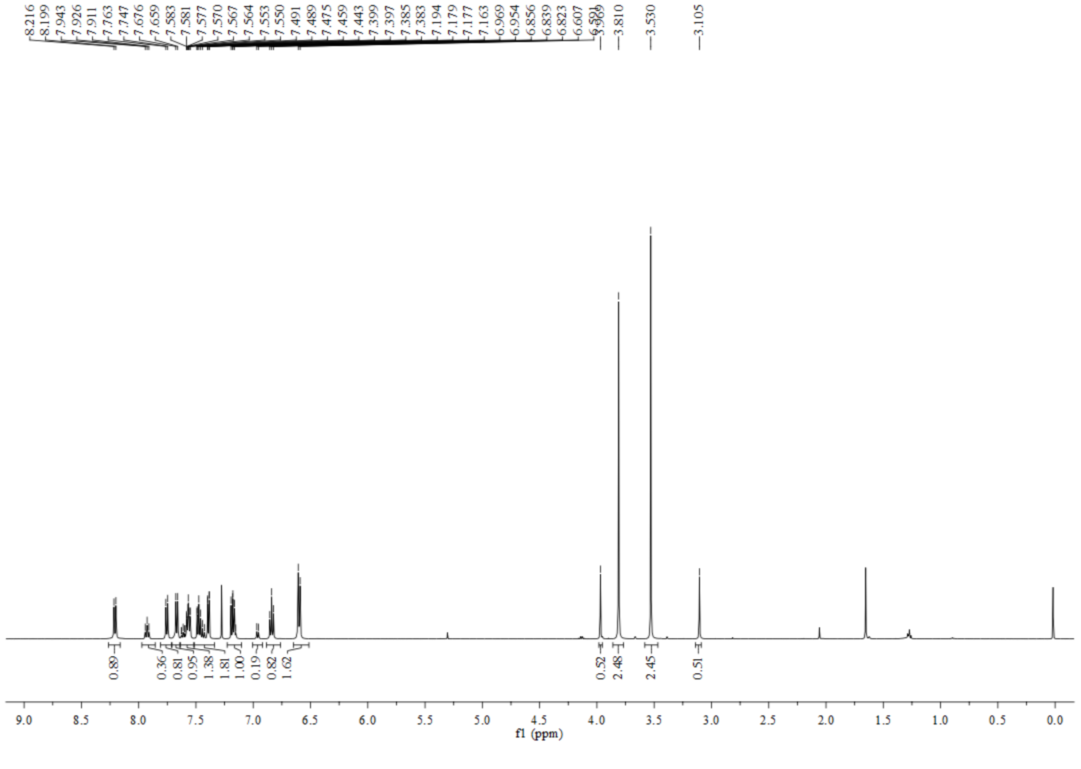

Supplementary Figure 7. ^{1}H NMR Spectra of compound **1c**.

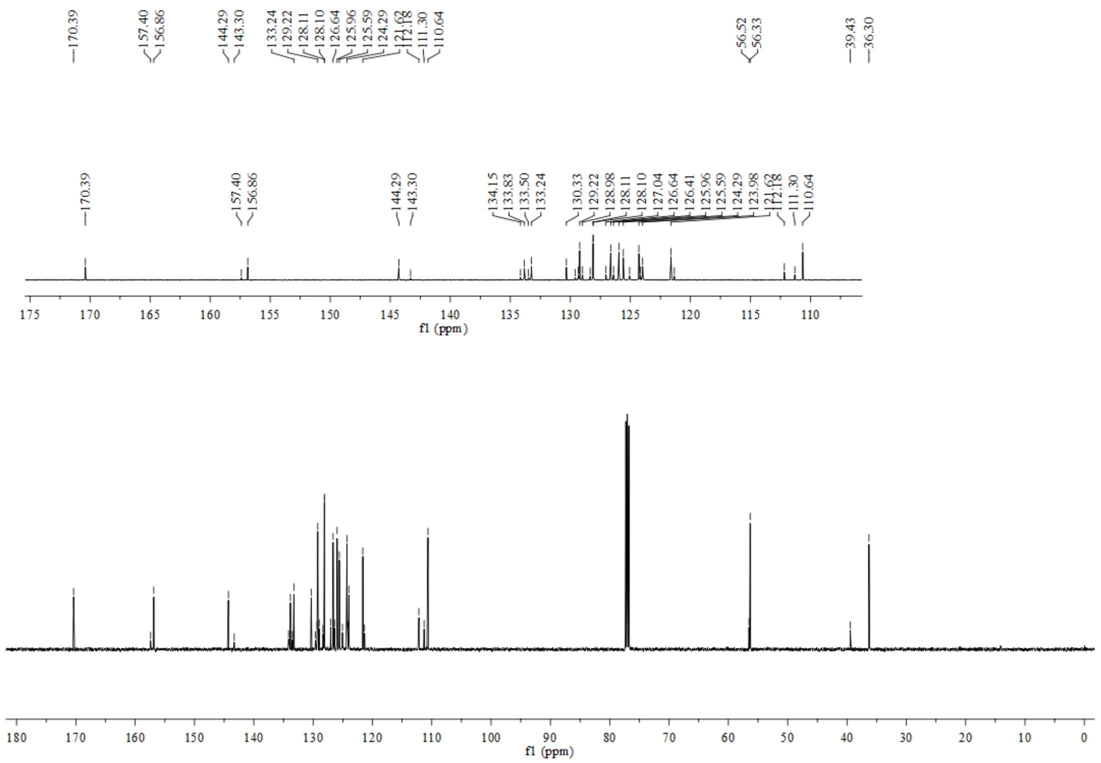

Supplementary Figure 8. ^{13}C NMR Spectra of compound **1c**.

N-(2-bromo-3-fluorophenyl)-*N*-methyl-1-naphthamide (**1d**)

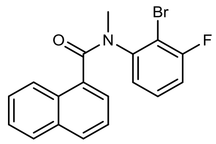

Purified by chromatography on silica gel, eluting with ethyl acetate/petroleum ether 1:10 (*v*/*v*); white solid, Mp = 133-135 °C, 64% yield; ^{1}H NMR (500 MHz, $CDCl_3$): δ 8.19 (d, *J* = 8.5 Hz, 0.94H), 7.97-7.92 (m, 0.4H), 7.77 (d, *J* = 8.5 Hz, 0.77H), 7.71-7.66 (m, 0.95H), 7.64-7.56 (m, 1.40H), 7.51-7.43 (m, 1.00H), 7.37-7.35 (m, 0.99H), 7.22-7.18 (m, 1.00H), 6.88-6.76 (m, 2.32H), 3.54 (s, 2.34H), 3.12 (s, 0.61H). ^{13}C NMR (125 MHz, $CDCl_3$): δ 170.5, 170.3, 160.2 (d, *J* = 247.5 Hz), 159.7 (d, *J* = 247.5 Hz), 144.7 (d, *J* = 1.2 Hz), 143.8, 133.7, 133.5, 133.2, 130.2, 129.58, 129.51, 129.3 (d, *J* = 8.8 Hz), 128.45 (d, *J* = 8.8 Hz), 128.44, 128.2, 127.1, 126.8, 126.5, 126.1, 125.3, 125.2 (d, *J* = 2.5 Hz), 125.0, 124.2, 124.0, 115.9 (d, *J* = 22.5 Hz), 115.4 (d, *J* = 22.5 Hz), 110.1 (d, *J* = 21.3 Hz), 39.4, 36.3. HRMS *m*/*z* (ESI+): Calculated for $C_{18}H_{14}{}^{79}BrFNO$ ($[M+H]^+$): 358.0237, found 358.0237.

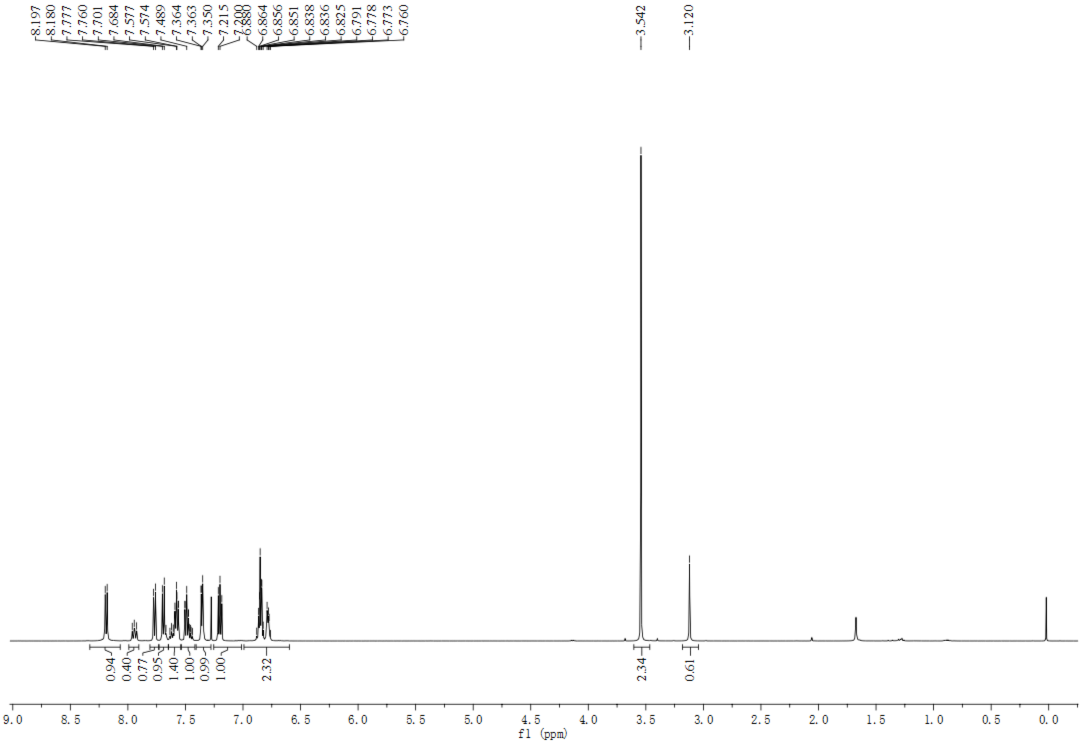

Supplementary Figure 9. ^{1}H NMR Spectra of compound **1d**.

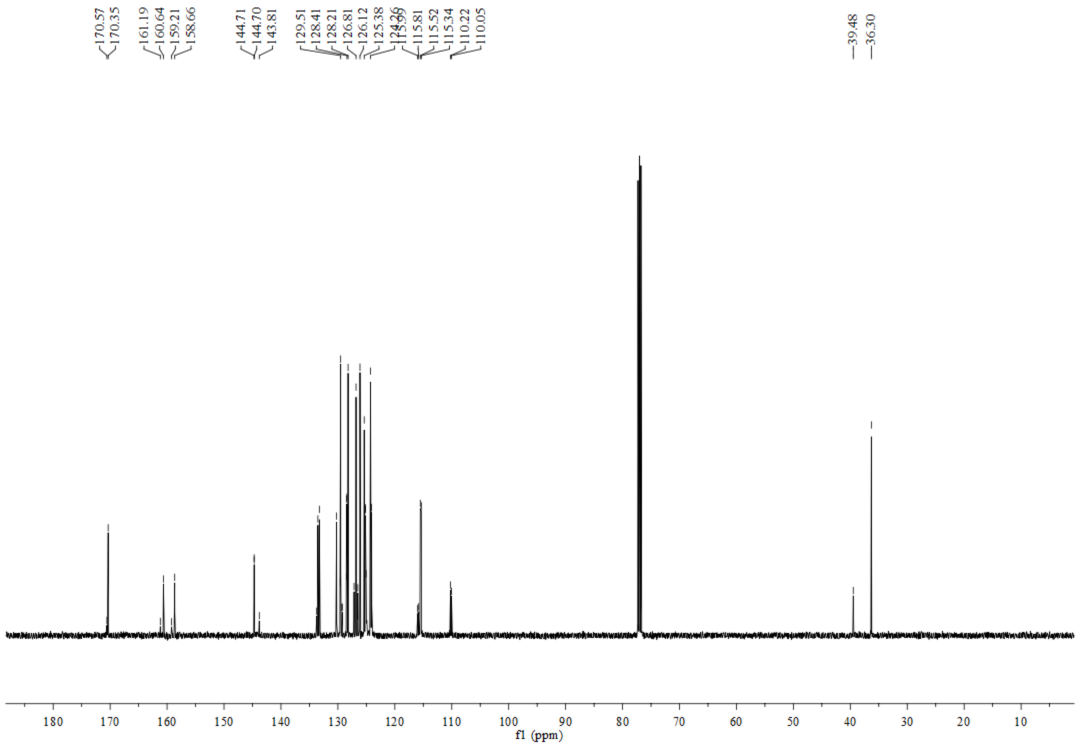

Supplementary Figure 10. ^{13}C NMR Spectra of compound **1d**.

N-(2-bromo-3-(trifluoromethyl)phenyl)-*N*-methyl-1-naphthamide (**1e**)

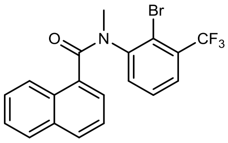

Purified by chromatography on silica gel, eluting with ethyl acetate/petroleum ether 1:10 (*v*/*v*); white solid, Mp = 142-144 ^{o}C, 65% yield; ^{1}H NMR (500 MHz, $CDCl_3$): δ 8.16 (d, *J* = 8.5 Hz, 0.96H), 7.98-7.93 (m, 0.55H), 7.78-7.68 (m, 2.25H), 7.64-7.56 (m, 1.86H), 7.52-7.48 (m, 0.75H), 7.40 (d, *J* = 7.0 Hz, 1.48H), 7.22-7.13 (m, 1.46H), 7.00-6.96 (m, 0.75H), 3.55 (s, 2.20H), 3.13 (s, 0.81H). ^{13}C NMR (125 MHz, $CDCl_3$): δ 170.7, 170.3, 145.0, 144.2, 133.58, 133.53, 133.39, 133.35, 133.2, 132.8, 131.9 (q, *J* = 31.3 Hz), 130.1, 129.7, 129.6, 129.5, 128.7, 128.5, 128.3, 127.9, 127.4 (q, *J* = 5.0 Hz), 127.2, 126.96, 126.94 (q, *J* = 6.3 Hz), 126.5, 126.2, 125.1, 125.0, 124.9 (q, *J* = 3.8 Hz), 124.3, 124.2, 123.8, 123.7, 122.4 (q, *J* = 271.3 Hz) ,121.2, 39.4, 36.3. HRMS *m*/*z* (ESI+): Calculated for $C_{19}H_{14}{}^{79}BrF_3NO$ ($[M+H]^+$): 408.0205, found 408.0209.

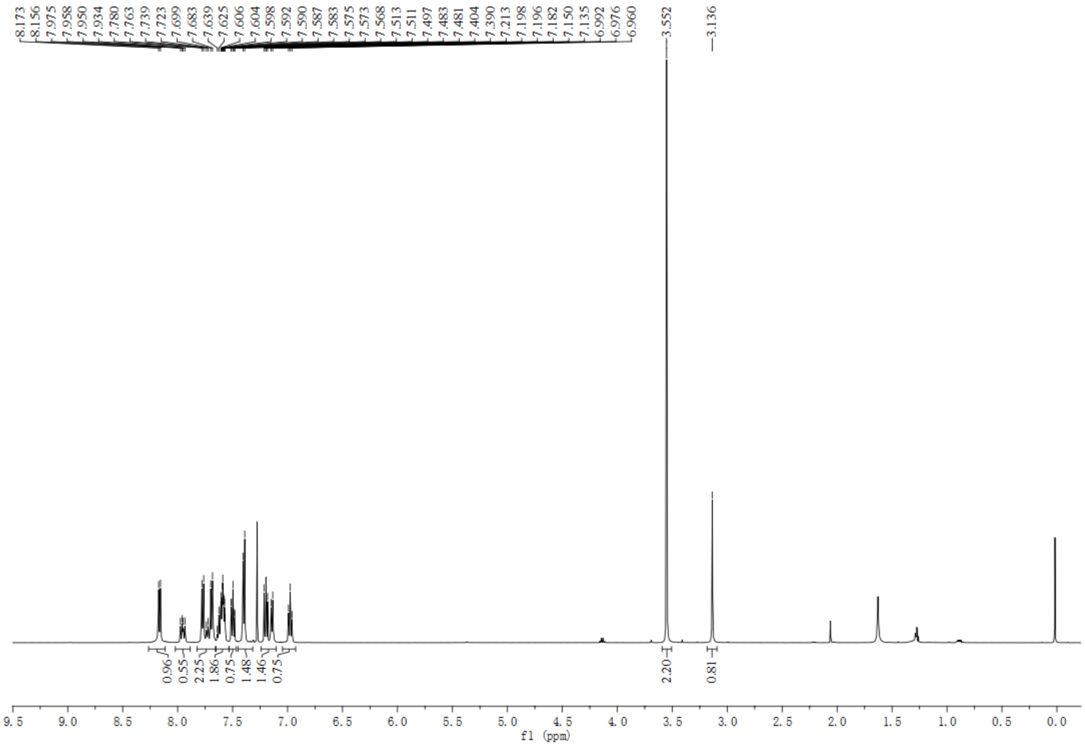

Supplementary Figure 11. ^{1}H NMR Spectra of compound **1e**.

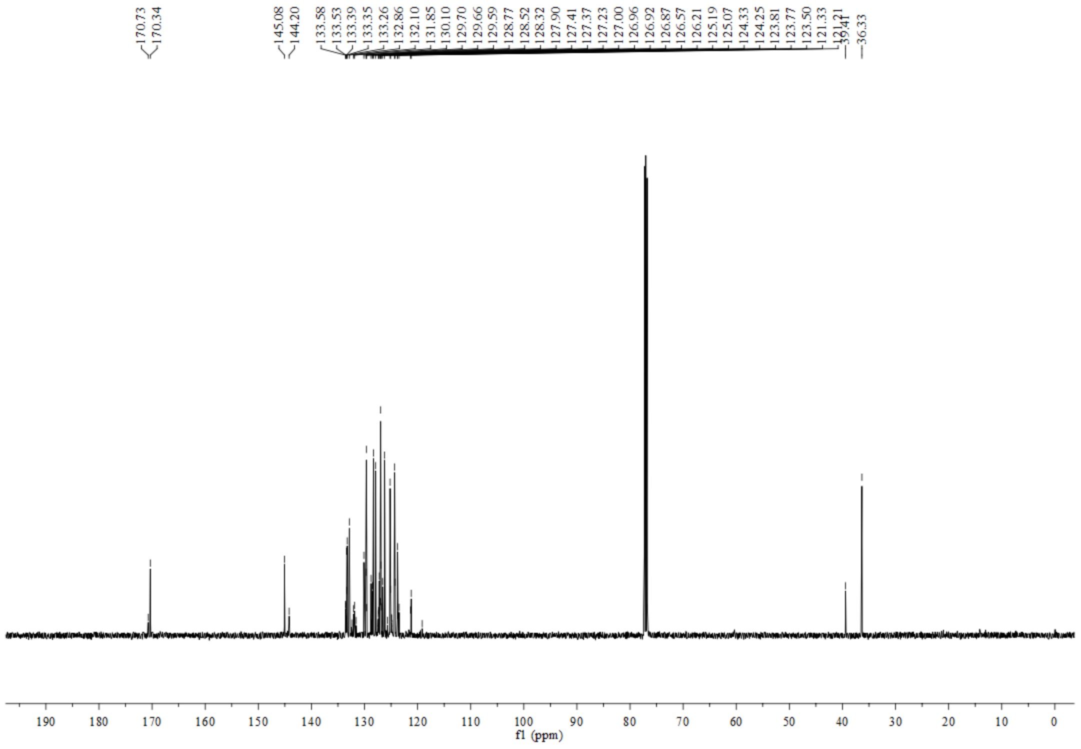

Supplementary Figure 12. ^{13}C NMR Spectra of compound **1e**.

N-(2-bromo-4-methylphenyl)-*N*-methyl-1-naphthamide (**1f**)

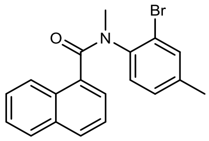

Purified by chromatography on silica gel, eluting with ethyl acetate/petroleum ether 1:10 (*v*/*v*); white solid, Mp = 102-104 ^{o}C, 70% yield; ^{1}H NMR (500 MHz, $CDCl_3$): δ 8.19 (d, J = 8.5 Hz, 0.93H), 7.95-7.91 (m, 0.35H), 7.76 (d, J = 8.5 Hz, 0.80H), 7.69-7.65 (m, 0.95H), 7.63-7.54 (m, 1.51H), 7.50-7.46 (m, 0.83H), 7.42-7.39 (m, 0.96H), 7.29 (d, J = 1.0 Hz, 1.23H), 7.23-7.19 (m, 0.80H), 6.85 (d, J = 8.0 Hz, 0.81H), 6.67 (d, J = 8.0 Hz, 0.80H), 3.51 (s, 2.43H), 3.09 (s, 0.53H), 2.41 (s, 0.53H), 2.13 (s, 2.45H). ^{13}C NMR (125 MHz, $CDCl_3$): δ 170.5, 140.1, 139.6, 139.3, 139.1, 134.1, 134.0, 133.9, 133.7, 133.4, 133.1, 130.2, 129.5, 129.2, 129.18, 129.10, 128.7, 128.3, 128.0, 126.9, 126.5, 126.3, 125.9, 125.5, 125.0, 124.2, 124.09, 124.03, 122.0, 39.5, 36.3, 20.7, 20.3. HRMS *m*/*z* (ESI+): Calculated for $C_{19}H_{17}{}^{79}BrNO$ ($[M+H]^+$): 354.0488, found 354.0489.

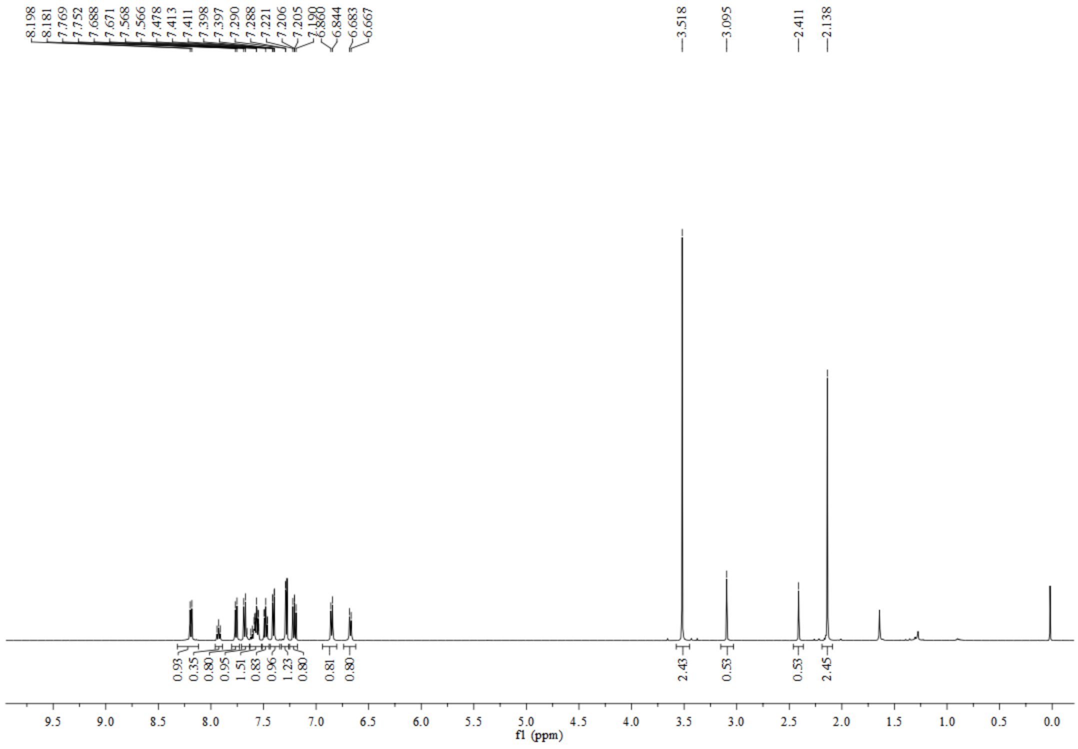

Supplementary Figure 13. ^{1}H NMR Spectra of compound **1f**.

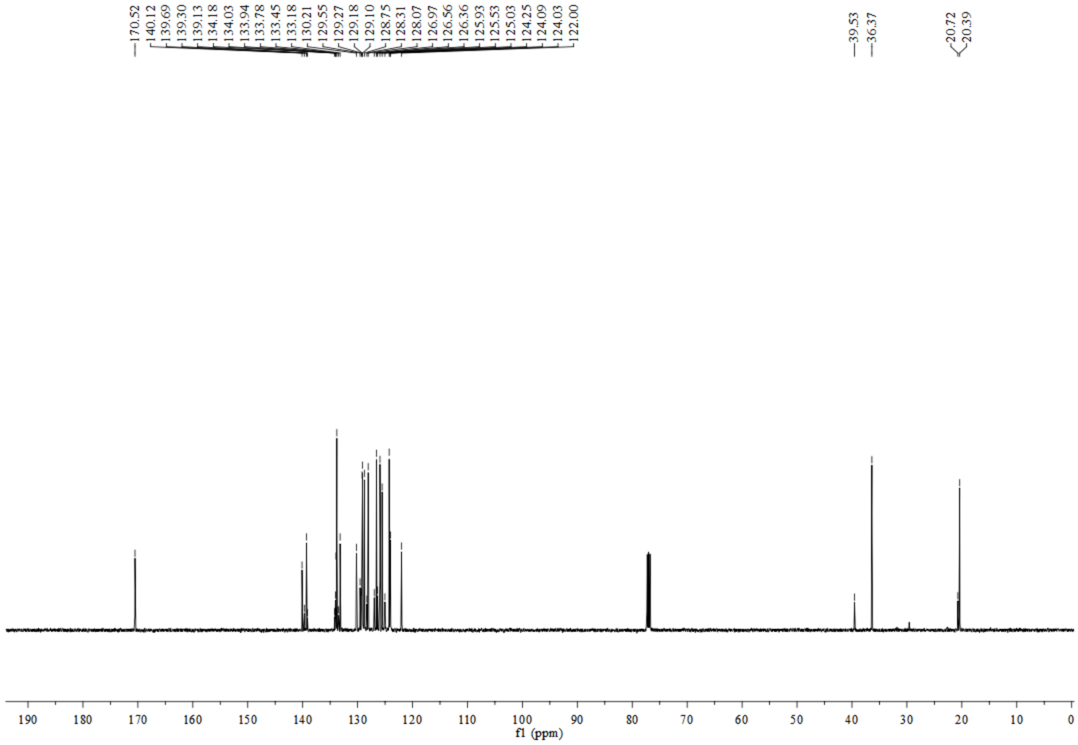

Supplementary Figure 14. ^{13}C NMR Spectra of compound **1f**.

N-(2-bromo-4-methoxyphenyl)-*N*-methyl-1-naphthamide (**1g**)

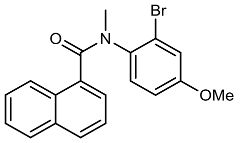

Purified by chromatography on silica gel, eluting with ethyl acetate/petroleum ether 1:5 (*v*/*v*); white solid, Mp = 114-116 °C, 72% yield; ^{1}H NMR (500 MHz, $CDCl_3$): δ 8.17 (d, *J* = 8.0 Hz, 0.95H), 7.94-7.90 (m, 0.36H), 7.76 (d, *J* = 8.5 Hz, 0.85H), 7.69-7.54 (m, 2.34H), 7.49-7.40 (m, 1.89H), 7.29 (d, *J* = 2.5 Hz, 0.19H), 7.24-7.20 (m, 0.82H), 7.02-6.88 (m, 1.84H), 6.41-6.38 (m, 0.80H), 3.85 (s, 0.51H), 3.61 (s, 2.49H), 3.51 (s, 2.54H), 3.08 (s, 0.51H). ^{13}C NMR (125 MHz, $CDCl_3$): δ 170.8, 170.7, 159.4, 158.8, 135.6, 134.5, 134.2, 134.0, 133.4, 133.2, 130.1, 129.9, 129.8, 129.5, 129.2, 129.0, 128.3, 128.1, 126.9, 126.5, 126.3, 125.9, 125.5, 125.0, 124.3, 124.0, 123.9, 122.9, 118.6, 118.3, 114.7, 113.6, 55.7, 55.3, 39.7, 36.5. HRMS *m*/*z* (ESI+): Calculated for $C_{19}H_{17}{}^{79}BrNO_2$ ($[M+H]^+$): 370.0437, found 370.0445.

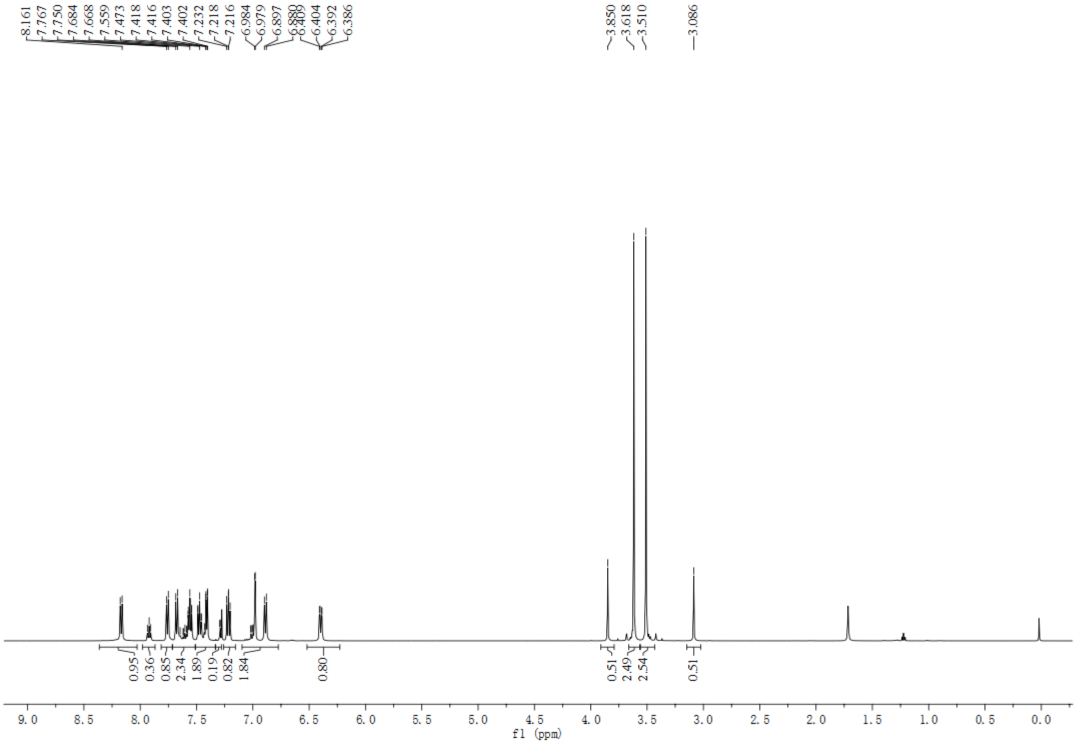

Supplementary Figure 15. ^{1}H NMR Spectra of compound **1g**.

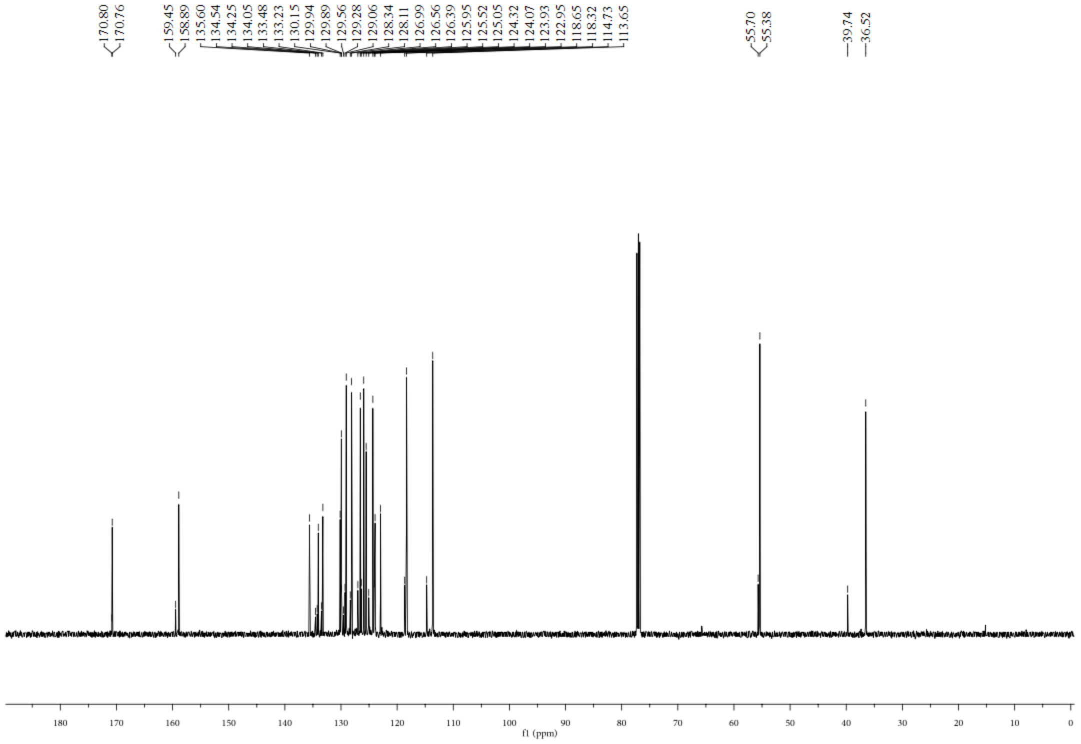

Supplementary Figure 16. ^{13}C NMR Spectra of compound **1g**.

N-(2-bromo-4-(trifluoromethoxy)phenyl)-*N*-methyl-1-naphthamide (**1h**)

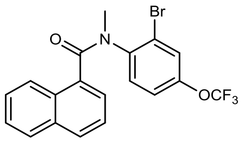

Purified by chromatography on silica gel, eluting with ethyl acetate/petroleum ether 1:10 (*v*/*v*); white solid, Mp = 88-90 ^{o}C, 62% yield; ^{1}H NMR (500 MHz, $CDCl_3$): δ 8.17 (d, *J* = 8.5 Hz, 0.96H), 7.97-7.92 (m, 0.52H), 7.79 (d, *J* = 8.0 Hz, 0.73H), 7.73-7.48 (m, 3.77H), 7.37-7.35 (m, 1.71H), 7.24-7.20 (m, 0.75H), 6.99 (d, *J* = 8.5 Hz, 0.74H), 6.75 (d, *J* = 7.0 Hz, 0.74H), 3.53 (s, 2.23H), 3.11 (s, 0.74H). ^{13}C NMR (125 MHz, $CDCl_3$): δ 170.7, 170.3, 148.4, 147.9, 141.6, 140.7, 133.5, 133.4, 133.3, 130.6, 130.5, 130.1, 129.65, 129.60, 129.5, 128.4, 128.3, 127.1, 126.8, 126.5, 126.2, 126.1, 125.7, 125.3, 125.0, 124.93, 124.91, 124.2, 124.1, 123.1, 121.2, 120.2, 120.0 (q, *J* = 253.8 Hz), 39.4, 36.2. HRMS *m*/*z* (ESI+): Calculated for $C_{19}H_{14}{}^{79}BrF_3NO_2$ ($[M+H]^+$): 424.0155, found 424.0158.

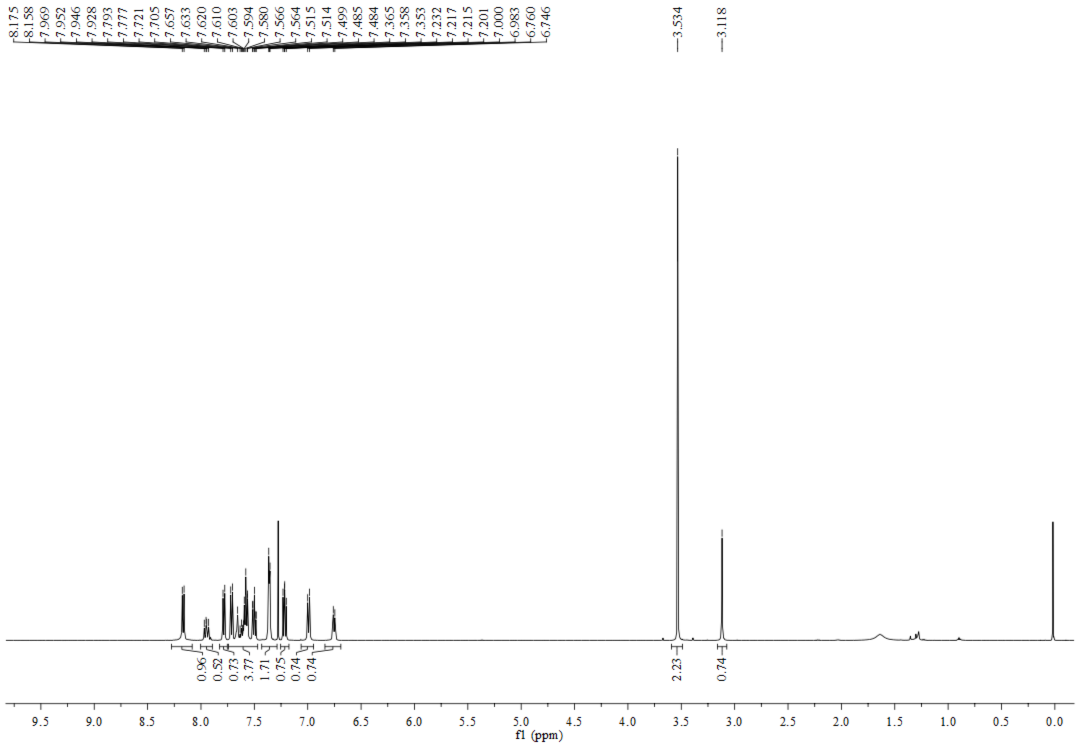

Supplementary Figure 17. ^{1}H NMR Spectra of compound **1h**.

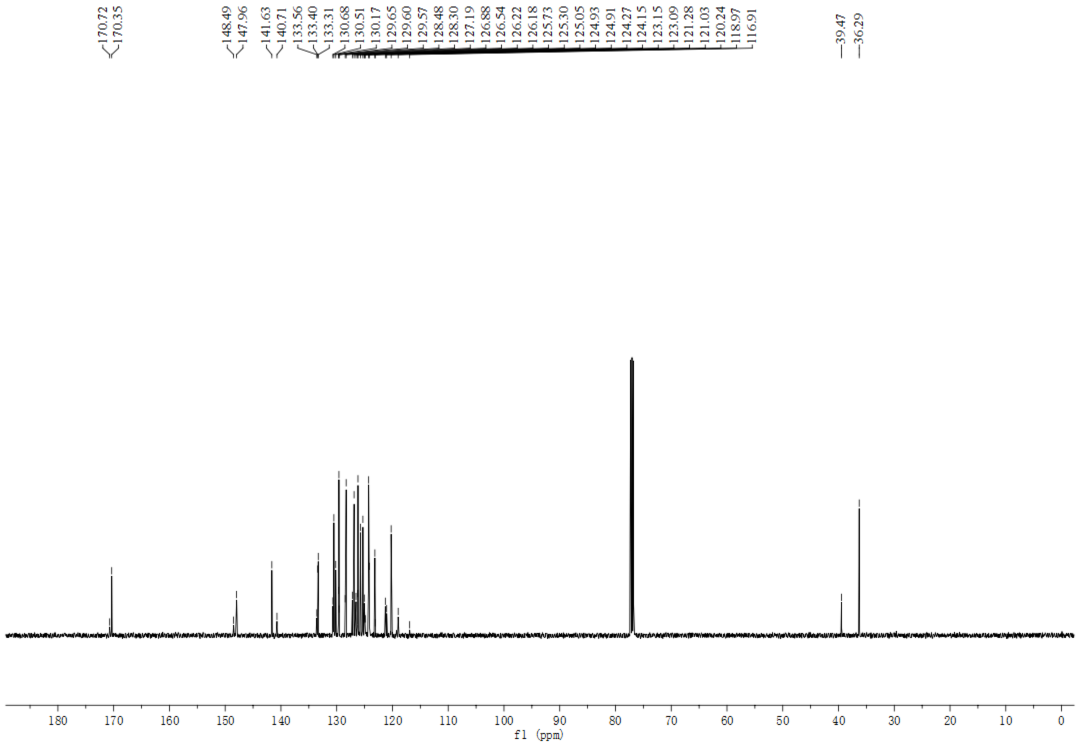

Supplementary Figure 18. ^{13}C NMR Spectra of compound **1h**.

N-(2-bromo-4-fluorophenyl)-*N*-methyl-1-naphthamide (**1i**)

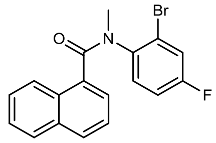

Purified by chromatography on silica gel, eluting with ethyl acetate/petroleum ether 1:10 (*v*/*v*); white solid, Mp = 82-84 °C, 67% yield; ^{1}H NMR (500 MHz, $CDCl_3$): δ 8.16 (d, *J* = 8.5 Hz, 0.99H), 7.96-7.92 (m, 0.41H), 7.77 (d, *J* = 8.5 Hz, 0.79H), 7.70 (d, *J* = 8.5 Hz, 0.80H), 7.67-7.55 (m, 1.68H), 7.53-7.47 (m, 1.23H), 7.40-7.37 (m, 0.81H), 7.24-7.18 (m, 1.77H), 6.98-6.94 (m, 0.80H), 6.62-6.58 (m, 0.80H), 3.52 (s, 2.44H), 3.09 (s, 0.62H). ^{13}C NMR (125 MHz, $CDCl_3$): δ 170.7, 170.5, 161.5 (d, *J* = 250.0 Hz), 160.9 (d, *J* = 251.3 Hz), 139.2 (d, *J* = 3.8 Hz), 138.2 (d, *J* = 2.5 Hz), 133.8, 133.6, 133.5, 133.2, 130.7 (d, *J* = 8.8 Hz), 130.6 (d, *J* = 8.8 Hz), 130.1, 129.56, 129.53, 129.4, 128.4, 128.2, 127.1, 126.7, 126.4, 126.1, 125.3, 125.0, 124.3, 124.1, 123.9, 123.1 (d, *J* = 10.0 Hz), 120.9 (d, *J* = 25.0 Hz), 120.6 (d, *J* = 25.0 Hz), 116.0 (d, *J* = 21.3 Hz), 115.1 (d, *J* = 22.5 Hz), 39.5, 36.3. HRMS *m*/*z* (ESI+): Calculated for $C_{18}H_{14}{}^{79}BrFNO$ ($[M+H]^+$): 358.0237, found 358.0240.

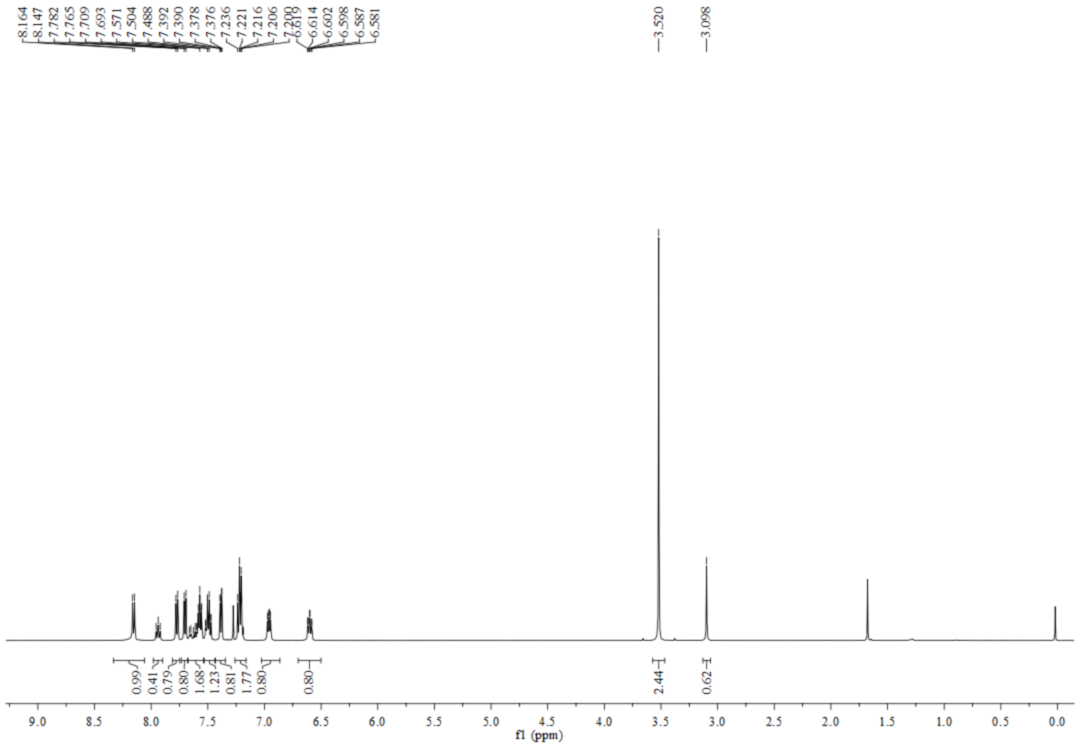

Supplementary Figure 19. ^{1}H NMR Spectra of compound **1i**.

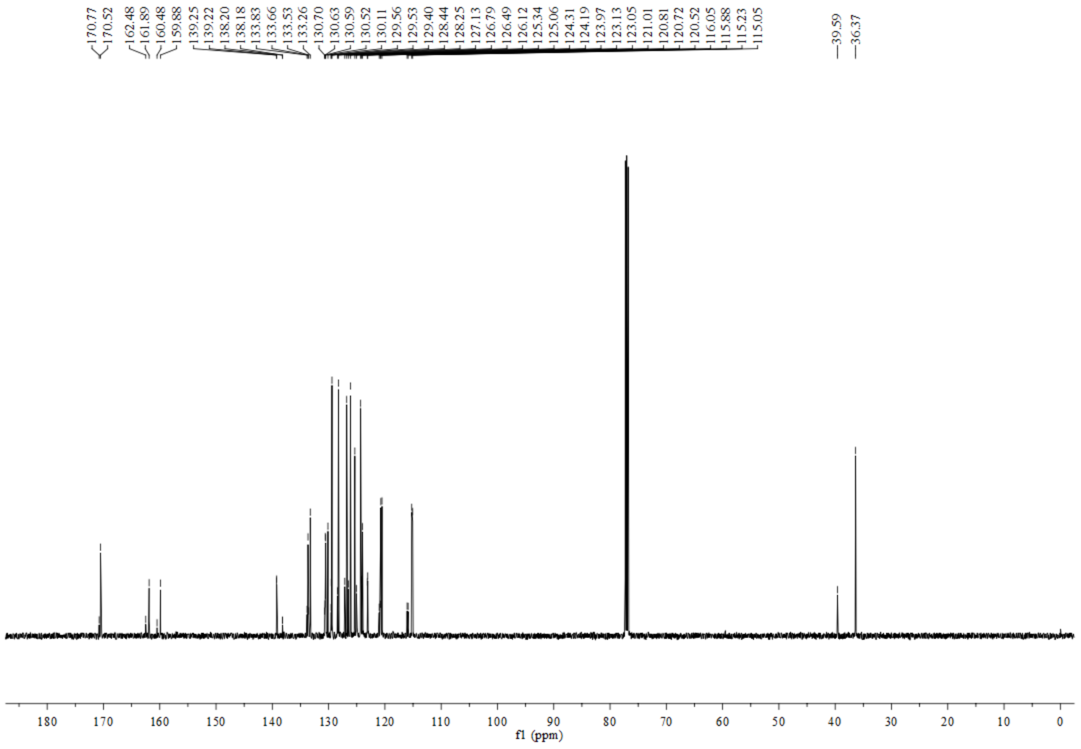

Supplementary Figure 20. ^{13}C NMR Spectra of compound **1i**.

N-(2-bromo-4-chlorophenyl)-*N*-methyl-1-naphthamide (**1j**)

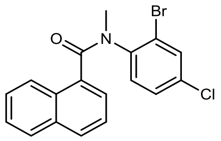

Purified by chromatography on silica gel, eluting with ethyl acetate/petroleum ether 1:10 (*v*/*v*); white solid, Mp = 80-82 ^{o}C, 62% yield; ^{1}H NMR (500 MHz, $CDCl_3$): δ 8.16 (d, *J* = 8.5 Hz, 0.97H), 7.96-7.92 (m, 0.40H), 7.78 (d, *J* = 8.0 Hz, 0.96H), 7.72 (d, *J* = 8.0 Hz, 0.79H), 7.66-7.55 (m, 1.63H), 7.51-7.46 (m, 1.94H), 7.39-7.36 (m, 0.81H), 7.25-7.21 (m, 0.79H), 6.92-6.85 (m, 1.56H), 3.51 (s, 2.40H), 3.09 (s, 0.60H). ^{13}C NMR (125 MHz, $CDCl_3$): δ 170.6, 170.3, 141.61, 140.63, 134.4, 133.9, 133.6, 133.5, 133.4, 133.2, 133.1, 130.5, 130.3, 130.1, 129.58, 129.54, 129.1, 128.4, 128.3, 128.2, 127.1, 126.8, 126.5, 126.1, 125.3, 125.0, 124.3, 124.2, 124.1, 123.0, 39.4, 36.3. HRMS *m*/*z* (ESI+): Calculated for $C_{18}H_{14}{}^{79}Br^{35}ClNO$ ($[M+H]^+$): 373.9942, found 373.9946.

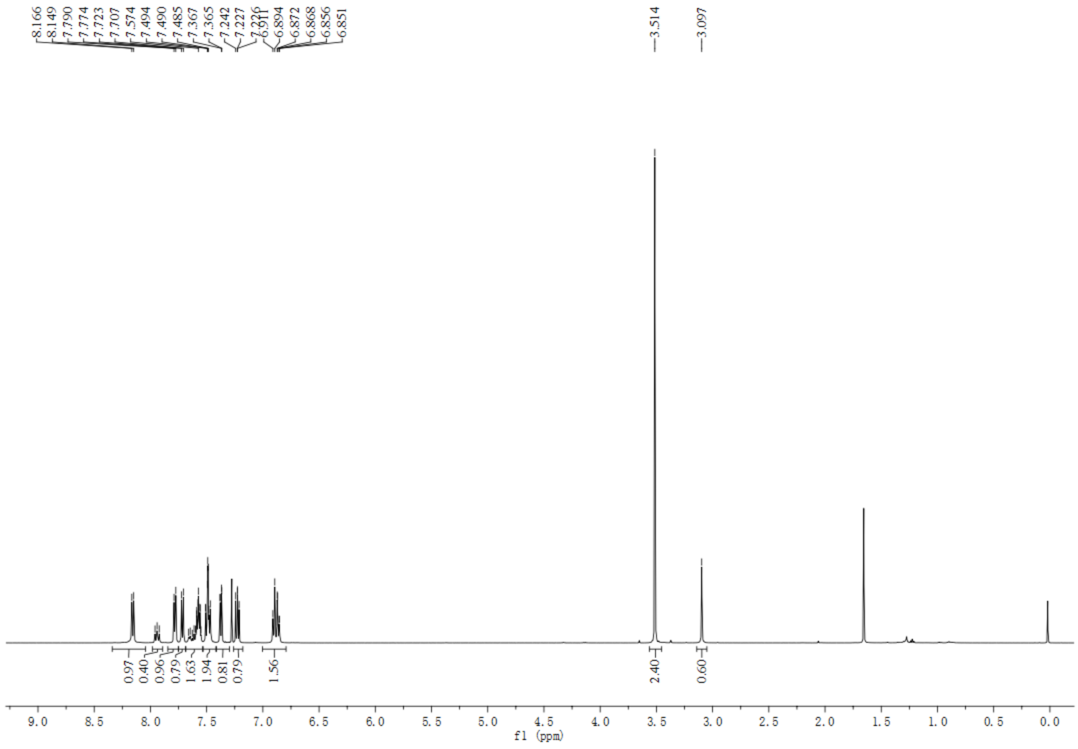

Supplementary Figure 21. ^{1}H NMR Spectra of compound **1j**.

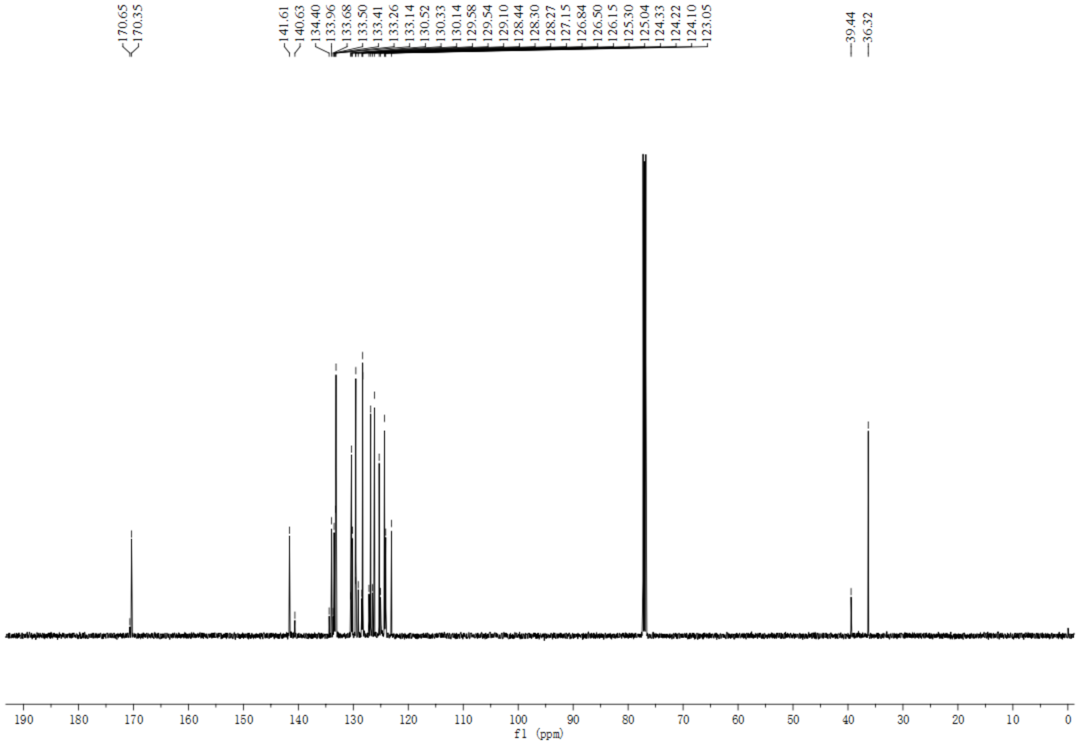

Supplementary Figure 22. ^{13}C NMR Spectra of compound **1j**.

N-(2-bromo-4-(trifluoromethyl)phenyl)-*N*-methyl-1-naphthamide (**1k**)

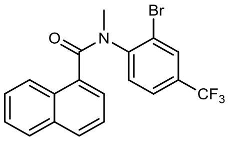

Purified by chromatography on silica gel, eluting with ethyl acetate/petroleum ether 1:10 (*v*/*v*); white solid, Mp = 108-110 ^{o}C, 64% yield; ^{1}H NMR (500 MHz, $CDCl_3$): δ 8.20 (d, *J* = 8.5 Hz, 0.98H), 8.05 (s, 0.23H), 7.98-7.93 (m, 0.50H), 7.80-7.75 (m, 1.72H), 7.73-7.56 (m, 2.81H), 7.53-7.49 (m, 0.80H), 7.35 (d, *J* = 7.0 Hz, 0.75H), 7.23-7.19 (m, 0.77H), 7.16 (d, *J* = 8.0 Hz, 0.75H), 7.07 (d, *J* = 8.0 Hz, 0.77H), 3.54 (s, 2.32H), 3.13 (s, 0.77H). ^{13}C NMR (125 MHz, $CDCl_3$): δ 170.6, 170.1, 146.2, 145.3, 133.5, 133.39, 133.32, 133.2, 130.4, 130.98 (q, *J* = 2.5 Hz), 130.93 (q, *J* = 32.5 Hz), 130.8 (q, *J* = 3.8 Hz), 130.2, 130.1, 129.8, 129.7, 129.5, 128.5, 128.3, 127.2, 127.0, 126.5, 126.2, 125.9, 125.2, 125.1 (q, *J* = 3.8 Hz), 124.9 (q, *J* = 2.5 Hz), 123.1, 122.9, 122.5 (q, *J* = 271.5 Hz), 39.2, 36.2. HRMS *m*/*z* (ESI+): Calculated for $C_{19}H_{14}{}^{79}BrF_3NO$ ($[M+H]^+$): 408.0205, found 408.0216.

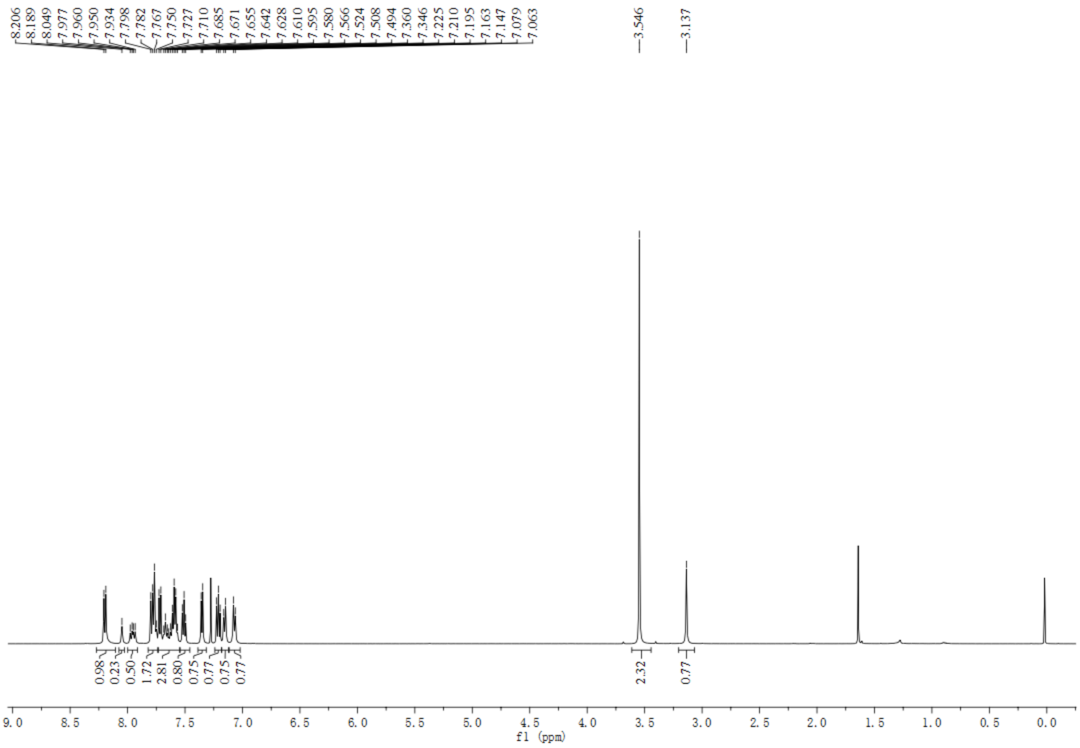

Supplementary Figure 23. ^{1}H NMR Spectra of compound **1k**.

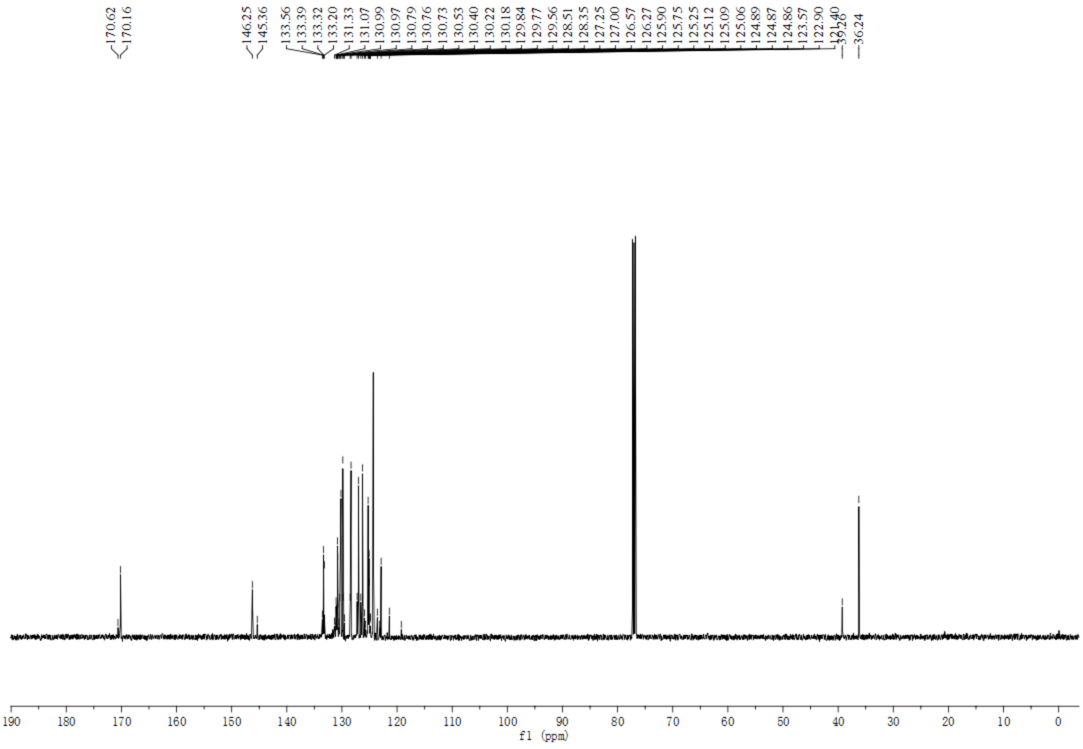

Supplementary Figure 24. ^{13}C NMR Spectra of compound **1k**.

N-(4-acetyl-2-bromophenyl)-*N*-methyl-1-naphthamide (**1l**)

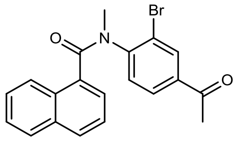

Purified by chromatography on silica gel, eluting with ethyl acetate/petroleum ether 1:5 (*v*/*v*); white solid, Mp = 152-154 ^{o}C, 27% yield; ^{1}H NMR (600 MHz, $CDCl_3$): δ 8.54 (s, 0.16H), 8.37 (s, 0.85H), 8.27 (d, *J* = 9.0 Hz, 0.96H), 8.16 (d, *J* = 8.4 Hz, 0.15H), 8.12 (d, *J* = 9.0 Hz, 0.80H), 8.08 (d, *J* = 8.4 Hz, 0.14H), 7.83-7.76 (m, 1.22H), 7.67-7.64 (m, 0.15H), 7.55-7.47 (m, 2.02H), 7.33-7.26 (m, 0.99H), 6.99-6.89 (m, 2.72H), 3.55 (s, 2.56H), 3.11 (s, 0.44H), 2.76 (s, 0.44H), 2.72 (s, 2.56H). ^{13}C NMR (150 MHz, $CDCl_3$): δ 197.8, 169.8, 142.6, 134.6, 134.2, 133.9, 133.8, 133.6, 132.8, 132.4, 131.8, 131.1, 130.9, 130.3, 130.2, 129.8, 129.7, 129.6, 129.2, 129.0, 128.2, 126.7, 126.6, 126.2, 126.1, 125.3, 125.2, 124.7, 122.6, 39.6, 36.4, 26.7, 26.6. HRMS *m*/*z* (ESI+): Calculated for $C_{20}H_{17}{}^{79}BrNO_2$ ($[M+H]^+$): 382.0437, found 382.0438.

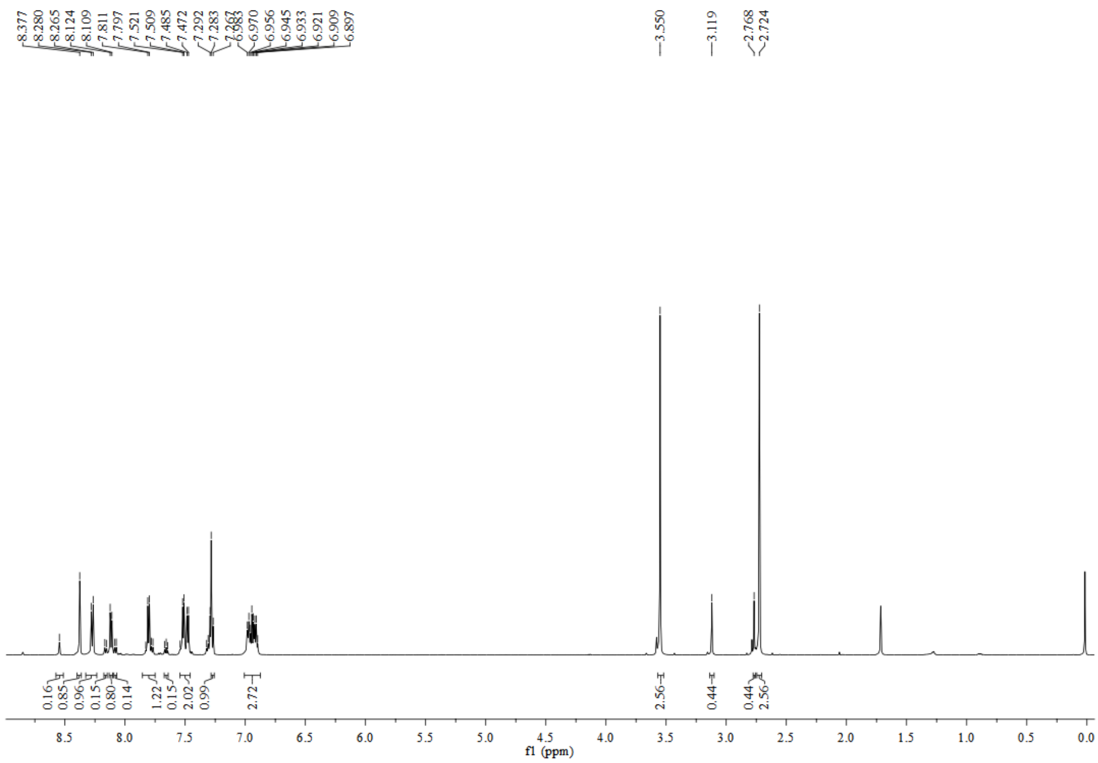

Supplementary Figure 25. ^{1}H NMR Spectra of compound **1l**.

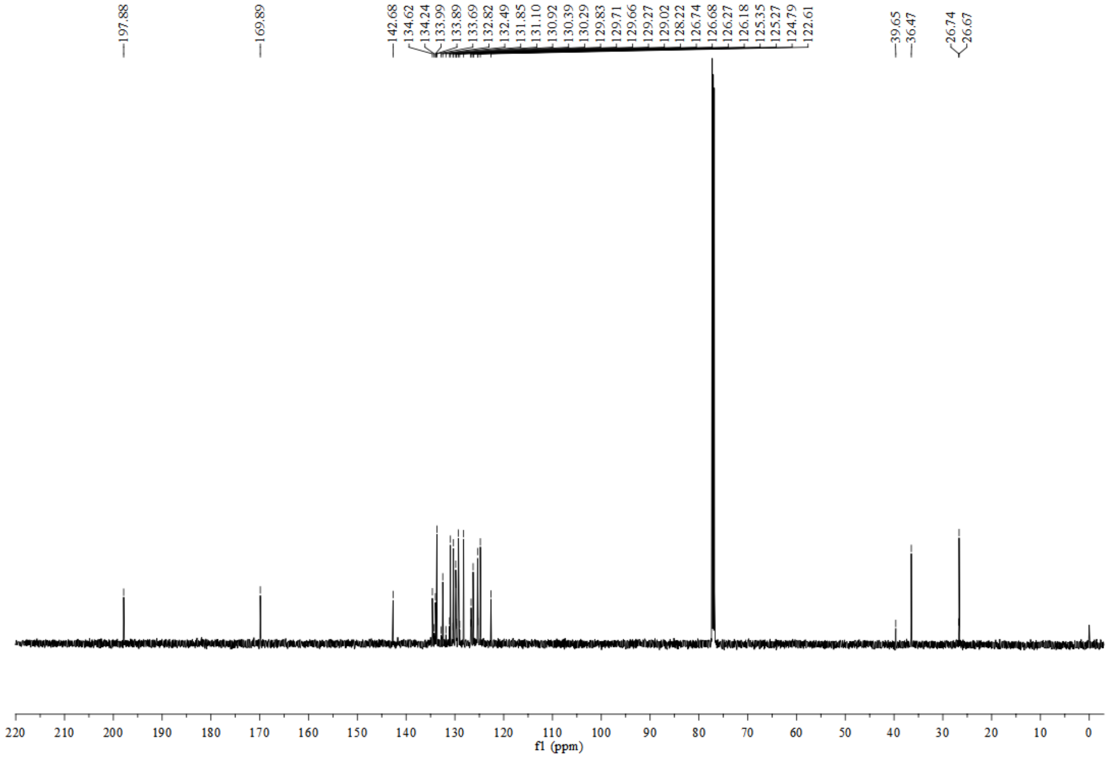

Supplementary Figure 26. ^{13}C NMR Spectra of compound **1l**.

N-(2-bromo-5-methylphenyl)-*N*-methyl-1-naphthamide (**1m**)

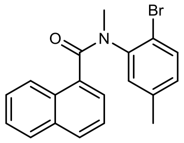

Purified by chromatography on silica gel, eluting with ethyl acetate/petroleum ether 1:10 (*v*/*v*); white solid, Mp = 133-135 °C, 72% yield; ^{1}H NMR (500 MHz, $CDCl_3$): δ 8.21-8.13 (m, 0.95H), 7.95-7.91 (m, 0.48H), 7.75 (d, *J* = 8.5 Hz, 0.76H), 7.66 (d, *J* = 8.0 Hz, 0.97H), 7.63-7.54 (m, 1.78H), 7.50-7.45 (m, 0.79H), 7.43-7.40 (m, 0.74H), 7.35-7.27 (m, 1.16H), 7.22-7.18 (m, 0.76H), 7.11-7.08 (m, 0.25H), 6.81 (s, 0.75H), 6.72-6.69 (m, 0.74H), 3.52 (s, 2.29H), 3.09 (s, 0.73H), 2.42 (s, 0.73H), 1.90 (s, 2.27H). ^{13}C NMR (125 MHz, $CDCl_3$): δ 170.4, 142.3, 141.5, 139.0, 138.1, 134.1, 133.9, 133.4, 133.2, 133.1, 132.9, 130.4, 130.3, 130.2, 129.8, 129.5, 129.3, 129.1, 128.3, 128.0, 127.0, 126.4, 126.3, 125.9, 125.5, 125.0, 124.2, 124.1, 124.0, 118.9, 39.5, 36.2, 20.8, 20.2. HRMS *m*/*z* (ESI+): Calculated for $C_{19}H_{17}{}^{79}BrNO$ ($[M+H]^+$): 354.0488, found 354.0488.

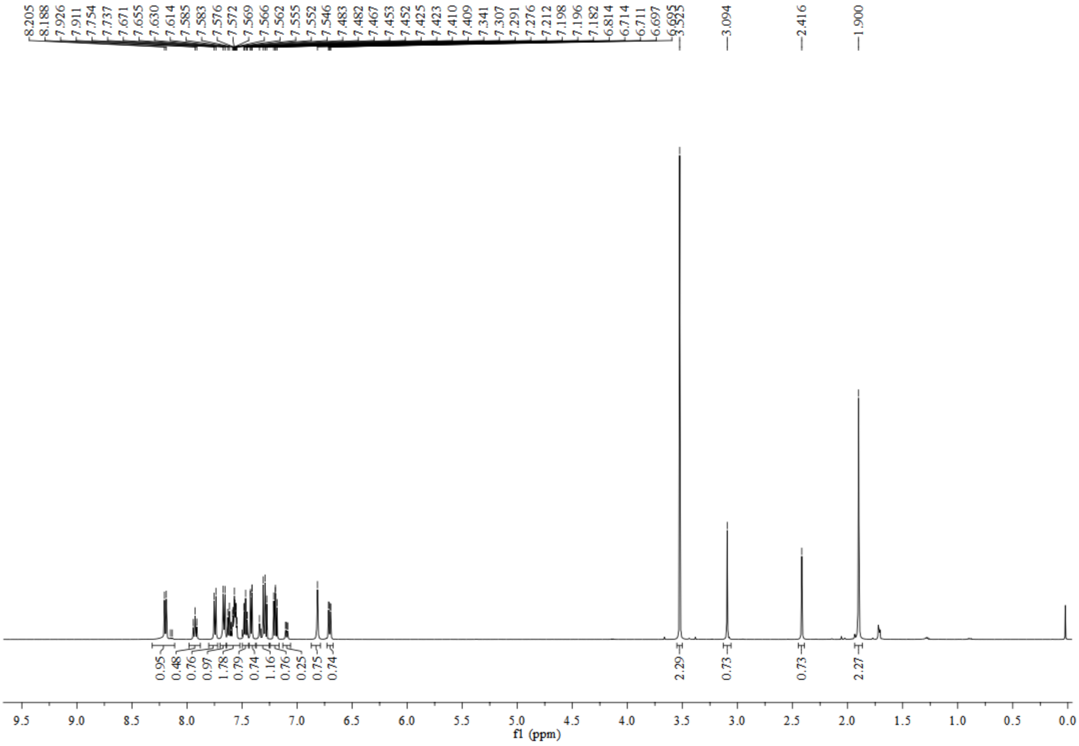

Supplementary Figure 27. ^{1}H NMR Spectra of compound **1m**.

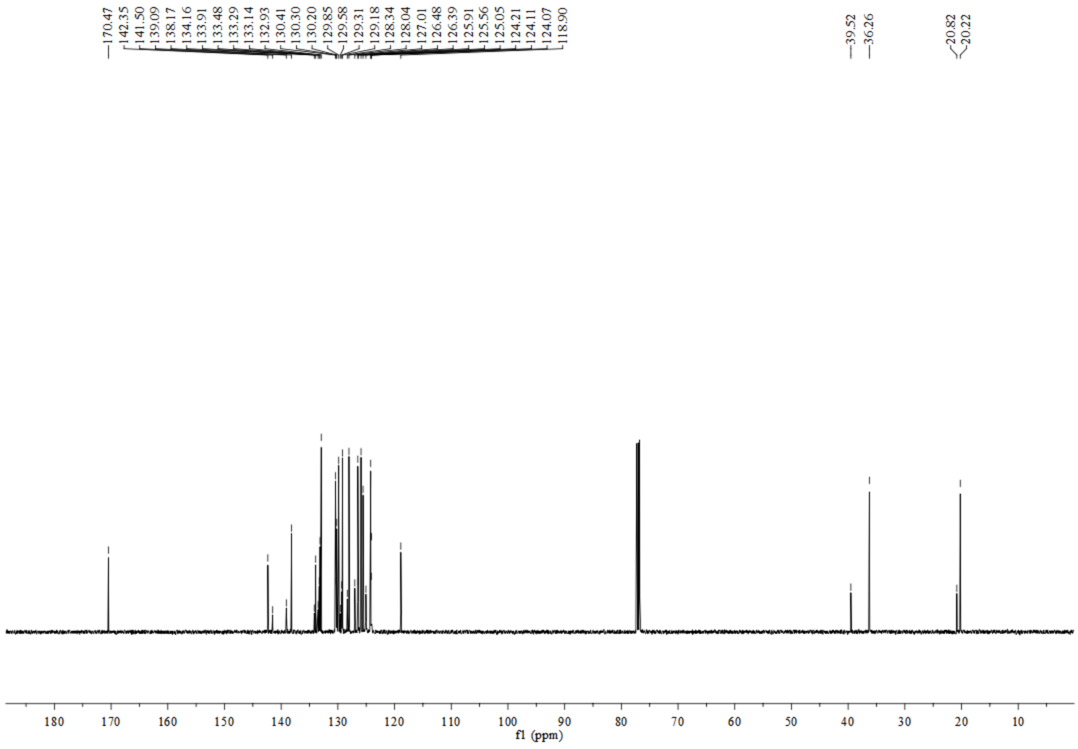

Supplementary Figure 28. ^{13}C NMR Spectra of compound **1m**.

N-(2-bromo-5-methoxyphenyl)-*N*-methyl-1-naphthamide (**1n**)

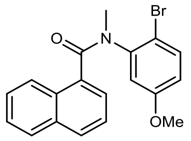

Purified by chromatography on silica gel, eluting with ethyl acetate/petroleum ether 1:5 (*v*/*v*); white solid, Mp = 122-124 ^{o}C, 77% yield; ^{1}H NMR (500 MHz, $CDCl_3$): δ 8.18 (d, *J* = 8.5 Hz, 0.92H), 7.95-7.91 (m, 0.33H), 7.77 (d, *J* = 8.5 Hz, 0.83H), 7.70-7.55 (m, 2.50H), 7.50-7.46 (m, 0.84H), 7.42-7.39 (m, 0.82H), 7.34-7.30 (m, 0.84H), 7.24-7.20 (m, 0.84H), 7.06 (d, *J* = 2.5 Hz, 0.16H), 6.88-6.85 (m, 0.16H), 6.50-6.47 (m, 1.63H), 3.88 (s, 0.50H), 3.53 (s, 2.48H), 3.25 (s, 2.48H), 3.10 (s, 0.52H). ^{13}C NMR (125 MHz, $CDCl_3$): δ 170.5, 160.0, 158.9, 143.3, 142.5, 134.0, 133.9, 133.6, 133.5, 133.1, 130.3, 129.6, 129.4, 129.2, 128.4, 128.2, 127.0, 126.6, 126.4, 125.9, 125.3, 125.0, 124.4, 124.2, 123.8, 115.9, 115.39, 115.35, 114.6, 112.5,

55.6, 55.1, 39.5, 36.1. HRMS *m/z* (ESI+): Calculated for $C_{19}H_{17}{}^{79}BrNO_2$ ($[M+H]^+$): 370.0437, found 370.0437.

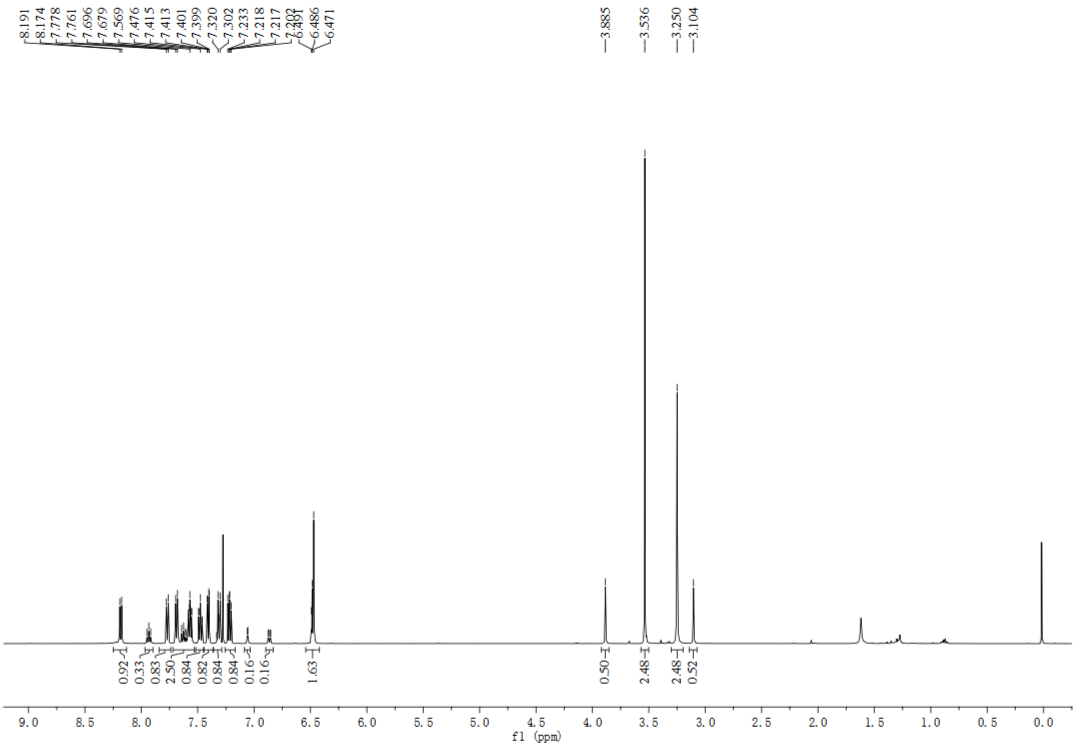

Supplementary Figure 29. ^{1}H NMR Spectra of compound **1n**.

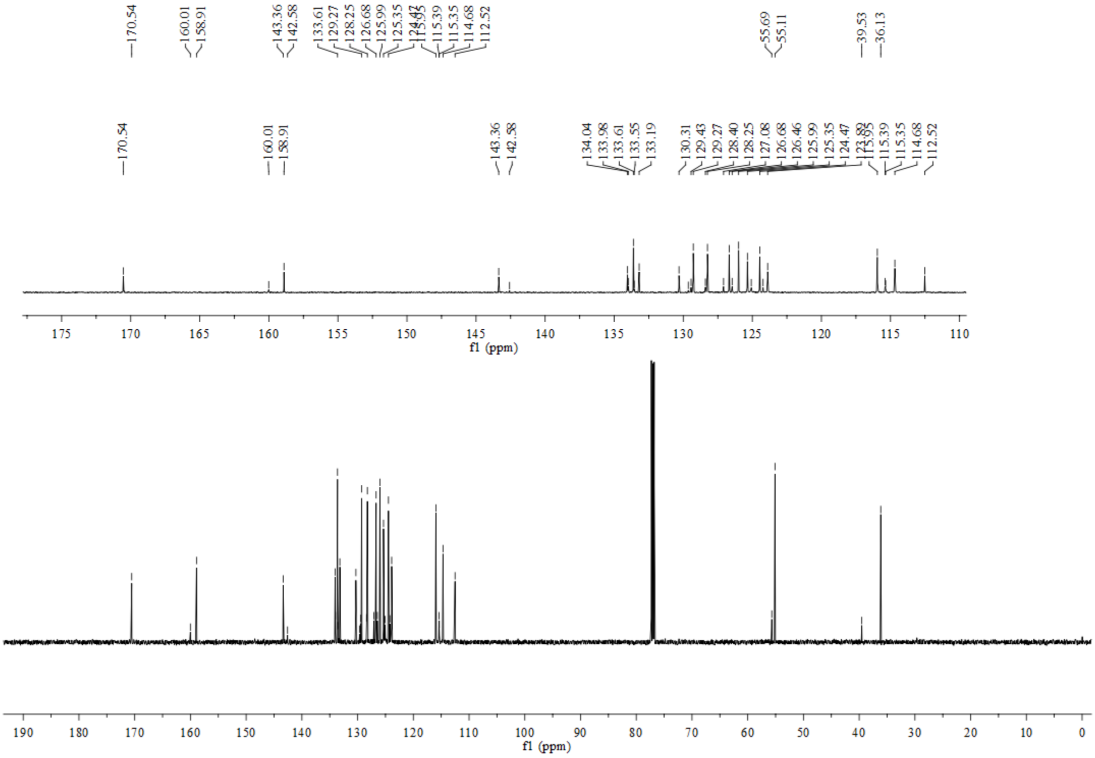

Supplementary Figure 30. ^{13}C NMR Spectra of compound **1n**.

N-(2-bromo-5-(trifluoromethoxy)phenyl)-*N*-methyl-1-naphthamide (**1o**)

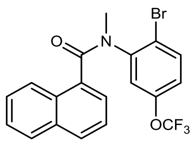

Purified by chromatography on silica gel, eluting with ethyl acetate/petroleum ether 1:10 (*v*/*v*); white solid, Mp = 100-102 °C, 70% yield; ^{1}H NMR (500 MHz, $CDCl_3$): δ 8.17-8.13 (m, 0.96H), 7.97-7.92 (m, 0.43H), 7.80-7.75 (m, 1.01H), 7.73-7.45 (m, 4.08H), 7.41-7.35 (m, 0.99H), 7.22-7.18 (m, 0.99H), 6.87-6.77 (m, 1.53H), 3.54 (s, 2.39H), 3.12 (s, 0.62H). ^{13}C NMR (125 MHz, $CDCl_3$): δ 170.5, 170.3, 149.0, 148.0, 147.9, 143.9, 143.3, 14.6, 134.3, 133.59, 133.53, 133.38, 133.31, 130.0, 129.7, 129.6, 128.5, 128.2, 127.2, 126.9, 126.5, 126.2, 125.1, 124.3, 124.2, 124.1, 122.6, 122.5, 121.7, 120.6, 120.5, 119.8 (q, *J* = 257.5 Hz), 39.4, 36.1. HRMS *m*/*z* (ESI+): Calculated for $C_{19}H_{14}{}^{79}BrF_3NO_2$ ($[M+H]^+$): 424.0155, found 424.0159.

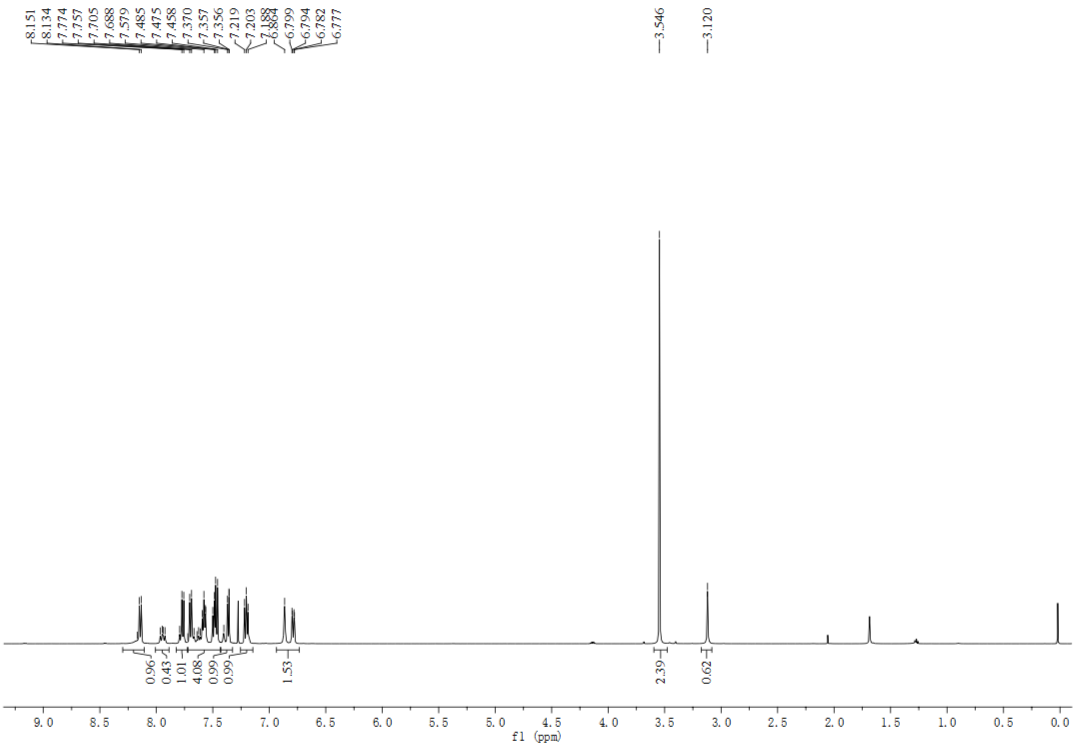

Supplementary Figure 31. ^{1}H NMR Spectra of compound **1o**.

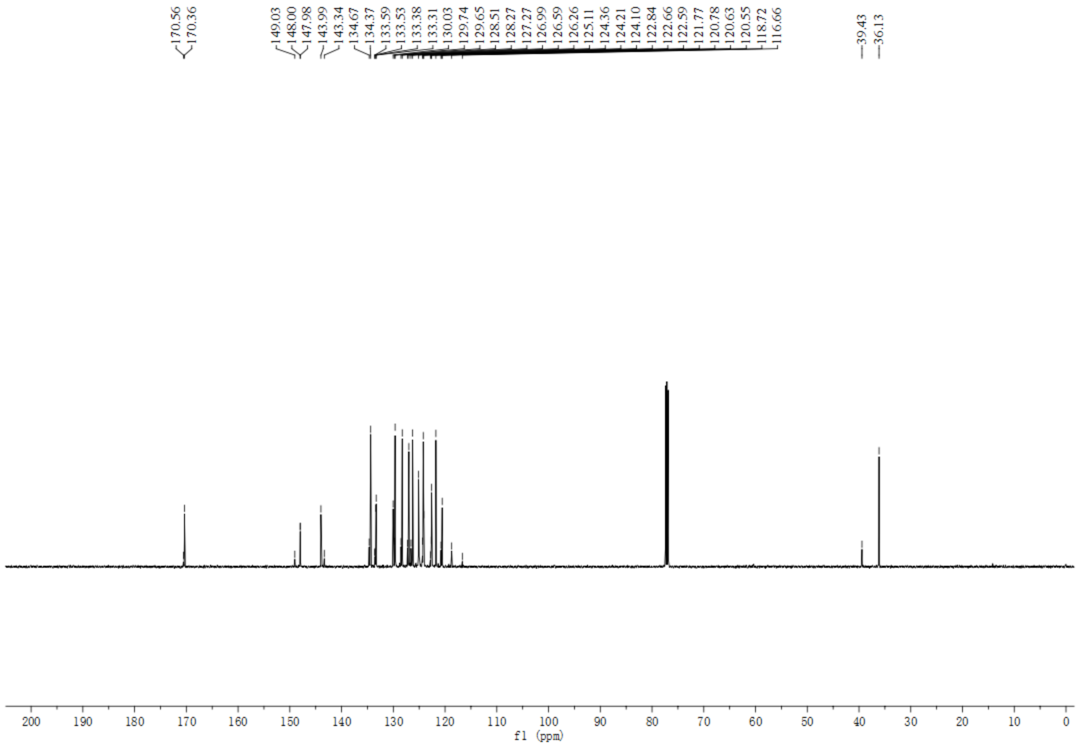

Supplementary Figure 32. ^{13}C NMR Spectra of compound **1o**.

N-(2-bromo-5-fluorophenyl)-*N*-methyl-1-naphthamide (**1p**)

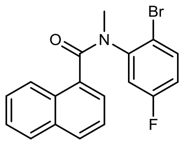

Purified by chromatography on silica gel, eluting with ethyl acetate/petroleum ether 1:10 (*v*/*v*); white solid, Mp = 124-126 ^{o}C, 76% yield; ^{1}H NMR (500 MHz, $CDCl_3$): δ 8.17 (d, *J* = 8.5 Hz, 0.94H), 7.97-7.92 (m, 0.49H), 7.79-7.70 (m, 1.71H), 7.68-7.55 (m, 1.75H), 7.52-7.48 (m, 0.77H), 7.44-7.39 (m, 1.49H), 7.30-7.27 (m, 0.26H), 7.24-7.20 (m, 0.77H), 7.08-7.03 (m, 0.25H), 6.76-6.66 (m, 1.47H), 3.52 (s, 2.25H), 3.10 (s, 0.73H). ^{13}C NMR (125 MHz, $CDCl_3$): δ 170.4, 170.1, 162.2 (d, *J* = 248.8 Hz), 161.2 (d, *J* = 248.8 Hz), 144.0 (d, *J* = 10.0 Hz), 143.1 (d, *J* = 8.8 Hz), 134.5 (d, *J* = 8.8 Hz), 134.2 (d, *J* = 8.8 Hz), 133.5 (d, *J* = 8.8 Hz), 133.3 (d, *J* = 8.8 Hz), 130.1, 129.59, 129.50, 128.4, 128.2, 127.1, 126.8, 126.4, 126.1, 125.2, 124.9, 124.8, 124.1, 117.3, 117.15, 117.14 (d, *J* = 23.8 Hz), 117.12, 116.8 (d, *J* = 22.5 Hz), 116.4 (d, *J* = 22.5 Hz), 39.2, 36.1. HRMS *m/z* (ESI+): Calculated for $C_{18}H_{14}{}^{79}BrFNO$ ($[M+H]^+$): 358.0237,

found 358.0242.

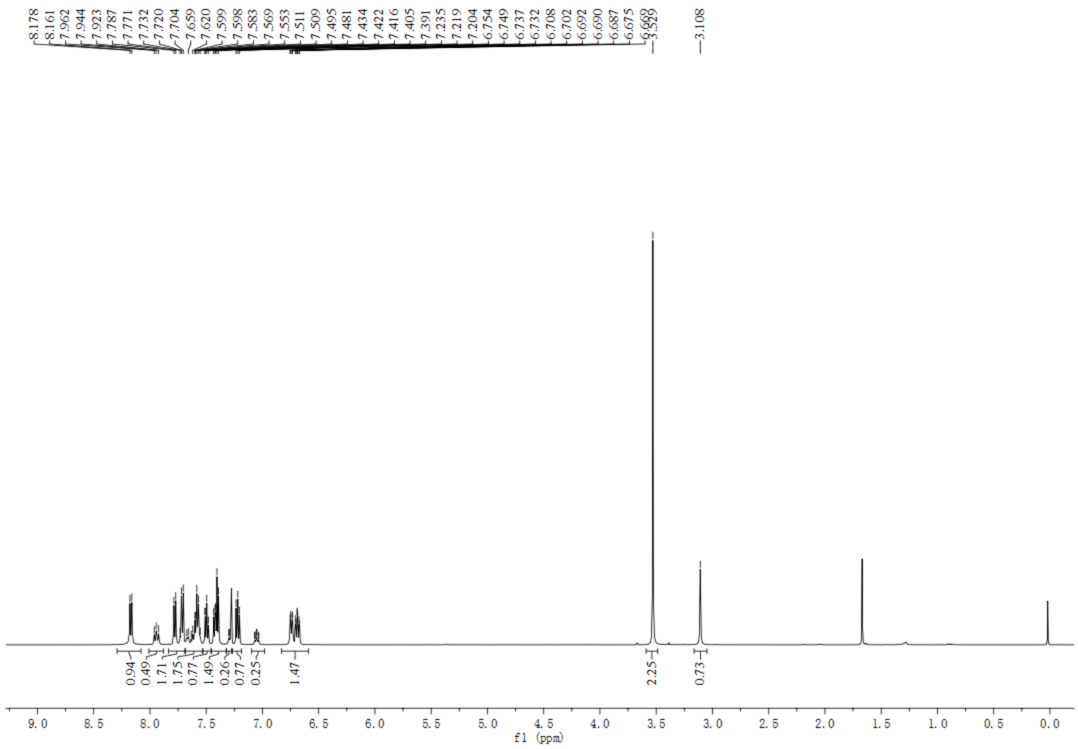

Supplementary Figure 33. ^{1}H NMR Spectra of compound **1p**.

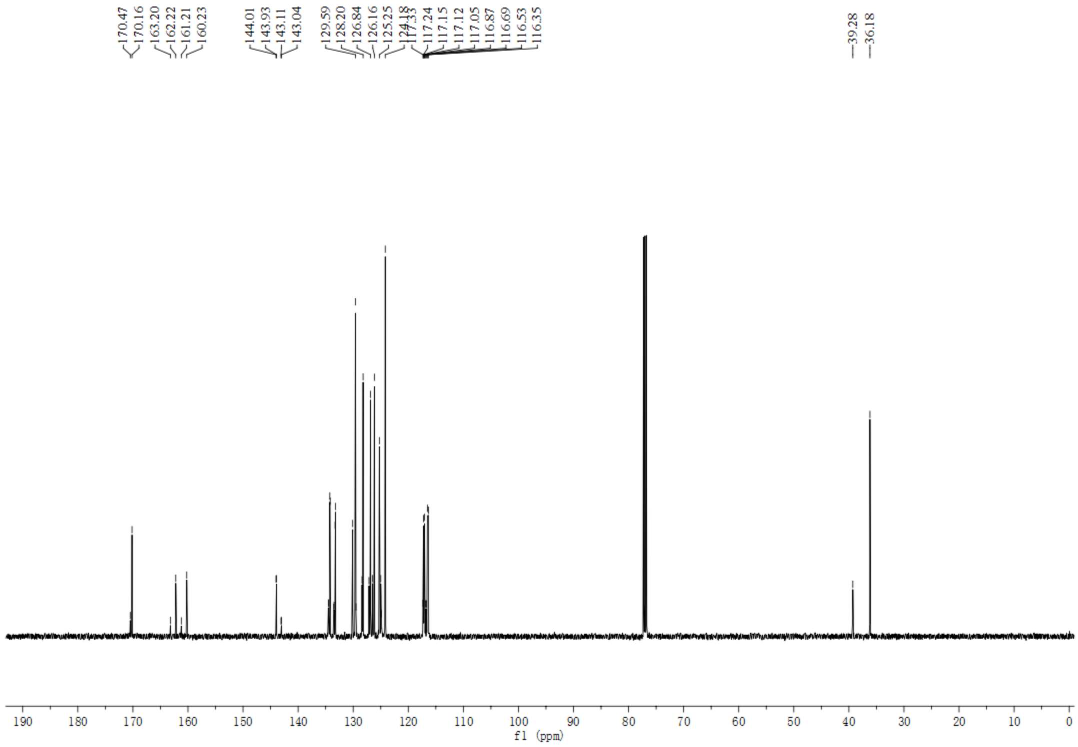

Supplementary Figure 34. ^{13}C NMR Spectra of compound **1p**.

N-(2-bromo-5-(trifluoromethyl)phenyl)-N-methyl-1-naphthamide (**1q**)

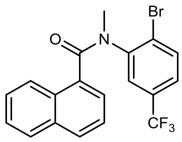

Purified by chromatography on silica gel, eluting with ethyl acetate/petroleum ether 1:10 (*v*/*v*); white solid, Mp = 126-128 ^{o}C, 66% yield; ^{1}H NMR (500 MHz, $CDCl_3$): δ 8.18-8.13 (m, 0.96H), 7.98-7.89 (m, 0.90H), 7.78-7.74 (m, 1.00H), 7.70-7.46 (m, 4.37H), 7.37 (d, J = 7.0 Hz, 0.72H), 7.25 (s, 0.62H), 7.22-7.18 (m, 0.74H), 7.15-7.12 (m, 0.69H), 3.56 (s, 2.13H), 3.14 (s, 0.87H). ^{13}C NMR (125 MHz, $CDCl_3$): δ 170.6, 170.4, 143.3, 142.7, 134.5, 134.1, 133.5, 133.3, 133.1, 130.4 (q, J = 33.8 Hz), 129.9, 129.7, 129.6, 129.5, 128.5, 128.2, 127.2, 126.98, 126.93, 126.7, 126.5, 126.2, 126.1, 125.5 (q, J = 3.8 Hz), 125.08, 125.05, 124.9, 124.3, 124.1, 124.0, 122.6 (q, J = 271.3 Hz), 39.4, 36.1. HRMS *m*/*z* (ESI+): Calculated for $C_{19}H_{14}{}^{79}BrF_3NO$ ($[M+H]^+$): 408.0205, found 408.0219.

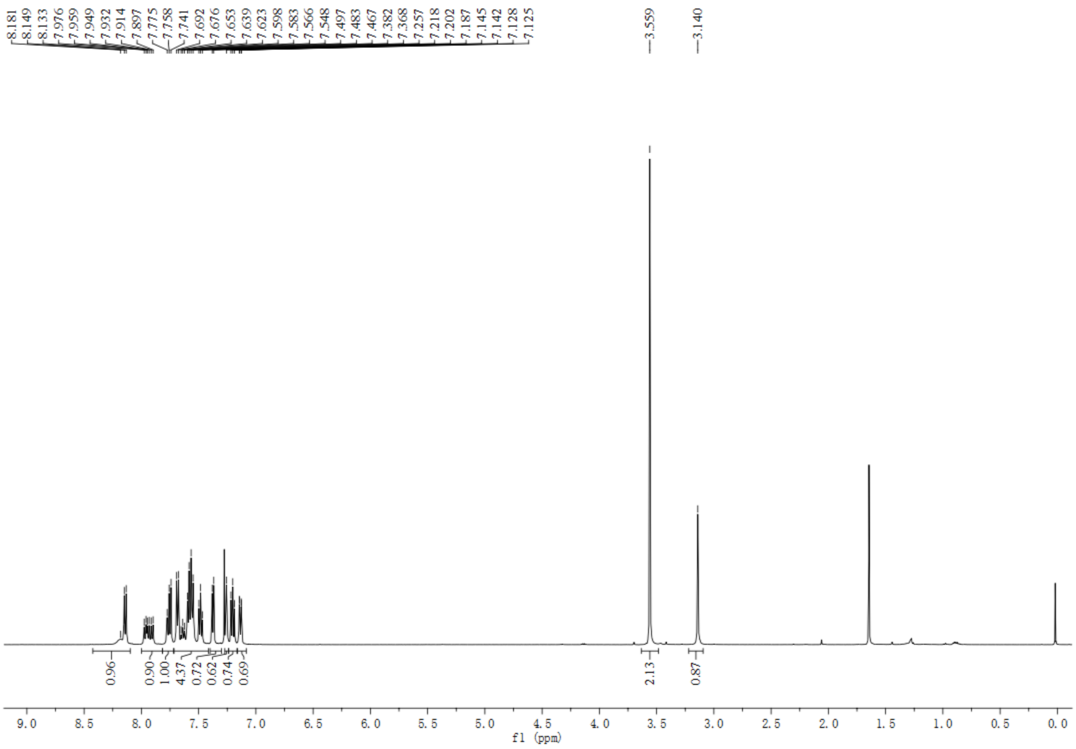

Supplementary Figure 35. ^{1}H NMR Spectra of compound **1q**.

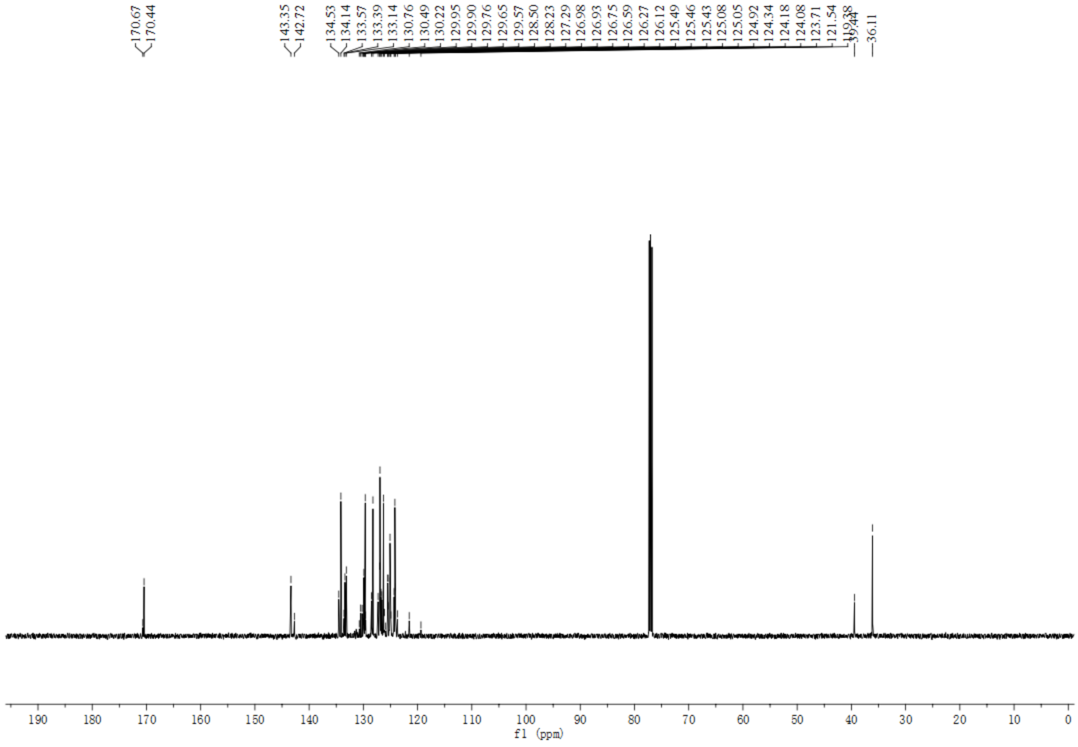

Supplementary Figure 36. ^{13}C NMR Spectra of compound **1q**.

Methyl 4-bromo-3-(*N*-methyl-1-naphthamido)benzoate (**1r**)

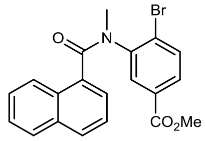

Purified by chromatography on silica gel, eluting with ethyl acetate/petroleum ether 1:10 (*v*/*v*); yellow solid, Mp = 61-63 ^{o}C, 50% yield; ^{1}H NMR (500 MHz, $CDCl_3$): δ 8.21-8.17 (m, 1.22H), 7.96-7.91(m, 0.86H), 7.85 (d, *J* = 8.5 Hz, 0.31H), 7.74-7.45 (m, 6.08H), 7.38 (d, *J* = 6.5 Hz, 0.70H), 7.20-7.17 (m, 0.70H), 3.97 (s, 0.88H), 3.71 (s, 2.08H), 3.54 (s, 2.10H), 3.12 (s, 0.90H). ^{13}C NMR (125 MHz, $CDCl_3$): δ 170.6, 170.4, 165.6, 164.9, 143.0, 142.2, 133.9, 133.6, 133.5, 133.18, 131.14, 130.8, 130.19, 130.13, 130.0, 129.66, 129.60, 129.5, 128.4, 128.1, 127.2, 126.7, 126.5, 126.1, 125.4, 125.0, 124.9, 124.25, 124.20, 124.1, 52.4, 52.1, 39.4, 36.2. HRMS *m*/*z* (ESI+): Calculated for $C_{20}H_{17}{}^{79}BrNO_3$ ($[M+H]^+$): 398.0386, found 398.0395.

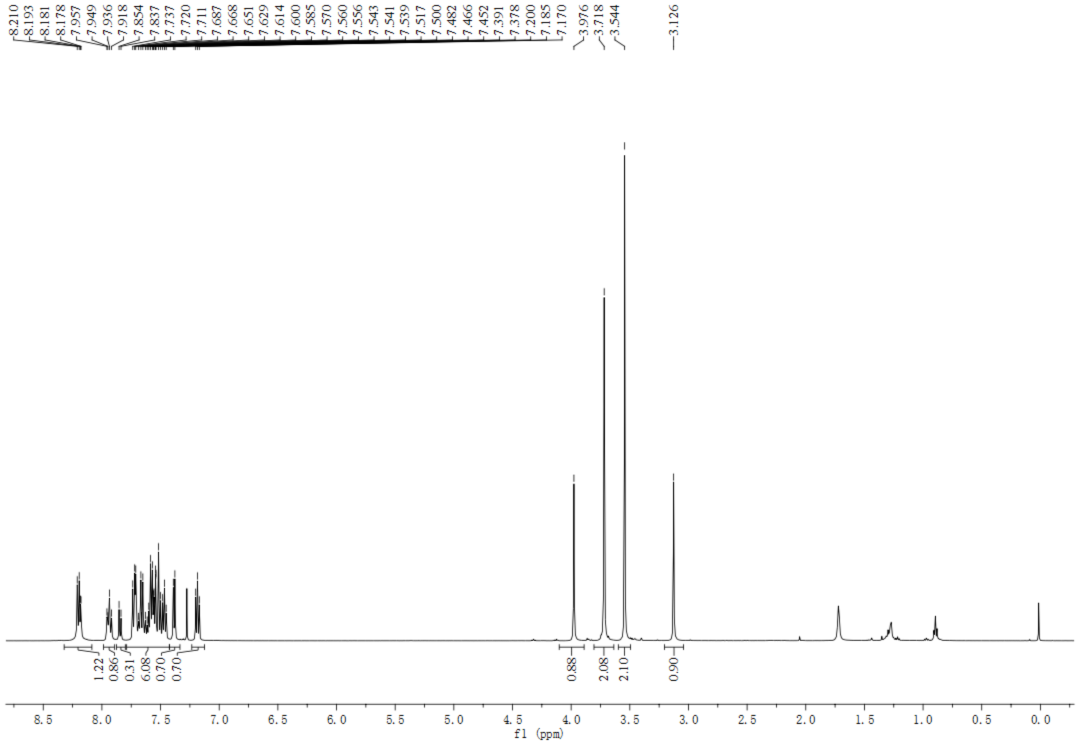

Supplementary Figure 37. ^{1}H NMR Spectra of compound **1r**.

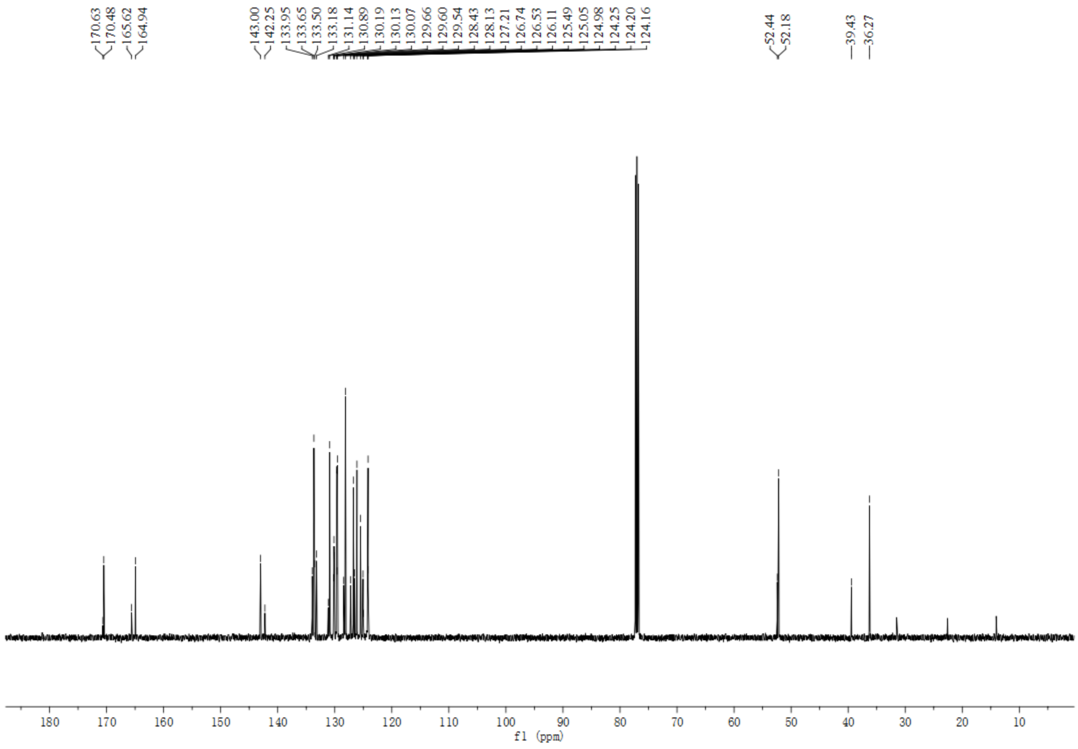

Supplementary Figure 38. ^{13}C NMR Spectra of compound **1r**.

N-(2-bromo-5-(diethylcarbamoyl)phenyl)-*N*-methyl-1-naphthamide (**1s**)

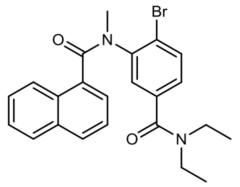

Purified by chromatography on silica gel, eluting with ethyl acetate/petroleum ether 1:10 (*v*/*v*); white solid, Mp = 154-156 °C, 52% yield; ^{1}H NMR (600 MHz, $CDCl_3$): δ 8.17 (d, *J* = 8.4 Hz, 0.90H), 7.96-7.40 (m, 5.98H), 7.33-7.28 (m, 0.45H), 7.23-7.20 (m, 0.81H), 6.95-6.87 (m, 1.59H), 3.53-3.05 (m, 5.00H), 1.94-1.70 (m, 1.92H), 1.29-0.97 (m, 3.61H), 0.33 (s, 2.47H). ^{13}C NMR (150 MHz, $CDCl_3$): δ 170.2, 168.4, 142.8, 137.4, 134.06, 134.00, 133.8, 133.5, 133.4, 130.2, 129.6, 129.1, 128.5, 128.1, 127.4, 127.2, 127.1, 127.07, 127.03, 126.5, 126.3, 125.4, 124.6, 124.2, 123.8, 123.0, 41.9, 39.4, 38.9, 36.0, 13.3, 12.6. HRMS *m*/*z* (ESI+): Calculated for $C_{23}H_{24}{}^{79}BrN_2O_2$ ($[M+H]^+$): 439.1016, found 439.1016.

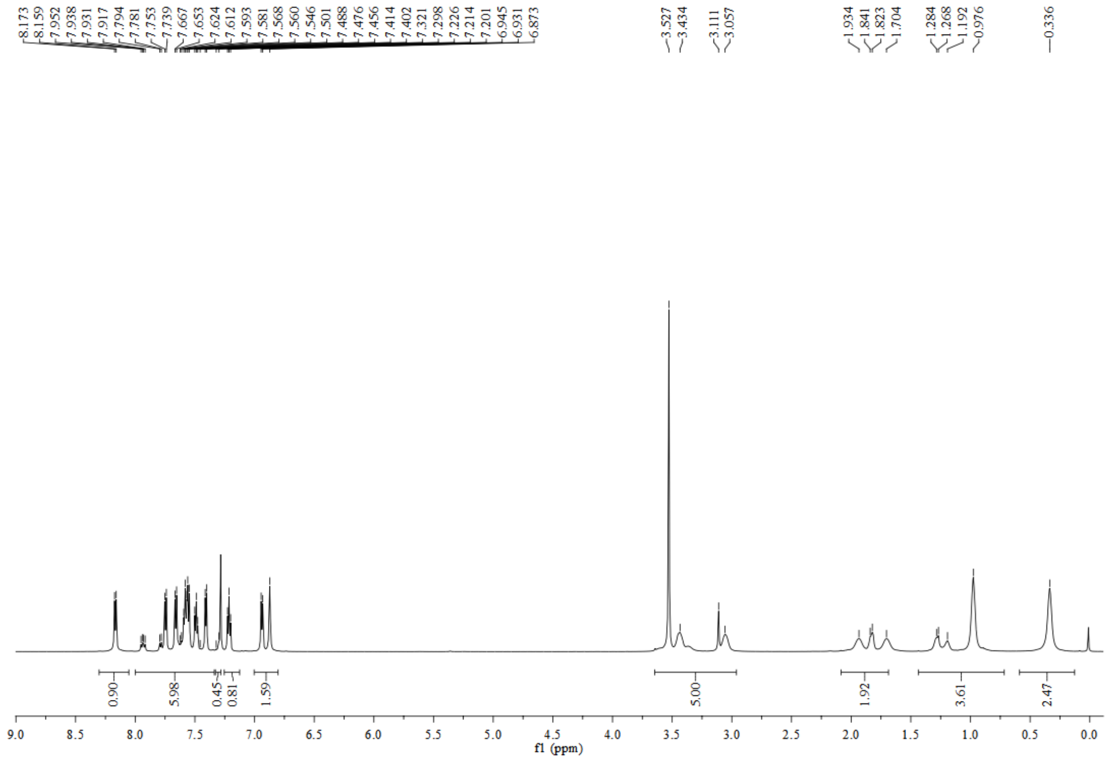

Supplementary Figure 39. ^{1}H NMR Spectra of compound **1s.**

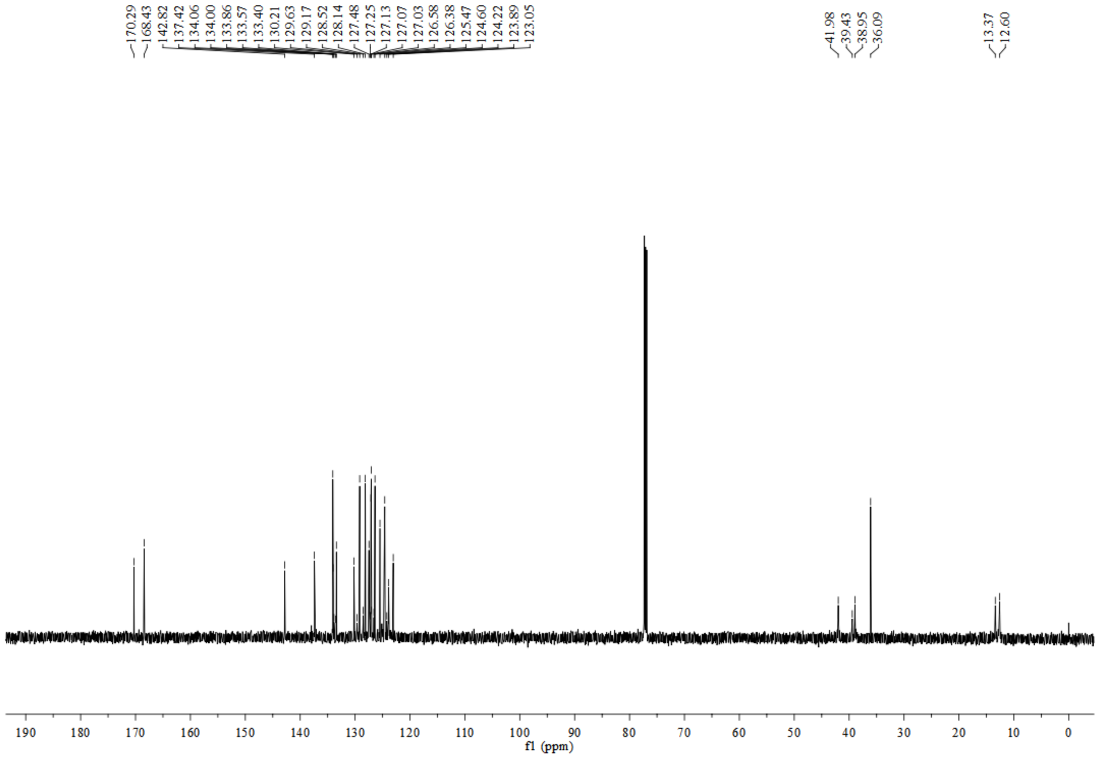

Supplementary Figure 40. ^{13}C NMR Spectra of compound **1s**.

N-(2-bromo-4,5-difluorophenyl)-*N*-methyl-1-naphthamide (**1t**)

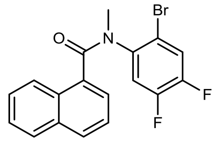

Purified by chromatography on silica gel, eluting with ethyl acetate/petroleum ether 1:10 (*v*/*v*); pale yellow oil, 63% yield; ^{1}H NMR (500 MHz, $CDCl_3$): δ 8.12 (d, *J* = 8.0 Hz, 0.94H), 7.97-7.92 (m, 0.56H), 7.81-7.73 (m, 1.46H), 7.66-7.49 (m, 2.85H), 7.43-7.38 (m, 1.00H), 7.32-7.23 (m, 1.47H), 6.90-6.85 (m, 0.71H), 3.50 (s, 2.20H), 3.09 (s, 0.81H). ^{13}C NMR (125 MHz, $CDCl_3$): δ 170.7, 170.2, 149.1 (dd, *J* = 253.8 Hz, *J* = 13.8 Hz), 148.8 (dd, *J* = 252.5, *J* = 13.8 Hz), 139.3 (dd, *J* = 6.3 Hz, *J* = 3.8 Hz), 133.5, 133.3, 133.27, 133.20, 129.9, 129.7, 129.4, 128.4, 128.3, 127.2, 126.9, 126.5, 126.2, 125.1, 125.0, 124.8, 124.2, 124.0, 122.1 (d, *J* = 20.0 Hz), 121.8 (d, *J* = 20.0 Hz), 118.7 (d, *J* = 18.8), 118.4 (d, *J* =18.8 Hz), 117.0 (dd, *J* = 7.5 Hz, *J* = 3.8 Hz), 39.3, 36.2. HRMS *m*/*z* (ESI+): Calculated for $C_{18}H_{13}{}^{79}BrF_2NO$ ($[M+H]^+$): 376.0143, found 376.0150.

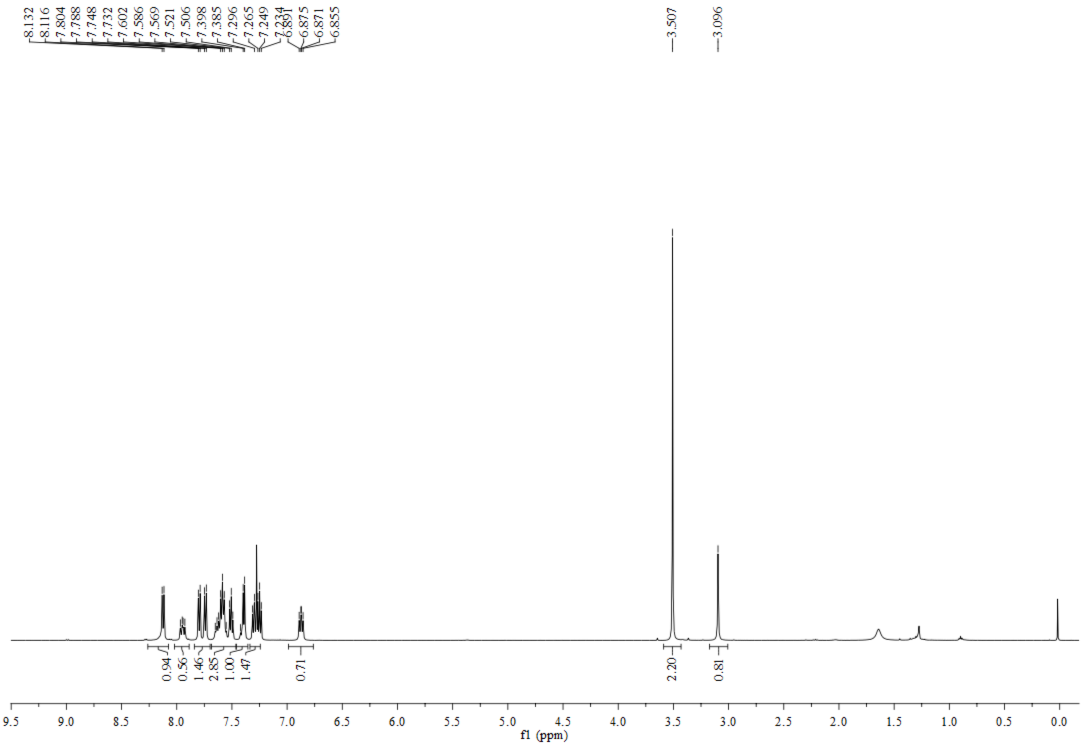

Supplementary Figure 41. ^{1}H NMR Spectra of compound **1t**.

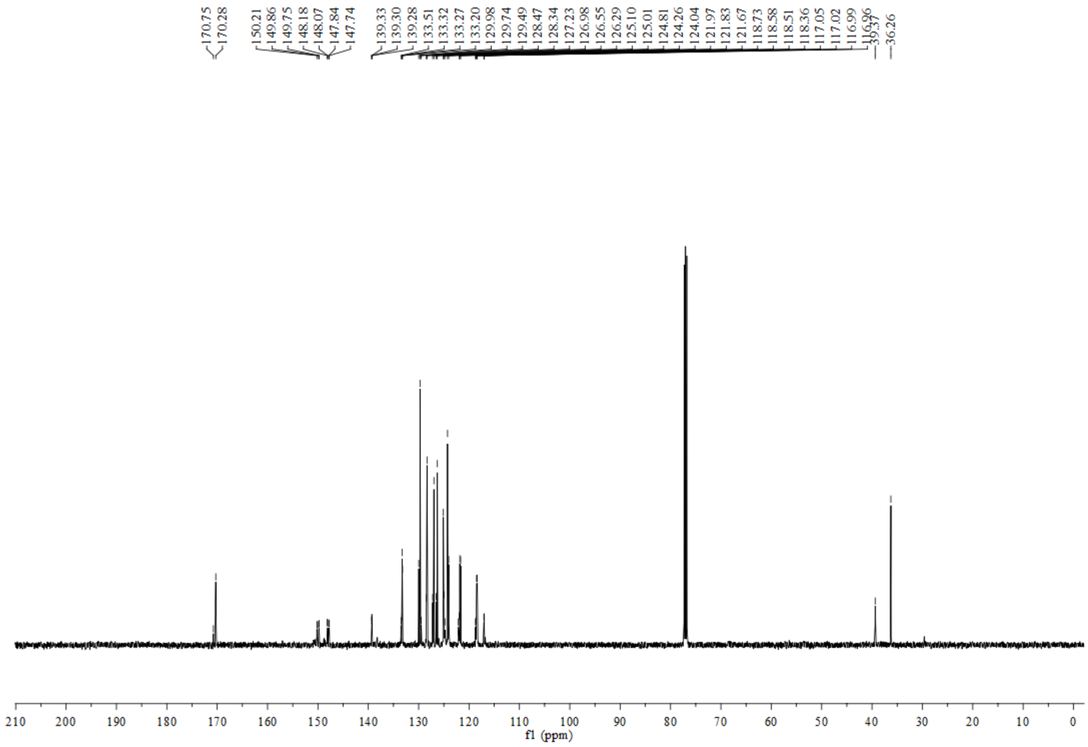

Supplementary Figure 42. ^{13}C NMR Spectra of compound **1t**.

N-benzyl-*N*-(2-bromophenyl)-1-naphthamide (**1u**)

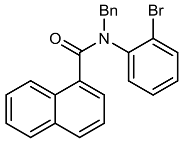

Purified by chromatography on silica gel, eluting with ethyl acetate/petroleum ether 1:10 (*v*/*v*); white solid, Mp = 193-195 ^{o}C, 52% yield; ^{1}H NMR (500 MHz, $CDCl_3$): δ 8.11 (d, *J* = 8.0 Hz, 0.88H), 7.99-7.94 (m, 0.22H), 7.74 (d, *J* = 8.0 Hz, 1.03H), 7.65 (d, *J* = 8.0 Hz, 0.98H), 7.62-7.40 (m, 5.36H), 7.37-7.09 (m, 4.42H), 6.88-6.84 (m, 1.09H), 6.68-6.64 (m, 0.89H), 6.44 (d, *J* = 7.5 Hz, 0.85H), 6.09 (d, *J* = 14.0 Hz, 0.88H), 4.79 (s, 0.09H), 4.54 (d, *J* = 14.5 Hz, 0.10H), 4.37 (d, *J* = 14.5 Hz, 0.89H). ^{13}C NMR (125 MHz, $CDCl_3$): δ 170.1, 140.3, 139.2, 136.8, 133.7, 133.6, 133.3, 133.1, 131.6, 130.2, 129.57, 129.52, 129.3, 129.2, 129.0, 128.4, 128.2, 128.1, 127.9, 127.8, 127.6, 127.3, 127.0, 126.7, 126.5, 125.9, 125.2, 124.9, 124.2, 123.7, 122.9, 51.2. HRMS *m*/*z* (ESI+): Calculated for $C_{24}H_{19}{}^{79}BrNO$ ($[M+H]^+$): 416.0645, found 416.0651.

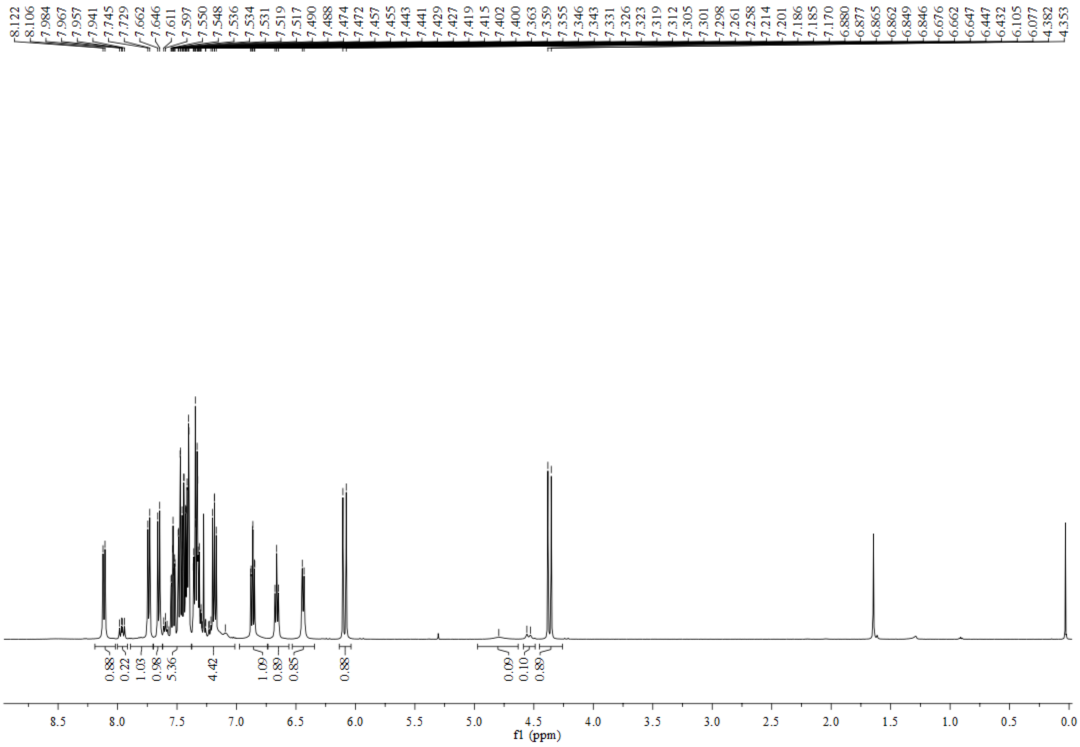

Supplementary Figure 43. ^{1}H NMR Spectra of compound **1u**.

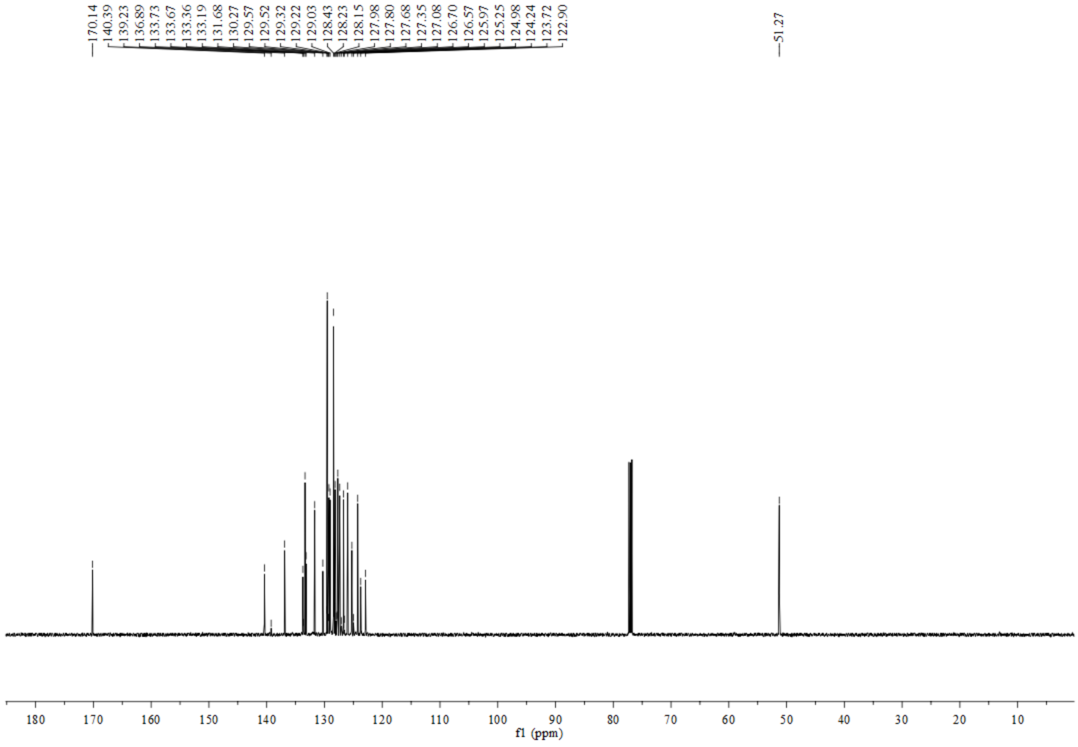

Supplementary Figure 44. ^{13}C NMR Spectra of compound **1u**.

N-(3-bromopyridin-2-yl)-*N*-methyl-1-naphthamide (**1v**)

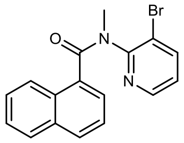

Purified by chromatography on silica gel, eluting with ethyl acetate/petroleum ether 1:5 (*v*/*v*); yellow oil, 31% yield; ^{1}H NMR (600 MHz, $CDCl_3$): δ 8.56-6.82 (m, 10.09H), 3.55 (s, 2.25H), 3.18 (s, 0.75H). ^{13}C NMR (150 MHz, $CDCl_3$): δ 170.5, 154.6, 147.8, 142.3, 133.6, 133.4, 130.6, 129.8, 127.9, 126.6, 126.1, 125.4, 123.9, 119.4, 35.0. HRMS *m*/*z* (ESI+): Calculated for $C_{17}H_{13}{}^{79}BrN_2NaO$ ($[M+Na]^+$): 363.0103, found 363.0099.

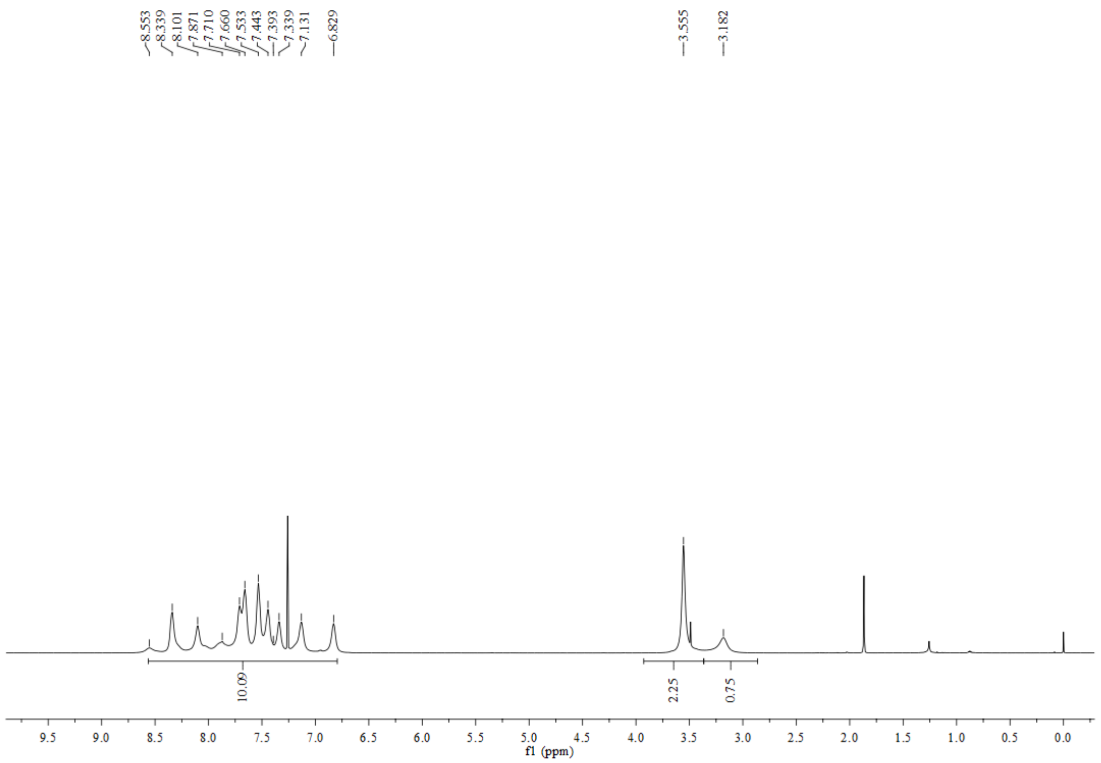

Supplementary Figure 45. ^{1}H NMR Spectra of compound **1v**.

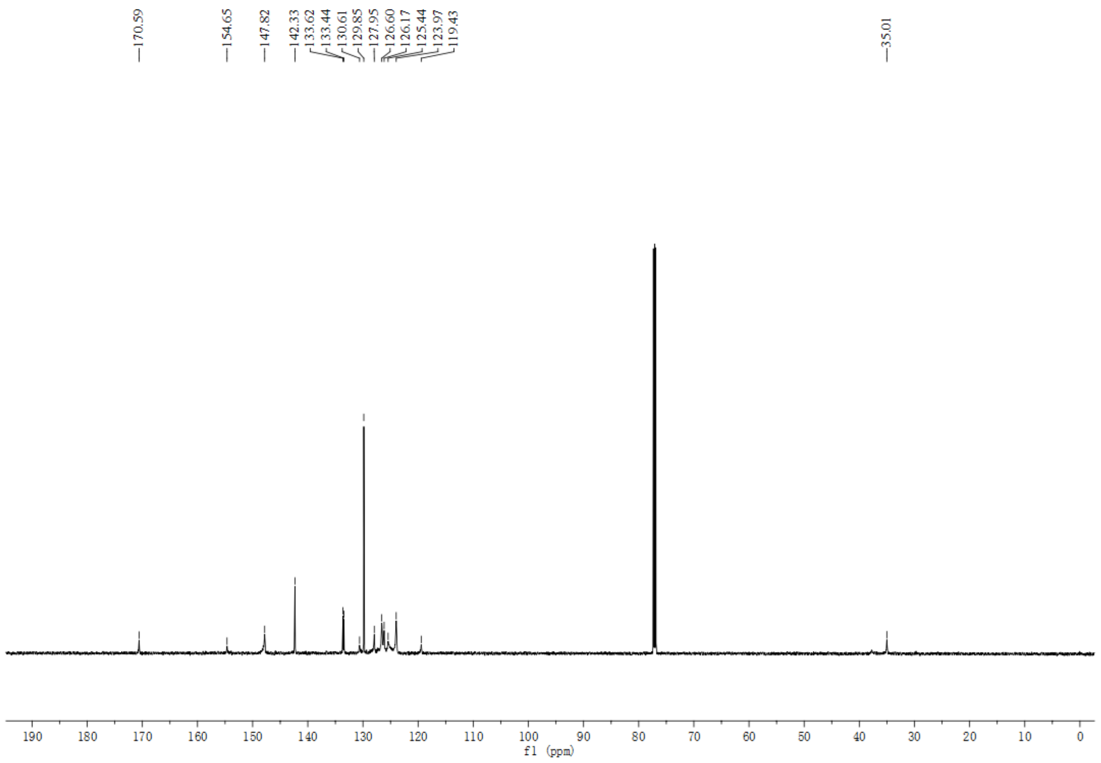

Supplementary Figure 46. ^{13}C NMR Spectra of compound **1v**.

N-(2-bromophenyl)-6-methoxy-*N*-methyl-1-naphthamide (**1w**)

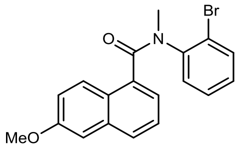

Purified by chromatography on silica gel, eluting with ethyl acetate/petroleum ether 1:5 (*v*/*v*); white solid, Mp = 110-112 °C, 70% yield; ^{1}H NMR (500 MHz, $CDCl_3$): δ 8.11 (d, *J* = 9.0 Hz, 1.00H), 7.84-7.81 (m, 0.18H), 7.77-7.74 (m, 0.18H), 7.57-7.46 (m, 2.41H), 7.30-7.26 (m, 0.33H), 7.25-7.20 (m, 1.85H), 7.16-7.12 (m, 0.81H), 7.04 (d, *J* = 2.5 Hz, 0.81H), 6.98-6.88 (m, 2.48H), 3.95 (s, 0.52H), 3.90 (s, 2.47H), 3.52 (s, 2.46H), 3.10 (s, 0.54H). ^{13}C NMR (125 MHz, $CDCl_3$): δ 170.69, 170.62, 157.9, 157.5, 142.9, 141.9, 134.9, 134.6, 133.9, 133.7, 133.6, 133.5, 129.8, 129.7, 129.4, 128.9, 128.8, 128.2, 128.1, 128.0, 127.1, 125.74, 125.71, 125.0, 124.8, 122.5, 122.0, 121.8, 119.8, 119.4, 106.3, 106.0, 55.3, 55.2, 39.5, 36.3. HRMS *m*/*z* (ESI+): Calculated for $C_{19}H_{16}{}^{79}BrNNaO_2$ ($[M+Na]^+$): 392.0257, found 392.0260.

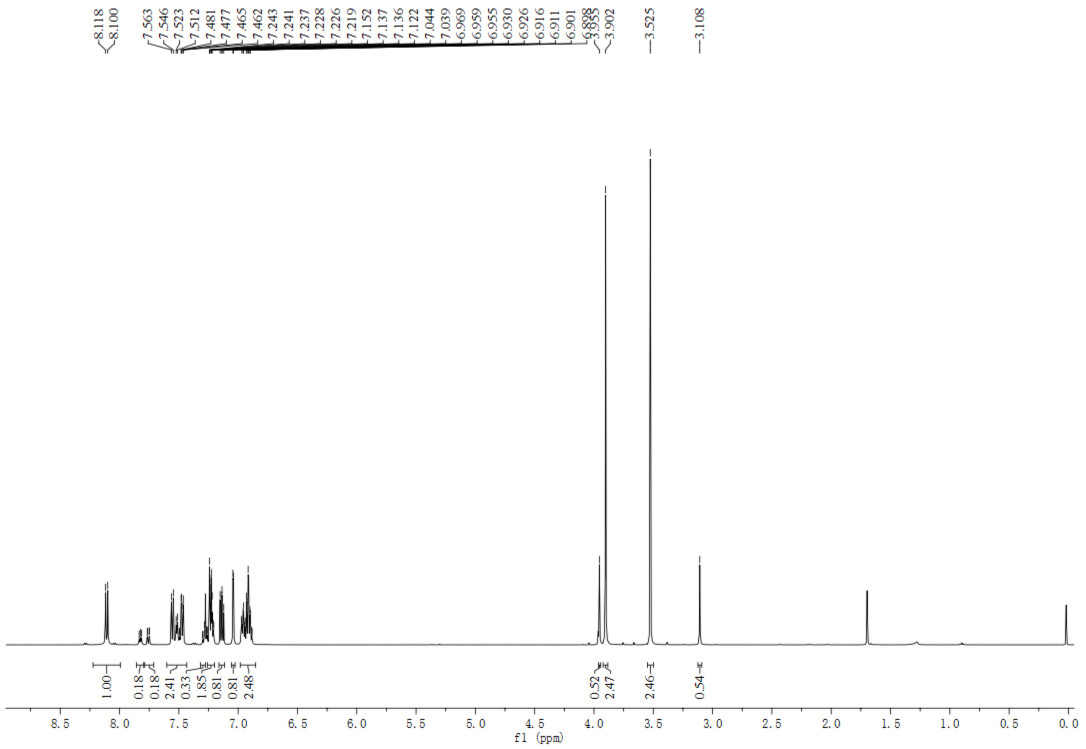

Supplementary Figure 47. ^{1}H NMR Spectra of compound **1w**.

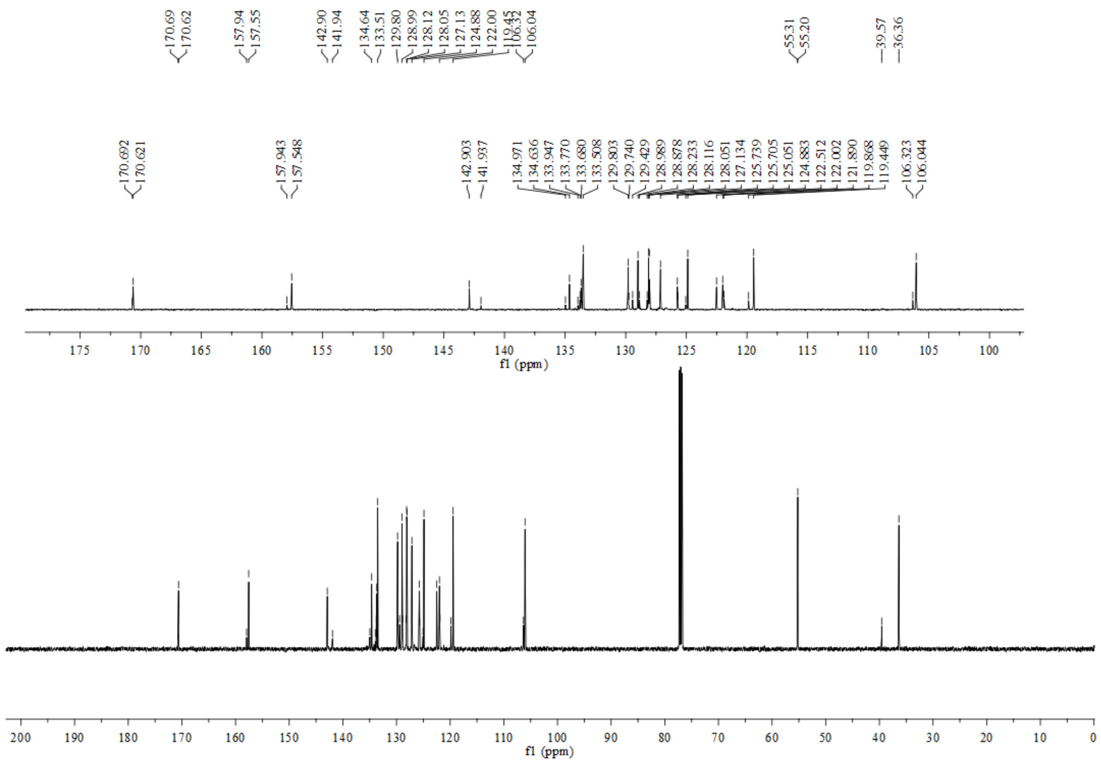

Supplementary Figure 48. ^{13}C NMR Spectra of compound **1w**.

5-Bromo-*N*-(2-bromophenyl)-*N*-methyl-1-naphthamide (**1x**)

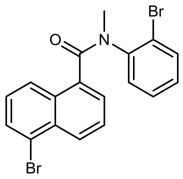

Purified by chromatography on silic a gel, eluting with ethyl acetate/petroleum ether 1:10 (*v*/*v*); white solid, Mp = 131-133 °C, 65% yield; ^{1}H NMR (500 MHz, $CDCl_3$): δ 8.37 (d, *J* = 7.5 Hz, 0.20H), 8.21-8.36 (m, 1.73H), 7.87-7.67 (m, 1.53H), 7.51-7.38 (m, 2.94H), 7.32-7.27 (m, 1.07H), 6.96-6.89 (m, 2.53H), 3.53 (s, 2.50H), 3.07 (s, 0.50H). ^{13}C NMR (125 MHz, $CDCl_3$): δ 169.7, 142.4, 141.5, 134.5, 134.2, 133.6, 133.5, 131.9, 131.6, 131.3, 130.7, 130.5, 130.2, 129.6, 129.5, 129.4, 129.1, 128.8, 128.5, 128.3, 128.1, 127.3, 126.8, 126.5, 125.7, 125.5, 124.96, 124.93, 123.2, 122.9, 122.3, 39.4, 36.3. HRMS *m*/*z* (ESI+): Calculated for $C_{18}H_{13}{}^{79}Br_2NNaO$ ($[M+Na]^+$): 439.9256, found 439.9260.

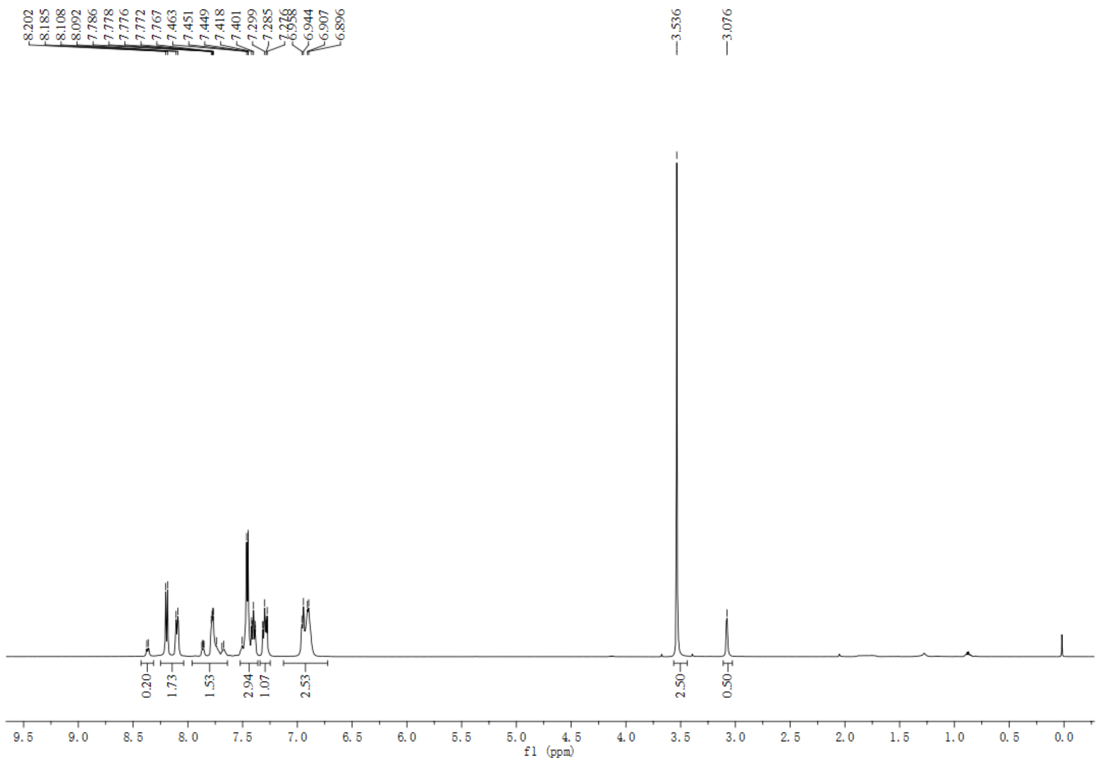

Supplementary Figure 49. ^{1}H NMR Spectra of compound **1x**.

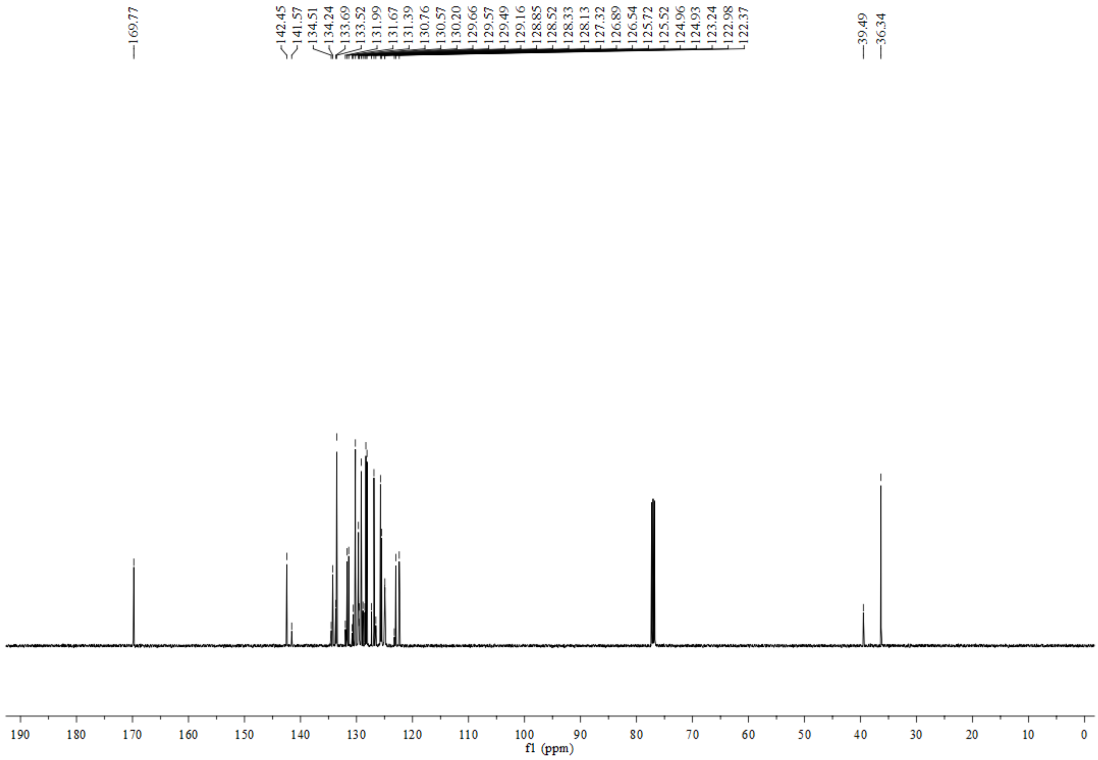

Supplementary Figure 50. ^{13}C NMR Spectra of compound **1x**.

N-(2-bromo-5-chlorophenyl)-*N*-methyl-1-naphthamide (**1y**)

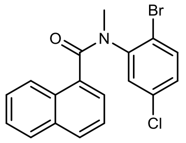

Purified by chromatography on silica gel, eluting with ethyl acetate/petroleum ether 1:10 (*v*/*v*); white solid, Mp = 114-116 °C, 71% yield; ^{1}H NMR (500 MHz, $CDCl_3$): δ 8.16 (d, *J* = 8.5 Hz, 0.94H), 7.97-7.92 (m, 0.60H), 7.79-7.48 (m, 4.63H), 7.41-7.35 (m, 1.38H), 7.29 (d, *J* = 2.5 Hz, 0.22H), 7.25-7.21 (m, 0.70H), 7.03 (d, *J* = 2.0 Hz, 0.65H), 6.92-6.89 (m, 0.67H), 3.52 (s, 2.13H), 3.10 (s, 0.86H). ^{13}C NMR (125 MHz, $CDCl_3$): δ 170.5, 170.2, 143.7, 142.9, 134.4, 134.1, 133.56, 133.51, 133.3, 133.2, 130.07, 130.00, 129.66, 129.63, 129.5, 129.1, 128.7, 128.4, 128.2, 127.2, 126.8, 126.5, 126.1, 125.3, 125.0, 124.9, 124.2, 124.1, 120.7, 39.3, 36.2. HRMS *m*/*z* (ESI+): Calculated for $C_{18}H_{14}{}^{79}Br^{35}ClNO$ ($[M+H]^+$): 373.9942, found 373.9944.

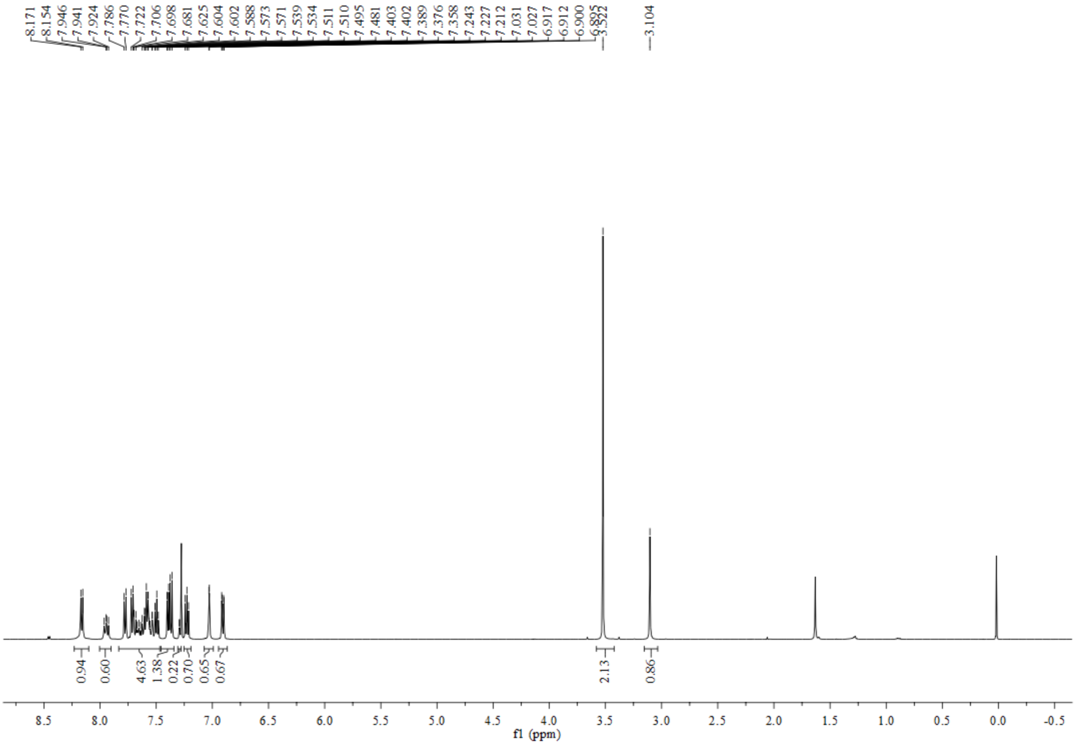

Supplementary Figure 51. ^{1}H NMR Spectra of compound **1y**.

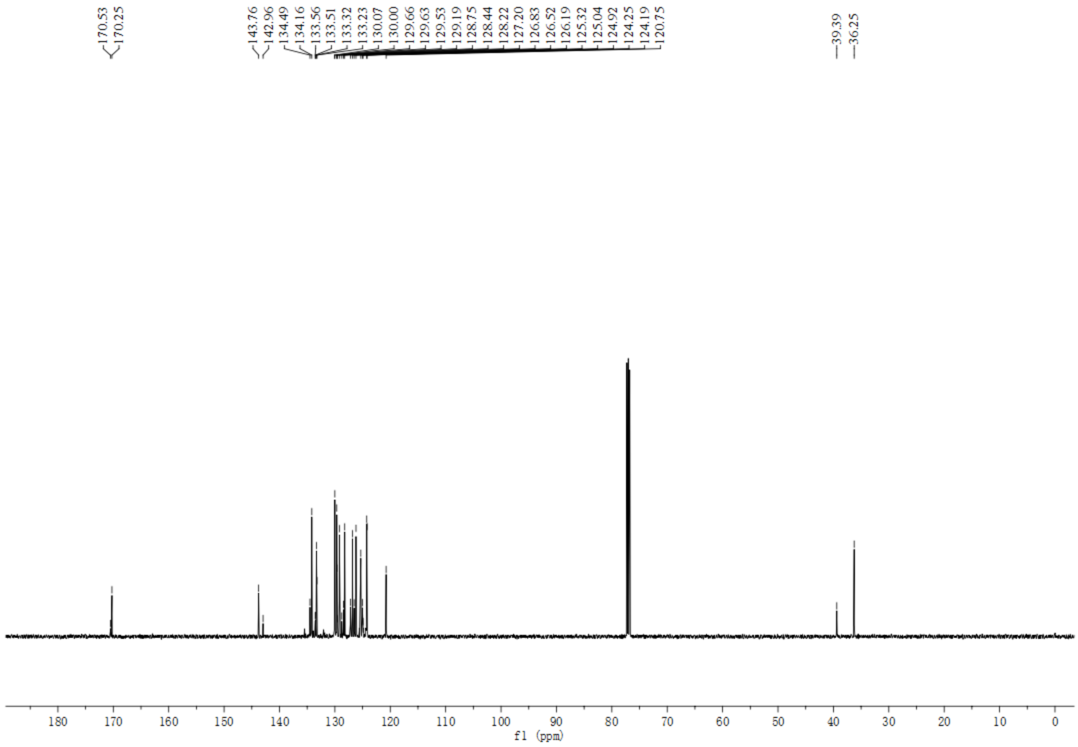

Supplementary Figure 52. ^{13}C NMR Spectra of compound **1y**.

N-(2-bromophenyl)-*N*-methylanthracene-1-carboxamide (**1z**)

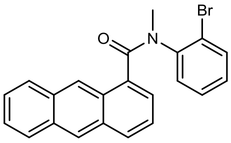

Purified by chromatography on silica gel, eluting with ethyl acetate/petroleum ether 1:10 (*v*/*v*); white solid, Mp = 131-133 ^{o}C, 69% yield; ^{1} H NMR (600 MHz, $CDCl_3$): δ 8.84 (s, 0.91H), 8.48 (s, 0.16H), 8.31 (s, 0.82H), 8.14 (d, *J* = 7.8 Hz, 0.82H), 8.08 (d, *J* = 7.8 Hz, 0.34H), 8.04-8.01 (m, 0.17H), 7.95 (d, *J* = 8.4 Hz, 0.83H), 7.82-7.78 (m, 0.98H), 7.68 (d, *J* = 6.0 Hz, 0.16H), 7.61 (d, J = 7.2 Hz, 0.16H), 7.55-7.42 (m, 4.05H), 7.32-7.29 (m, 0.17H), 7.19-7.15 (m, 0.84H), 6.99 (d, *J* = 7.8 Hz, 0.81H), 6.87-6.83 (m, 0.84H), 6.78-6.75 (m, 0.82H), 3.63 (s, 2.49H), 3.13 (s, 0.51H). ^{13}C NMR (150 MHz, $CDCl_3$): δ 170.8, 170.7, 142.9, 141.9, 134.2, 133.9, 133.8, 133.6, 132.3, 132.1, 131.9, 131.6, 131.5, 131.3, 129.95, 129.92, 129.8, 129.7, 129.5, 129.1, 129.0, 128.6, 128.5, 128.4, 128.1, 128.0, 127.7, 127.0, 126.8, 126.05, 126.01, 125.87, 125.84, 124.4, 124.3, 124.1, 123.9, 123.6, 122.4, 39.6, 36.5. HRMS *m*/*z* (ESI+): Calculated for

$C_{22}H_{17}{}^{79}BrNO$ ($[M+H]^+$): 390.0488, found 390.0477.

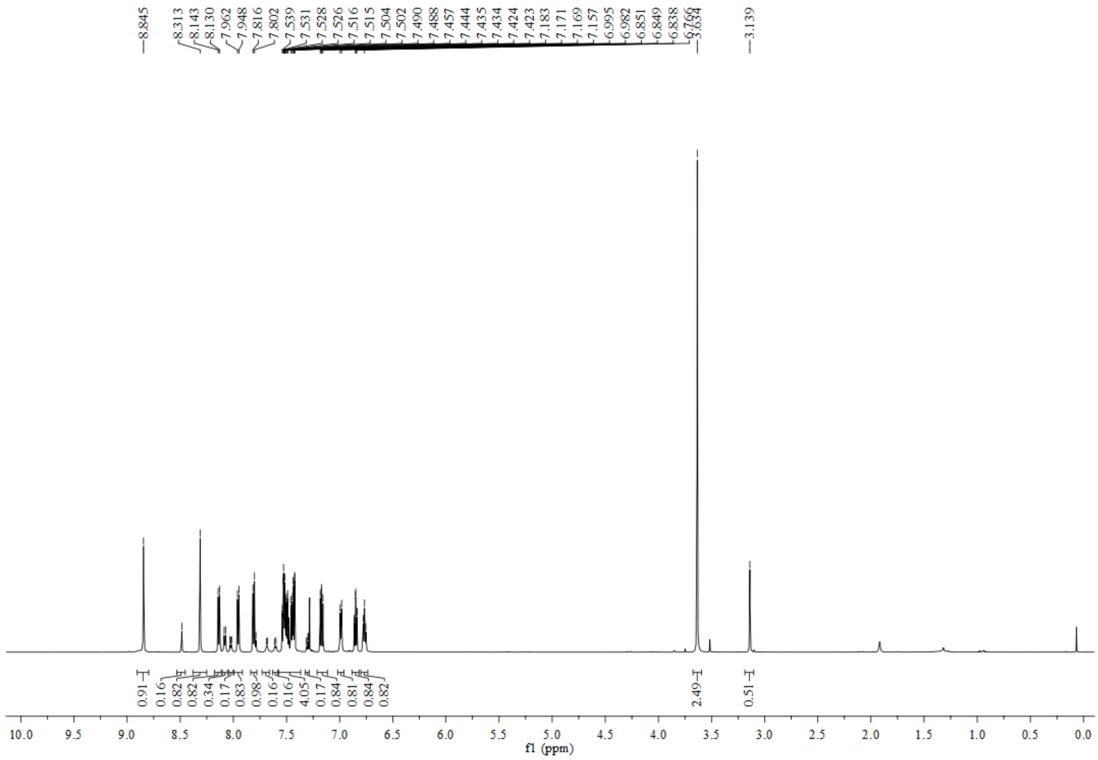

Supplementary Figure 53. ^{1}H NMR Spectra of compound **1z**.

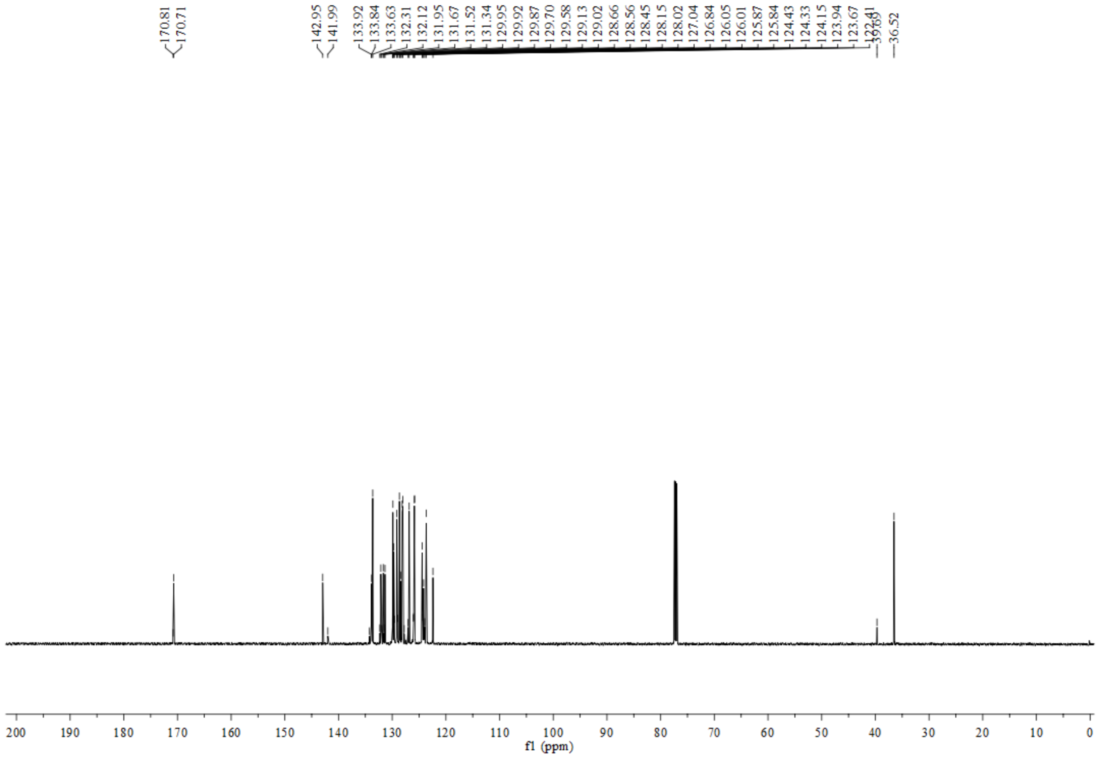

Supplementary Figure 54. ^{13}C NMR Spectra of compound **1z**.

N-(2-bromophenyl)-4-methoxy-*N*-methyl-1-naphthamide (**8**)

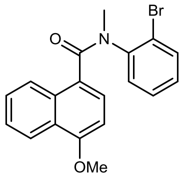

Purified by chromatography on silica gel, eluting with ethyl acetate/petroleum ether 1:5 (*v*/*v*); white solid, Mp = 132-134 °C, 60% yield; ^{1}H NMR (600 MHz, $CDCl_3$): δ 8.22-8.19 (m, 1.75H), 7.60-7.46 (m, 3.49H), 7.31 (d, *J* = 8.4 Hz, 1.16H), 6.99-6.93 (m, 2.70H), 6.51 (d, *J* = 7.8 Hz, 0.84H), 4.07 (s, 0.45H), 3.90 (s, 2.55H), 3.52 (s, 2.55H), 3.14 (s, 0.45H). ^{13}C NMR (150 MHz, $CDCl_3$): δ 170.8, 156.1, 143.5, 134.7, 133.5, 131.7, 129.8, 129.2, 128.8, 128.2, 127.2, 126.1, 125.7, 125.5, 125.4, 125.3, 122.5, 122.4, 122.0, 102.6, 102.1, 55.4, 36.5. HRMS *m*/*z* (ESI+): Calculated for $C_{19}H_{17}{}^{79}BrNO_2$ ($[M+H]^+$): 370.0437, found 370.0433.

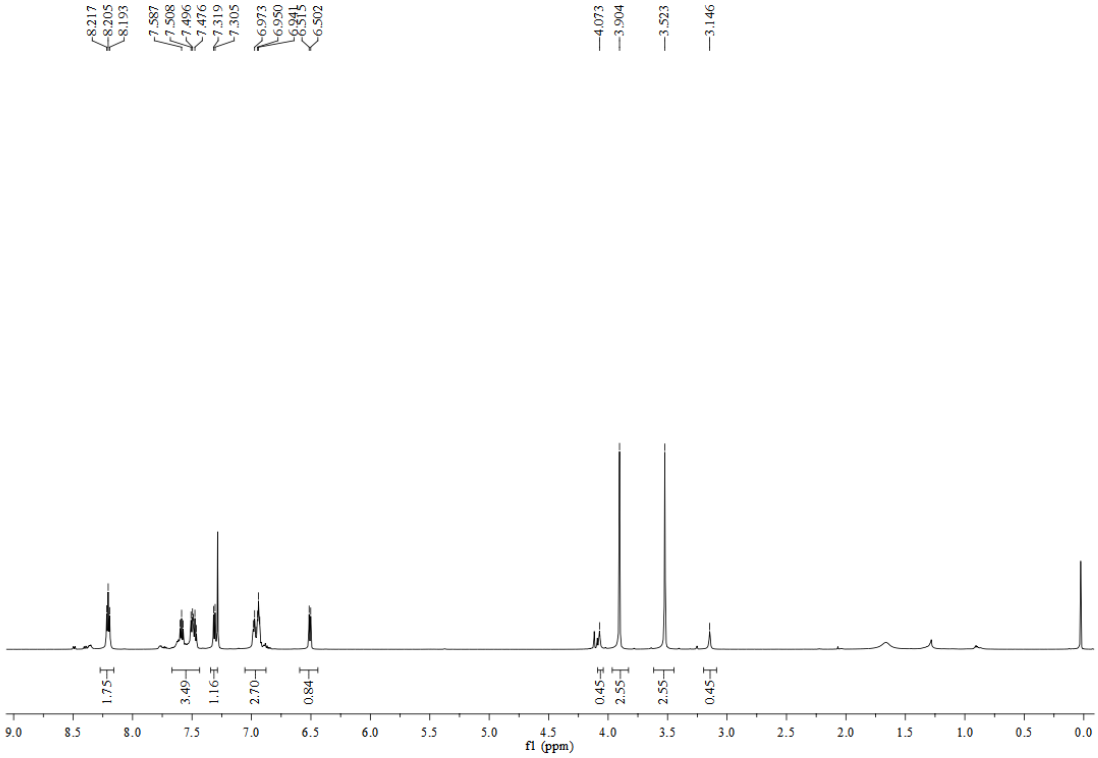

Supplementary Figure 55. ^{1}H NMR Spectra of compound **8**.

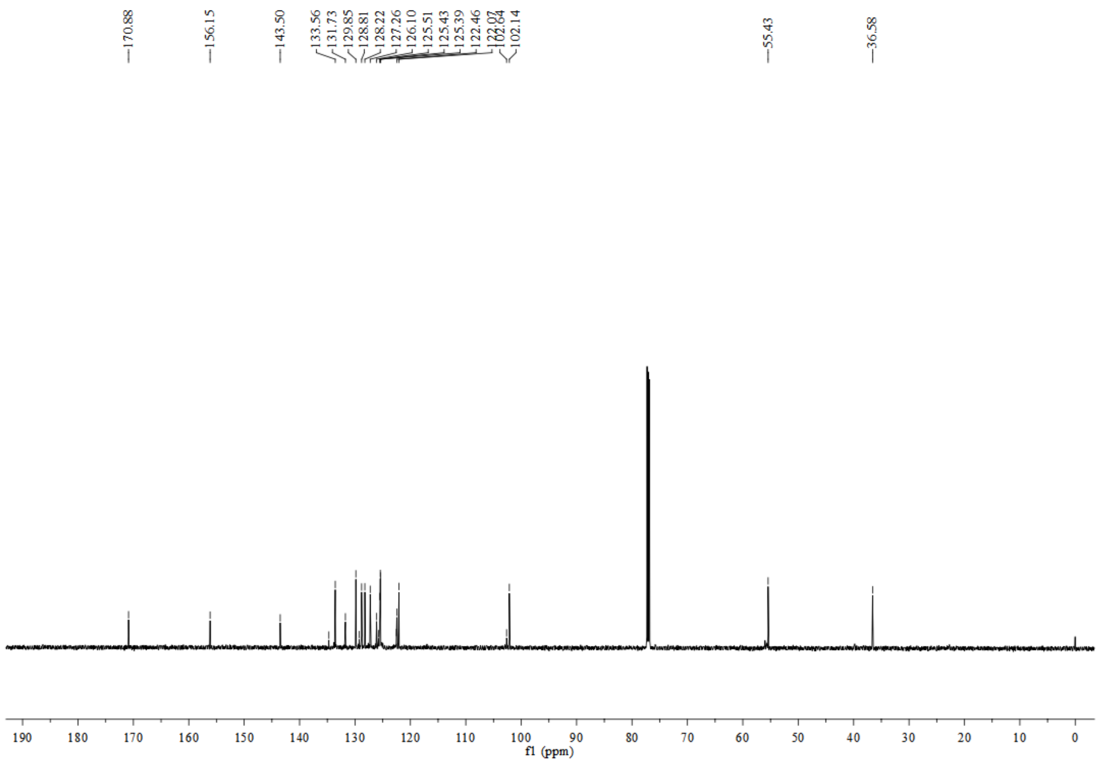

Supplementary Figure 56. ^{13}C NMR Spectra of compound **8**.

The synthesis of substrate 11

Substrate **11** was prepared by the condensation of 1-(bromomethyl)naphthalene with methyl 4-bromo-3-hydroxybenzoate.

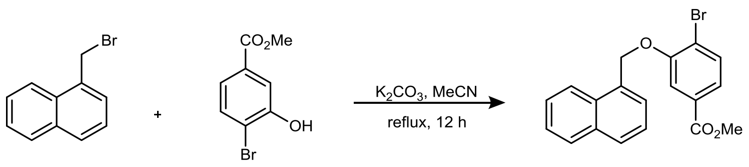

To a stirred solution of 1-(bromomethyl)naphthalene (5.0 mmol, 1.0 equiv.) and 4-bromo-3-hydroxybenzoate (5.5 mmol, 1.1 equiv.) in MeCN (30 mL) were added K_2CO_3 (10.0 mmol, 2.0 equiv.), and the mixture was refluxed for 12 h and then concentrated under vacuum. The residue was diluted with EtOAc, washed with brine, and dried over Na_2SO_4. After filtration, the solvent was concentrated under vacuum and the residue was purified by flash chromatography on silica gel, eluting with ethyl acetate/petroleum ether 1:20 (*v*/*v*) to afford the substrate; white solid, Mp = 126-128 ^{o}C, 41% yield; ^{1}H NMR (500 MHz, $CDCl_3$): δ 8.11 (d, *J* = 8.5 Hz, 1H), 7.93-7.87 (m, 2H), 7.80 (d, *J* = 2.0 Hz, 1H), 7.74 (d, *J* = 7.0 Hz, 1H), 7.66 (d, *J* = 8.5 Hz, 1H), 7.61-7.50 (m, 4H), 5.64 (s, 2H), 3.94 (s, 3H). ^{13}C NMR (125 MHz, $CDCl_3$): δ 166.3, 155.0, 133.6, 133.4, 131.2, 131.1, 130.4, 129.0, 128.6, 126.4, 126.1, 125.9, 125.2, 123.5, 123.1, 118.3, 114.0, 69.5, 52.3. HRMS *m*/*z* (ESI+): Calculated for $C_{19}H_{15}{}^{79}BrNaO_3$ ($[M+Na]^+$): 393.0097, found 393.0100.

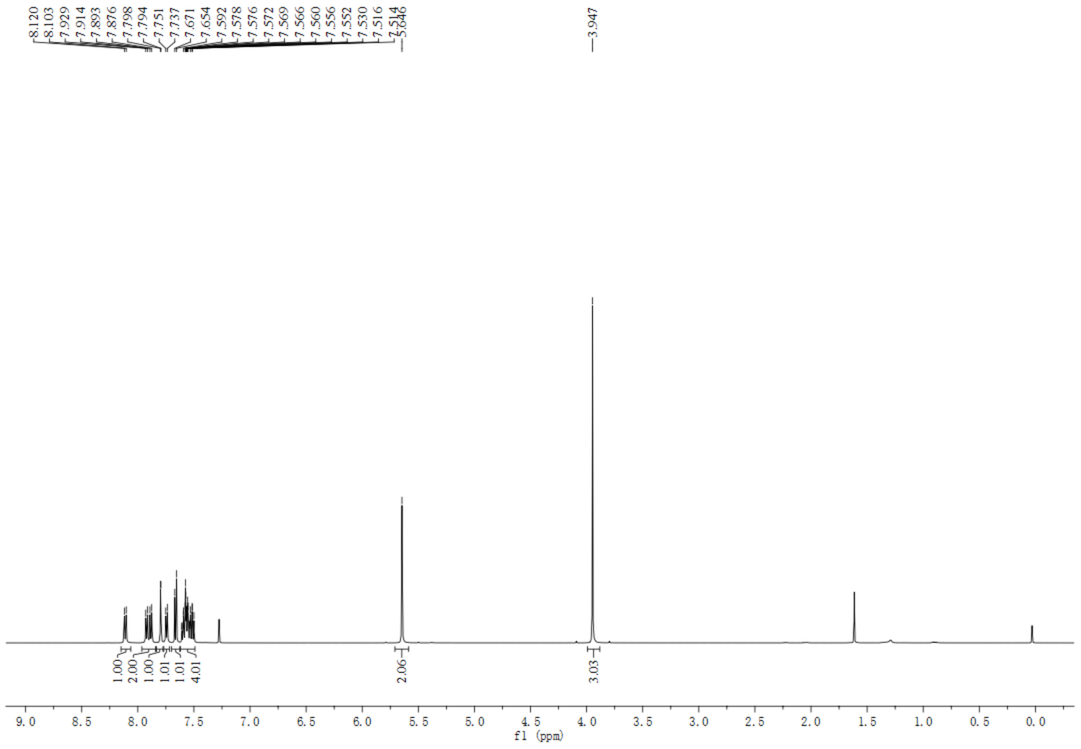

Supplementary Figure 57. ^{1}H NMR Spectra of compound **11**.

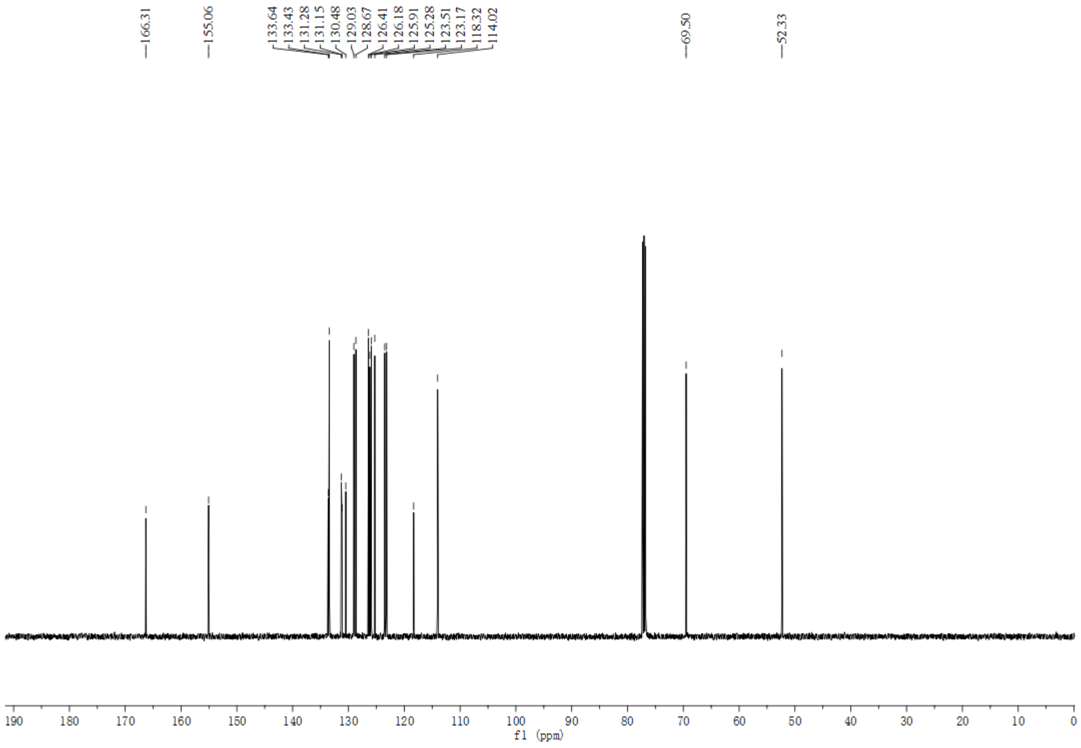

Supplementary Figure 58. ^{13}C NMR Spectra of compound **11**.

Typical procedure for the synthesis of substrates 13

Substrates **13** were prepared according to the literature procedures.[3]

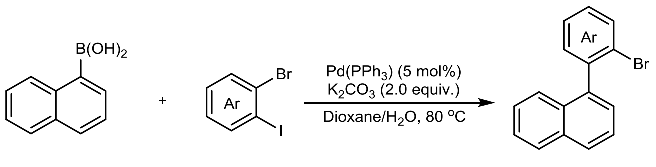

A 25 mL round bottom flask with a stir bar was fitted with a rubber septum and flame dried under high vacuum. The flask was purged with N_2 and charged with $Pd(PPh_3)_4$ (289.0 mg, 0.25 mmol), K_2CO_3 (1.38 g, 10.0 mmol), naphthalen-1-ylboronic acid (1.03 g, 6.0 mmol), *ortho*-dihalogenated aryl derivatives (5.0 mmol), 10.0 mL deoxygenated dioxane and 2.0 mL deoxygenated water. The mixture was stirred at 80 ℃ until the reaction was judged to be completed by TLC analysis. Water was added and the mixture was diluted with ethyl acetate. The layers were separated and the organic layer was washed with water and brine, dried over anhydrous Na_2SO_4 and concentrated under reduced pressure. The residue was purified by flash chromatography on silica gel, eluting with petroleum ether to afford the substrates.

1-(2-Bromo-4-methoxyphenyl)naphthalene (**13c**)

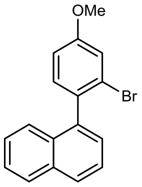

Purified by chromatography on silica gel, eluting with dichloromethane/petroleum ether 1:5 (*v*/*v*); white solid, Mp = 104-106 °C, 29% yield; ^{1}H NMR (600 MHz, $CDCl_3$): δ 7.93 (t, *J* = 7.8 Hz, 2H), 7.58-7.50 (m, 3H), 7.47-7.44 (m, 1H), 7.40-7.38 (m, 1H), 7.33 (d, *J* = 2.4 Hz, 1H), 7.30 (d, *J* = 8.4 Hz, 1H), 7.01 (dd, *J* = 8.4, 2.4 Hz, 1H), 3.91 (s, 3H). ^{13}C NMR (150 MHz, $CDCl_3$): δ 159.5, 138.8, 133.5, 133.4, 132.3, 132.0, 128.2, 128.0, 127.4, 126.07, 126.05, 125.8, 125.2, 124.6, 117.8, 113.4, 55.6. HRMS *m*/*z* (EI+): Calculated for $C_{17}H_{13}O^{79}Br$ (M^+): 312.0150, found 312.0147.

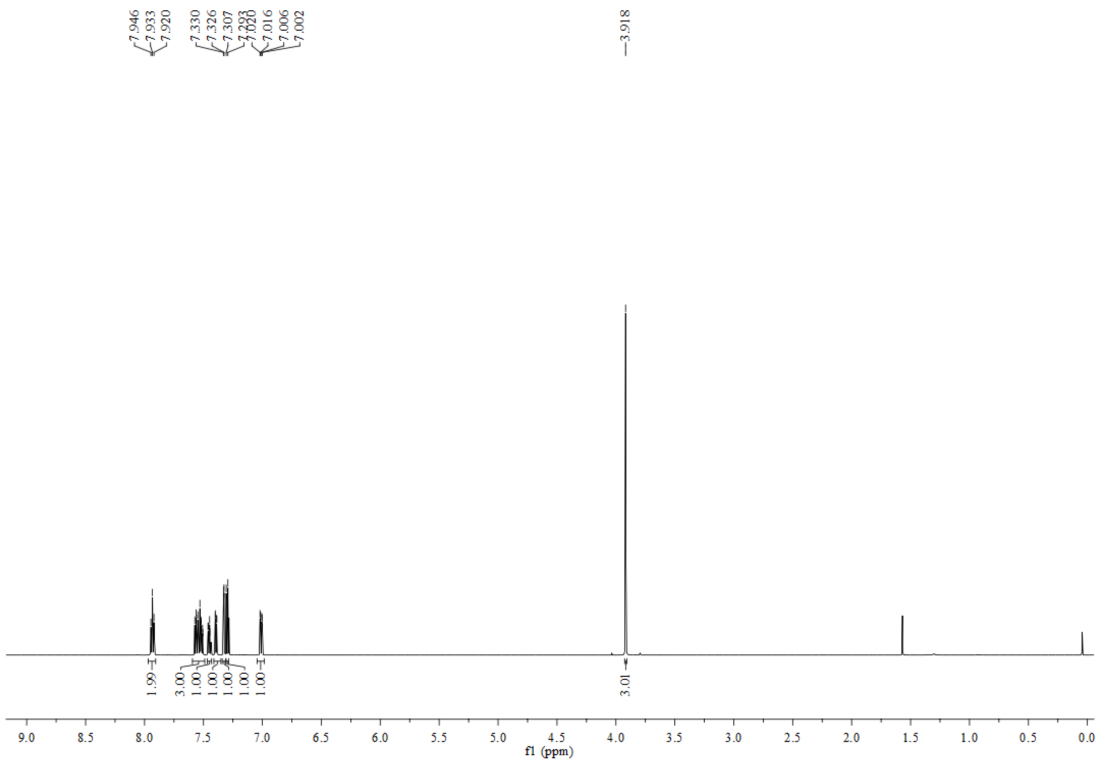

Supplementary Figure 59. ^{1}H NMR Spectra of compound **13c**.

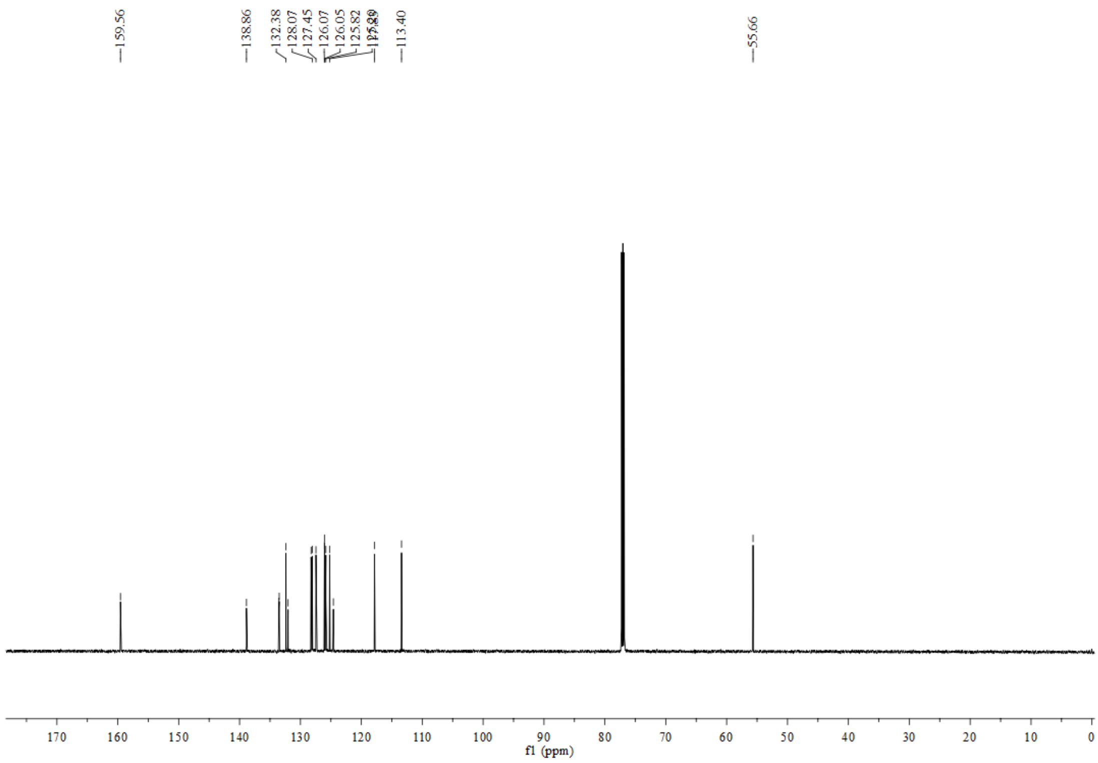

Supplementary Figure 60. ^{13}C NMR Spectra of compound **13c**.

1-(2-Bromo-4-fluorophenyl)naphthalene (**13d**)

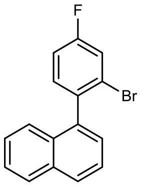

Purified by chromatography on silica gel, eluting with petroleum ether; white solid, Mp = 100-102 °C, 33% yield; ^{1}H NMR (600 MHz, $CDCl_3$): δ 7.95 (d, *J* = 8.4 Hz, 2H), 7.59-7.56 (m, 1H), 7.55-7.51 (m, 2H), 7.48-7.44 (m, 2H), 7.39-7.35 (m, 2H), 7.21-7.16 (m, 1H). ^{13}C NMR (150 MHz, $CDCl_3$): δ 161.9 (d, *J* = 249.0 Hz), 138.11, 137.4 (d, *J* = 4.5 Hz), 133.48, 132.8 (d, *J* = 7.5 Hz), 131.67, 128.44, 128.35, 127.30, 126.30, 125.98, 125.71, 125.18, 124.5 (d, *J* = 9.0 Hz), 119.9 (d, *J* = 24.0 Hz), 114.5 (d, *J* = 21.0 Hz). HRMS *m/z* (EI+): Calculated for $C_{16}H_{10}F^{79}Br$ (M^+): 299.9950, found 299.9935.

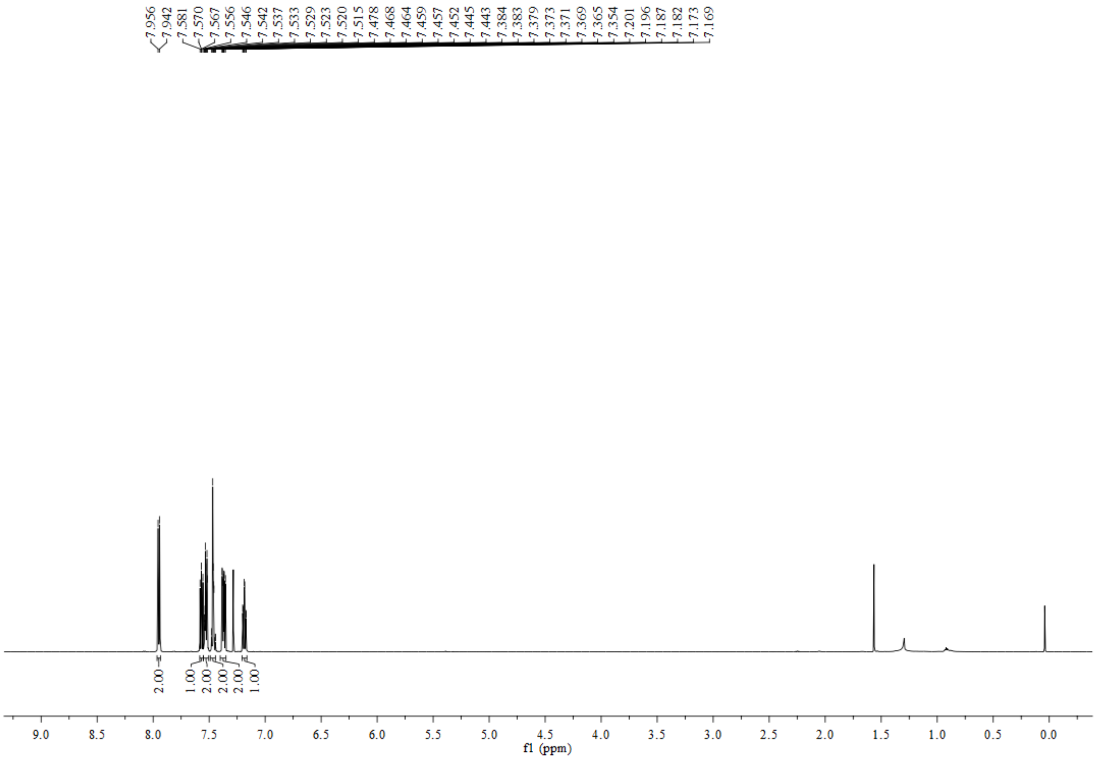

Supplementary Figure 61. ^{1}H NMR Spectra of compound **13d**.

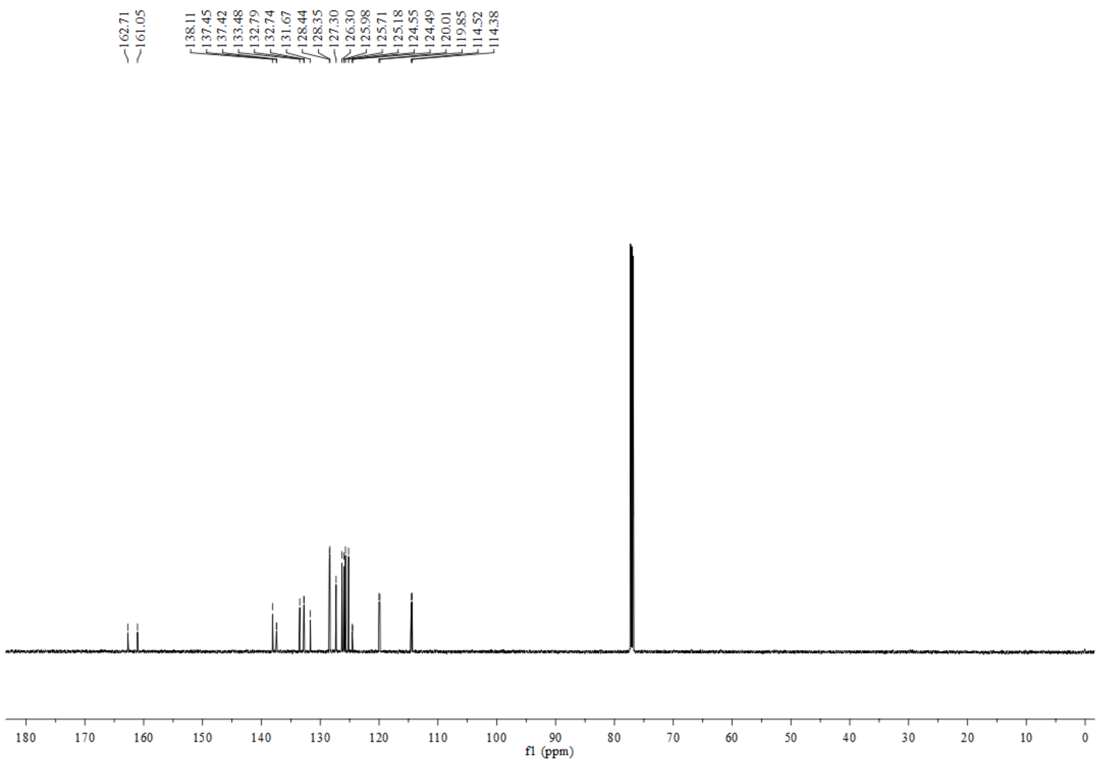

Supplementary Figure 62. ^{13}C NMR Spectra of compound **13d**.

1-(2-Bromo-5-methylphenyl)naphthalene (**13e**)

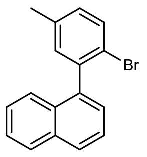

Purified by chromatography on silica gel, eluting with petroleum ether; white solid, Mp = 55-57 ^{o}C, 46% yield; ^{1}H NMR (600 MHz, $CDCl_3$): δ 7.97-7.94 (m, 2H), 7.65 (d, J = 7.8 Hz, 1H), 7.60-7.52 (m, 3H), 7.48-7.40 (m, 2H), 7.23 (s, 1H), 7.16 (d, J = 7.8 Hz, 1H), 2.40 (s, 3H). ^{13}C NMR (150 MHz, $CDCl_3$): δ 141.0, 139.2, 137.0, 133.4, 132.7, 132.4, 131.6, 129.9, 128.2, 128.1, 126.9, 126.1, 126.0, 125.8, 125.2, 120.9, 20.9. HRMS *m/z* (EI+): Calculated for $C_{17}H_{13}{}^{79}Br$ (M^{+}): 296.0201, found 296.0186.

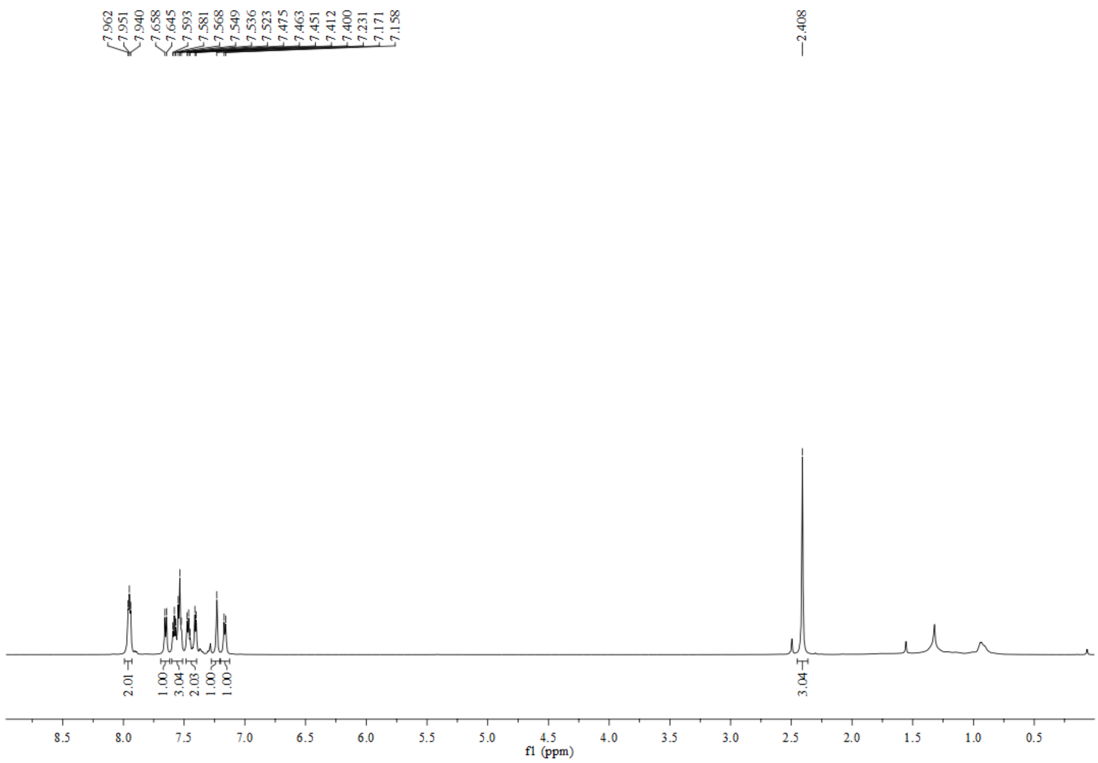

Supplementary Figure 63. ^{1}H NMR Spectra of compound **13e**.

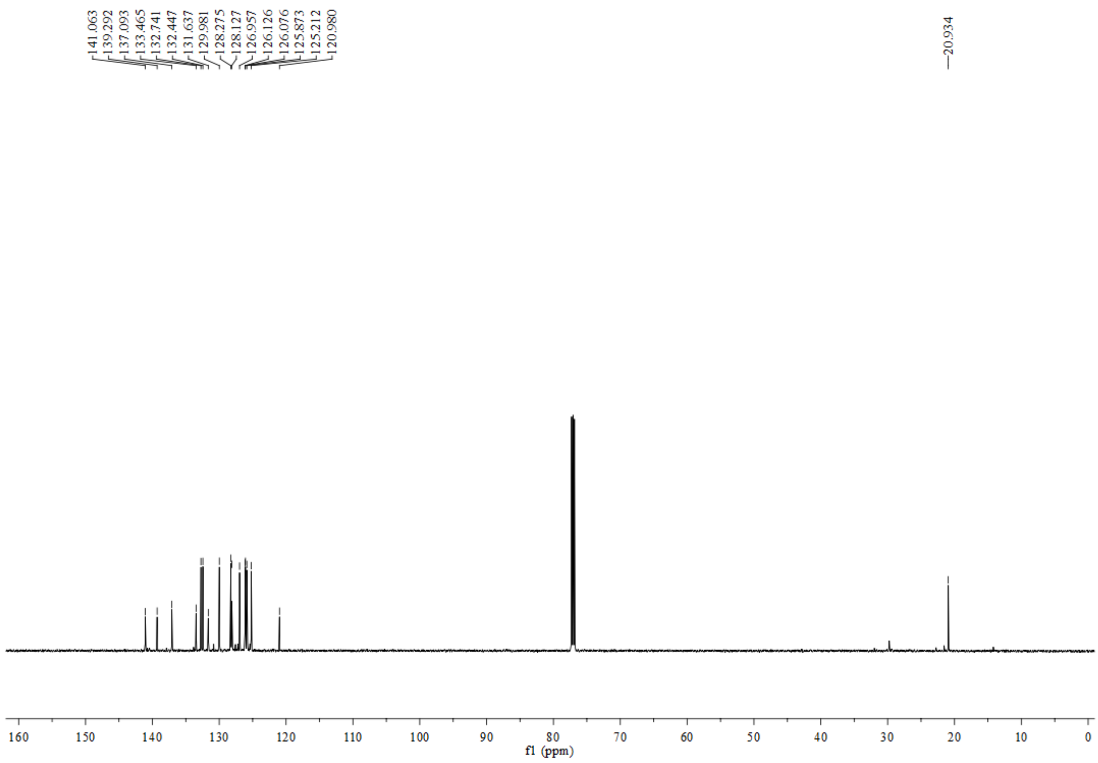

Supplementary Figure 64. ^{13}C NMR Spectra of compound **13e**.

1-(2-Bromo-5-fluorophenyl)naphthalene (**13f**)

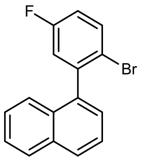

Purified by chromatography on silica gel, eluting with petroleum ether; white solid, Mp = 68-70 °C, 41% yield; ^{1}H NMR (600 MHz, $CDCl_3$): δ 7.96 (d, *J* = 7.8 Hz, 2H), 7.74-7.70 (m, 1H), 7.60-7.53 (m, 2H), 7.51-7.46 (m, 2H), 7.39 (d, *J* = 6.6 Hz, 1H), 7.16 (dd, *J* = 9.0, 3.0 Hz, 1H), 7.11-7.07 (m, 1H). ^{13}C NMR (150 MHz, $CDCl_3$): δ 161.7 (d, *J* = 246.0 Hz), 143.2 (d, *J* = 9.0 Hz), 138.1 (d, *J* = 1.5 Hz), 133.7 (d, *J* = 36.0 Hz), 133.4, 131.2, 128.6, 128.3, 126.9, 126.4, 126.0, 125.6, 125.1, 119.1 (d, *J* = 22.5 Hz), 118.7 (d, *J* = 3.0 Hz), 116.4 (d, *J* = 22.5 Hz). HRMS *m*/*z* (EI+): Calculated for $C_{16}H_{10}F^{79}Br$ (M^+): 299.9950, found 299.9941.

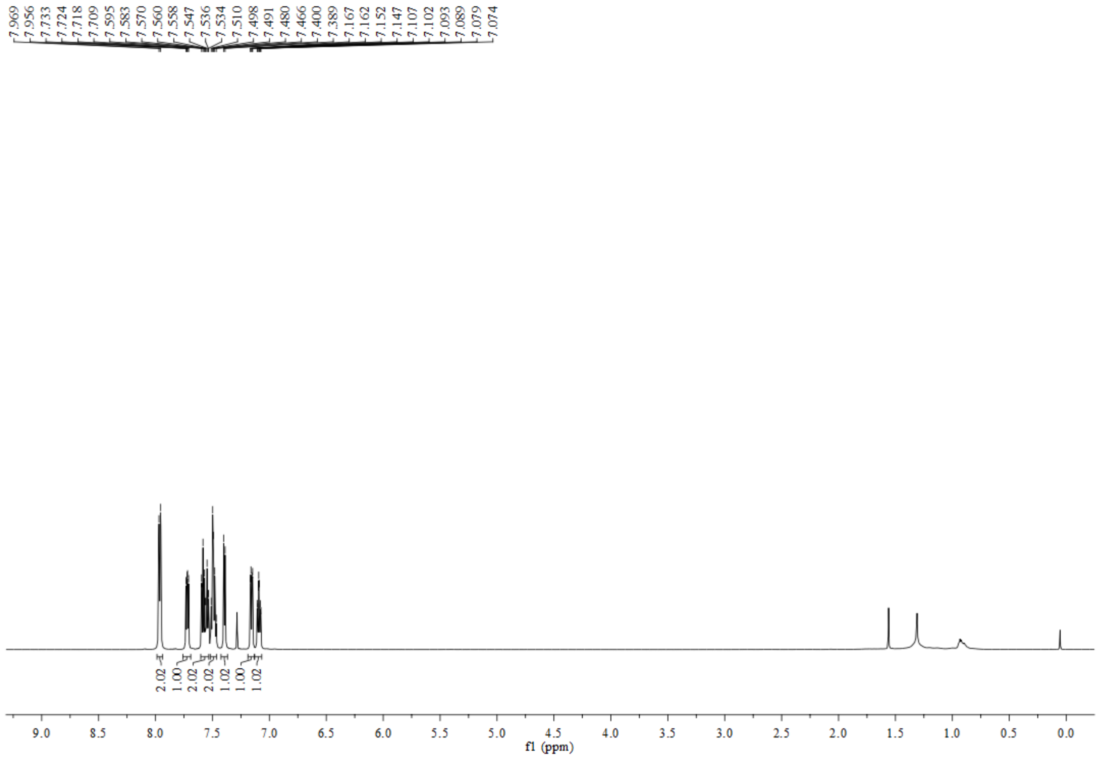

Supplementary Figure 65. ^{1}H NMR Spectra of compound **13f**.

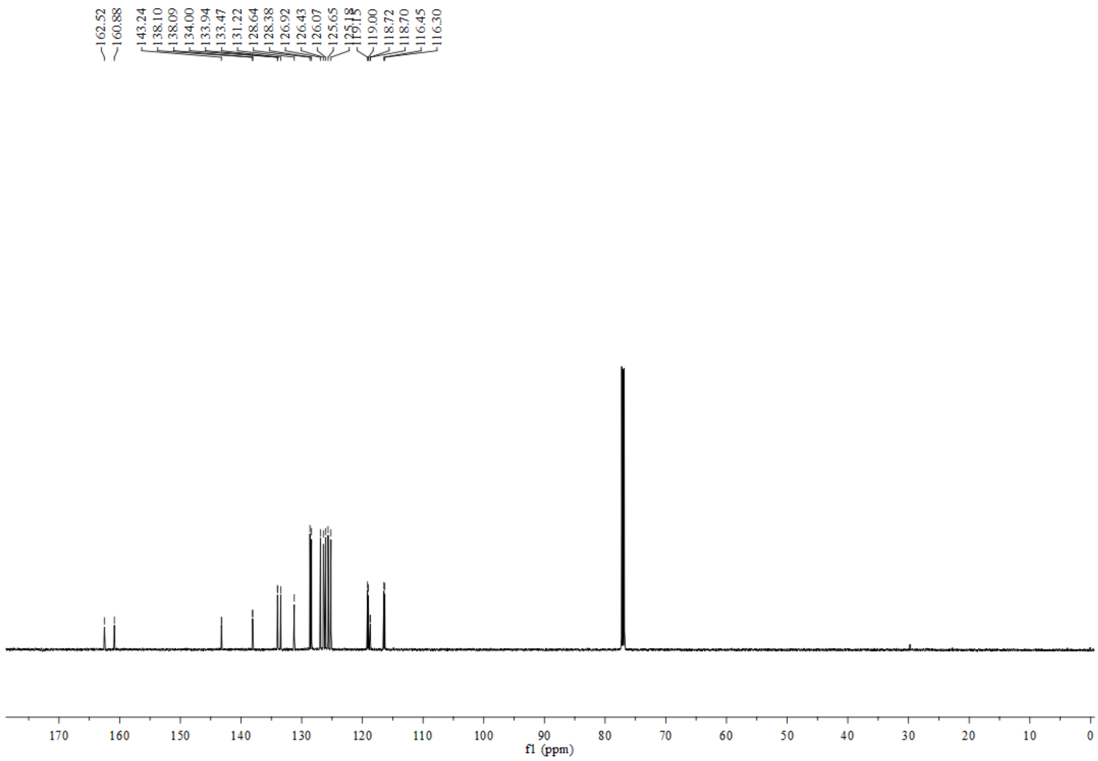

Supplementary Figure 66. ^{13}C NMR Spectra of compound **13f**.

Palladium-catalyzed dearomative 1,4-diarylation and vinylarylation of naphthalenes

General procedure for the dearomative 1,4-diarylation reaction

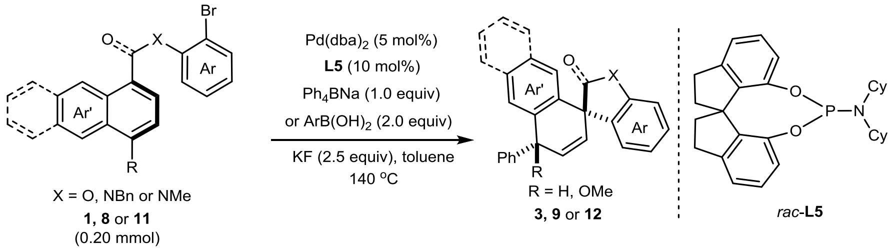

To a dried Schlenk tube were added **1**, **8** or **11** (0.20 mmol), $Pd(dba)_2$ (5.8 mg, 0.010 mmol), ligand **L5** (9.2 mg, 0.020 mmol), $NaBPh_4$ (68.4 mg, 0.20 mmol) or $ArB(OH)_2$ (0.40 mmol), KF (29.1 mg, 0.50 mmol) under N_2. 2.0 mL toluene was then introduced via syringe and the tube was sealed using Teflon cap. The mixture was stirred at 140 ^oC until the starting material **1**, **8 or 11** was consumed (monitored by TLC). The solvent was then removed under vacuum and the residue was purified by chromatography on silica gel, eluting with ethyl acetate/petroleum ether (*v*/*v*) to afford the products **3**, **9** or **12**.

1-Methyl-4'-phenyl-4'*H*-spiro[indoline-3,1'-naphthalen]-2-one (**3a**)

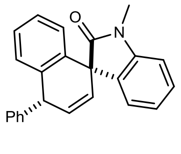

Purified by chromatography on silica gel, eluting with ethyl acetate/petroleum ether 1:15 (*v*/*v*); white solid, Mp = 148-150 ^oC, 56.2 mg, 83% yield; ^1H NMR (500 MHz, $CDCl_3$): δ 7.41-7.36 (m, 3H), 7.32-7.29 (m, 3H), 7.16 (d, J = 6.5 Hz, 1H), 7.11 (t, J = 7.5 Hz, 2H), 7.04-6.97 (m, 3H), 6.61-6.58 (m, 1H), 6.27 (dd, J = 10.0, 3.0 Hz, 1H), 5.61 (dd, J = 10.0, 2.5 Hz, 1H), 5.01 (s, 1H), 3.32 (s, 3H). ^{13}C NMR (150 MHz, $CDCl_3$): δ 178.0, 145.1, 143.9, 137.6, 135.5, 133.5, 132.6, 130.1, 128.9, 128.7, 128.6, 127.5, 126.9, 126.7, 124.8, 123.6, 123.3, 108.2, 54.7, 45.1, 26.7. HRMS *m*/*z* (ESI+): Calculated for $C_{24}H_{20}NO$ ($[M+H]^+$): 338.1539, found 338.1542.

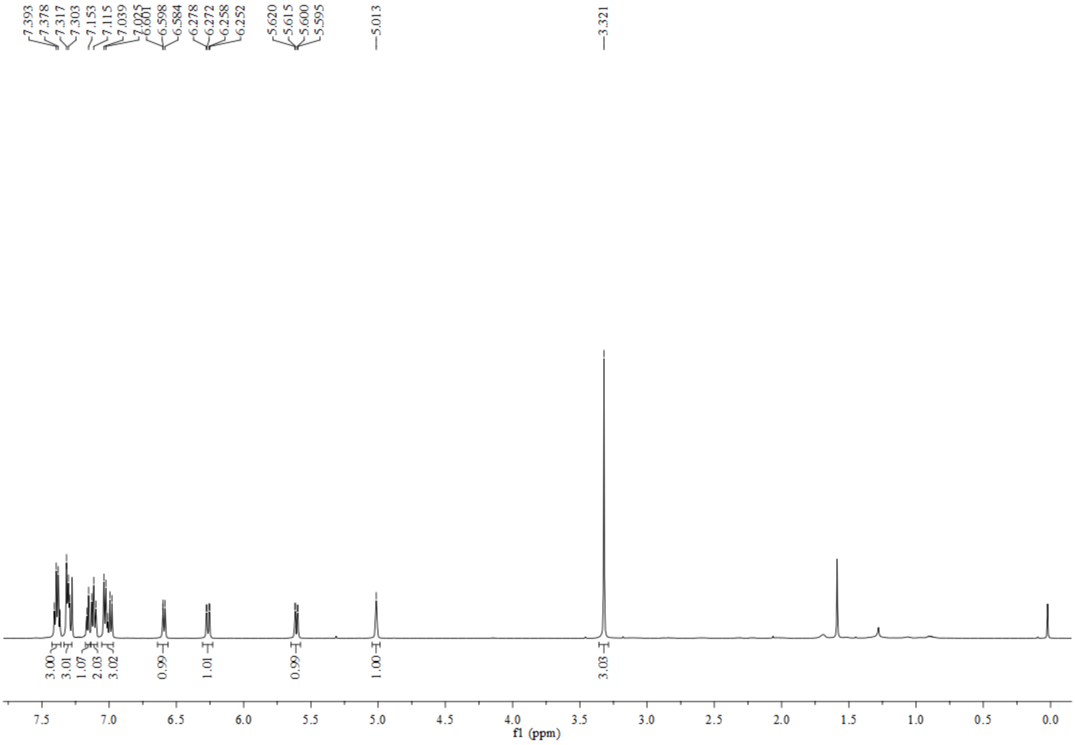

Supplementary Figure 67. ^{1}H NMR Spectra of compound **3a**.

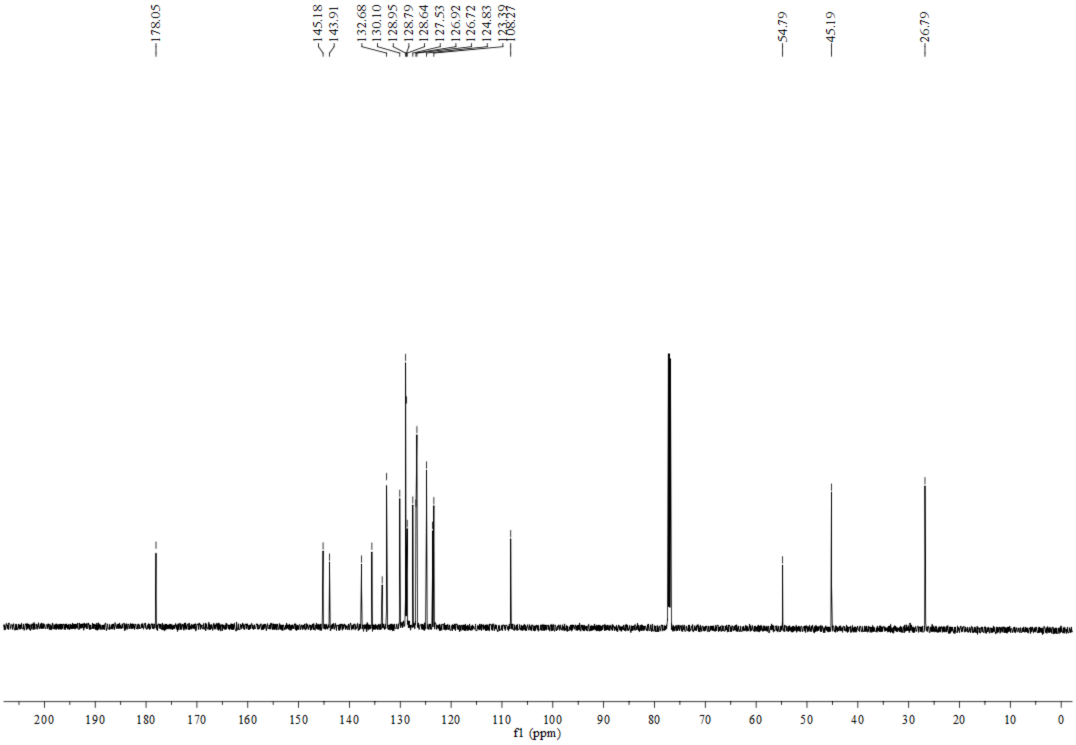

Supplementary Figure 68. ^{13}C NMR Spectra of compound **3a**.

N-([1,1'-biphenyl]-2-yl)-*N*-methyl-1-naphthamide (**4**)

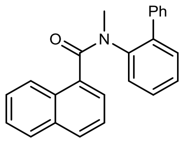

Purified by chromatography on silica gel, eluting with ethyl acetate/petroleum ether 1:10 (*v*/*v*); colourless oil; ^{1}H NMR (600 MHz, $CDCl_3$): δ 7.87 (d, *J* = 8.4 Hz, 1.00H), 7.70 (d, *J* = 8.4 Hz, 0.66H), 7.65 (d, *J* = 8.4 Hz, 0.64H), 7.62-7.47 (m, 6.16H), 7.38 (t, *J* = 7.2 Hz, 0.66H), 7.33-7.16 (m, 4.15H), 7.11-7.04 (m, 1.16H), 6.95-6.85 (m, 1.59H), 3.63 (s, 1.60H), 2.82 (s, 1.40H). ^{13}C NMR (150 MHz, $CDCl_3$): δ 171.2, 169.8, 141.6, 140.9, 140.5, 139.4, 139.2, 138.7, 134.5, 133.4, 133.19, 133.12, 131.3, 131.2, 130.8, 129.5, 129.2, 129.1, 129.0, 128.9, 128.4, 128.42, 128.41, 128.3, 128.2, 128.14, 128.11, 127.76, 127.74, 127.70, 127.6, 126.9, 126.5, 126.3, 125.7, 125.58, 125.51, 125.1, 124.9, 123.5, 40.3, 38.8. HRMS *m*/*z* (ESI+): Calculated for $C_{24}H_{19}NNaO$ ($[M+Na]^+$): 360.1359, found 360.1361.

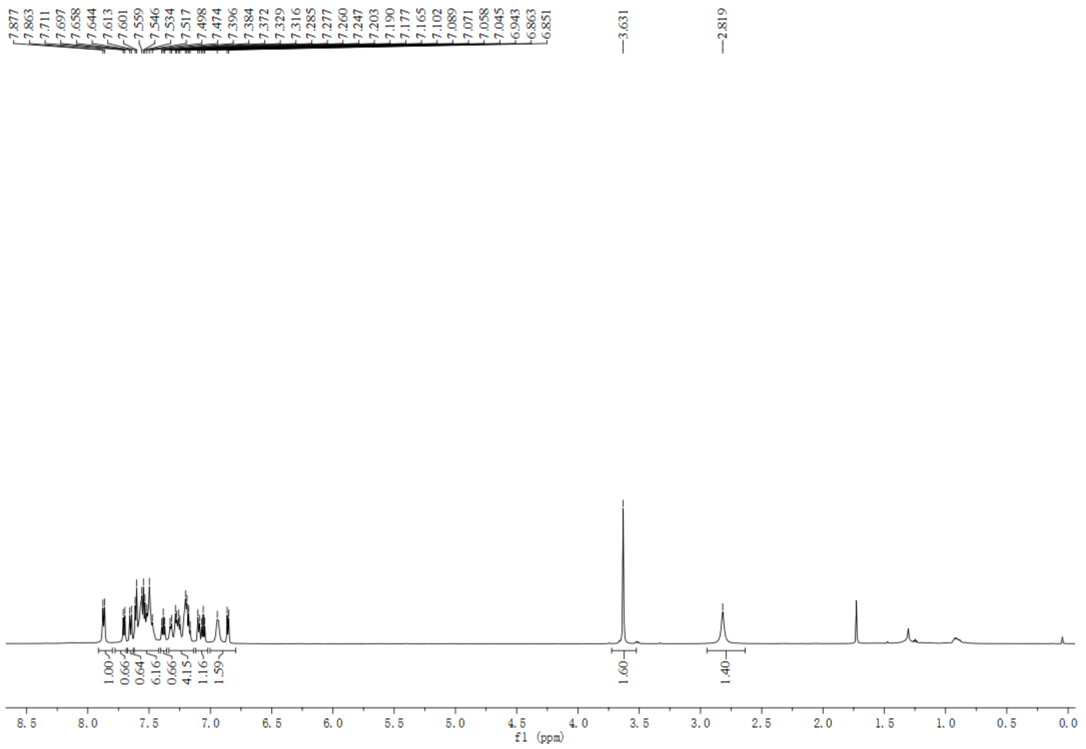

Supplementary Figure 69. ^{1}H NMR Spectra of compound **4**.

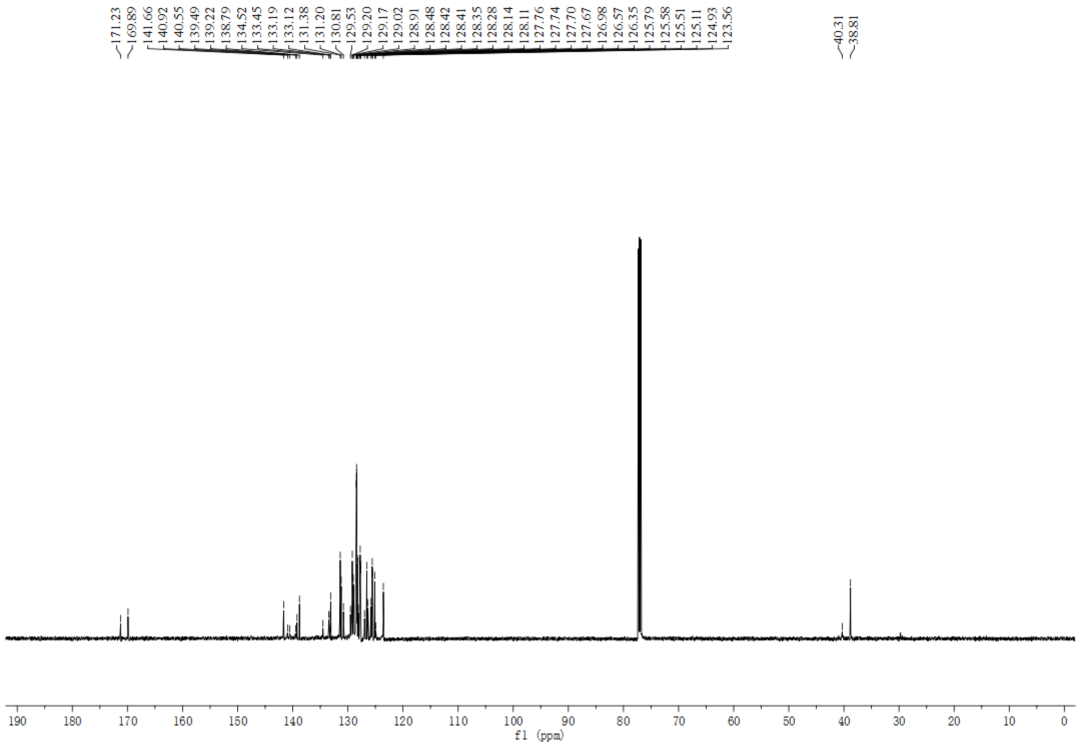

Supplementary Figure 70. ^{13}C NMR Spectra of compound **4**.

1-Methyl-2'*H*-spiro[indoline-3,1'-naphthalen]-2-one (**5**)[2]

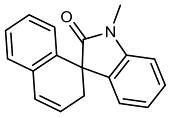

Purified by chromatography on silica gel, eluting with ethyl acetate/petroleum ether 1:15 (*v*/*v*); ^{1}H NMR (600 MHz, $CDCl_3$): δ 7.35 (d, *J* = 7.8 Hz, 1H), 7.29-7.26 (m, 1H), 7.23-7.17 (m, 2H), 7.09-7.06 (m, 1H), 6.97-6.90 (m, 2H), 6.75 (d, *J* = 7.8 Hz, 1H), 6.70 (dd, *J* = 9.6, 2.4 Hz, 1H), 6.08-6.04 (m, 1H), 3.35 (s, 3H), 3.10 (dt, *J* = 17.4, 3.0 Hz, 1H), 2.50 (dd, *J* = 16.8, 5.4 Hz, 1H).

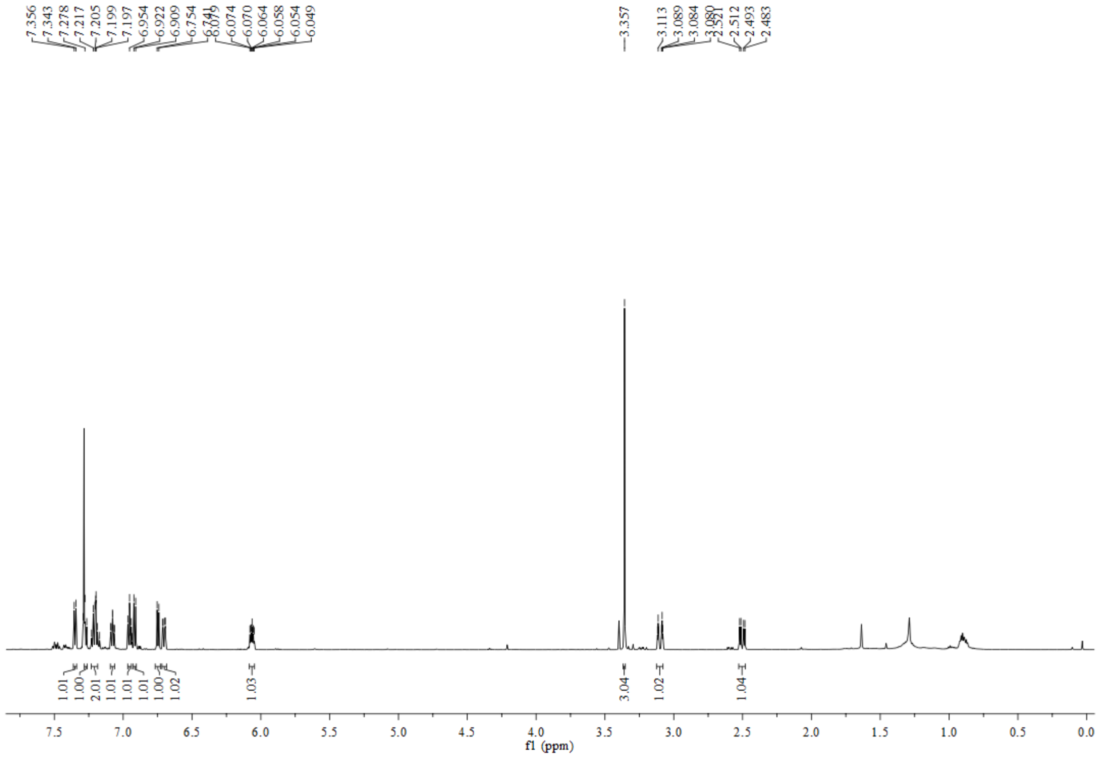

Supplementary Figure 71. ^{1}H NMR Spectra of compound **5**.

1-Methyl-2'-phenyl-2'*H*-spiro[indoline-3,1'-naphthalen]-2-one (**6**)

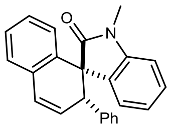

Purified by chromatography on silica gel, eluting with ethyl acetate/petroleum ether 1:15 (*v*/*v*); white solid, Mp = 171-173 ^{o}C; ^{1}H NMR (500 MHz, $CDCl_3$): δ 7.33-7.31 (m, 1H), 7.28-7.23 (m, 2H), 7.15-7.06 (m, 3H), 7.05-7.01 (m, 2H), 6.95-6.92 (m, 2H), 6.89-6.85 (m, 1H), 6.85-6.82 (m, 2H), 6.49 (d, *J* = 7.5 Hz, 1H), 6.13 (dd, *J* = 9.5, 2.5 Hz, 1H), 4.62 (t, *J* = 3.0 Hz, 1H), 2.99 (s, 3H). ^{13}C NMR (125 MHz, $CDCl_3$): δ 179.3, 141.5, 138.2, 134.7, 133.0, 130.3, 130.2, 129.16, 129.14, 128.6, 128.1, 127.9, 127.29, 127.22, 126.9, 126.2, 124.8, 122.1, 107.8, 59.0, 49.6, 26.0. HRMS *m*/*z* (ESI+): Calculated for $C_{24}H_{19}NNaO$ ($[M+Na]^+$): 360.1359, found 360.1359.

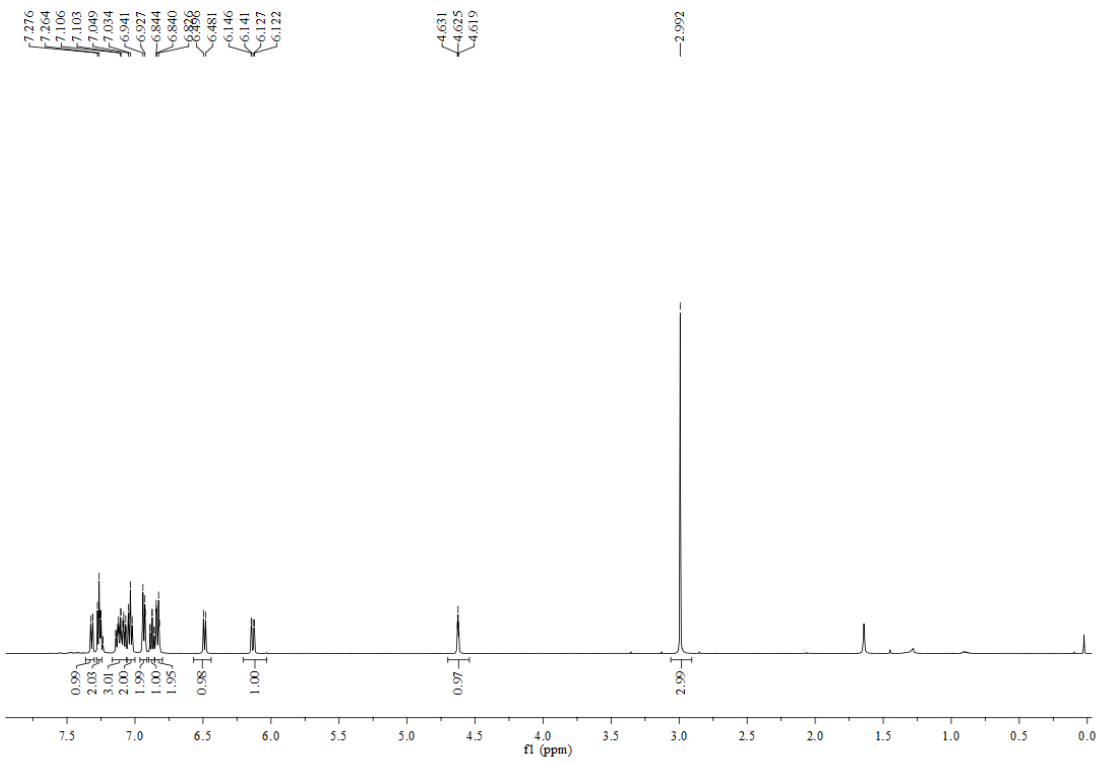

Supplementary Figure 72. ^{1}H NMR Spectra of compound **6**.

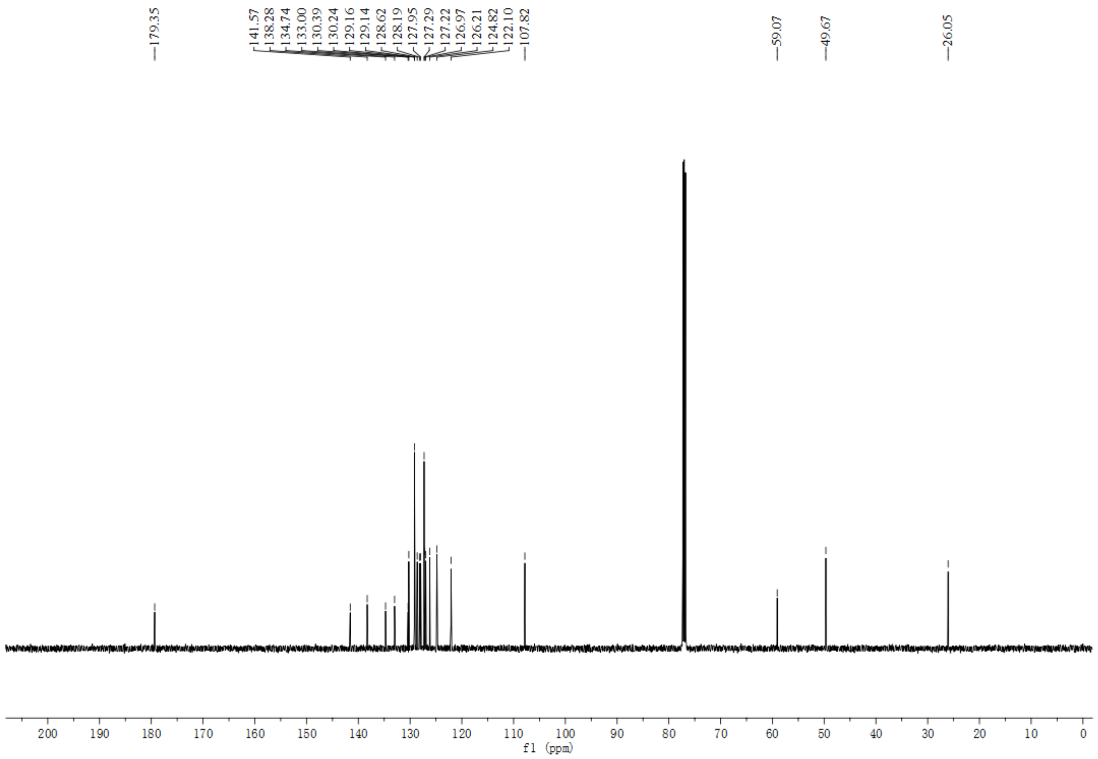

Supplementary Figure 73. ^{13}C NMR Spectra of compound **6**.

6-Methylbenzo[*i*]phenanthridin-5(6*H*)-one (**7**) [2]

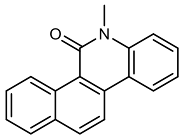

Purified by chromatography on silica gel, eluting with ethyl acetate/petroleum ether 1:20 (*v*/*v*); ^{1}H NMR (500 MHz, $CDCl_3$): δ 10.28 (d, *J* = 8.5 Hz, 1H), 8.39-8.32 (m, 2H), 8.13 (d, *J* = 9.0 Hz, 1H), 7.93 (d, *J* = 7.5 Hz, 1H), 7.79-7.74 (m, 1H), 7.66-7.58 (m, 2H), 7.45 (d, *J* = 8.0 Hz, 1H), 7.37-7.33 (m, 1H), 3.89 (s, 3H).

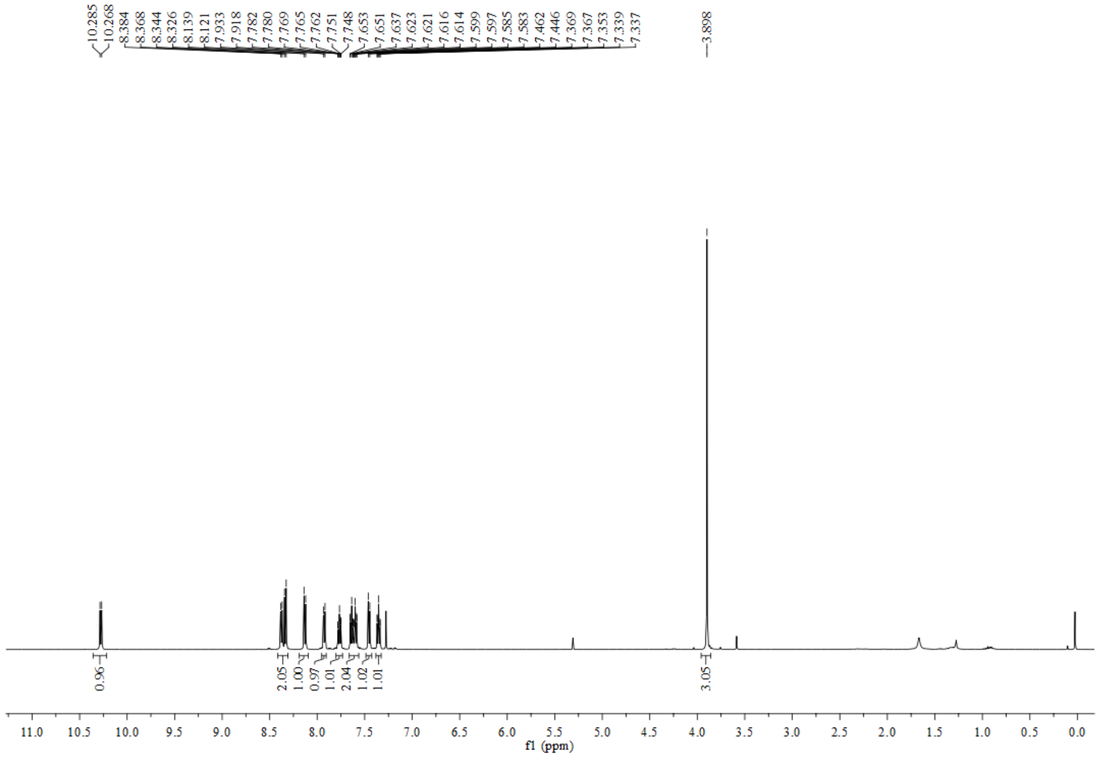

Supplementary Figure 74. ^{1}H NMR Spectra of compound **7**.

1,4-Dimethyl-4'-phenyl-4'*H*-spiro[indoline-3,1'-naphthalen]-2-one (**3b**)

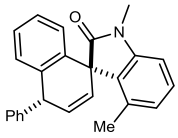

Purified by chromatography on silica gel, eluting with ethyl acetate/petroleum ether 1:15 (*v*/*v*); white solid, Mp = 146-148 ^{o}C, 58.0 mg, 83% yield; ^{1}H NMR (500 MHz,

$CDCl_3$): δ 7.41-7.37 (m, 2H), 7.34-7.30 (m, 2H), 7.29-7.26 (m, 2H), 7.13-7.09 (m, 1H), 7.03-6.99 (m, 1H), 6.96 (d, *J* = 8.0 Hz, 1H), 6.92 (d, *J* = 7.5 Hz, 1H), 6.84 (d, *J* = 8.0 Hz, 1H), 6.57 (dd, *J* = 7.5, 1.0 Hz, 1H), 6.30 (dd, *J* = 9.5, 2.0 Hz, 1H), 5.66 (dd, *J* = 9.5, 2.5 Hz, 1H), 5.08 (s, 1H), 3.25 (s, 3H), 2.07 (s, 3H). ^{13}C NMR (125 MHz, $CDCl_3$): δ 177.3, 145.4, 144.2, 138.0, 135.3, 134.0, 132.9, 131.7, 130.2, 129.2, 128.7, 128.6, 127.4, 126.7, 126.6, 125.5, 125.1, 123.7, 105.7, 55.0, 45.4, 26.8, 18.0. HRMS *m/z* (ESI+): Calculated for $C_{25}H_{22}NO$ ($[M+H]^+$): 352.1696, found 352.1695.

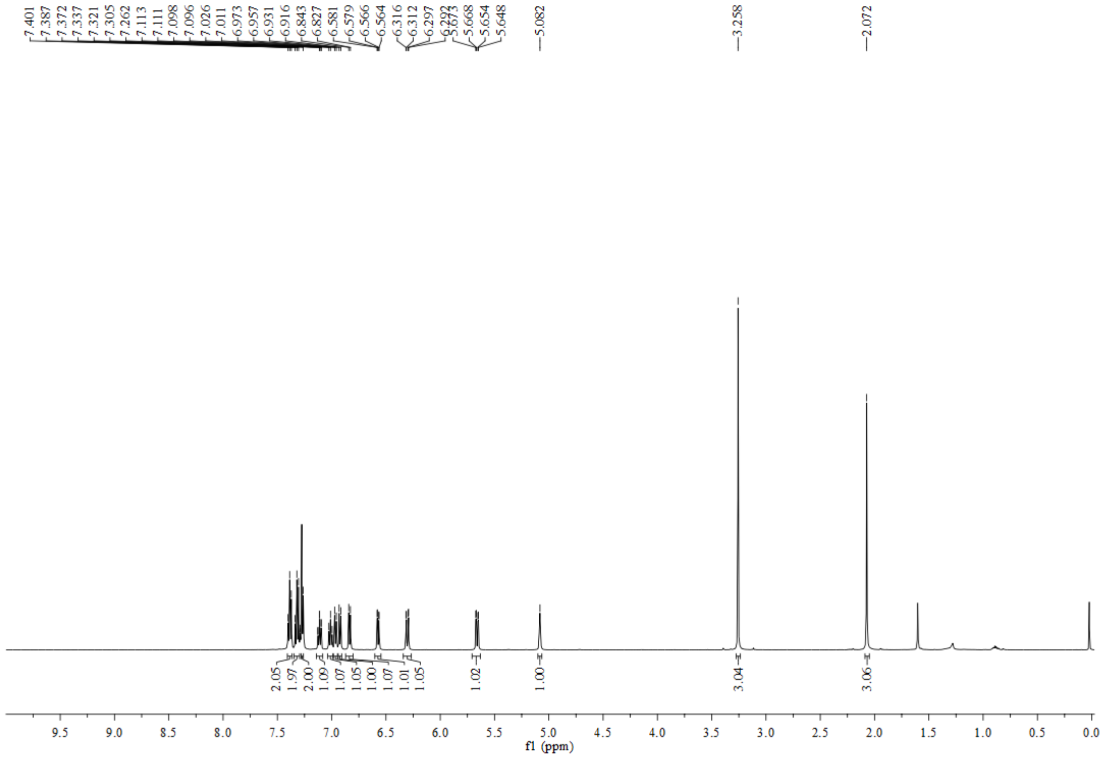

Supplementary Figure 75. ^{1}H NMR Spectra of compound **3b**.

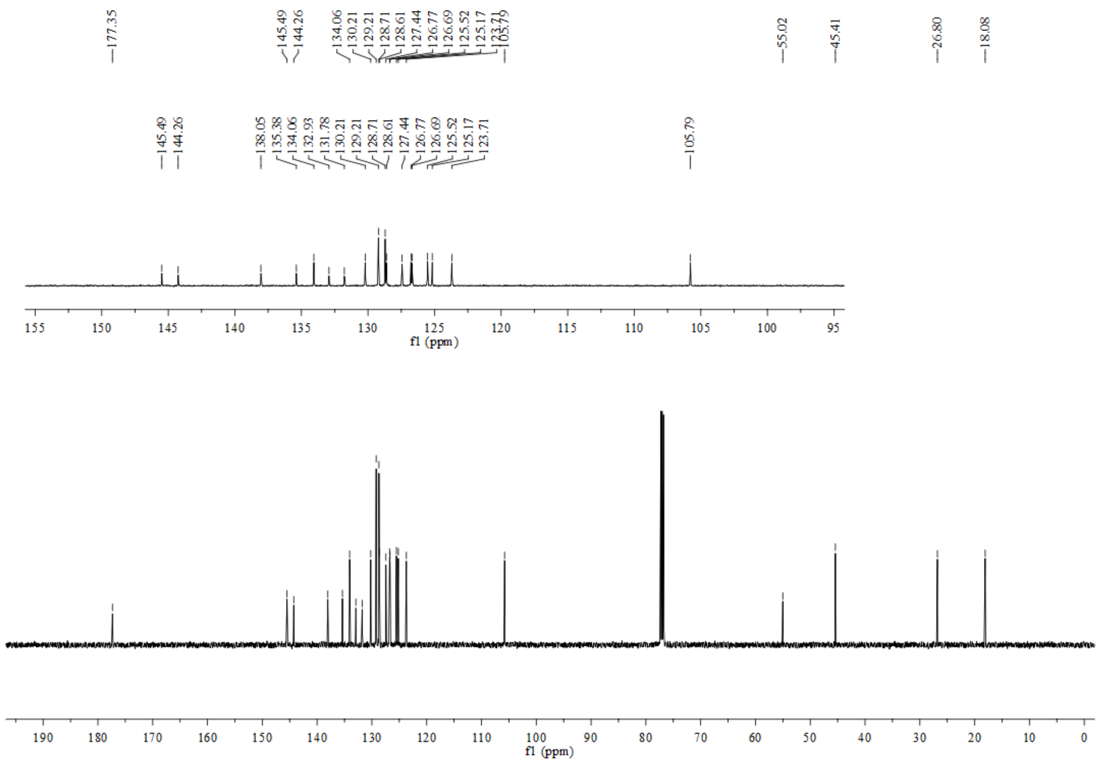

Supplementary Figure 76. ^{13}C NMR Spectra of compound **3b**.

4-Methoxy-1-methyl-4'-phenyl-4'*H*-spiro[indoline-3,1'-naphthalen]-2-one (**3c**)

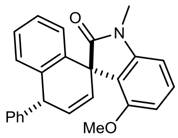

Purified by chromatography on silica gel, eluting with ethyl acetate/petroleum ether 1:15 (*v*/*v*); white solid, Mp = 194-196 °C, 66.1 mg, 90% yield; ^{1}H NMR (500 MHz, $CDCl_3$): δ 7.40-7.33 (m, 5H), 7.29-7.25 (m, 1H), 7.12-7.07 (m, 1H), 7.03-6.98 (m, 2H), 6.67 (t, *J* = 8.0 Hz, 2H), 6.63-6.61 (m, 1H), 6.23 (dd, *J* = 10.0, 3.0 Hz, 1H), 5.57 (dd, *J* = 9.5, 2.5 Hz, 1H), 4.98 (t, *J* = 2.5 Hz, 1H), 3.70 (s, 3H), 3.28 (s, 3H). ^{13}C NMR (150 MHz, $CDCl_3$): δ 178.0, 155.9, 146.0, 145.3, 137.5, 132.7, 132.3, 130.3, 130.0, 129.2, 128.4, 127.2, 126.6, 126.4, 125.5, 122.5, 120.5, 106.7, 101.5, 55.4, 53.8, 45.3, 27.0. HRMS *m*/*z* (ESI+): Calculated for $C_{25}H_{22}NO_2$ ([M+H]$^+$): 368.1645, found 368.1645.

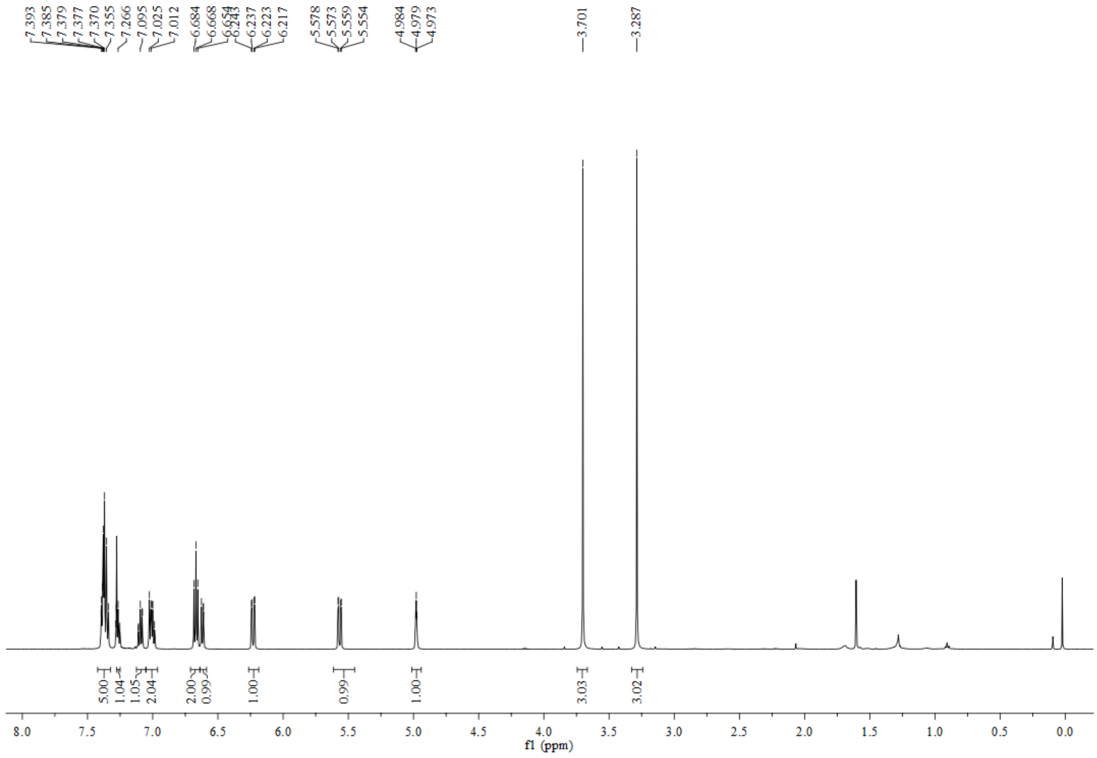

Supplementary Figure 77. ^{1}H NMR Spectra of compound **3c**.

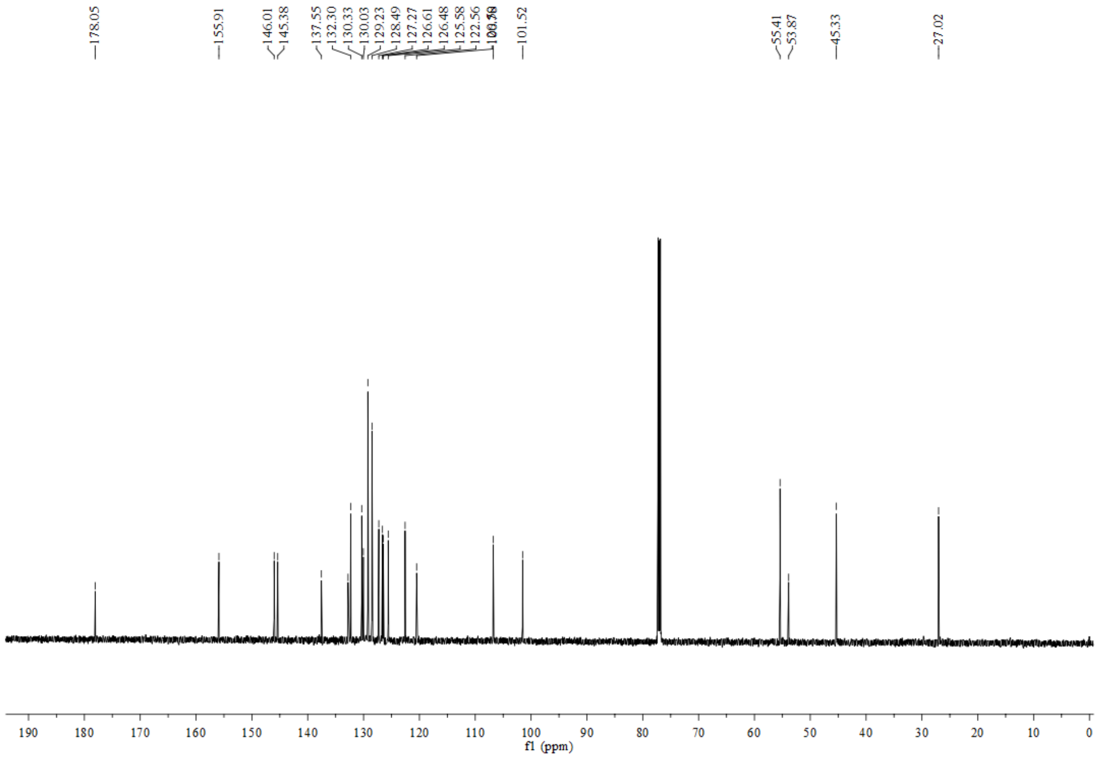

Supplementary Figure 78. ^{13}C NMR Spectra of compound **3c**.

4-Fluoro-1-methyl-4'-phenyl-4'*H*-spiro[indoline-3,1'-naphthalen]-2-one (**3d**)

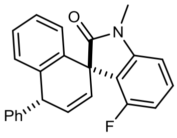

Purified by chromatography on silica gel, eluting with ethyl acetate/petroleum ether 1:15 (*v*/*v*); white solid, Mp = 156-158 °C, 61.7 mg, 87% yield; ^{1}H NMR (500 MHz, $CDCl_3$): δ 7.40-7.31 (m, 5H), 7.30-7.25 (m, 1H), 7.15-7.11 (m, 1H), 7.06-7.01 (m, 2H), 6.83-6.78 (m, 2H), 6.65-6.63 (m, 1H), 6.29 (dd, *J* = 10.0, 3.0 Hz, 1H), 5.63 (dd, *J* = 10.0, 2.5 Hz, 1H), 4.99 (t, *J* = 2.5 Hz, 1H), 3.30 (s, 3H). ^{13}C NMR (125 MHz, $CDCl_3$): δ 177.2, 158.7 (d, *J* = 248.8 Hz), 145.9 (d, *J* = 8.8 Hz), 145.1, 137.6, 133.2, 131.8, 130.5 (d, *J* = 8.8 Hz), 130.4, 129.1 (d, *J* = 2.5 Hz), 128.6, 127.7, 126.8, 126.6, 125.6, 121.8, 120.4 (d, *J* = 18.8 Hz), 110.9 (d, *J* = 20.0 Hz), 104.3 (d, *J* =2.5 Hz), 53.3, 45.3, 27.1. HRMS *m*/*z* (ESI+): Calculated for $C_{24}H_{19}FNO$ ($[M+H]^+$): 356.1445, found 356.1443.

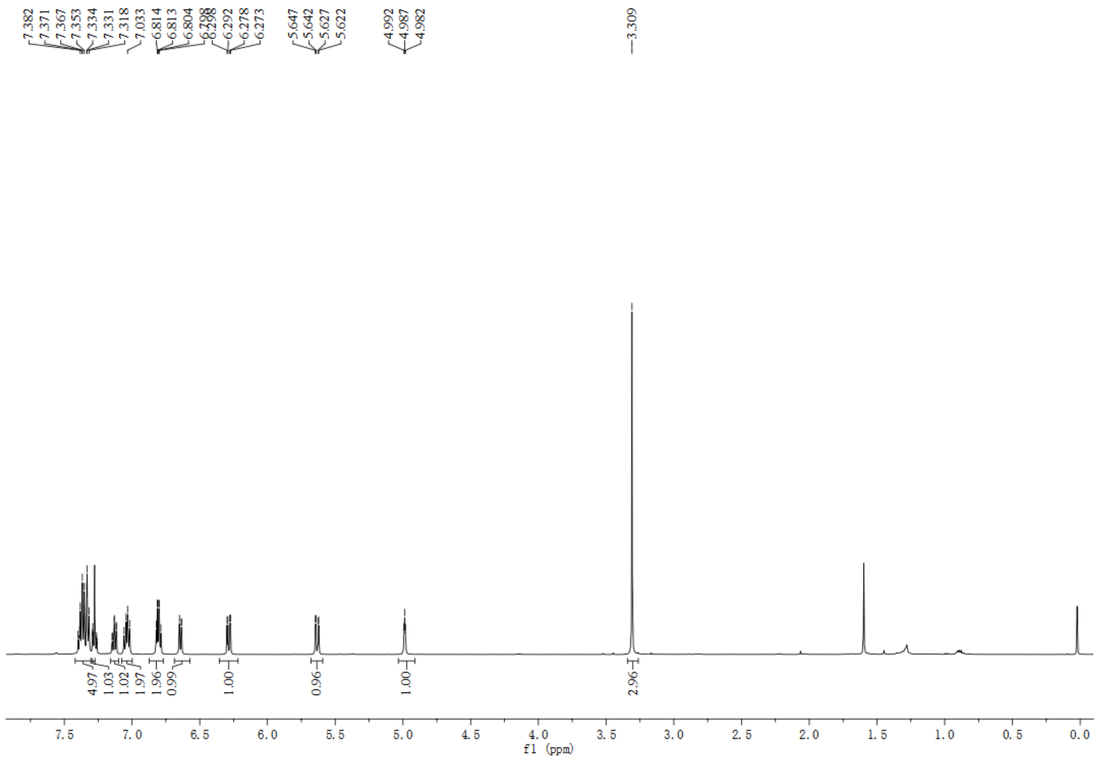

Supplementary Figure 79. ^{1}H NMR Spectra of compound **3d**.

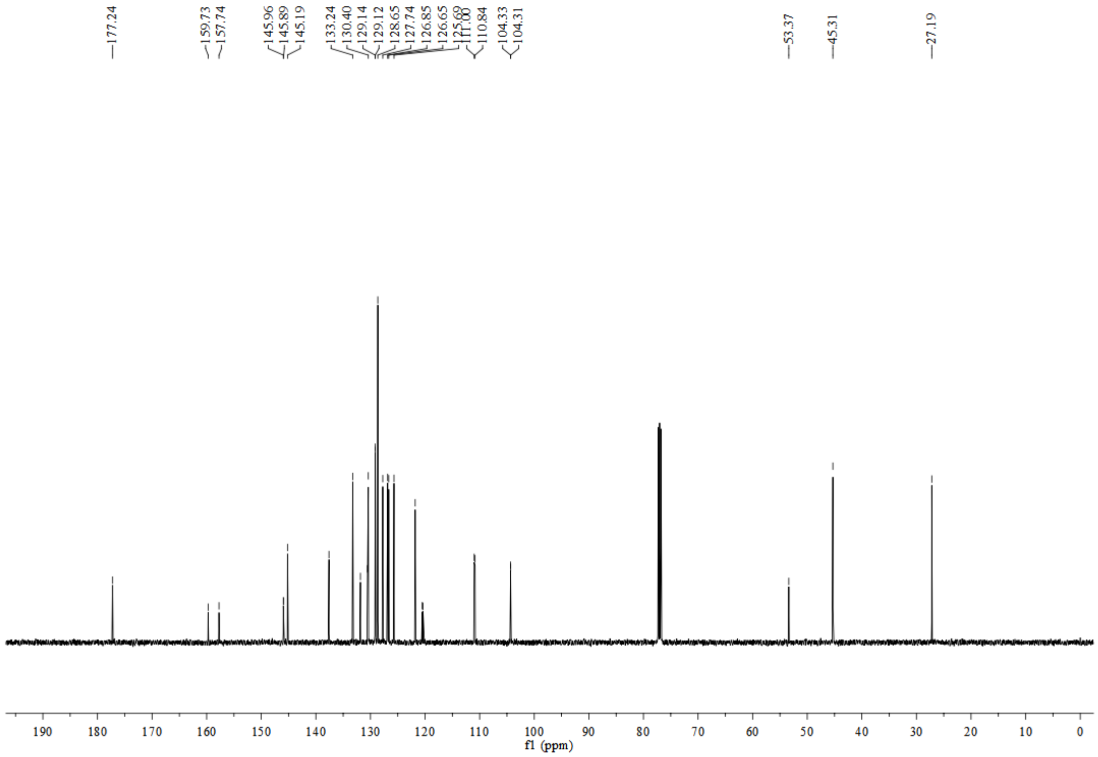

Supplementary Figure 80. ^{13}C NMR Spectra of compound **3d**.

1-Methyl-4'-phenyl-4-(trifluoromethyl)-4'*H*-spiro[indoline-3,1'-naphthalen]-2-one (**3e**)

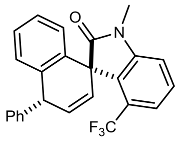

Purified by chromatography on silica gel, eluting with ethyl acetate/petroleum ether 1:15 (*v*/*v*); pale yellow solid, Mp = 237-239 °C, 67.3 mg, 83% yield; ^{1}H NMR (600 MHz, $CDCl_3$): δ 7.58 (t, *J* = 7.8 Hz, 1H), 7.44 (d, *J* = 8.4 Hz, 1H), 7.42-7.39 (m, 2H), 7.33-7.30 (m, 3H), 7.19 (d, *J* = 8.4 Hz, 1H), 7.13-7.10 (m, 1H), 7.02-6.98 (m, 1H), 6.96 (d, *J* = 7.8 Hz, 1H), 6.45 (dd, *J* = 7.8, 1.2 Hz, 1H), 6.28 (dd, *J* = 9.6, 2.4 Hz, 1H), 5.72 (dd, *J* = 9.6, 3.0 Hz, 1H), 5.06 (s, 1H), 3.29 (s, 3H). ^{13}C NMR (150 MHz, $CDCl_3$): δ 176.7, 145.8, 145.2, 137.4, 133.4, 133.2, 131.2, 130.3, 129.5, 129.3, 128.7, 127.7 (q, *J* = 34.5 Hz), 127.5, 126.7, 126.5, 125.0, 123.5 (q, *J* =273.0 Hz), 123.2, 120.8 (q, *J* = 6.0 Hz), 111.5, 55.7, 45.4, 27.0. HRMS *m*/*z* (ESI+): Calculated for $C_{25}H_{19}F_3NO$ ($[M+H]^+$): 406.1413, found 406.1413.

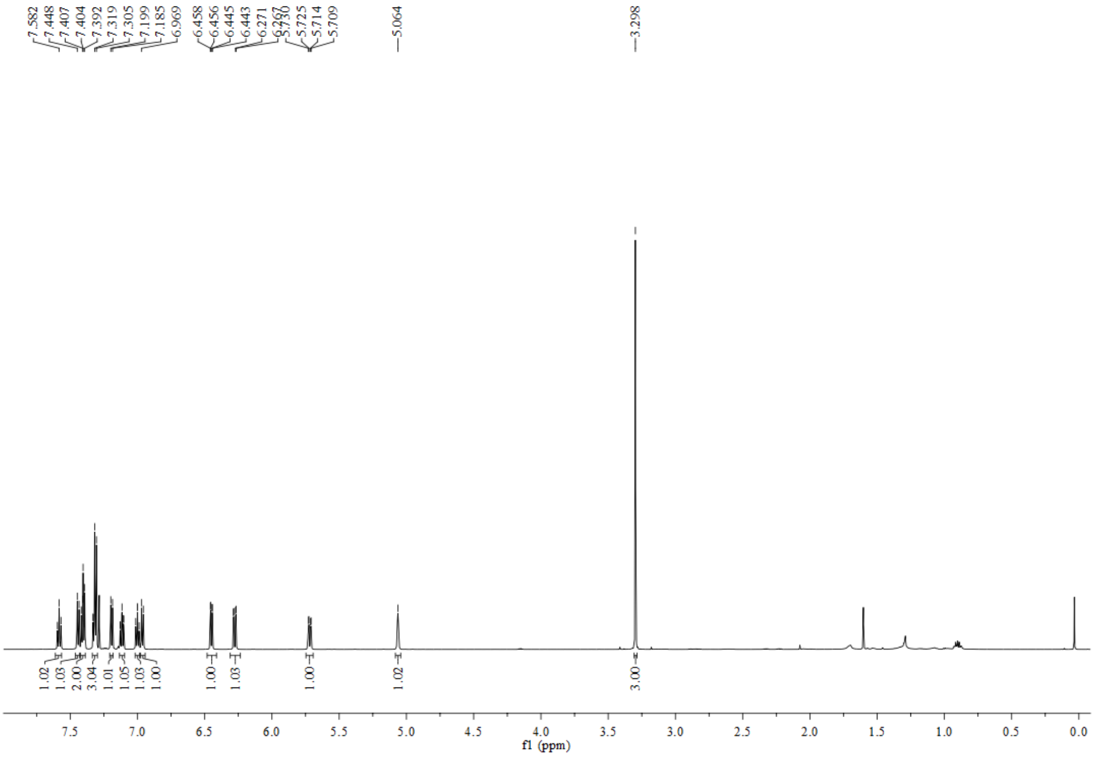

Supplementary Figure 81. ^{1}H NMR Spectra of compound **3e**.

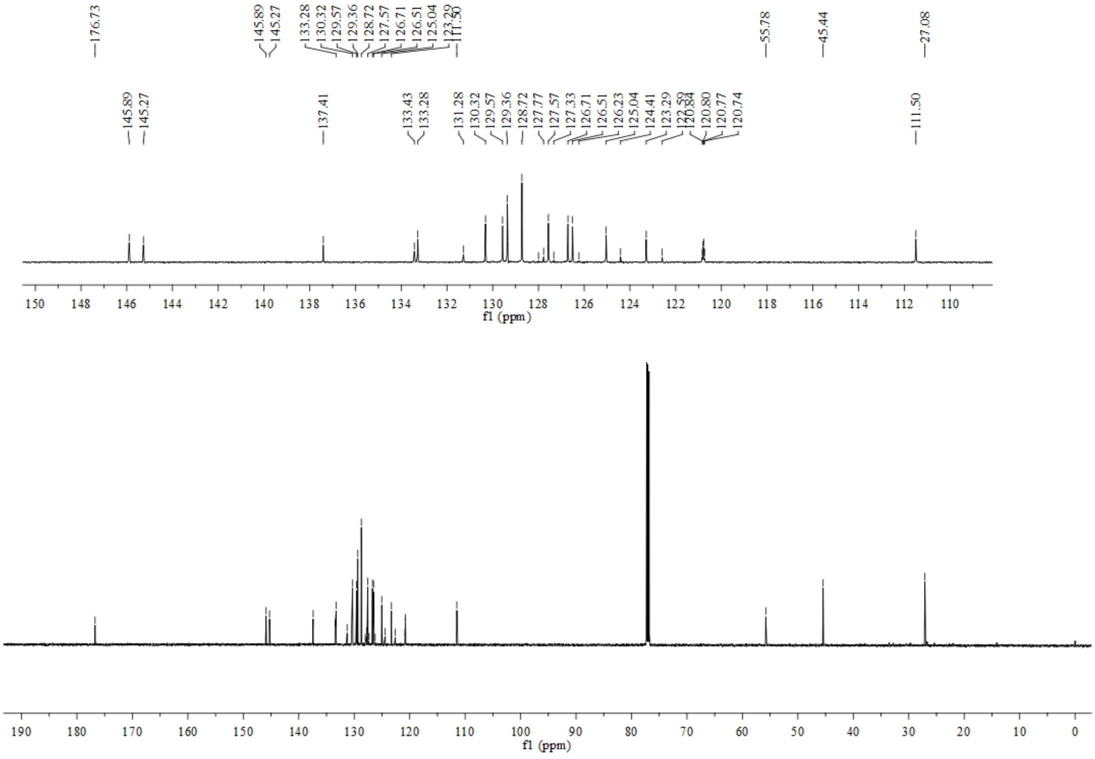

Supplementary Figure 82. ^{13}C NMR Spectra of compound **3e**.

1,5-Dimethyl-4'-phenyl-4'*H*-spiro[indoline-3,1'-naphthalen]-2-one (**3f**)

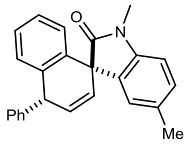

Purified by chromatography on silica gel, eluting with ethyl acetate/petroleum ether 1:15 (*v*/*v*); white solid, Mp = 146-148 °C, 64.3 mg, 91% yield; ^{1}H NMR (600 MHz, $CDCl_3$): δ 7.43-7.39 (m, 2H), 7.34-7.30 (m, 3H), 7.20-7.18 (m, 1H), 7.15-7.11 (m, 1H), 7.06-7.03 (m, *J* = 7.0 Hz, 2H), 6.97 (s, 1H), 6.89 (d, *J* = 7.8 Hz, 1H), 6.62 (d, *J* = 8.4 Hz, 1H), 6.27 (dd, *J* = 10.2, 3.0 Hz, 1H), 5.62 (dd, *J* = 9.6, 1.2 Hz, 1H), 5.02 (s, 1H), 3.31 (s, 3H), 2.34 (s, 3H). ^{13}C NMR (150 MHz, $CDCl_3$): δ 177.9, 145.2, 141.5, 137.6, 135.5, 133.7, 132.9, 132.5, 130.0, 128.9, 128.8, 128.7, 127.4, 126.8, 126.7, 126.6, 125.5, 123.8, 107.9, 54.8, 45.1, 26.8, 21.1. HRMS *m*/*z* (ESI+): Calculated for $C_{25}H_{22}NO$ ($[M+H]^+$): 352.1696, found 352.1694.

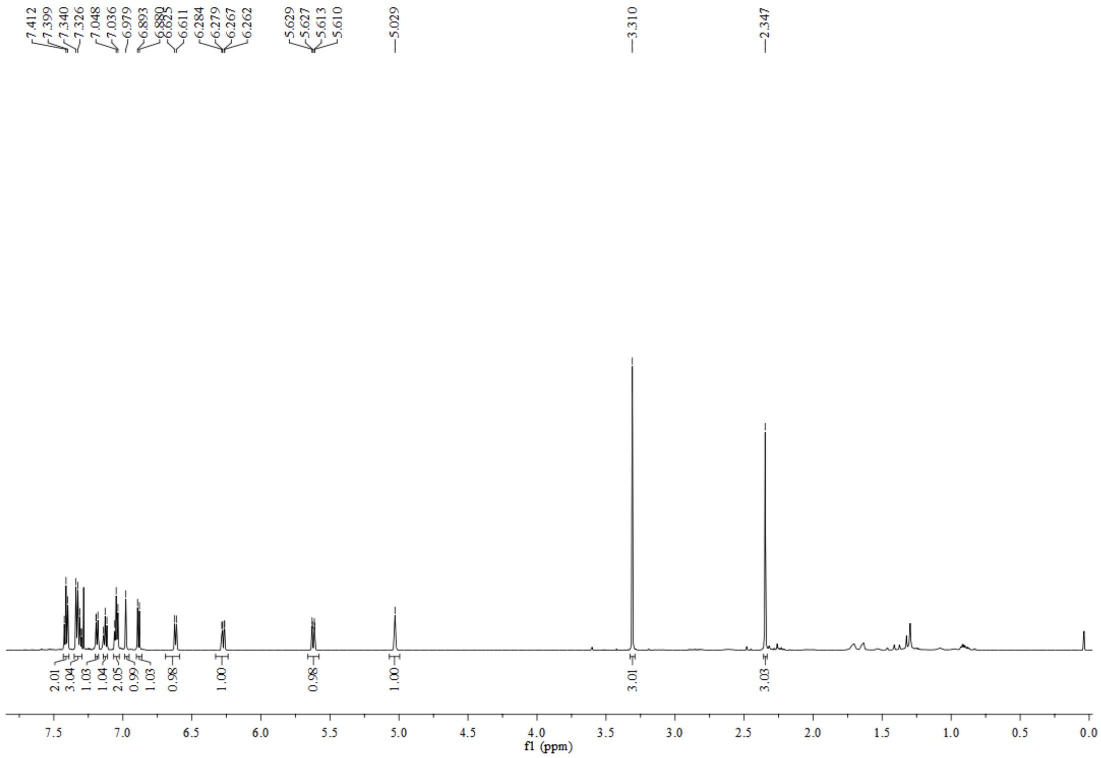

Supplementary Figure 83. ^{1}H NMR Spectra of compound **3f**.

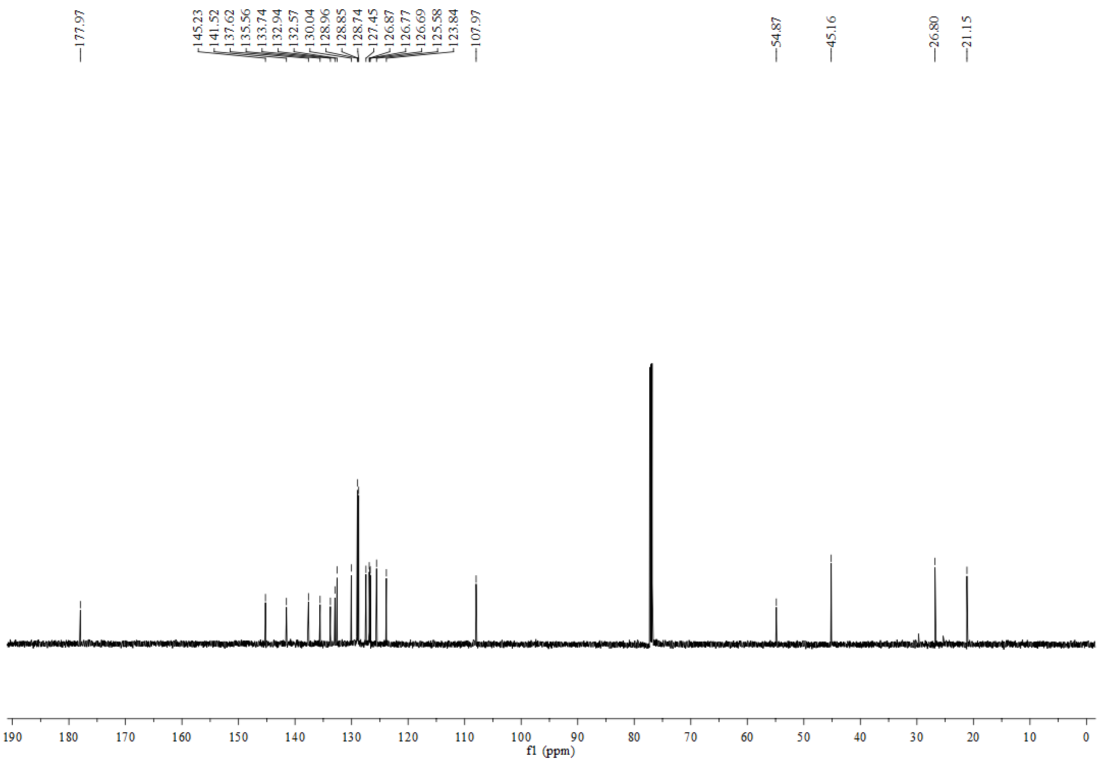

Supplementary Figure 84. ^{13}C NMR Spectra of compound **3f**.

5-Methoxy-1-methyl-4'-phenyl-4'*H*-spiro[indoline-3,1'-naphthalen]-2-one (**3g**)

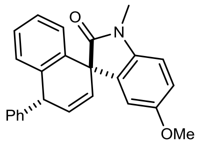

Purified by chromatography on silica gel, eluting with ethyl acetate/petroleum ether 1:15 (*v*/*v*); white solid, Mp = 187-189 °C, 56.9 mg, 77% yield; ^{1}H NMR (500 MHz, $CDCl_3$): δ 7.41-7.37 (m, 2H), 7.32-7.28 (m, 3H), 7.14-7.10 (m, 1H), 7.06-7.02 (m, 2H), 6.92-6.87 (m, 2H), 6.77-6.75 (m, 1H), 6.63-6.61 (m, 1H), 6.28 (dd, *J* = 10.0, 3.5 Hz, 1H), 5.62 (dd, *J* = 10.0, 2.5 Hz, 1H), 5.02 (t, *J* = 2.5 Hz, 1H), 3.77 (s, 3H), 3.29 (s, 3H). ^{13}C NMR (125 MHz, $CDCl_3$): δ 177.7, 156.6, 145.1, 137.5, 137.3, 136.7, 133.6, 132.7, 130.0, 128.8, 128.7, 127.5, 126.9, 126.7, 126.6, 123.7, 113.1, 111.9, 108.5, 55.7, 55.2, 45.1, 26.8. HRMS *m*/*z* (ESI+): Calculated for $C_{25}H_{22}NO_2$ ($[M+H]^+$): 368.1645, found 368.1641.

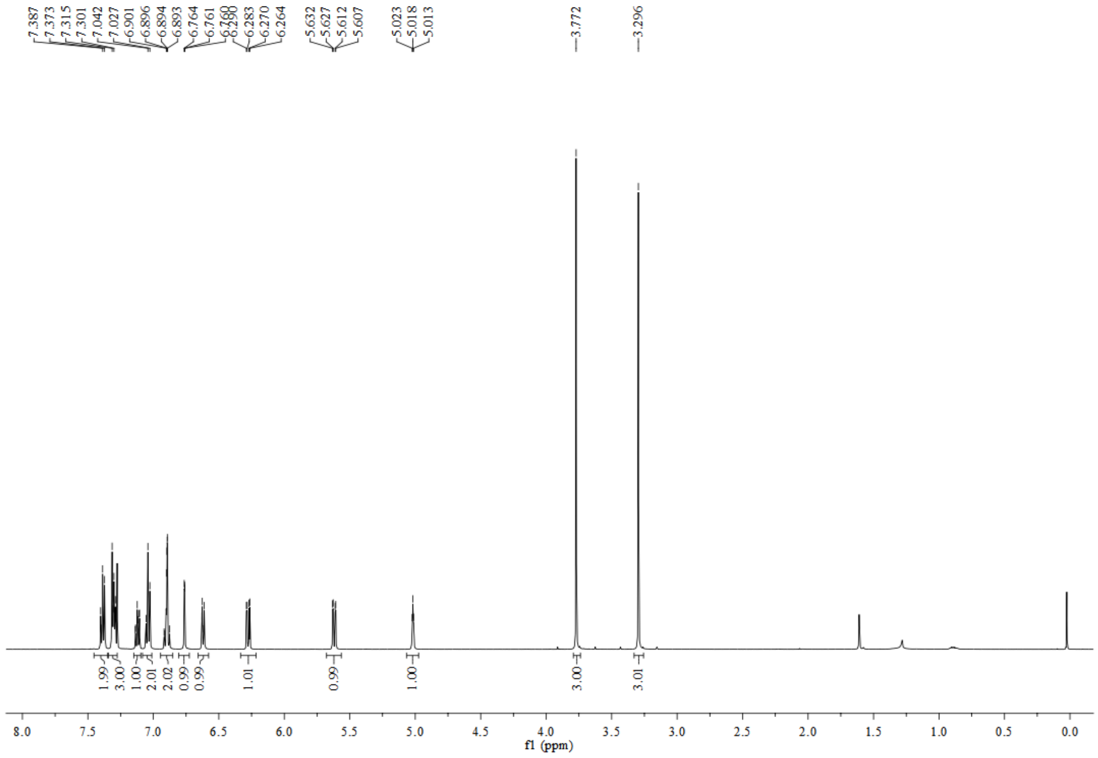

Supplementary Figure 85. ^{1}H NMR Spectra of compound **3g**.

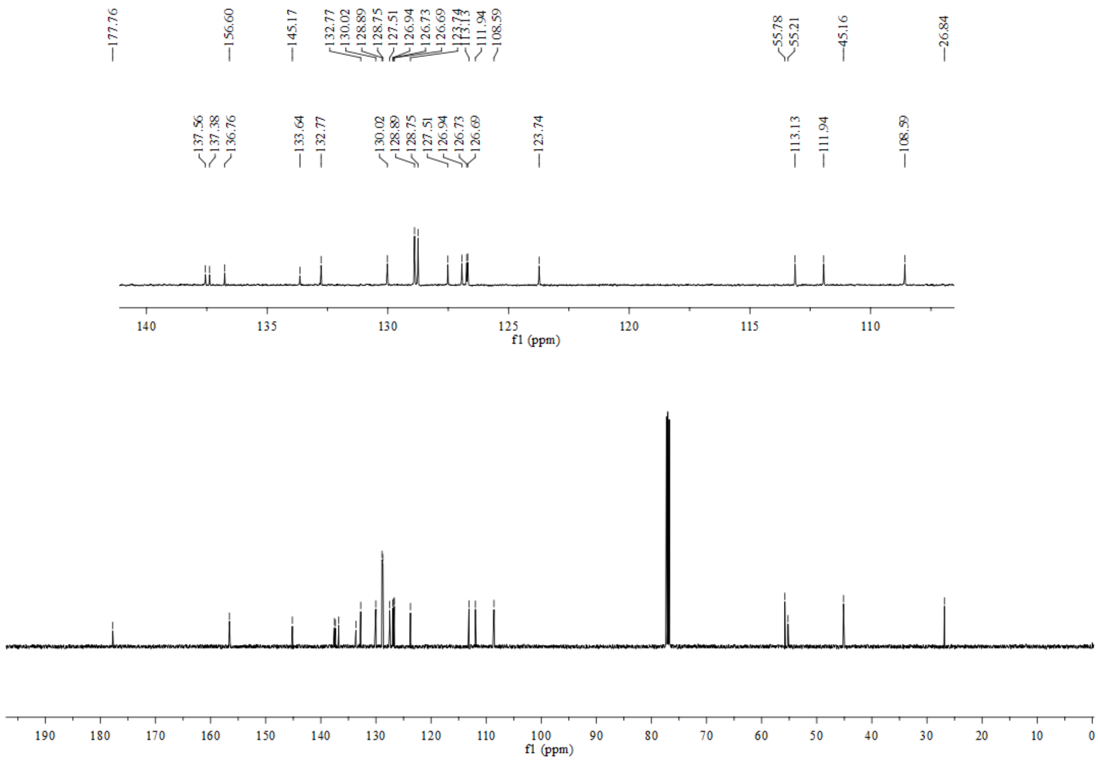

Supplementary Figure 86. ^{13}C NMR Spectra of compound **3g**.

1-Methyl-4'-phenyl-5-(trifluoromethoxy)-4'*H*-spiro[indoline-3,1'-naphthalen]-2-one (**3h**)

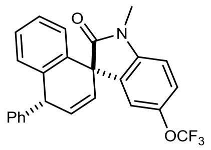

Purified by chromatography on silica gel, eluting with ethyl acetate/petroleum ether 1:15 (*v*/*v*); pale yellow oil, 64.7 mg, 77% yield; ^{1}H NMR (500 MHz, $CDCl_3$): δ 7.42-7.38 (m, 2H), 7.33-7.25 (m, 4H), 7.17-7.13 (m, 1H), 7.08-7.04 (m, 3H), 6.97 (d, *J* = 8.5 Hz, 1H), 6.59-6.57 (m, 1H), 6.31 (dd, *J* = 9.5, 3.0 Hz, 1H), 5.61 (dd, *J* = 10.0, 2.5 Hz, 1H), 5.01 (s, 1H), 3.32 (s, 3H). ^{13}C NMR (125 MHz, $CDCl_3$): δ 177.7, 145.3, 144.7, 142.5, 137.6, 136.8, 133.5, 132.7, 130.1, 128.8, 127.8, 127.0, 126.8, 126.4, 122.9, 121.6, 120.5 (q, *J* = 256.3 Hz), 118.6, 108.6, 55.0, 45.1, 26.9. HRMS *m*/*z* (ESI+): Calculated for $C_{25}H_{19}F_3NO_2$ ($[M+H]^+$): 422.1362, found 422.1360.

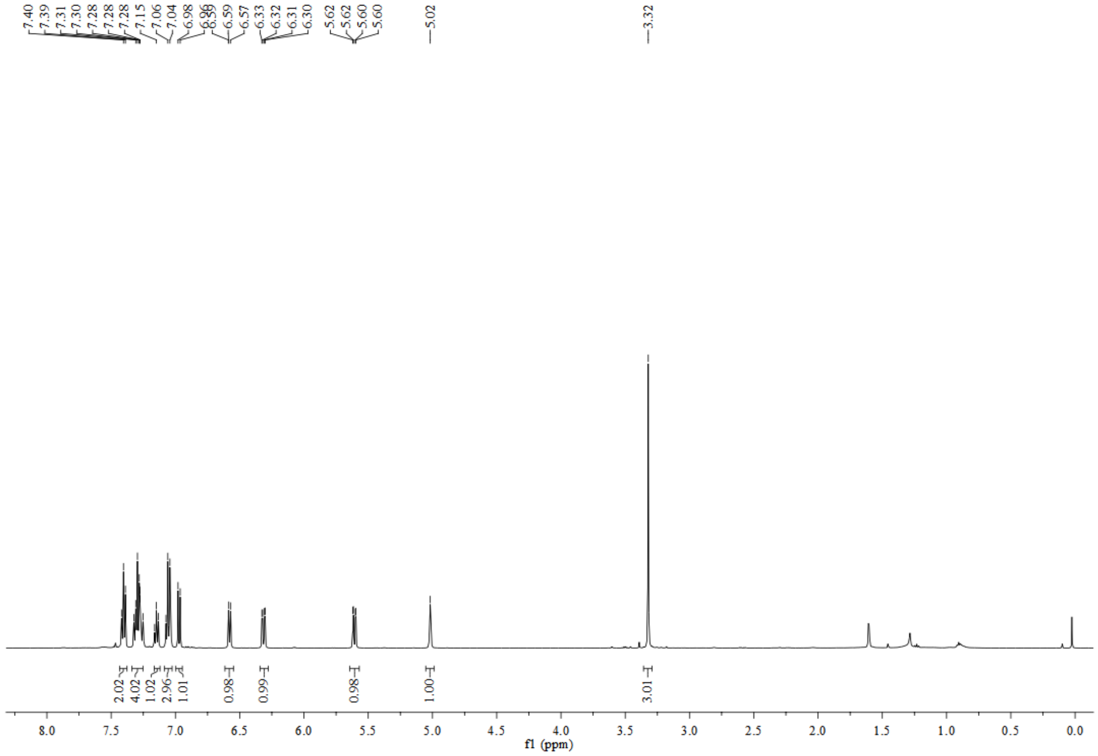

Supplementary Figure 87. ^{1}H NMR Spectra of compound **3h**.

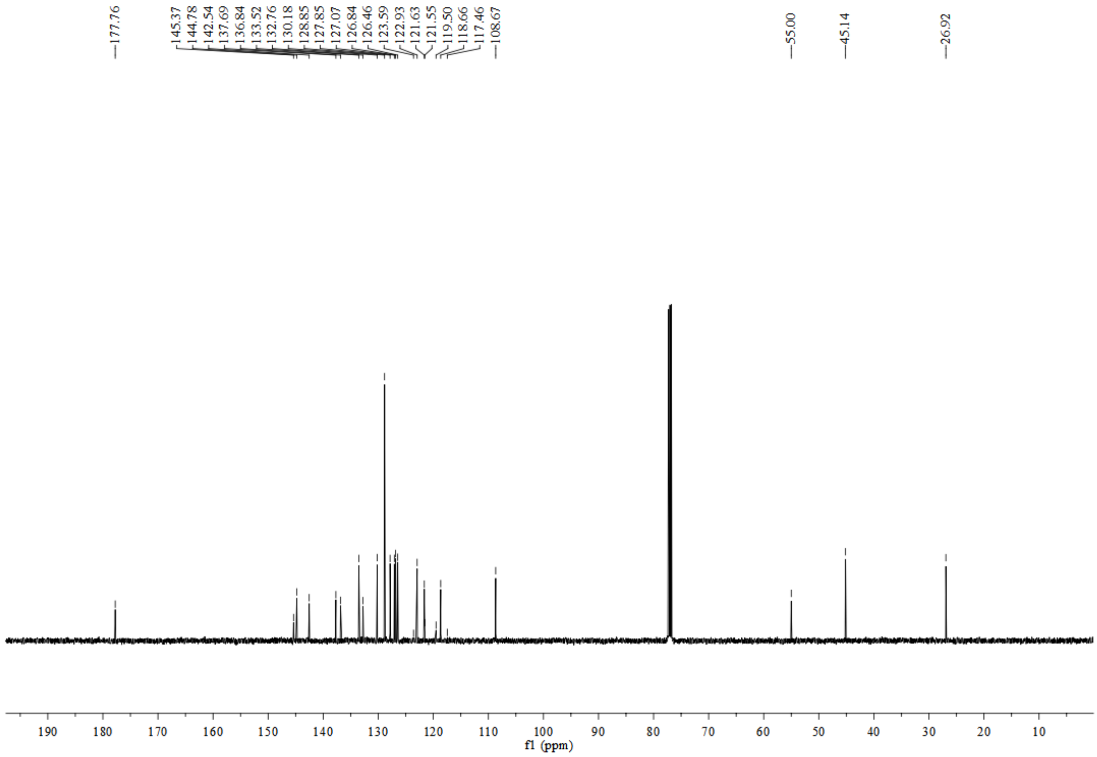

Supplementary Figure 88. ^{13}C NMR Spectra of compound **3h**.

5-Fluoro-1-methyl-4'-phenyl-4'*H*-spiro[indoline-3,1'-naphthalen]-2-one (**3i**)

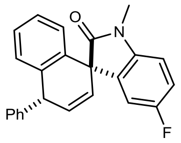

Purified by chromatography on silica gel, eluting with ethyl acetate/petroleum ether 1:15 (*v*/*v*); white solid, Mp = 132-134 ^{o}C, 56.4 mg, 79% yield; ^{1}H NMR (500 MHz, $CDCl_3$): δ 7.42-7.38 (m, *J* = 7.5 Hz, 2H), 7.32-7.28 (m, 3H), 7.16-7.12 (m, 1H), 7.11-7.03 (m, 3H), 6.93-6.89 (m, 2H), 6.59 (d, *J* = 8.0 Hz, 1H), 6.29 (dd, *J* = 10.0, 3.0 Hz, 1H), 5.59 (dd, *J* = 10.0, 2.5 Hz, 1H), 5.01 (s, 1H), 3.31 (s, 3H). ^{13}C NMR (125 MHz, $CDCl_3$): δ 177.7, 159.7 (d, *J* = 240 Hz), 144.8, 139.8 (d, *J* = 1.3 Hz), 137.5, 137.0 (d, *J* = 8.8 Hz), 133.1, 132.9, 130.1, 128.84, 128.80, 127.7, 126.9, 126.7, 126.5, 122.9, 114.9 (d, *J* = 22.5 Hz), 112.8 (d, *J* = 23.8 Hz), 108.7 (d, *J* = 7.5 Hz), 55.0, 45.0, 26.8. HRMS *m*/*z* (ESI+): Calculated for $C_{24}H_{19}FNO$ ([M+H]$^{+}$): 356.1445, found 356.1444.

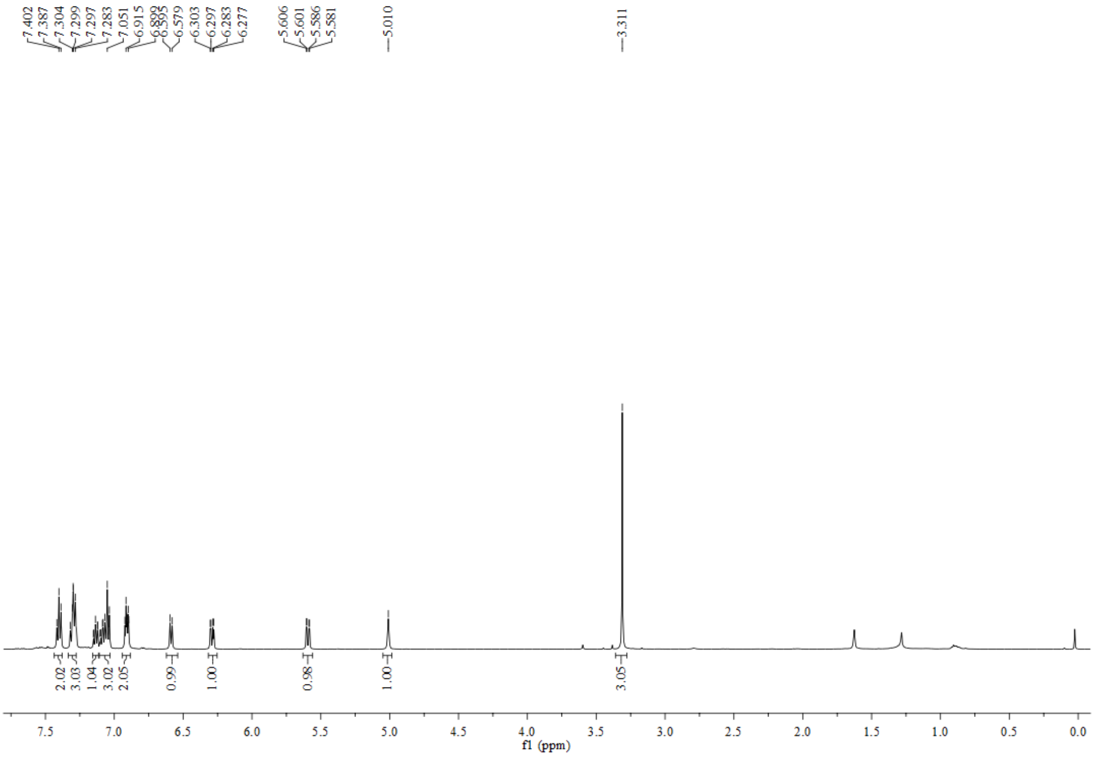

Supplementary Figure 89. ^{1}H NMR Spectra of compound **3i**.

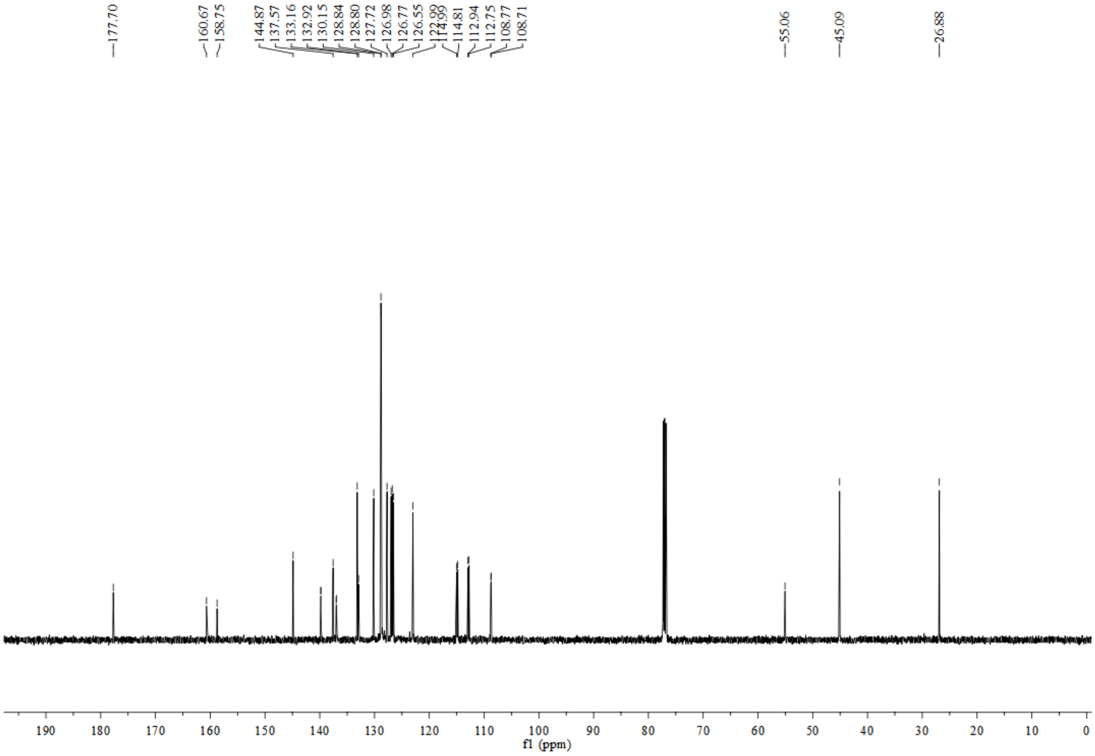

Supplementary Figure 90. ^{13}C NMR Spectra of compound **3i**.

5-Chloro-1-methyl-4'-phenyl-4'*H*-spiro[indoline-3,1'-naphthalen]-2-one (**3j**)

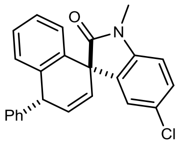

Purified by chromatography on silica gel, eluting with ethyl acetate/petroleum ether 1:15 (*v*/*v*); white solid, Mp = 88-90 °C, 51.4 mg, 69% yield; ^{1}H NMR (500 MHz, $CDCl_3$): δ 7.43-7.39 (m, 2H), 7.37-7.34 (m, 1H), 7.33-7.27 (m, 3H), 7.16-7.12 (m, 2H), 7.08-7.03 (m, 2H), 6.91 (d, *J* = 8.5 Hz, 1H), 6.58 (d, *J* = 8.0 Hz, 1H), 6.29 (dd, *J* = 9.5, 3.0 Hz, 1H), 5.59 (dd, *J* = 10.0, 2.5 Hz, 1H), 5.00 (s, 1H), 3.30 (s, 3H). ^{13}C NMR (125 MHz, $CDCl_3$): δ 177.5, 144.8, 142.5, 137.6, 137.0, 133.3, 132.8, 130.1, 128.89, 128.83, 128.65, 128.61, 127.7, 127.0, 126.8, 126.5, 125.3, 122.9, 109.1, 54.8, 45.1, 26.8. HRMS *m*/*z* (ESI+): Calculated for $C_{24}H_{19}{}^{35}ClNO$ ([M+H]$^+$): 372.1150, found 372.1146.

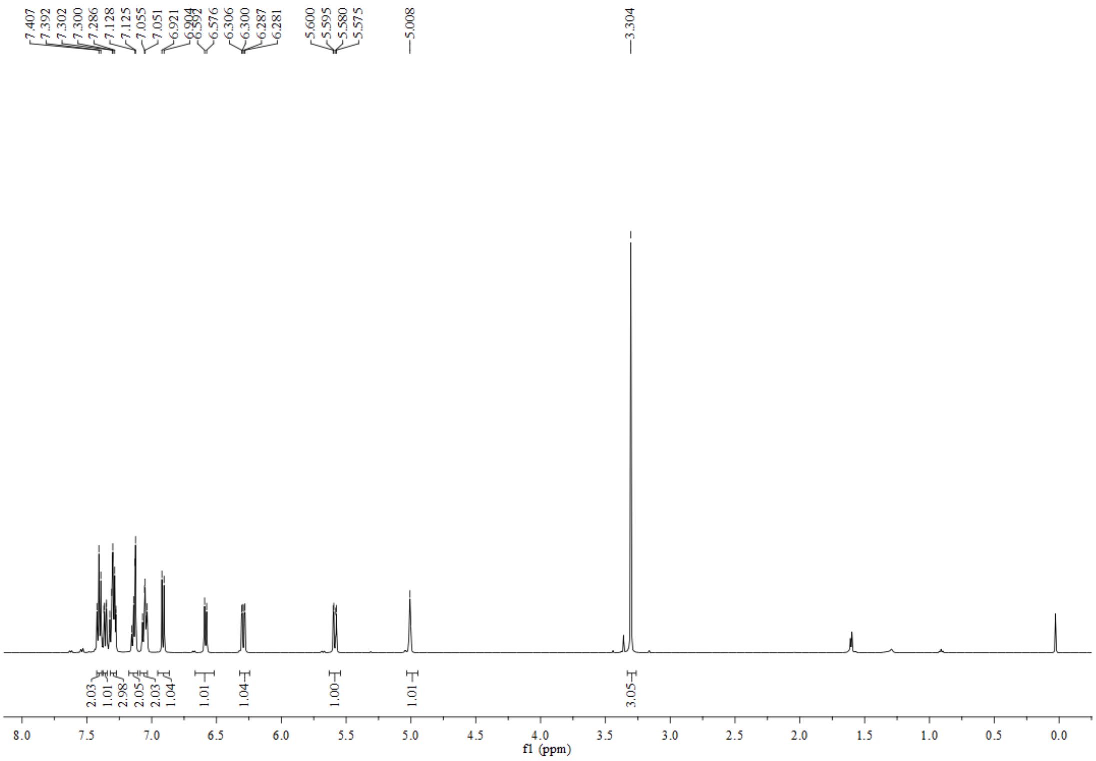

Supplementary Figure 91. ^{1}H NMR Spectra of compound **3j**.

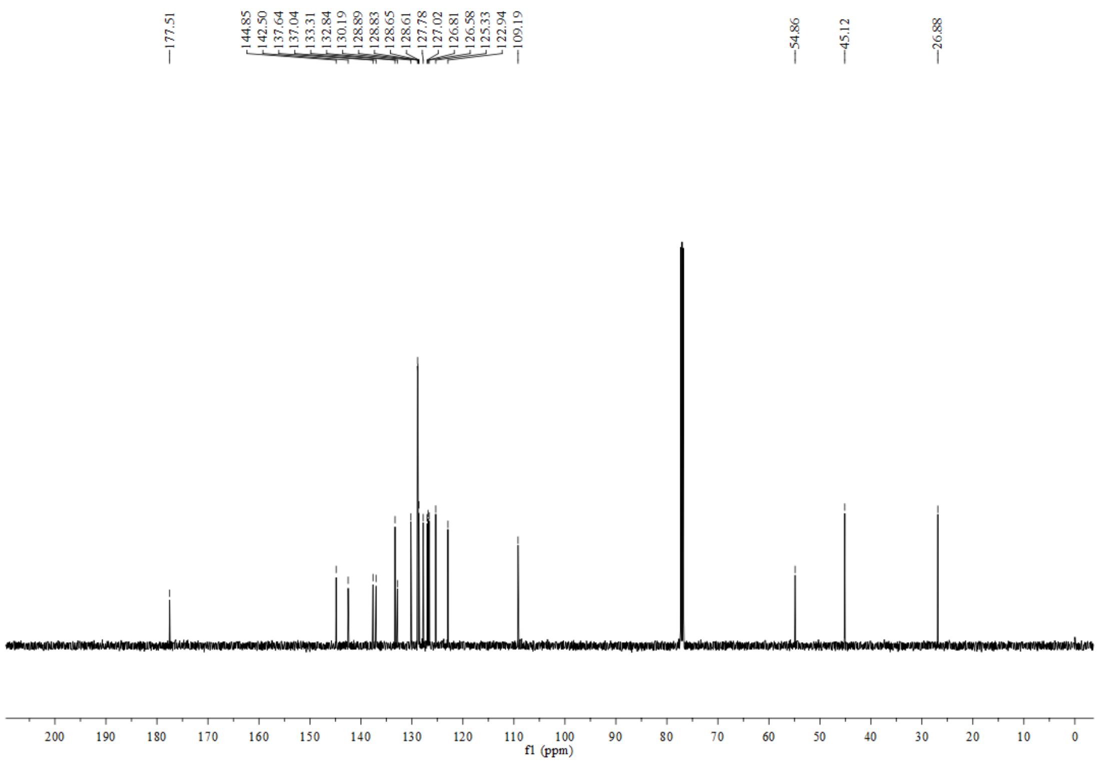

Supplementary Figure 92. ^{13}C NMR Spectra of compound **3j**.

1-Methyl-4'-phenyl-5-(trifluoromethyl)-4'*H*-spiro[indoline-3,1'-naphthalen]-2-one (**3k**)

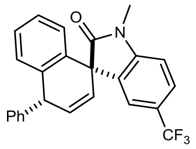

Purified by chromatography on silica gel, eluting with ethyl acetate/petroleum ether 1:15 (*v*/*v*); white solid, Mp = 157-159 °C, 70.2 mg, 87% yield; ^{1}H NMR (600 MHz, $CDCl_3$): δ 7.68 (d, *J* = 8.4 Hz, 1H), 7.43-7.39 (m, 3H), 7.34-7.29 (m, 3H), 7.18-7.15 (m, 1H), 7.08-7.06 (m, 3H), 6.57-6.55 (m, 1H), 6.34 (dd, *J* = 10.2, 3.0 Hz, 1H), 5.60 (dd, *J* = 9.6, 2.4 Hz, 1H), 5.03 (s, 1H), 3.35 (s, 3H). ^{13}C NMR (150 MHz, $CDCl_3$): δ 177.8, 146.9, 144.7, 137.7, 135.9, 133.7, 132.6, 130.2, 128.8, 127.9, 127.1, 126.8, 126.5 (q, *J* = 4.5 Hz), 126.4, 125.7 (q, *J* = 33.0 Hz), 124.3 (q, *J* = 270.0 Hz), 122.8, 121.9 (q, *J* = 3.0 Hz), 108.1, 54.6, 45.1, 26.9. HRMS *m*/*z* (ESI+): Calculated for $C_{25}H_{19}F_3NO$ ($[M+H]^+$): 406.1413, found 406.1414.

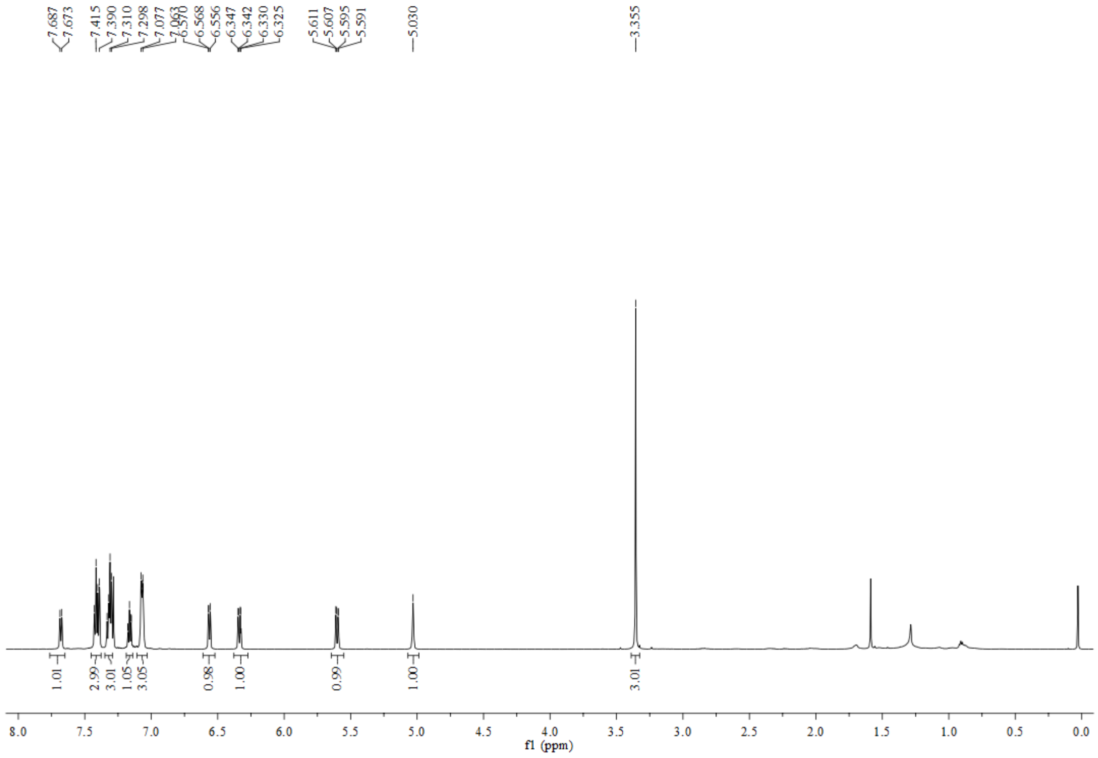

Supplementary Figure 93. ^{1}H NMR Spectra of compound **3k**.

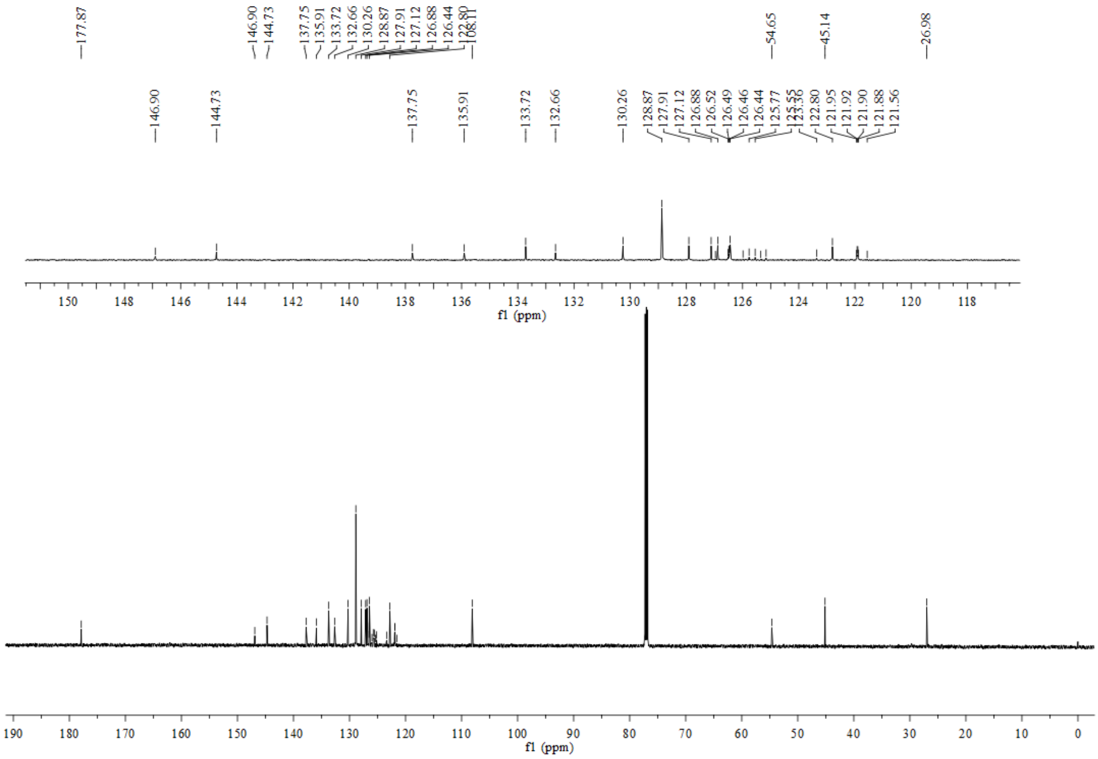

Supplementary Figure 94. ^{13}C NMR Spectra of compound **3k**.

5-Acetyl-1-methyl-4'-phenyl-4'*H*-spiro[indoline-3,1'-naphthalen]-2-one (**3l**)

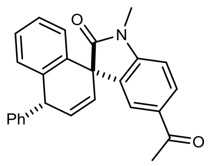

Purified by chromatography on silica gel, eluting with ethyl acetate/petroleum ether 1:15 (*v*/*v*); white solid, Mp = 171-173 °C, 48.6 mg, 64% yield; ^{1}H NMR (600 MHz, $CDCl_3$): δ 7.65 (s, 1H), 7.62 (d, *J* = 8.4 Hz, 1H), 7.43-7.40 (m, 3H), 7.32 (d, *J* = 8.4 Hz, 3H), 7.15-7.13 (m, 2H), 7.02 (d, *J* = 7.8 Hz, 1H), 6.71 (d, *J* = 8.4 Hz, 1H), 6.29 (dd, *J* = 9.6, 3.0 Hz, 1H), 5.62 (dd, *J* = 10.2, 2.4 Hz, 1H), 5.06 (s, 1H), 3.33 (s, 3H), 2.43 (s, 3H). ^{13}C NMR (150 MHz, $CDCl_3$): δ 197.5, 177.3, 144.4, 143.8, 138.7, 138.2, 136.3, 134.9, 132.5, 130.3, 129.0, 128.8, 127.2, 127.0, 126.5, 124.7, 123.5, 123.2, 108.5, 54.9, 45.1, 26.8, 26.5. HRMS *m*/*z* (ESI+): Calculated for $C_{26}H_{22}NO_2$ ($[M+H]^+$): 380.1645, found 380.1641.

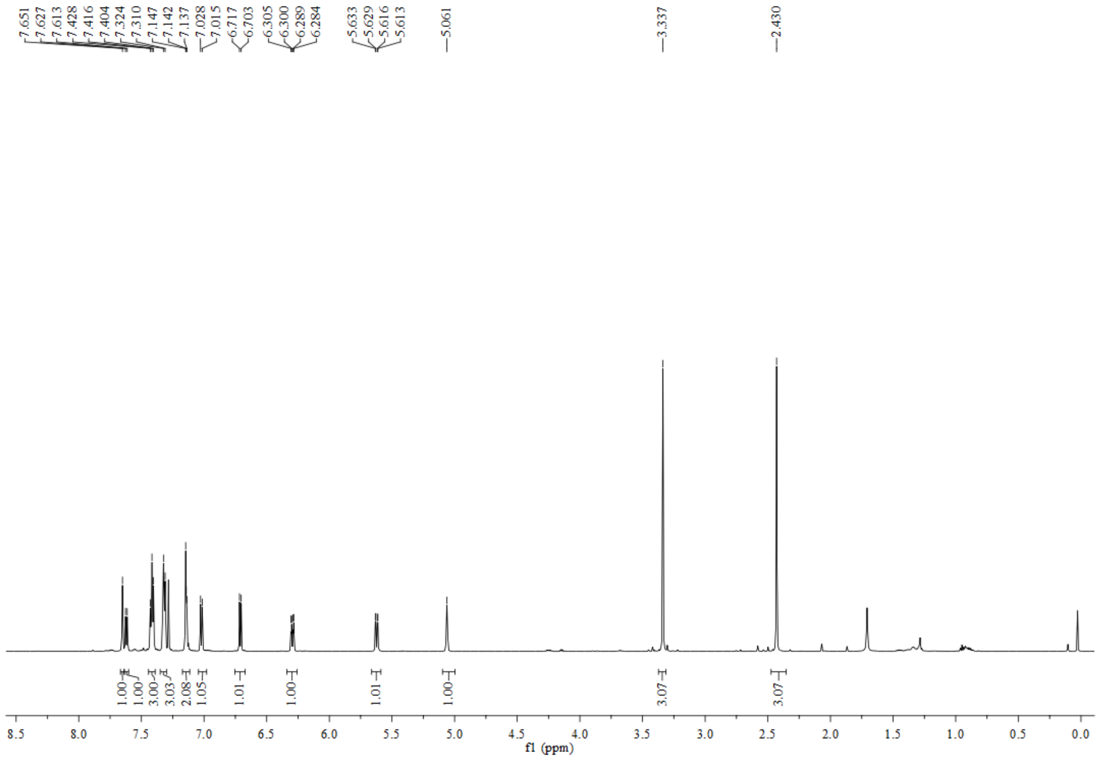

Supplementary Figure 95. ^{1}H NMR Spectra of compound **3l**.

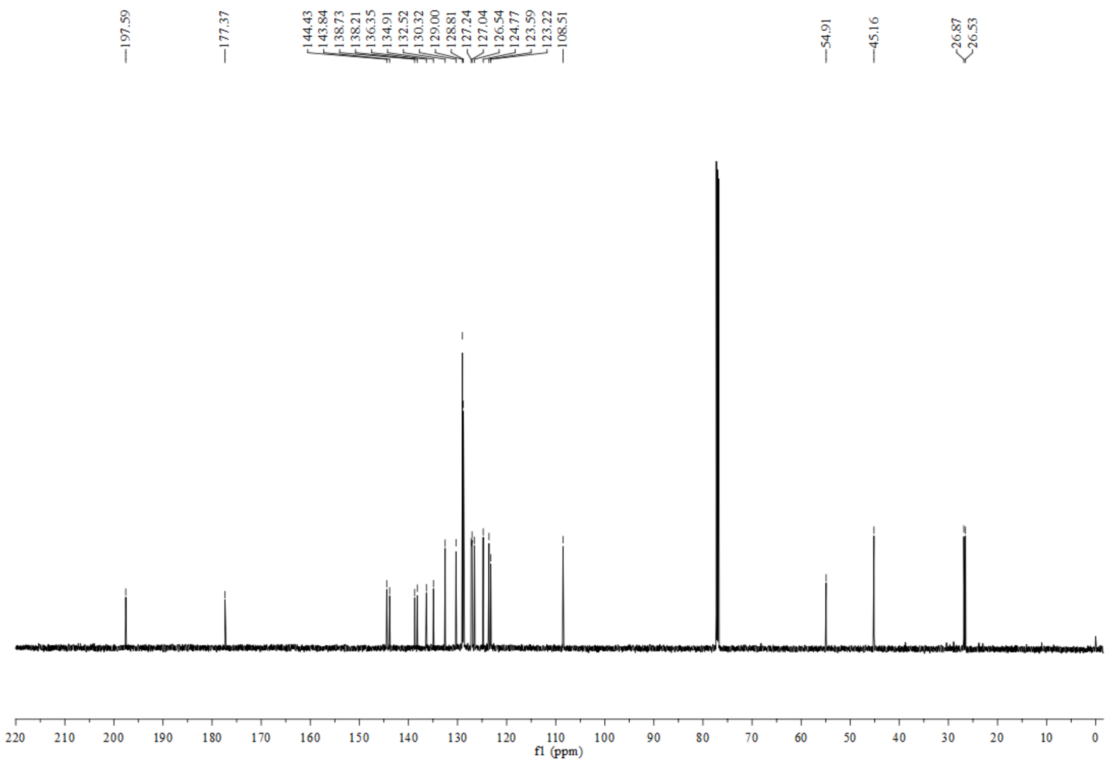

Supplementary Figure 96. ^{13}C NMR Spectra of compound **3l**.

1,6-Dimethyl-4'-phenyl-4'*H*-spiro[indoline-3,1'-naphthalen]-2-one (**3m**)

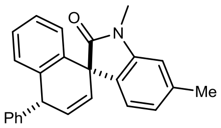

Purified by chromatography on silica gel, eluting with ethyl acetate/petroleum ether 1:15 (*v*/*v*); white solid, Mp = 207-209 °C, 58.5 mg, 83% yield; ^{1}H NMR (600 MHz, $CDCl_3$): δ 7.42-7.39 (m, 2H), 7.34-7.29 (m, 3H), 7.14-7.10 (m, 1H), 7.07-7.02 (m, 3H), 6.95 (d, *J* = 7.8 Hz, 1H), 6.83 (s, 1H), 6.64-6.61 (m, 1H), 6.26 (dd, *J* = 10.2, 3.6 Hz, 1H), 5.61 (dd, *J* = 9.6, 2.4 Hz, 1H), 5.02 (t, *J* = 3.0 Hz, 1H), 3.31 (s, 3H), 2.47 (s, 3H). ^{13}C NMR (150 MHz, $CDCl_3$): δ 178.3, 145.2, 143.9, 138.8, 137.6, 133.7, 132.7, 132.5, 130.0, 128.9, 128.7, 127.4, 126.8, 126.7, 126.6, 124.5, 123.89, 123.88, 109.1, 54.5, 45.1, 26.7, 21.9. HRMS *m*/*z* (ESI+): Calculated for $C_{25}H_{22}NO$ ($[M+H]^+$): 352.1696, found 352.1698.

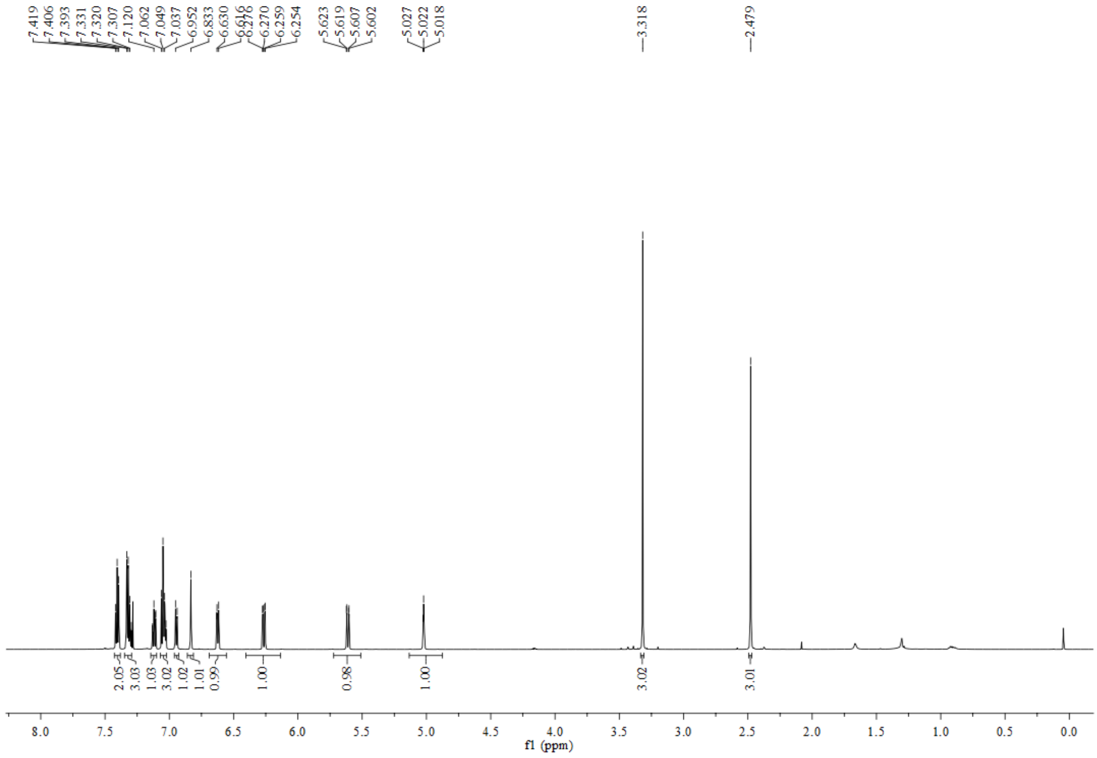

Supplementary Figure 97. ^{1}H NMR Spectra of compound **3m**.

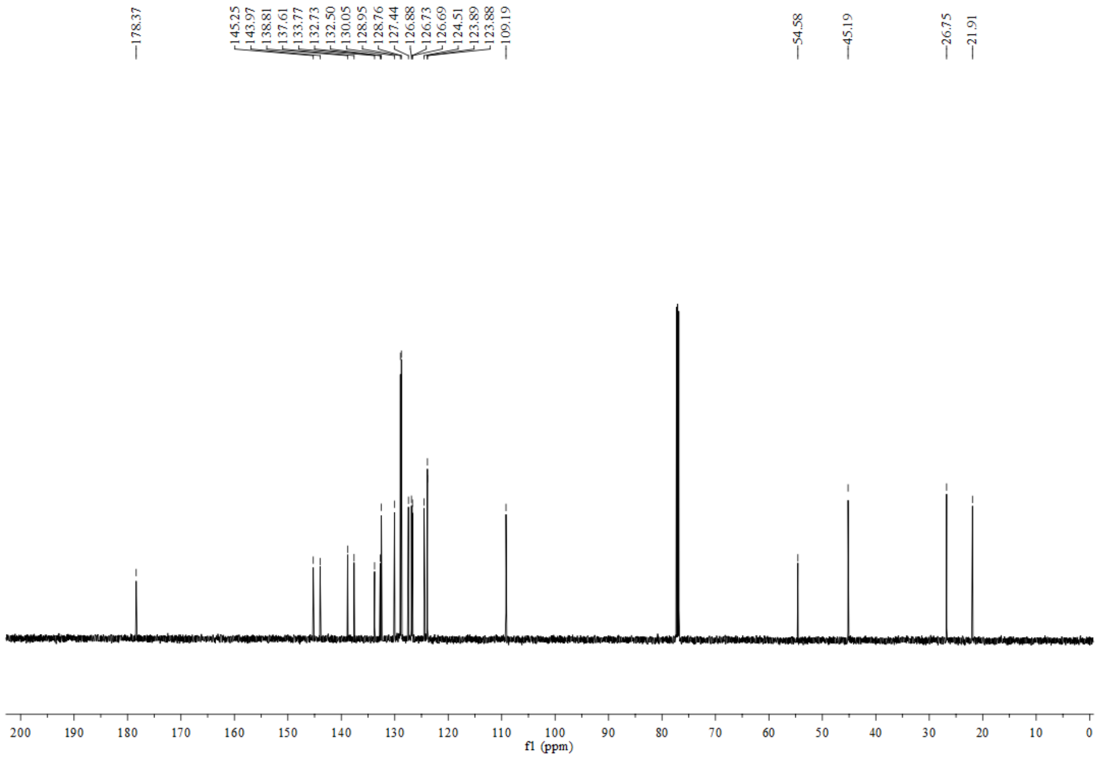

Supplementary Figure 98. ^{13}C NMR Spectra of compound **3m**.

6-Methoxy-1-methyl-4'-phenyl-4'*H*-spiro[indoline-3,1'-naphthalen]-2-one (**3n**)

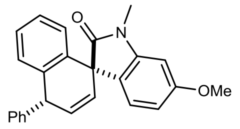

Purified by chromatography on silica gel, eluting with ethyl acetate/petroleum ether 1:15 (*v*/*v*); white solid, Mp = 162-164 °C, 59.3 mg, 81% yield; ^{1}H NMR (500 MHz, $CDCl_3$): δ 7.41-7.37 (m, 2H), 7.30 (d, *J* = 7.5 Hz, 3H), 7.11 (td, *J* = 7.5, 1.0 Hz, 1H), 7.06-7.01 (m, 3H), 6.63-6.56 (m, 3H), 6.24 (dd, *J* = 9.5, 3.0 Hz, 1H), 5.59 (dd, *J* = 9.5, 2.5 Hz, 1H), 5.00 (t, *J* = 2.5 Hz, 1H), 3.88 (s, 3H), 3.29 (s, 3H). ^{13}C NMR (150 MHz, $CDCl_3$): δ 178.5, 160.5, 145.2, 145.1, 137.6, 133.9, 132.4, 130.0, 128.9, 128.7, 127.6, 127.4, 126.8, 126.7, 126.6, 125.3, 124.0, 107.1, 96.2, 55.6, 54.2, 45.1, 26.8. HRMS *m*/*z* (ESI+): Calculated for $C_{25}H_{22}NO_2$ ($[M+H]^+$): 368.1645, found 368.1643.

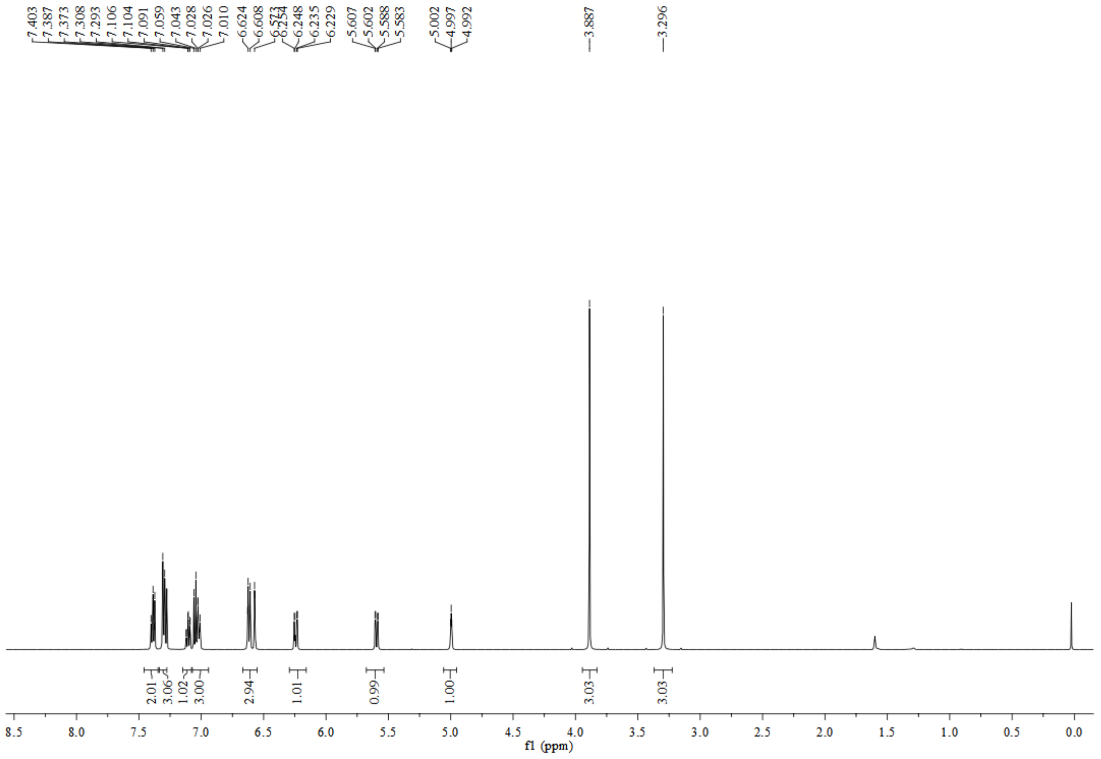

Supplementary Figure 99. ^{1}H NMR Spectra of compound **3n**.

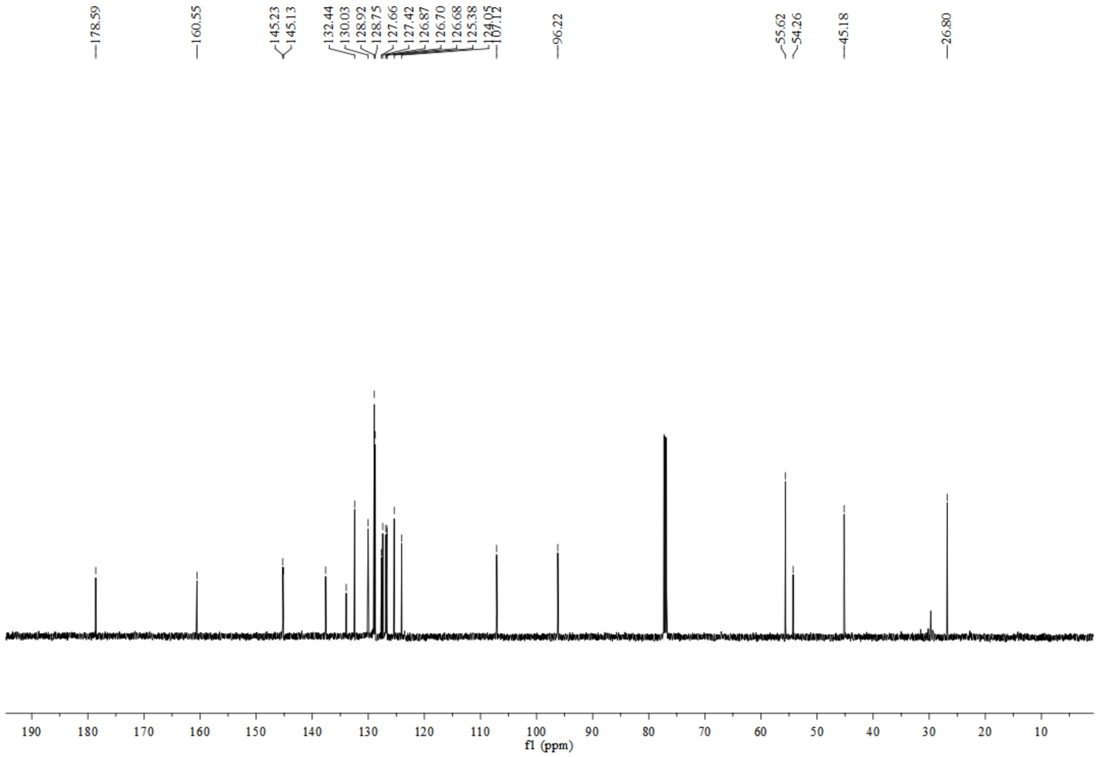

Supplementary Figure 100. ^{13}C NMR Spectra of compound **3n**.

1-Methyl-4'-phenyl-6-(trifluoromethoxy)-4'*H*-spiro[indoline-3,1'-naphthalen]-2-one (**3o**)

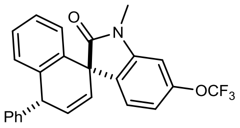

Purified by chromatography on silica gel, eluting with ethyl acetate/petroleum ether 1:15 (*v*/*v*); white solid, Mp = 146-148 ^{o}C, 77.3 mg, 92% yield; ^{1}H NMR (600 MHz, $CDCl_3$): δ 7.42-7.39 (m, 2H), 7.33-7.28 (m, 3H), 7.17-7.13 (m, 2H), 7.08-7.05 (m, 2H), 6.98 (d, *J* = 7.8 Hz, 1H), 6.86 (s, 1H), 6.60 (d, *J* = 7.8 Hz, 1H), 6.31-6.29 (m, 1H), 5.60 (d, *J* = 9.6 Hz, 1H), 5.02 (s, 1H), 3.32 (s, 3H). ^{13}C NMR (150 MHz, $CDCl_3$): δ 178.0, 149.6, 145.2, 144.8, 137.6, 133.7, 133.1, 132.9, 130.1, 128.86, 128.82, 127.7, 127.0, 126.8, 126.5, 125.7, 123.0, 120.5 (q, *J* = 255.0 Hz), 115.3, 102.0, 54.3, 45.1, 26.9. HRMS *m*/*z* (ESI+): Calculated for $C_{25}H_{19}F_3NO_2$ ($[M+H]^+$): 422.1362, found 422.1363.

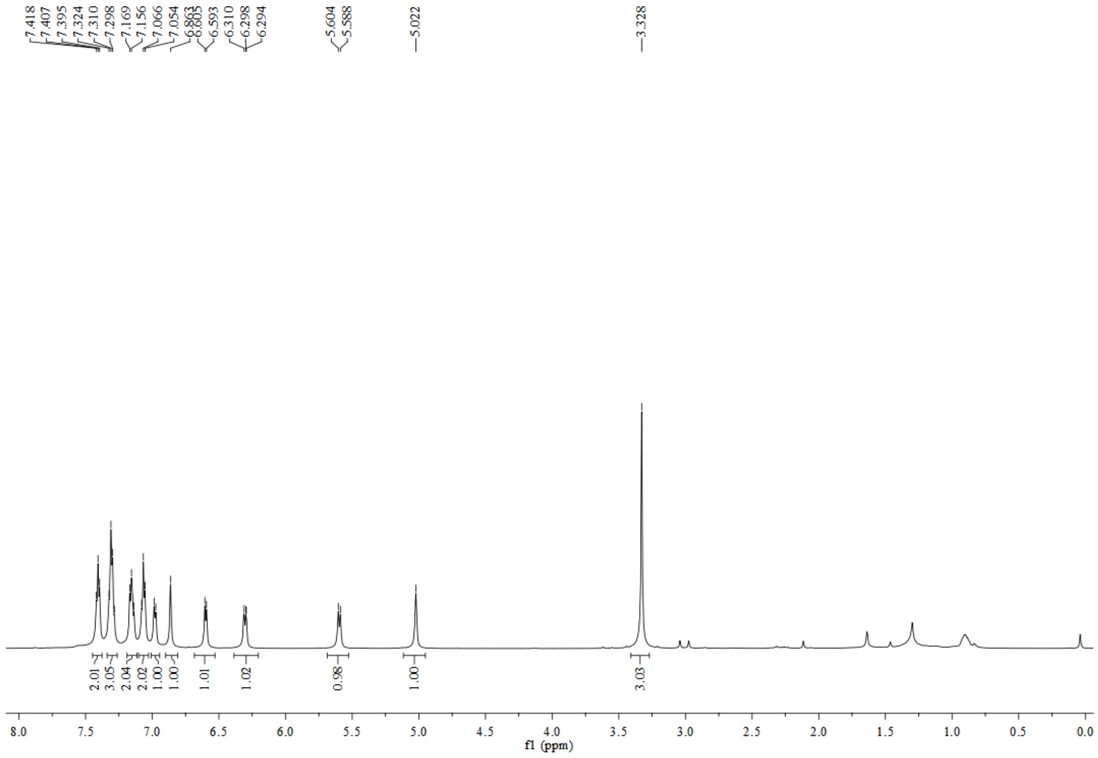

Supplementary Figure 101. ^{1}H NMR Spectra of compound **3o**.

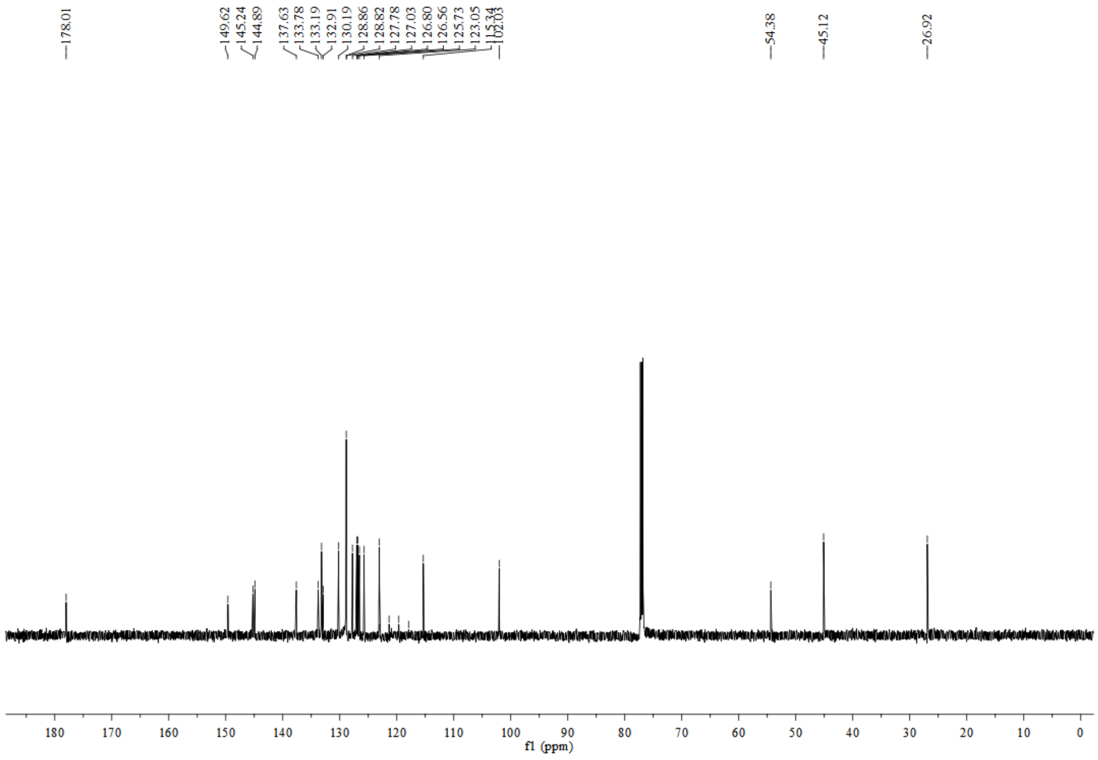

Supplementary Figure 102. ^{13}C NMR Spectra of compound **3o**.

6-Fluoro-1-methyl-4'-phenyl-4'*H*-spiro[indoline-3,1'-naphthalen]-2-one (**3p**)

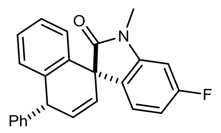

Purified by chromatography on silica gel, eluting with ethyl acetate/petroleum ether 1:15 (*v*/*v*); white solid, Mp = 195-196 °C, 64.3 mg, 90% yield; ^{1}H NMR (500 MHz, $CDCl_3$): δ 7.37-7.34 (m, 2H), 7.33-7.28 (m, 3H), 7.14-7.07 (m, 2H), 7.06-7.02 (m, 2H), 6.82-6.77 (m, 1H), 6.75-6.71 (m, 1H), 6.58 (d, *J* = 7.9 Hz, 1H), 6.27 (dd, *J* = 10.0, 3.5 Hz, 1H), 5.58 (dd, *J* = 10.0, 2.5 Hz, 1H), 5.00 (s, 1H), 3.29 (s, 3H). ^{13}C NMR (150 MHz, $CDCl_3$): δ 178.2, 163.3 (d, *J* = 244.6 Hz), 145.4 (d, *J* = 12.1 Hz), 145.0, 137.6, 133.3, 132.9, 130.8, 130.1, 128.9, 128.8, 127.6, 126.9, 126.7, 126.5, 125.9 (d, *J* = 10.6 Hz), 123.4, 109.4 (d, *J* = 22.7 Hz), 97.1 (d, *J* = 27.2 Hz), 54.3, 45.1, 26.9. HRMS *m*/*z* (ESI+): Calculated for $C_{24}H_{19}FNO$ ($[M+H]^+$): 356.1445, found 356.1445.

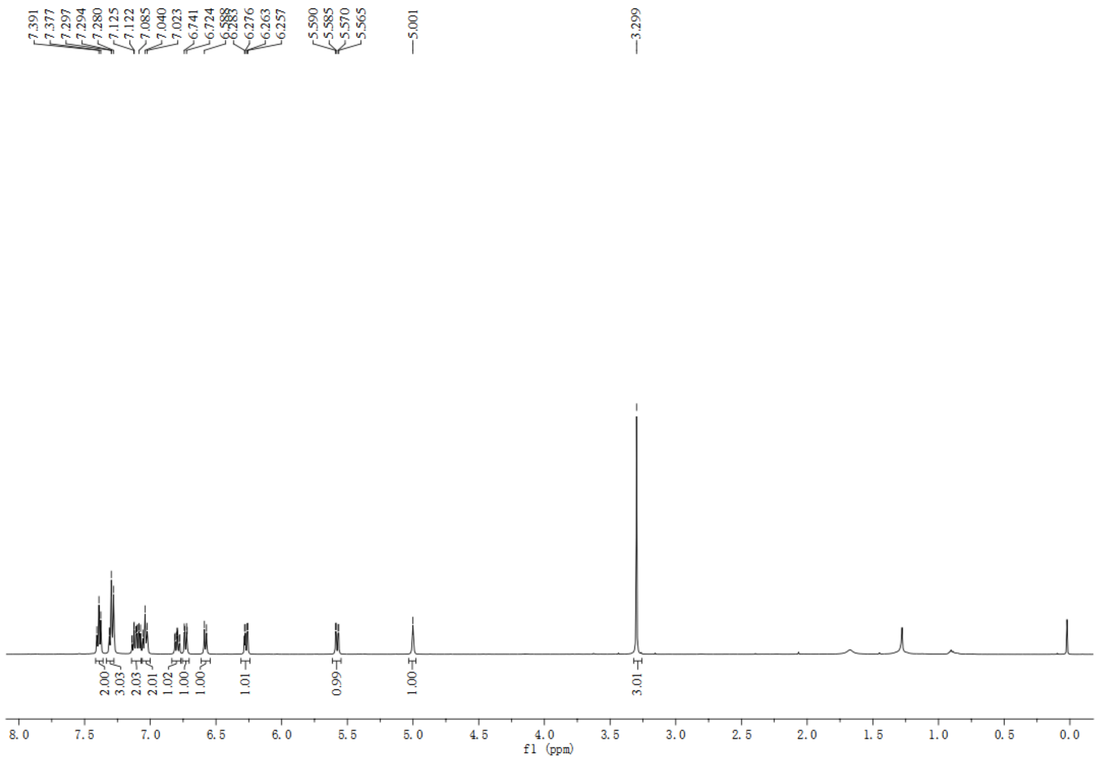

Supplementary Figure 103. ^{1}H NMR Spectra of compound **3p**.

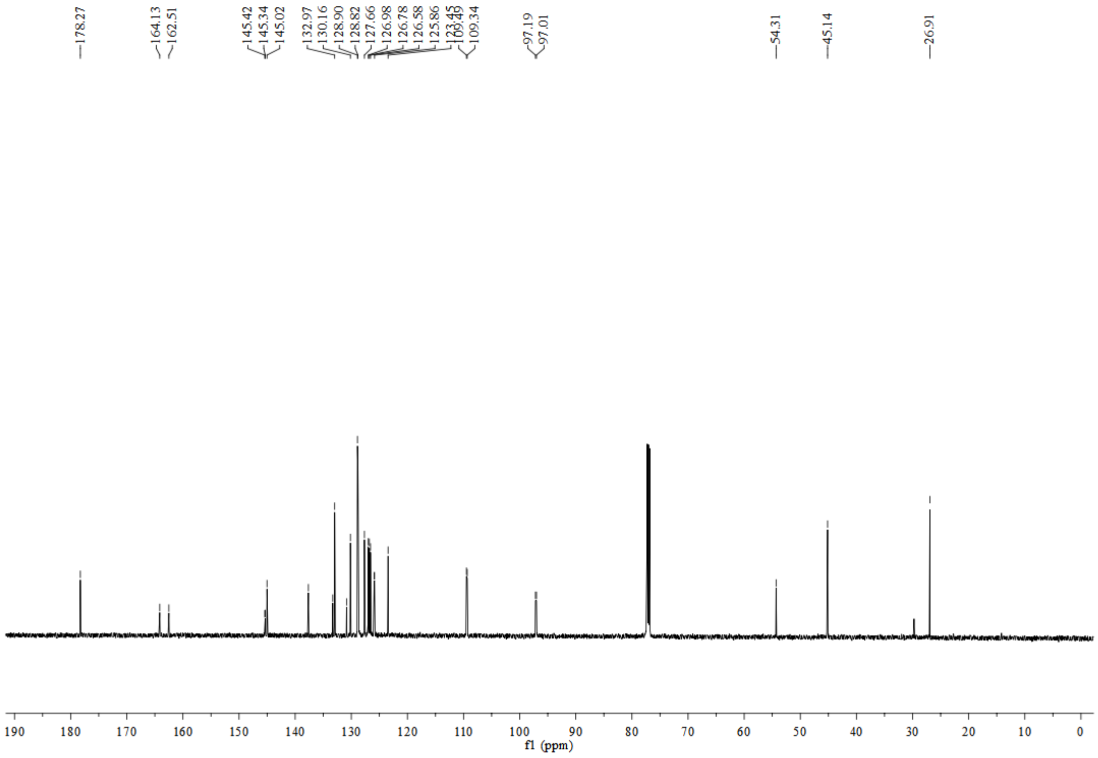

Supplementary Figure 104. ^{13}C NMR Spectra of compound **3p**.

1-Methyl-4'-phenyl-6-(trifluoromethyl)-4'*H*-spiro[indoline-3,1'-naphthalen]-2-one (**3q**)

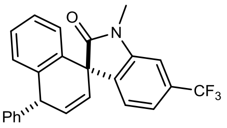

Purified by chromatography on silica gel, eluting with ethyl acetate/petroleum ether 1:15 (*v*/*v*); white solid, Mp = 150-153 °C, 68.2 mg, 84% yield; ^{1}H NMR (500 MHz, $CDCl_3$): δ 7.42-7.38 (m, 3H), 7.33-7.28 (m, 3H), 7.25 (d, *J* = 7.5 Hz, 1H), 7.20 (s, 1H), 7.17-7.12 (m, 1H), 7.07-7.03 (m, 2H), 6.57-6.54 (m, 1H), 6.31 (dd, *J* = 10.0, 3.0 Hz, 1H), 5.58 (dd, *J* = 10.0, 2.5 Hz, 1H), 5.02 (s, 1H), 3.36 (s, 3H). ^{13}C NMR (125 MHz, $CDCl_3$): δ 177.6, 144.8, 144.5, 139.1, 137.6, 133.4, 132.5, 131.2 (q, *J* = 32.5 Hz), 130.2, 128.87, 128.84, 127.9, 127.0, 126.8, 126.5, 125.0, 124.0 (q, *J* = 271.3 Hz), 122.6, 120.4 (q, *J* = 3.8 Hz), 105.1 (q, *J* = 2.5 Hz), 54.6, 45.1, 26.9. HRMS *m*/*z* (ESI+): Calculated for $C_{25}H_{18}F_3NNaO$ ([M+Na]$^+$): 428.1233, found 428.1232.

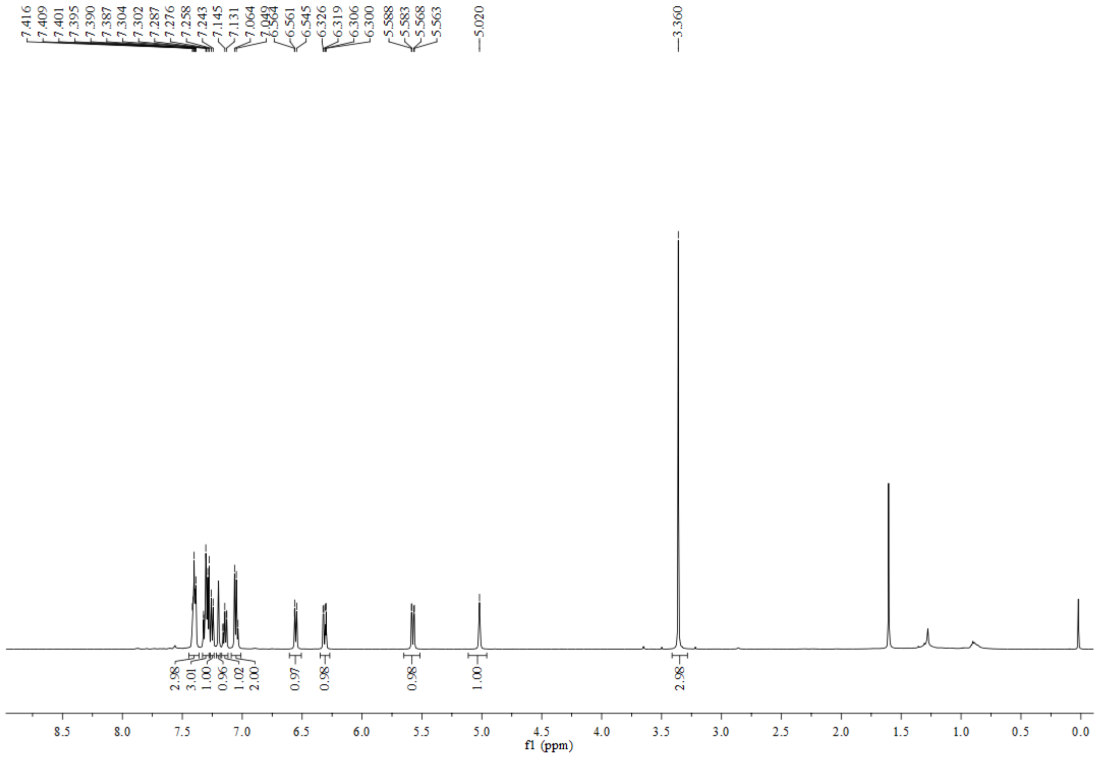

Supplementary Figure 105. ^{1}H NMR Spectra of compound **3q**.

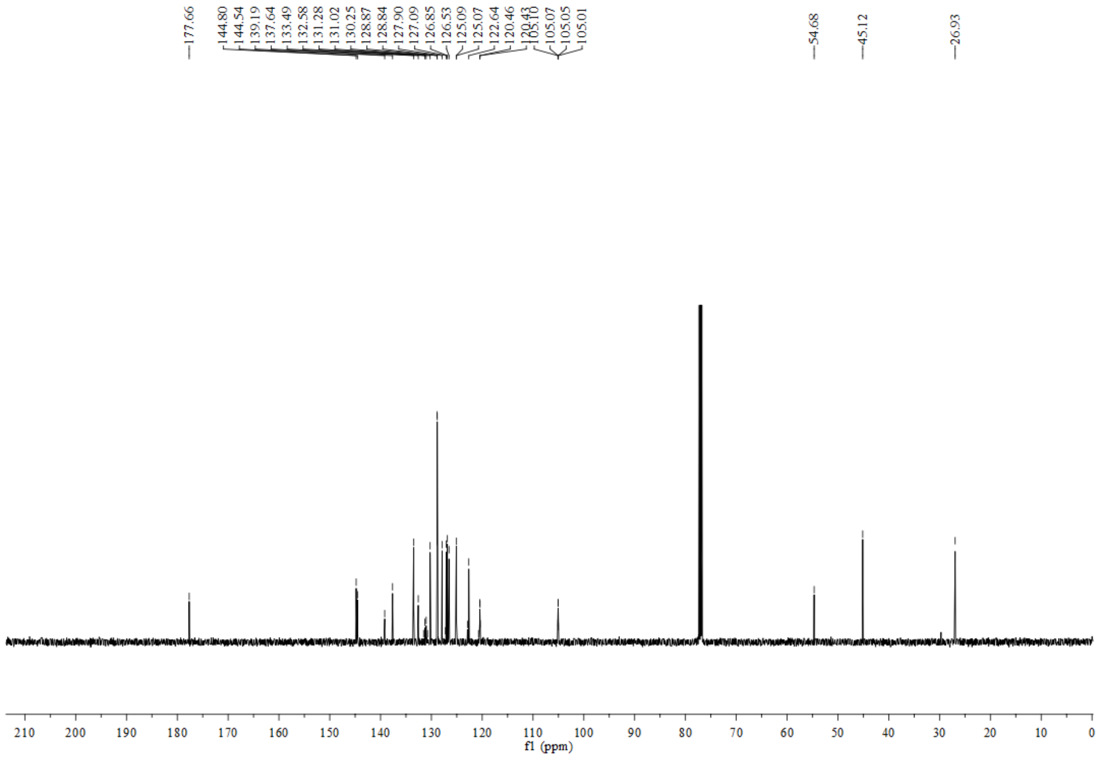

Supplementary Figure 106. ^{13}C NMR Spectra of compound **3q**.

Methyl 1-methyl-2-oxo-4'-phenyl-4'*H*-spiro[indoline-3,1'-naphthalene]-6-carboxylate (**3r**)

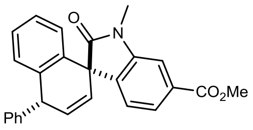

Purified by chromatography on silica gel, eluting with ethyl acetate/petroleum ether 1:15 (*v*/*v*); white solid, Mp = 178-180 °C, 63.0 mg, 80% yield; ^{1}H NMR (500 MHz, $CDCl_3$): δ 7.86-7.83 (m, 1H), 7.65 (d, *J* = 1.0 Hz, 1H), 7.42-7.38 (m, 2H), 7.32-7.29 (m, 3H), 7.22 (d, *J* = 8.0 Hz, 1H), 7.15-7.11 (m, 1H), 7.06-7.01 (m, 2H), 6.60-6.56-6.53 (m, 1H), 6.30 (dd, *J* = 10.0, 3.0 Hz, 1H), 5.58 (dd, *J* = 10.0, 2.5 Hz, 1H), 5.02 (t, *J* = 2.5 Hz, 1H), 3.98 (s, 3H), 3.36 (s, 3H). ^{13}C NMR (125 MHz, $CDCl_3$): δ 177.6, 166.6, 144.8, 144.2, 140.4, 137.5, 133.2, 132.7, 130.7, 130.1, 128.8, 128.7, 127.7, 126.9, 126.7, 126.5, 125.1, 124.6, 122.7, 108.9, 54.7, 52.3, 45.0, 26.9. HRMS *m*/*z* (ESI+): Calculated for $C_{26}H_{22}NO_3$ ([M+H]$^+$): 396.1594, found 396.1591.

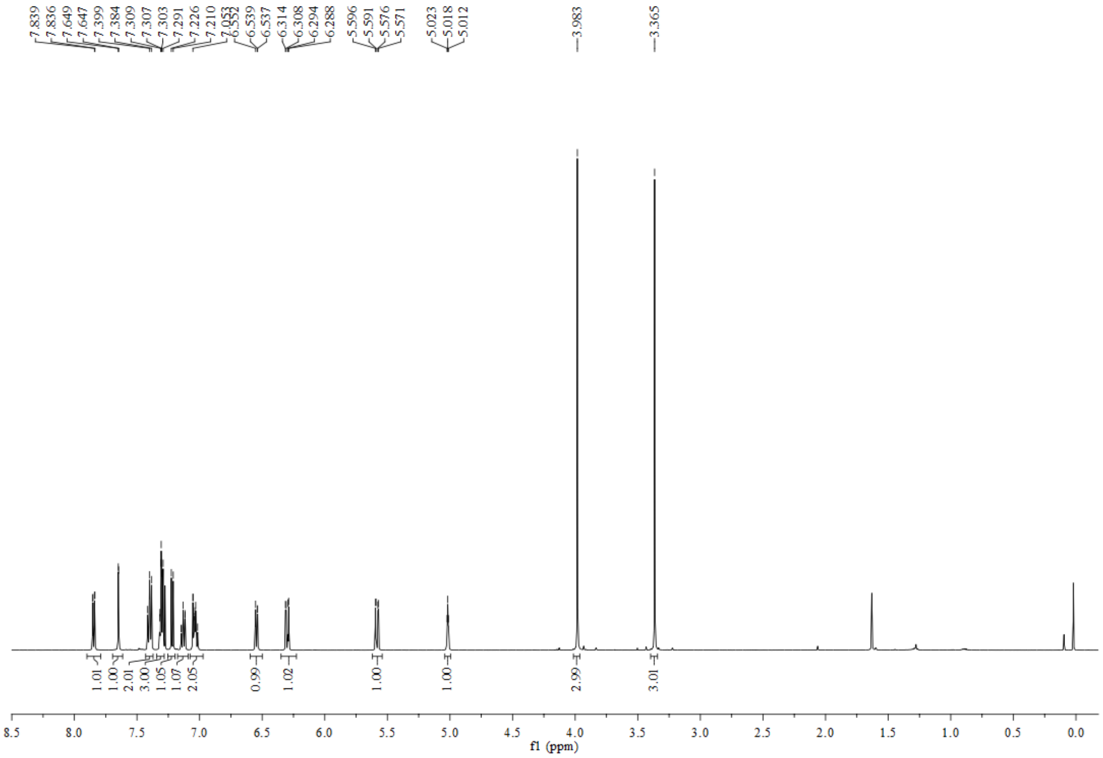

Supplementary Figure 107. ^{1}H NMR Spectra of compound **3r**.

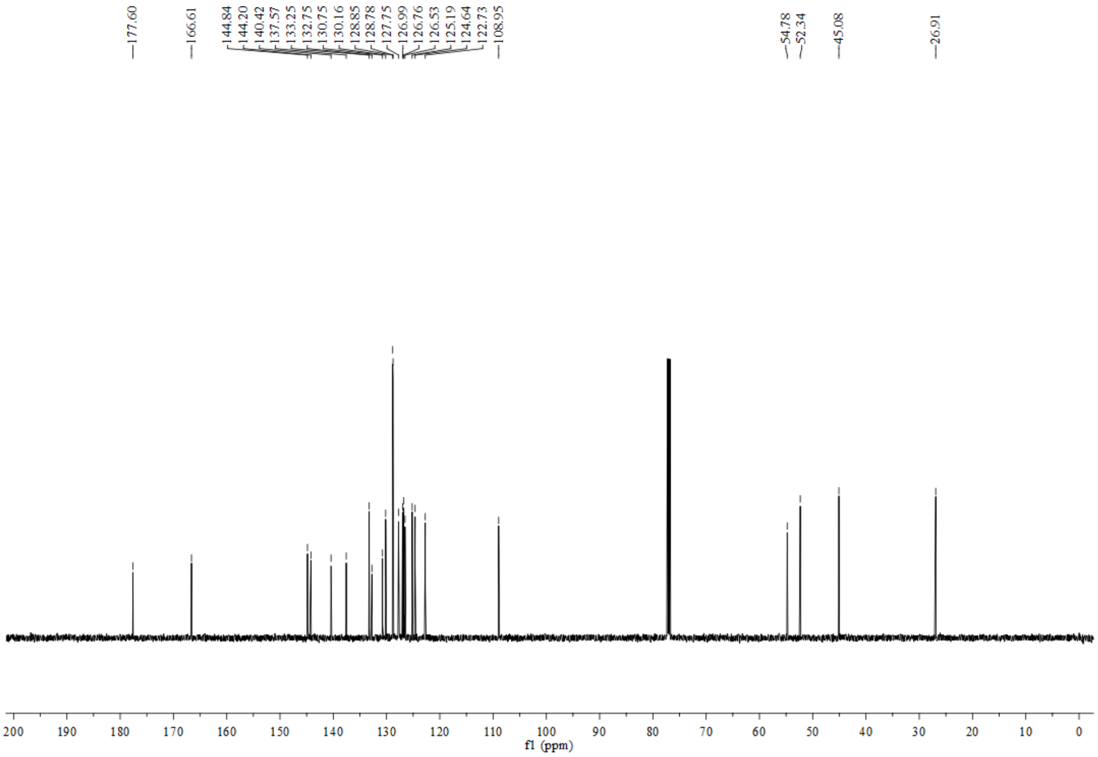

Supplementary Figure 108. ^{13}C NMR Spectra of compound **3r**.

N,*N*-diethyl-1-methyl-2-oxo-4'-phenyl-4'*H*-spiro[indoline-3,1'-naphthalene]-6-carbox-amide (**3s**)

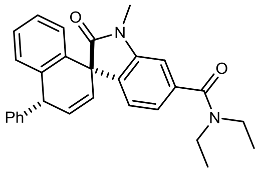

Purified by chromatography on silica gel, eluting with ethyl acetate/petroleum ether 1:10 (*v*/*v*); white solid, Mp = 140-142 °C, 78.5 mg, 90% yield; ^{1}H NMR (600 MHz, $CDCl_3$): δ 7.41-7.38 (m, 2H), 7.31-7.29 (m, 3H), 7.16-7.09 (m, 3H), 7.06-7.03 (m, 3H), 6.60 (d, *J* = 7.8 Hz, 1H), 6.28 (dd, *J* = 10.2, 3.6 Hz, 1H), 5.59 (dd, *J* = 10.2, 2.4 Hz, 1H), 5.01 (s, 1H), 3.60 (s, 2H), 3.37 (s, 2H), 3.32 (s, 3H), 1.30 (s, 3H), 1.20 (s, 3H). ^{13}C NMR (150 MHz, $CDCl_3$): δ 177.9, 170.7, 144.9, 144.2, 137.7, 137.5, 136.5, 133.0, 132.9, 130.1, 128.8, 128.7, 127.7, 126.9, 126.77, 126.74, 124.5, 123.1, 121.0, 106.8, 54.6, 45.1, 43.4, 39.4, 26.8. 14.3, 12.8. HRMS *m*/*z* (ESI+): Calculated for $C_{29}H_{29}N_2O_2$ ($[M+H]^+$): 437.2224, found 437.2226.

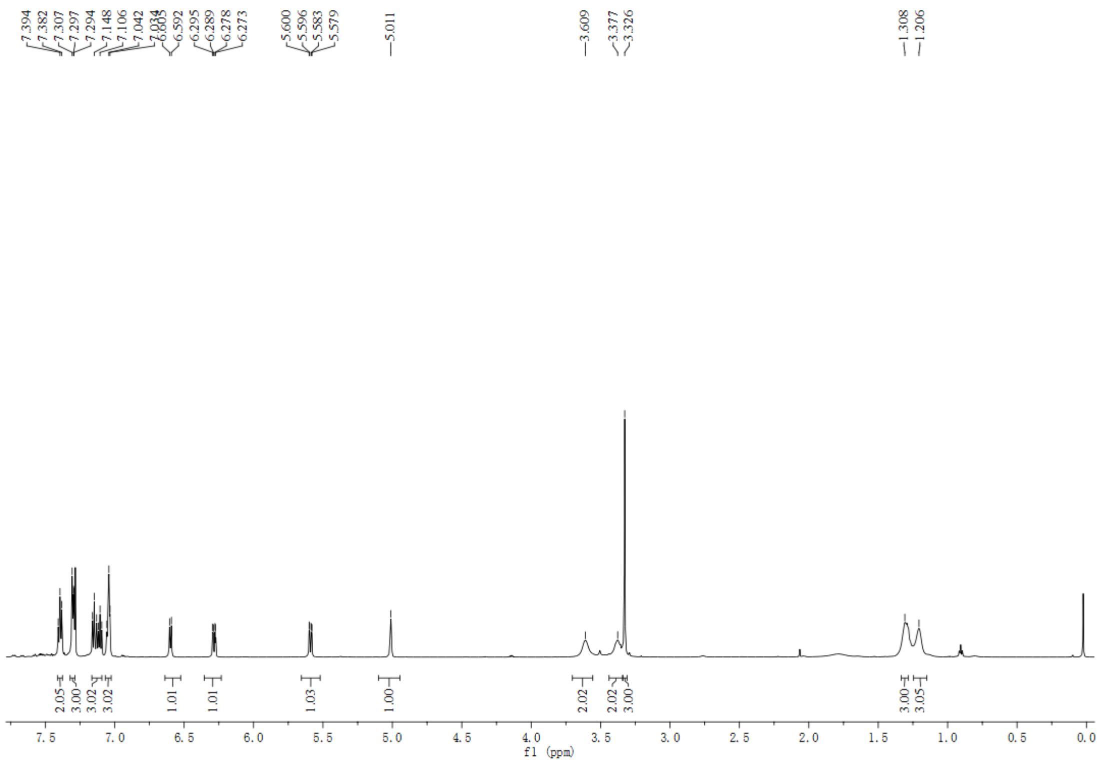

Supplementary Figure 109. ^{1}H NMR Spectra of compound **3s**.

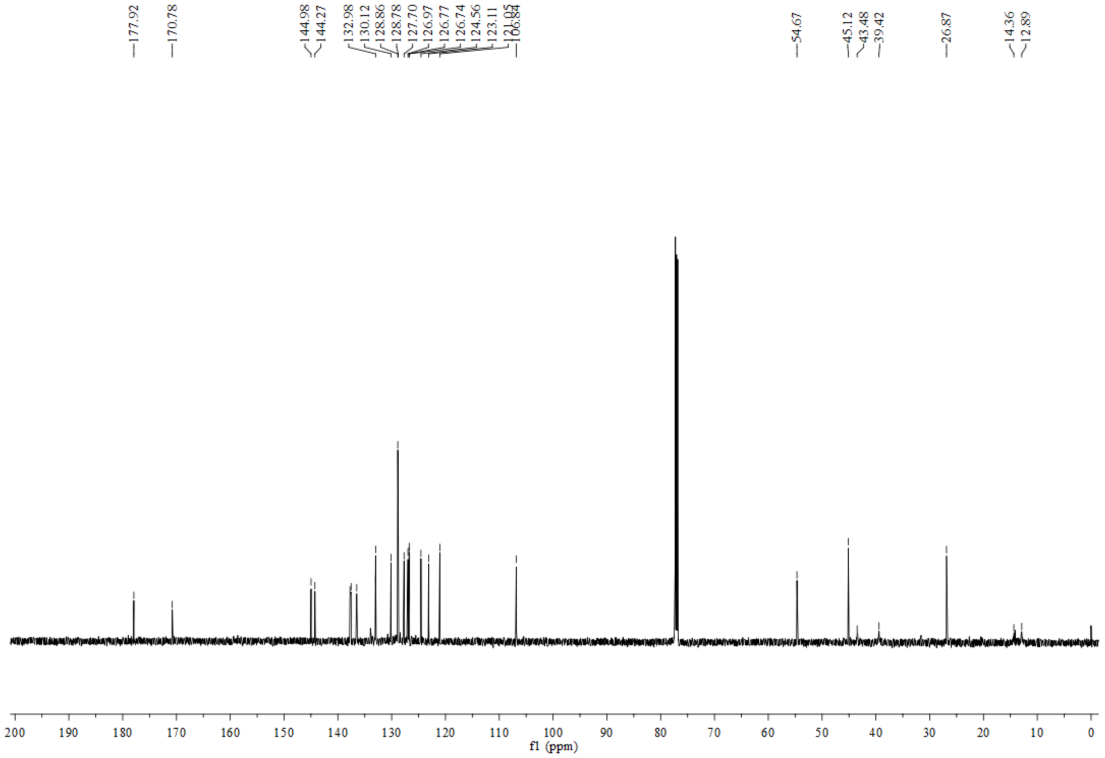

Supplementary Figure 110. ^{13}C NMR Spectra of compound **3s**.

5,6-Difluoro-1-methyl-4'-phenyl-4'*H*-spiro[indoline-3,1'-naphthalen]-2-one (**3t**)

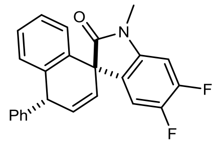

Purified by chromatography on silica gel, eluting with ethyl acetate/petroleum ether 1:15 (*v*/*v*); white solid, Mp = 146-148 °C, 58.5 mg, 78% yield; ^{1}H NMR (500 MHz, $CDCl_3$): δ 7.42-7.38 (m, 2H), 7.33-7.29 (m, 1H), 7.27 (d, *J* = 8.0 Hz, 2H), 7.16-7.12 (m, 1H), 7.08-7.03 (m, 2H), 7.01-6.97 (m, 1H), 6.85-6.81 (m, 1H), 6.58-6.56 (m, 1H), 6.29 (dd, *J* = 9.5, 3.0 Hz, 1H), 5.56 (dd, *J* = 10.0, 2.5 Hz, 1H), 5.00 (t, *J* = 2.5 Hz, 1H), 3.28 (s, 3H). ^{13}C NMR (125 MHz, $CDCl_3$): δ 177.7, 150.8 (dd, *J* = 247.5, 13.8 Hz), 147.1 (dd, *J* = 242.5, 13.8 Hz), 144.7, 140.1 (dd, *J* = 10.0, 2.5 Hz), 137.6, 133.4, 132.6, 130.7 (dd, *J* = 5.0, 3.8 Hz), 130.2, 128.84, 128.81, 127.8, 127.0, 126.8, 126.4, 122.8, 114.5 (d, *J* = 20.0 Hz), 98.5 (d, *J* = 23.8 Hz), 54.6, 45.0, 26.9. HRMS *m*/*z* (ESI+): Calculated for $C_{24}H_{18}F_2NO$ ($[M+H]^+$): 374.1351, found 374.1347.

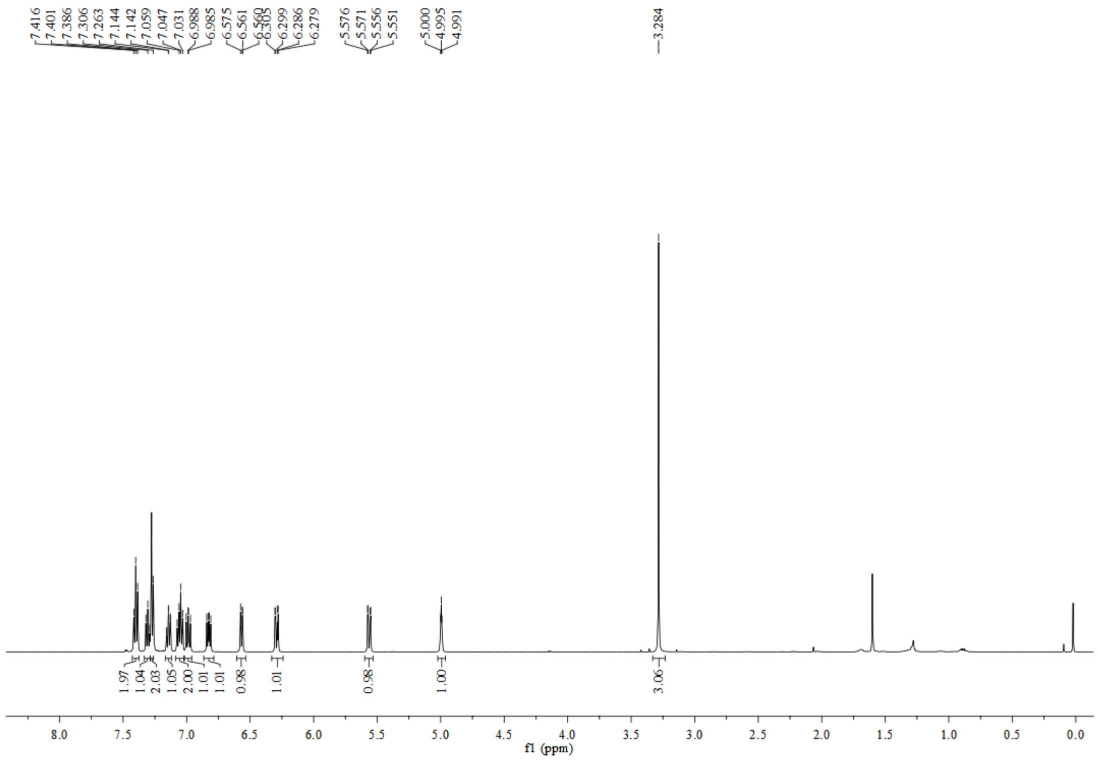

Supplementary Figure 111. ^{1}H NMR Spectra of compound **3t**.

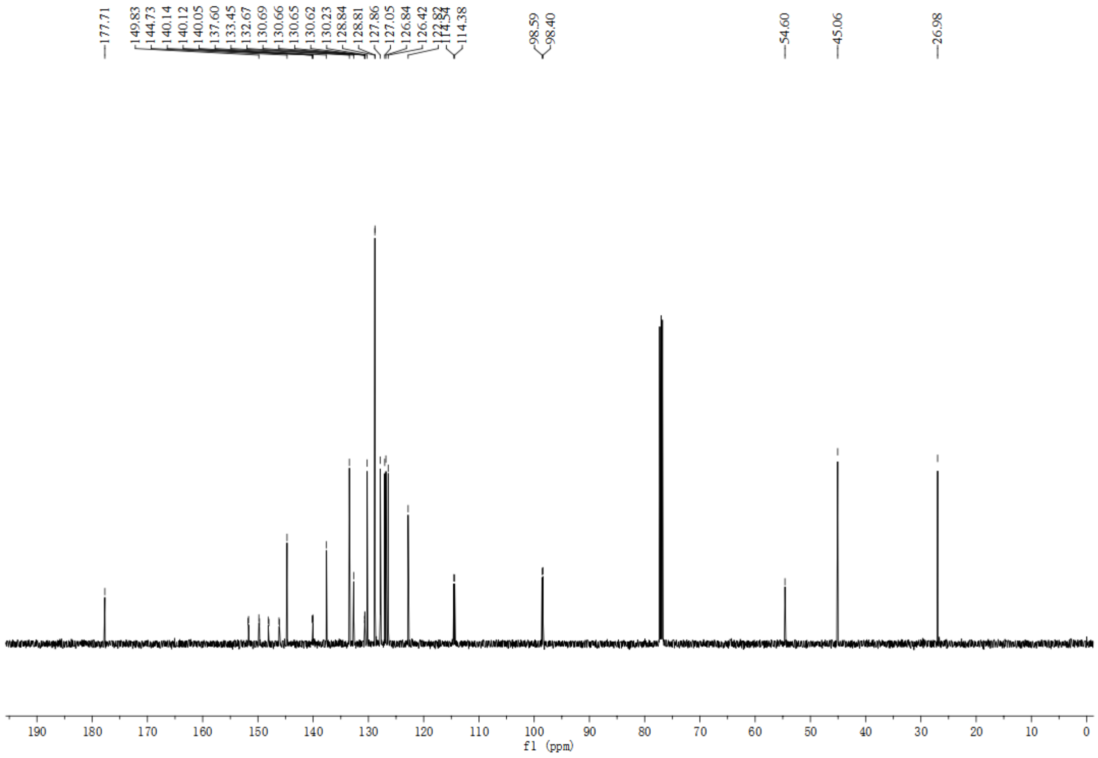

Supplementary Figure 112. ^{13}C NMR Spectra of compound **3t**.

1-Benzyl-4'-phenyl-4'*H*-spiro[indoline-3,1'-naphthalen]-2-one (**3u**)

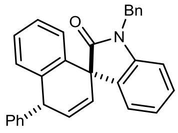

Purified by chromatography on silica gel, eluting with ethyl acetate/petroleum ether 1:15 (*v*/*v*); white solid, Mp = 143-145 °C, 78.8 mg, 95% yield; ^{1}H NMR (600 MHz, $CDCl_3$): δ 7.44-7.36 (m, 6H), 7.36-7.30 (m, 4H), 7.30-7.26 (m, 1H), 7.19 (d, *J* = 7.2 Hz, 1H), 7.17-7.13 (m, 1H), 7.11-7.08 (m, 1H), 7.08-7.04 (m, 2H), 6.91 (d, *J* = 7.8 Hz, 1H), 6.63 (d, *J* = 7.2 Hz, 1H), 6.32 (dd, *J* = 9.6, 3.0 Hz, 1H), 5.70 (dd, *J* = 9.6, 1.8 Hz, 1H), 5.08 (d, *J* = 15.6 Hz, 1H), 5.06 (s, 1H), 4.95 (d, *J* = 15.6 Hz, 1H). ^{13}C NMR (150 MHz, $CDCl_3$): δ 178.1, 145.1, 142.9, 137.7, 136.0, 135.5, 133.6, 132.9, 130.1, 128.9, 128.8, 128.7, 128.5, 127.7, 127.59, 127.53, 126.9, 126.74, 126.72, 124.9, 123.6, 123.4, 109.3, 54.7, 45.2, 44.2. HRMS *m/z* (ESI+): Calculated for $C_{30}H_{24}NO$ ($[M+H]^+$): 414.1852, found 414.1844.

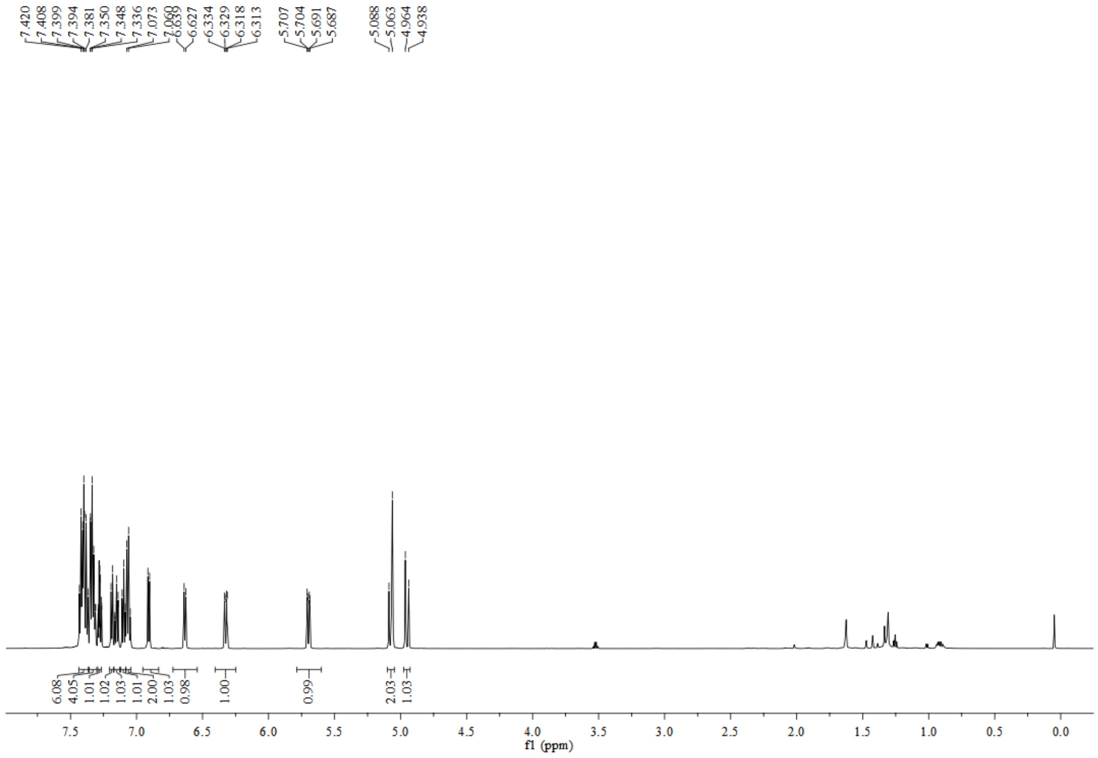

Supplementary Figure 113. ^{1}H NMR Spectra of compound **3u**.

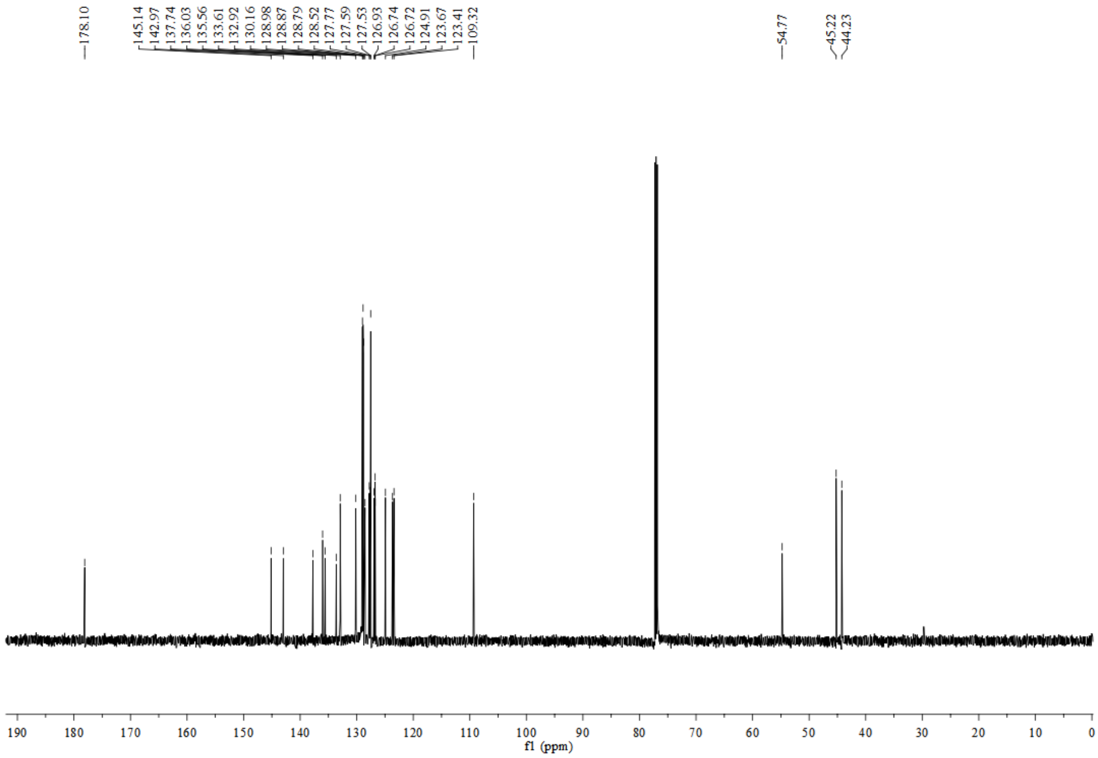

Supplementary Figure 114. ^{13}C NMR Spectra of compound **3u**.

1'-Methyl-4-phenyl-4*H*-spiro[naphthalene-1,3'-pyrrolo[2,3-b]pyridin]-2'(1'*H*)-one (**3v**)

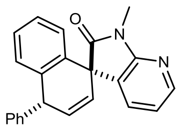

Purified by chromatography on silica gel, eluting with ethyl acetate/petroleum ether 1:5 (*v*/*v*); white solid, Mp = 93-95 °C, 48.6 mg, 72% yield; ^{1}H NMR (400 MHz, $CDCl_3$): δ 8.32-8.29 (m, 1H), 7.42-7.38 (m, 3H), 7.33-7.27 (m, 3H), 7.18-7.13 (m, 1H), 7.09-7.01 (m, 3H), 6.64-6.61 (m, 1H), 6.32 (dd, *J* = 9.6, 2.8 Hz, 1H), 5.60 (dd, *J* = 10.0, 2.8 Hz, 1H), 5.02 (t, *J* = 2.8 Hz, 1H), 3.42 (s, 3H). ^{13}C NMR (150 MHz, $CDCl_3$): δ 177.6, 157.4, 147.6, 144.8, 137.6, 133.4, 132.5, 132.1, 130.2, 130.0, 128.8, 127.9, 127.1, 126.8, 126.4, 122.5, 118.9, 54.2, 45.1, 25.9. HRMS *m*/*z* (ESI+): Calculated for $C_{23}H_{19}N_2O$ ([M+H]$^+$): 339.1492, found 339.1494.

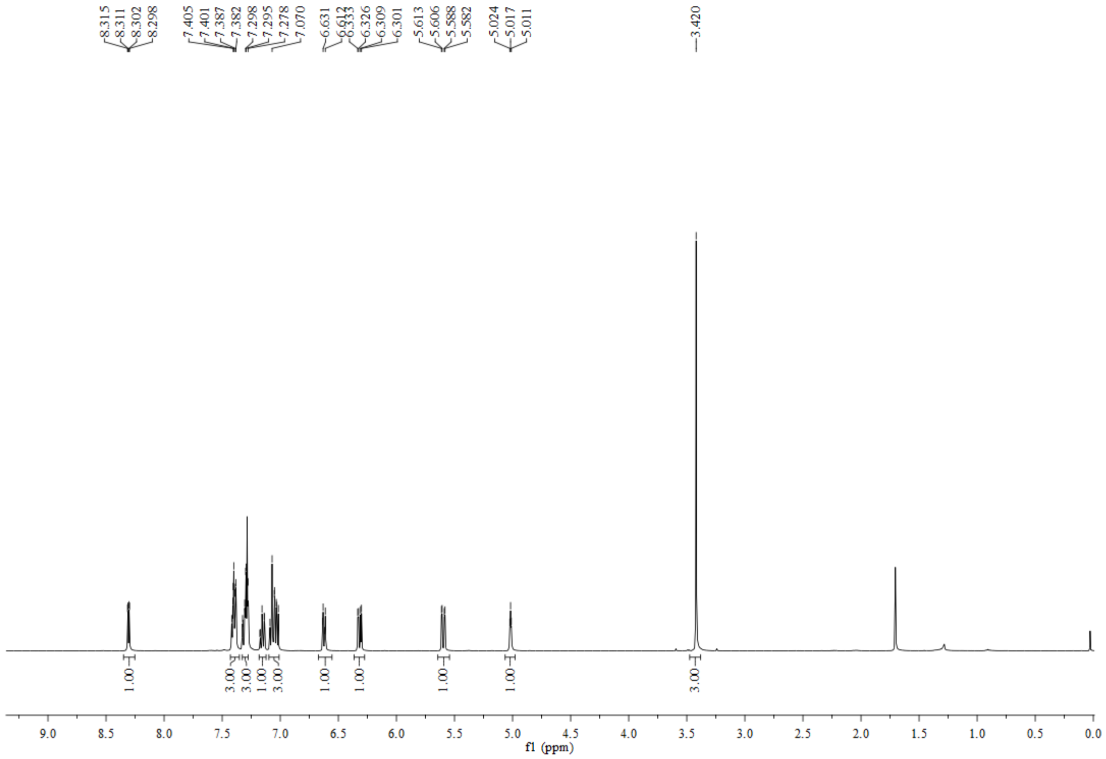

Supplementary Figure 115. ^{1}H NMR Spectra of compound **3v**.

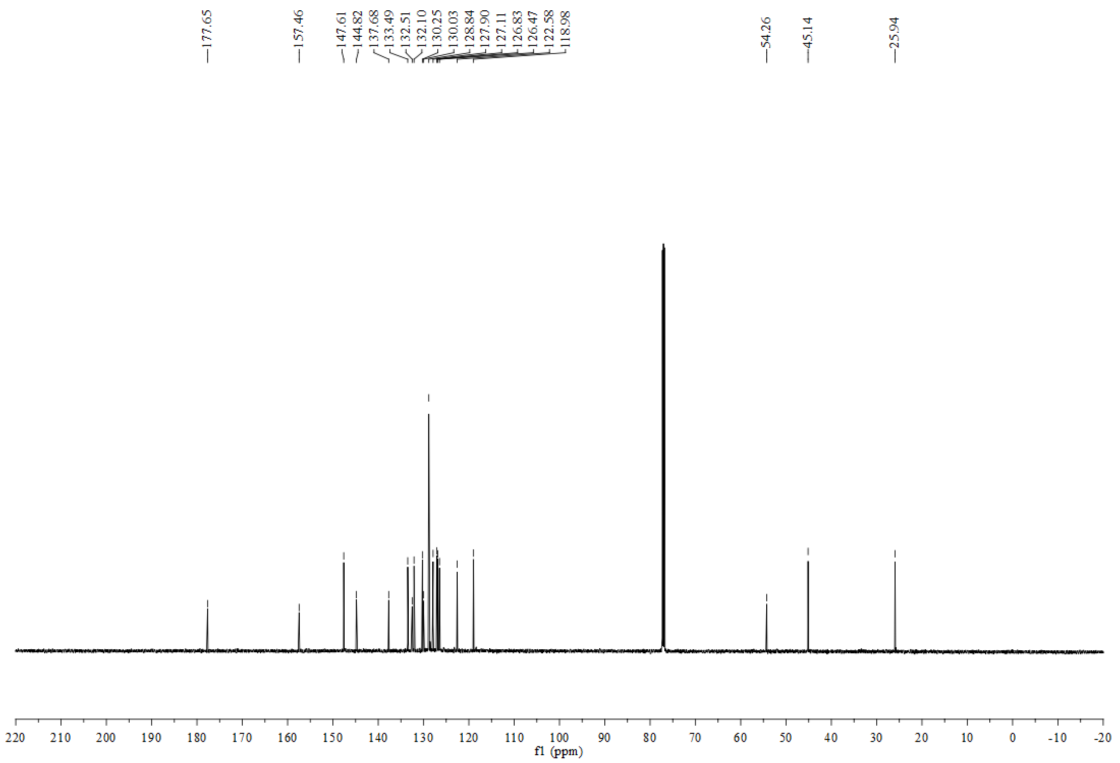

Supplementary Figure 116. ^{13}C NMR Spectra of compound **3v**.

6'-Methoxy-1-methyl-4'-phenyl-4'*H*-spiro[indoline-3,1'-naphthalen]-2-one (**3w**)

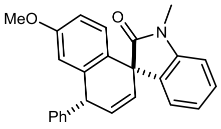

Purified by chromatography on silica gel, eluting with ethyl acetate/petroleum ether 1:10 (*v*/*v*); white solid, Mp = 182-184 °C, 67.1 mg, 91% yield; ^{1}H NMR (600 MHz, $CDCl_3$): δ 7.41-7.36 (m, 3H), 7.33-7.29 (m, 3H), 7.17-7.10 (m, 2H), 6.98 (d, *J* = 7.8 Hz, 1H), 6.62 (d, *J* = 9.0 Hz, 1H), 6.55-6.51 (m, 2H), 6.24 (d, *J* = 10.2 Hz, 1H), 5.60 (d, *J* = 9.6 Hz, 1H), 4.98 (s, 1H), 3.63 (s, 3H), 3.31 (s, 3H). ^{13}C NMR (150 MHz, $CDCl_3$): δ 178.2, 158.6, 144.9, 143.8, 139.0, 135.6, 132.4, 128.8, 128.7, 128.5, 127.8, 126.7, 125.9, 124.7, 123.8, 123.3, 114.5, 113.5, 108.2, 55.1, 54.3, 45.4, 26.7. HRMS *m*/*z* (ESI+): Calculated for $C_{25}H_{22}NO_2$ ($[M+H]^+$):368.1645, found 368.1643.

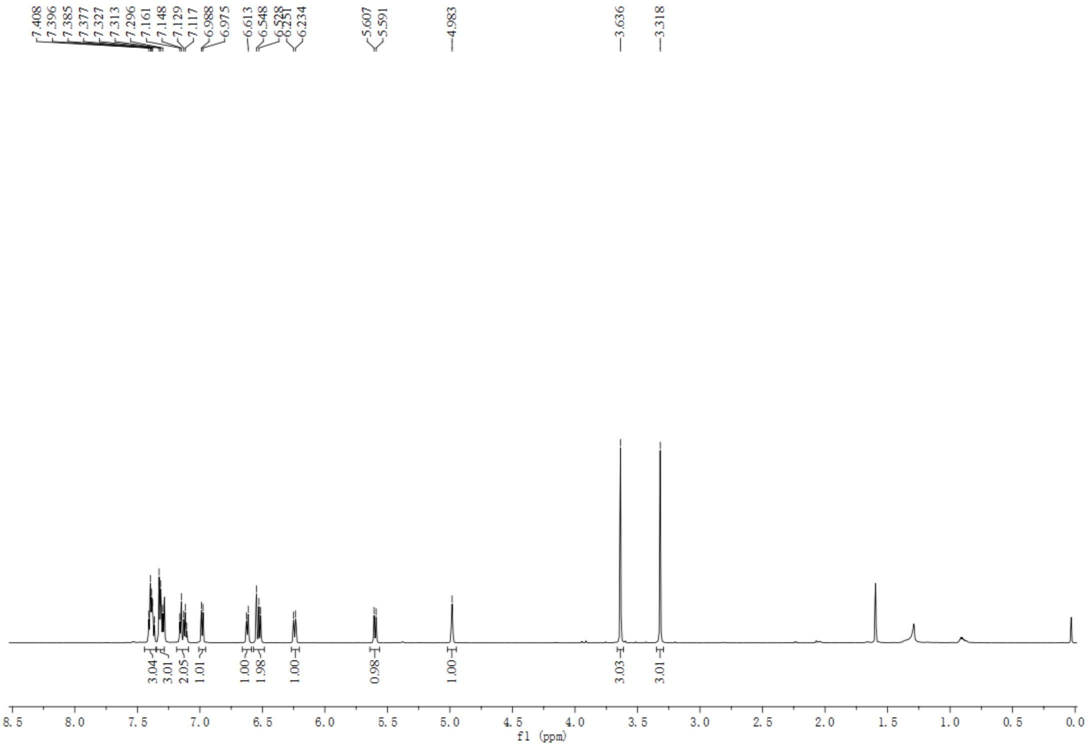

Supplementary Figure 117. ^{1}H NMR Spectra of compound **3w**.

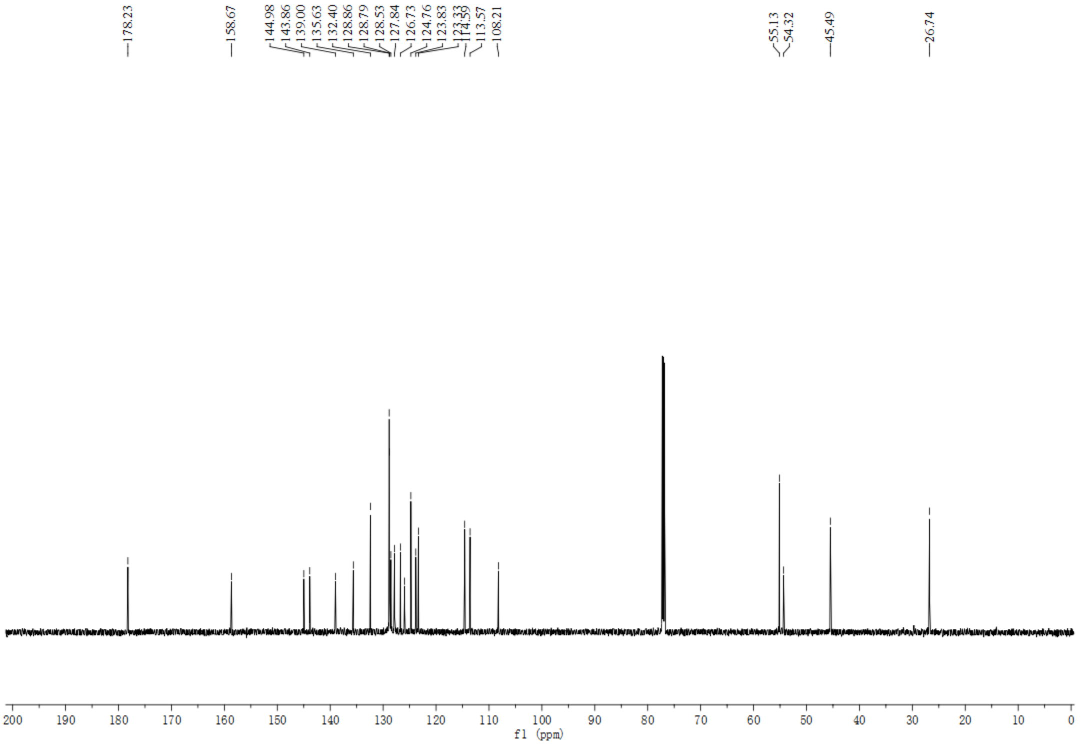

Supplementary Figure 118. ^{13}C NMR Spectra of compound **3w**.

1-Methyl-4',5'-diphenyl-4'*H*-spiro[indoline-3,1'-naphthalen]-2-one (**3x**)

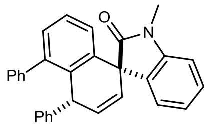

Purified by chromatography on silica gel, eluting with ethyl acetate/petroleum ether 1:10 (*v*/*v*); white solid, Mp = 103-105 °C, 58.4 mg, 71% yield; ^{1}H NMR (500 MHz, $CDCl_3$): δ 7.30-7.26 (m, 1H), 7.12 (d, *J* = 7.0 Hz, 1H), 7.09-6.99 (m, 5H), 6.97-6.66 (m, 7H), 6.60-6.54 (m, 3H), 6.03 (dd, *J* = 10.0, 4.0 Hz, 1H), 5.31 (dd, *J* = 9.5, 1.9 Hz, 1H), 4.98-4.96 (m, 1H), 3.27 (s, 3H). ^{13}C NMR (125 MHz, $CDCl_3$): δ 178.4, 144.9, 143.7, 143.4, 141.7, 136.0, 134.6, 134.1, 132.9, 129.9, 128.9, 128.5, 128.0, 127.5, 127.1, 126.3, 126.2, 125.5, 124.7, 123.3, 120.7, 108.3, 55.0, 43.6, 26.8. HRMS *m*/*z* (ESI+): Calculated for $C_{30}H_{24}NO$ ([M+H]$^+$): 414.1852, found 414.1853.

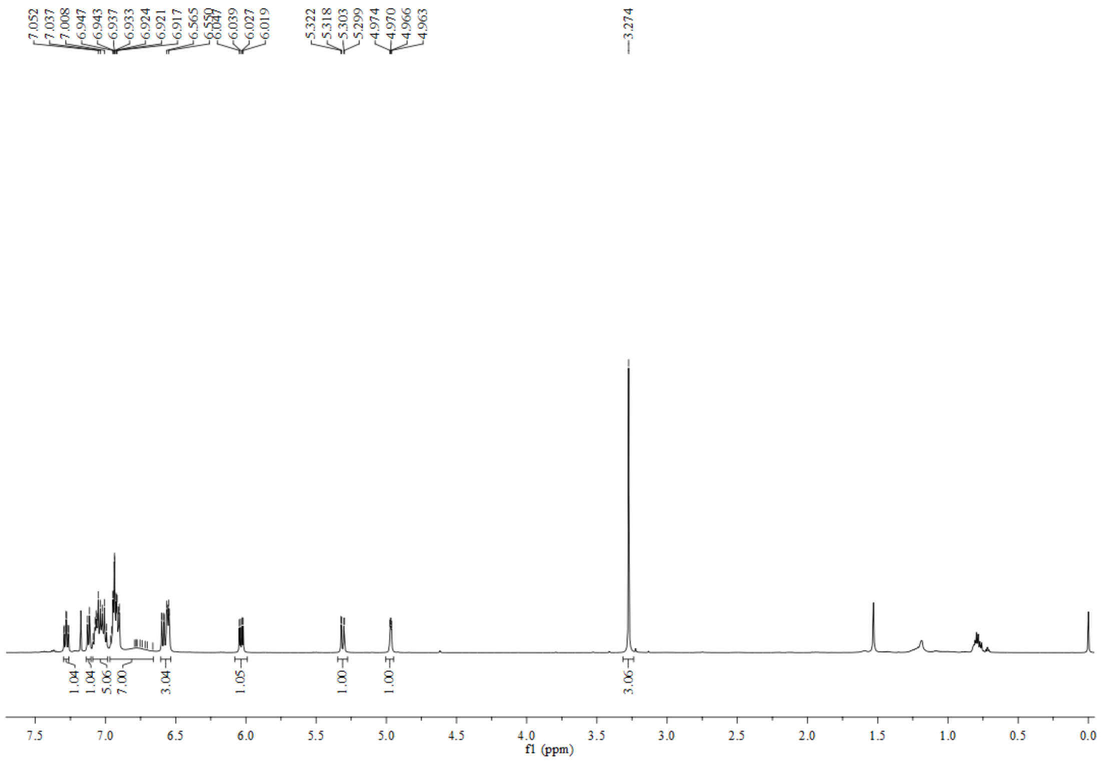

Supplementary Figure 119. ^{1}H NMR Spectra of compound **3x**.

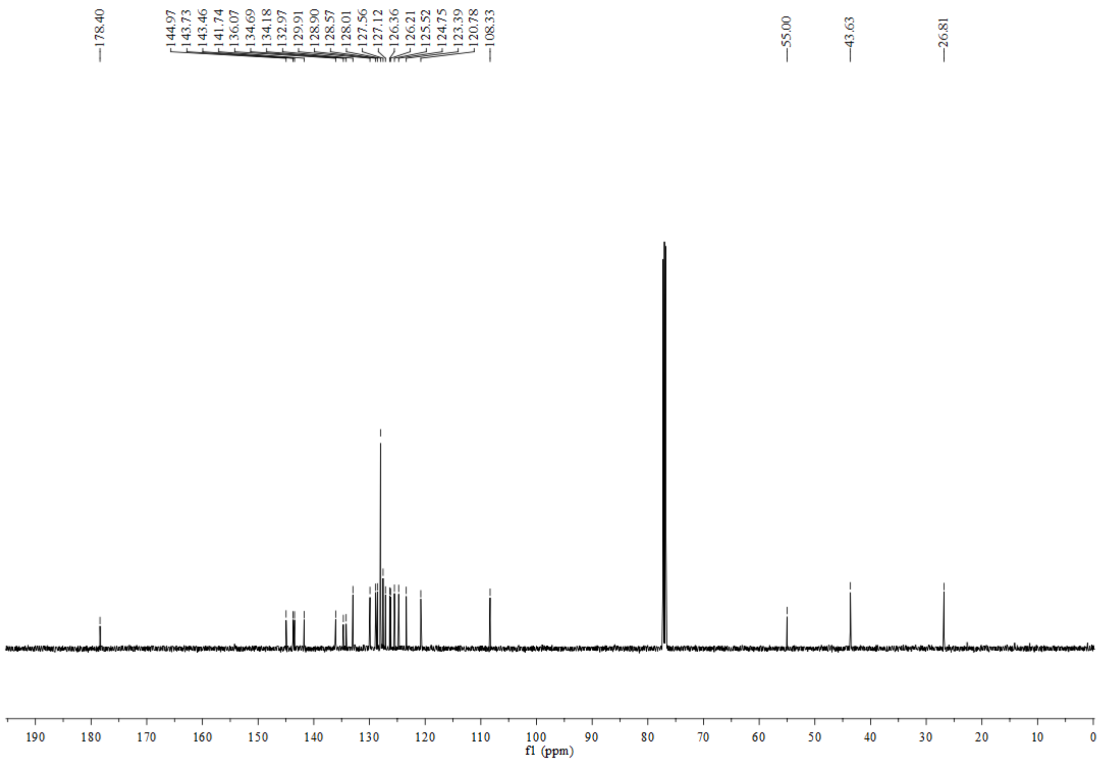

Supplementary Figure 120. ^{13}C NMR Spectra of compound **3x**.

1-Methyl-4',6-diphenyl-4'*H*-spiro[indoline-3,1'-naphthalen]-2-one (**3y**)

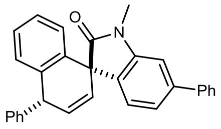

Purified by chromatography on silica gel, eluting with ethyl acetate/petroleum ether 1:15 (*v*/*v*); white solid, Mp = 95-97 ^{o}C, 48.3 mg, 58% yield; ^{1}H NMR (500 MHz, $CDCl_3$): δ 7.68-7.66 (m, 2H), 7.53-7.49 (m, 2H), 7.44-7.39 (m, 3H), 7.36-7.29 (m, 4H), 7.22 (d, *J* = 8.0 Hz, 1H), 7.19 (d, *J* = 1.5 Hz, 1H), 7.16-7.12 (m, 1H), 7.08-7.04 (m, 2H), 6.70-6.67 (m, 1H), 6.30 (dd, *J* = 10.0, 3.0 Hz, 1H), 5.66 (dd, *J* = 10.0, 2.5 Hz, 1H), 5.03 (s, 1H), 3.38 (s, 3H). ^{13}C NMR (125 MHz, $CDCl_3$): δ 178.2, 145.1, 144.4, 142.2, 140.9, 137.6, 134.5, 133.5, 132.7, 130.1, 128.93, 128.90, 128.7, 127.7, 127.5, 127.2, 126.9, 126.78, 126.72, 124.9, 123.6, 122.3, 107.1, 54.6, 45.2, 26.8. HRMS *m*/*z* (ESI+): Calculated for $C_{30}H_{24}NO$ ($[M+H]^+$): 414.1852, found 414.1848.

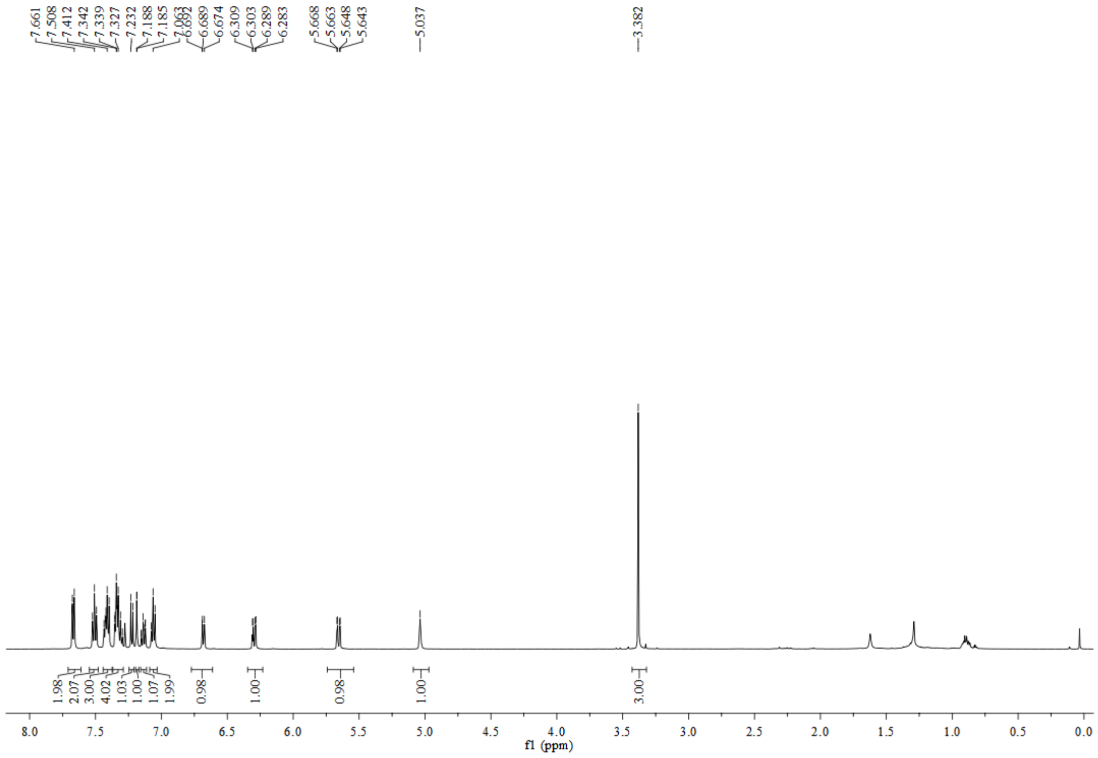

Supplementary Figure 121. ^{1}H NMR Spectra of compound **3y**.

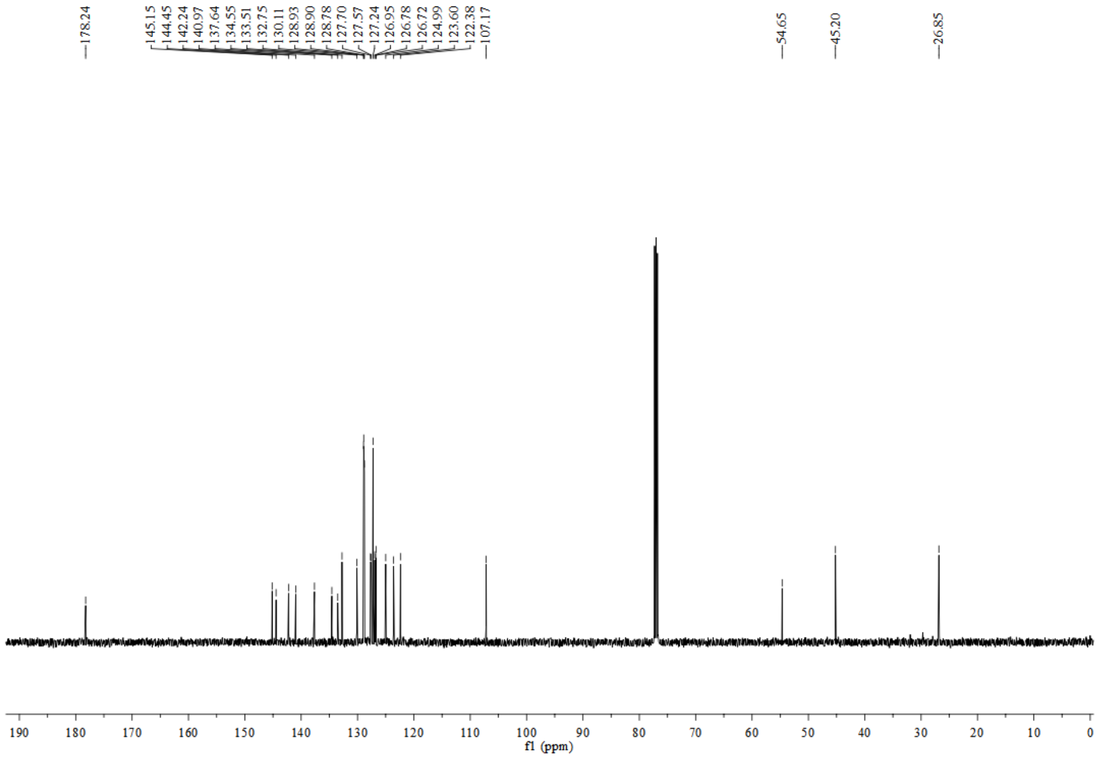

Supplementary Figure 122. ^{13}C NMR Spectra of compound **3y**.

1'-Methyl-4-phenyl-4*H*-spiro[anthracene-1,3'-indolin]-2'-one (**3z**)

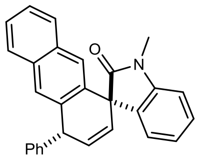

Purified by chromatography on silica gel, eluting with ethyl acetate/petroleum ether 1:15 (*v*/*v*); white solid, Mp = 166-168 ^{o}C, 68.2 mg, 88% yield; ^{1}H NMR (600 MHz, $CDCl_3$): δ 7.62 (d, *J* = 7.8 Hz, 1H), 7.57 (d, *J* = 7.8 Hz, 1H), 7.49 (s, 1H), 7.47-7.42 (m, 3H), 7.40-7.37 (m, 2H), 7.36-7.31 (m, 3H), 7.23-7.21 (m, 1H), 7.19-7.15 (m, 1H), 7.14 (s, 1H), 7.06 (d, *J* = 7.8 Hz, 1H), 6.39 (dd, *J* = 10.2, 3.0 Hz, 1H), 5.76 (dd, *J* = 9.6, 3.0 Hz, 1H), 5.29 (s, 1H), 3.34 (s, 3H). ^{13}C NMR (150 MHz, $CDCl_3$): δ 178.0, 145.5, 144.1, 136.3, 135.6, 133.8, 132.9, 132.7, 132.1, 129.1, 128.9, 128.8, 128.7, 127.49, 127.40, 126.7, 125.8, 125.65, 125.61, 124.9, 124.6, 123.5, 108.3, 55.2, 45.4, 26.8. HRMS *m*/*z* (ESI+): Calculated for $C_{28}H_{22}NO$ ([M+H]$^{+}$): 388.1696, found 388.1684.

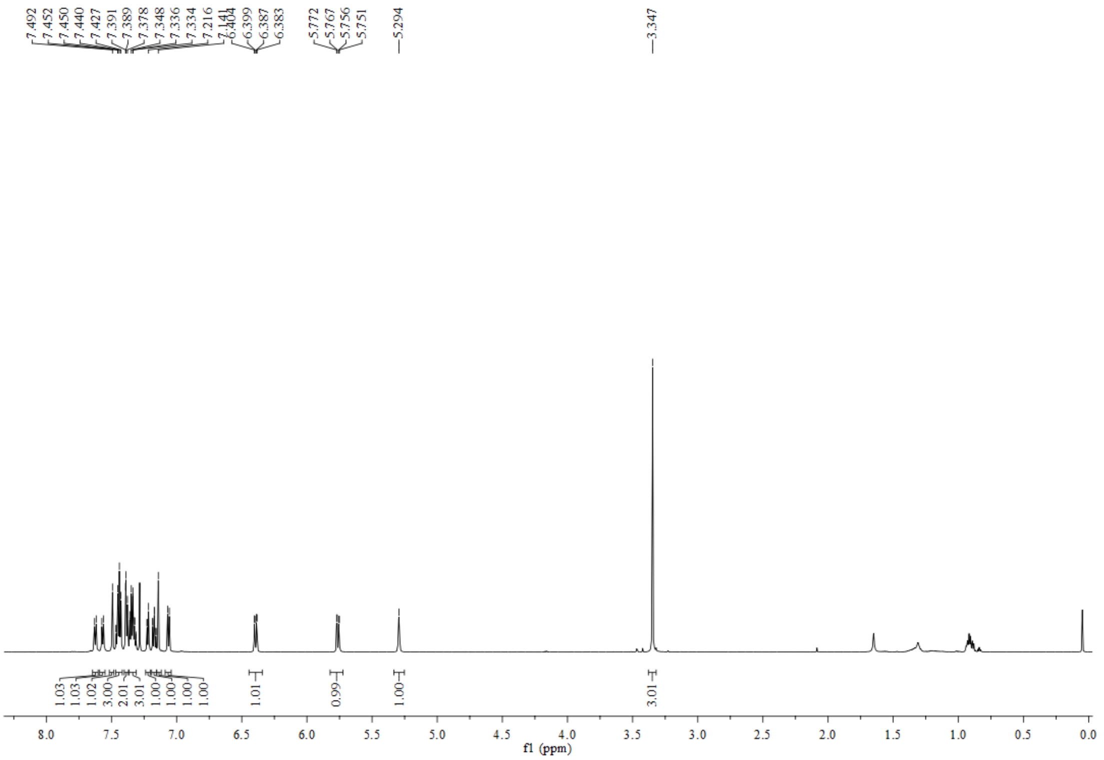

Supplementary Figure 123. ^{1}H NMR Spectra of compound **3z**.

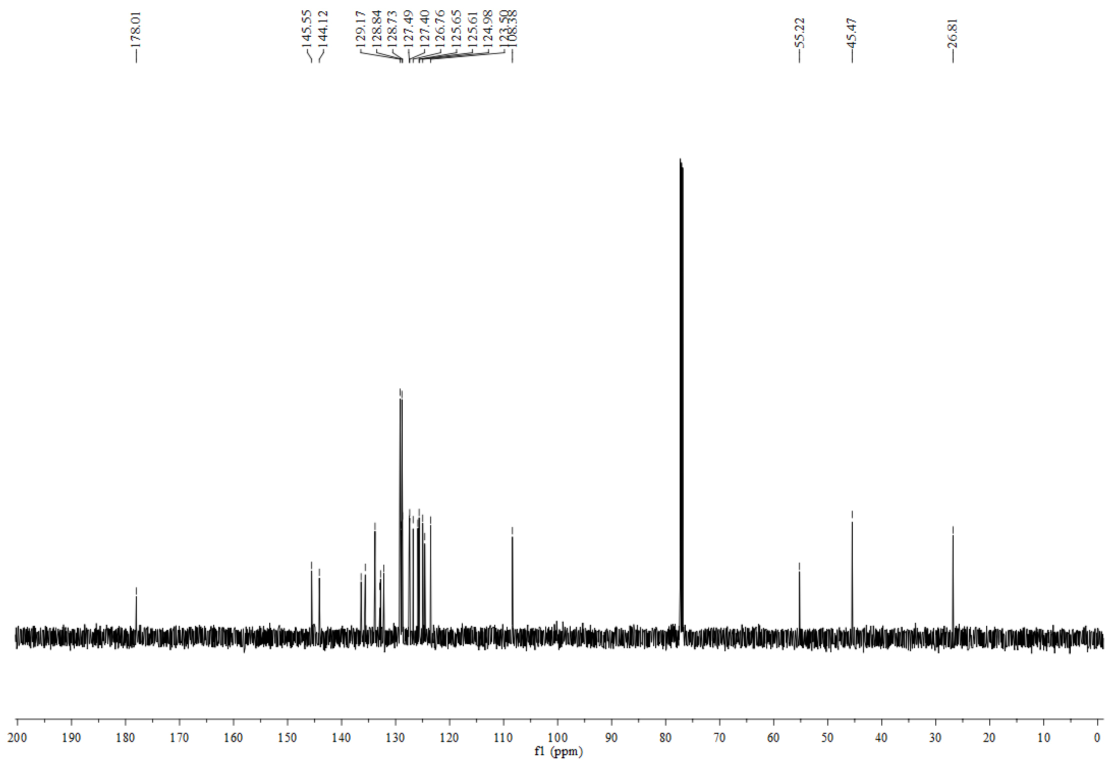

Supplementary Figure 124. ^{13}C NMR Spectra of compound **3z**.

4'-Methoxy-1-methyl-4'-phenyl-4'*H*-spiro[indoline-3,1'-naphthalen]-2-one (**9**)

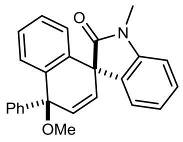

Purified by chromatography on silica gel, eluting with ethyl acetate/petroleum ether 1:15 (*v*/*v*); white solid, Mp = 180-182 °C, 37.3 mg, 51% yield; ^{1}H NMR (600 MHz, $CDCl_3$): δ 7.52 (d, *J* = 7.8 Hz, 2H), 7.40-7.37 (m, 3H), 7.33 (d, *J* = 7.8 Hz, 1H), 7.27 (d, *J* = 7.8 Hz, 1H), 7.24-7.21 (m, 1H), 7.15-7.11 (m, 1H), 7.11-7.07 (m, 1H), 7.05 (d, *J* = 7.2 Hz, 1H), 7.02 (d, *J* = 7.8 Hz, 1H), 6.66 (d, *J* = 8.4 Hz, 1H), 6.02 (d, *J* = 10.2 Hz, 1H), 5.80 (d, *J* = 10.2 Hz, 1H), 3.38 (s, 3H), 3.37 (s, 3H). ^{13}C NMR (150 MHz, $CDCl_3$): δ 177.3, 147.2, 143.8, 137.3, 135.8, 134.7, 132.7, 129.5, 128.8, 128.1, 128.0, 126.9, 126.8, 126.4, 126.3, 124.5, 123.4, 108.4, 77.3, 54.3, 51.8, 26.8. HRMS *m*/*z* (ESI+): Calculated for $C_{25}H_{21}NNaO_2$ ($[M+Na]^+$): 390.1465, found 390.1466.

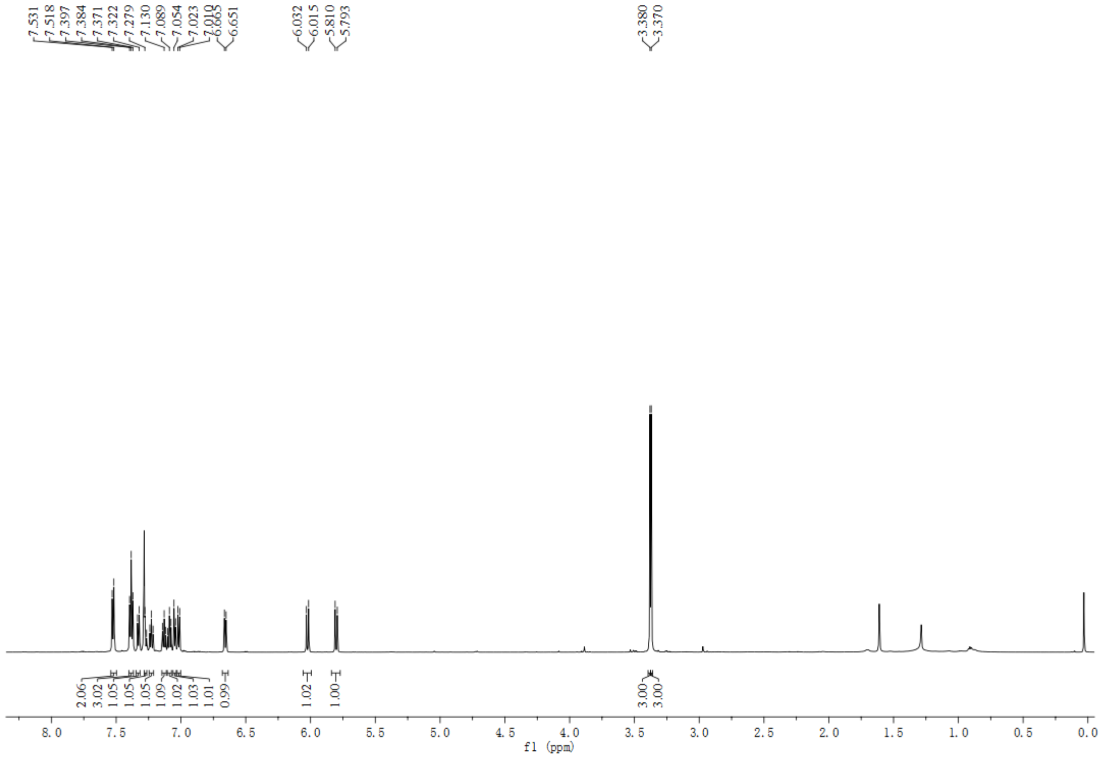

Supplementary Figure 125. ^{1}H NMR Spectra of compound **9**.

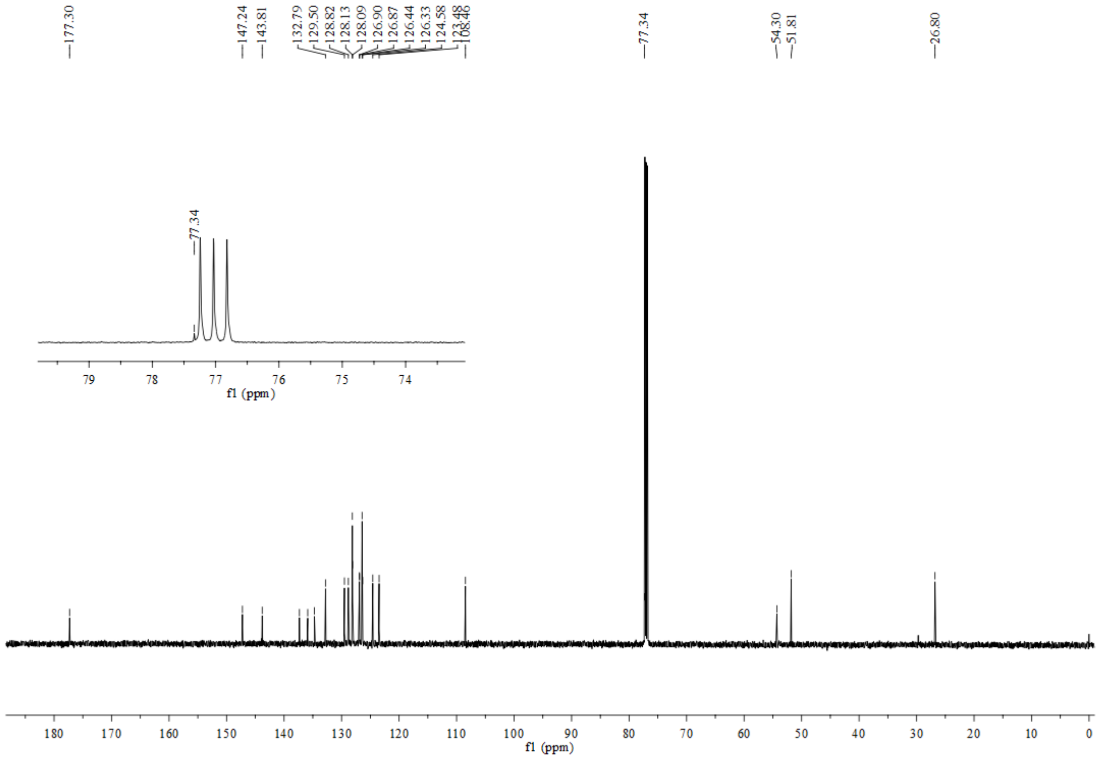

Supplementary Figure 126. ^{13}C NMR Spectra of compound **9**.

4'-Methoxy-1-methyl-2'-phenyl-2'*H*-spiro[indoline-3,1'-naphthalen]-2-one (**10**)

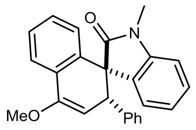

Purified by chromatography on silica gel, eluting with ethyl acetate/petroleum ether 1:15 (*v*/*v*); white solid, Mp = 212-214 ^{o}C, 16.0 mg, 22% yield; ^{1}H NMR (600 MHz, $CDCl_3$): δ 7.77-7.74 (m, 1H), 7.33-7.29 (m, 2H), 7.21-7.18 (m, 1H), 7.14-7.07 (m, 2H), 7.06-7.02 (m, 2H), 6.98-6.96 (m, 2H), 6.91-6.87 (m, 1H), 6.87-6.85 (m, 1H), 6.49 (d, *J* = 7.2 Hz, 1H), 5.04 (d, *J* = 2.4 Hz, 1H), 4.71 (d, *J* = 2.4 Hz, 1H), 3.88 (s, 3H), 2.96 (s, 3H). ^{13}C NMR (125 MHz, C_6D_6): δ 176.9, 152.5, 141.1, 138.7, 135.9, 130.0, 129.3, 128.2, 128.0, 126.8, 126.4, 126.1, 125.8, 125.2, 123.7, 121.8, 120.5, 106.5, 96.4, 58.2, 53.2, 47.9, 24.2. HRMS *m*/*z* (ESI+): Calculated for $C_{25}H_{22}NO_2$ ($[M+H]^+$): 368.1645, found 368.1637.

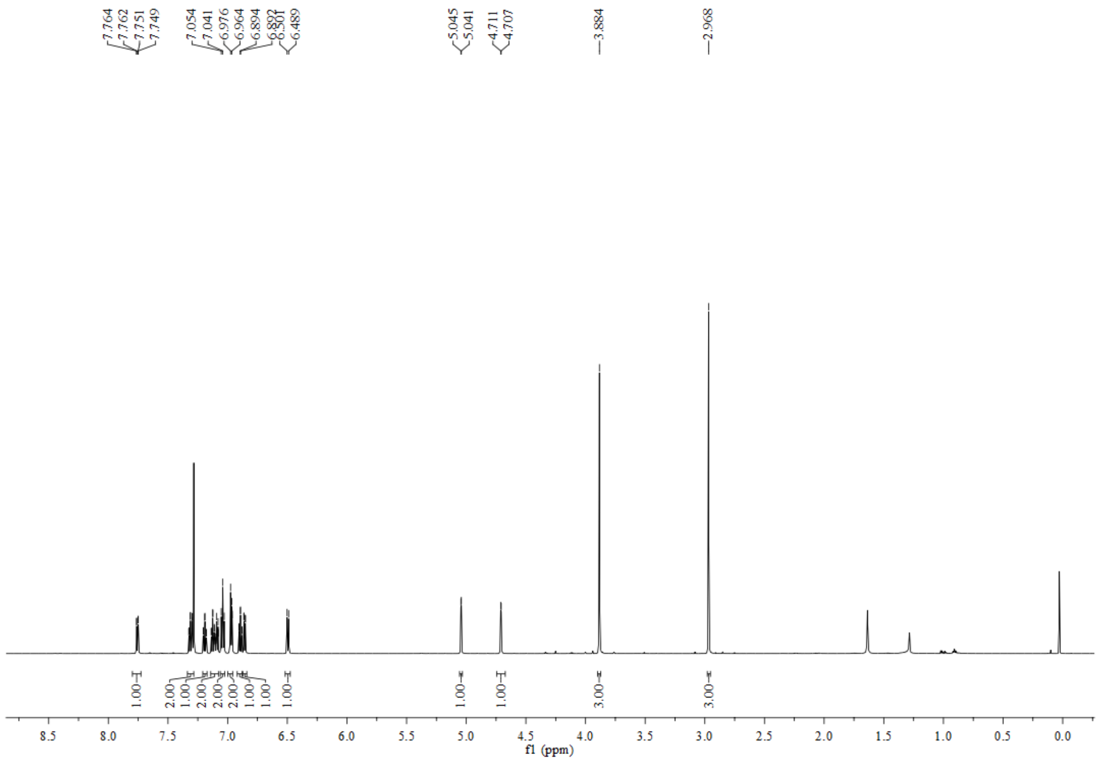

Supplementary Figure 127. ^{1}H NMR Spectra of compound **10**.

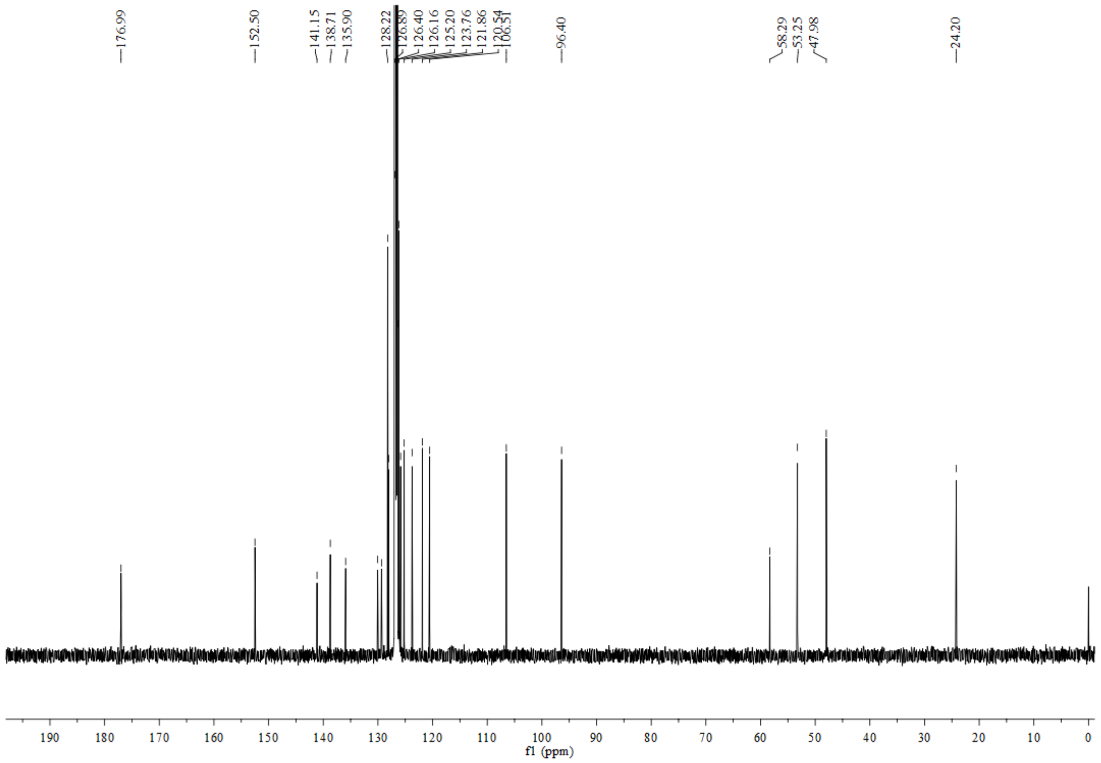

Supplementary Figure 128. ^{13}C NMR Spectra of compound **10**.

1-Methyl-4'-(*o*-tolyl)-4'*H*-spiro[indoline-3,1'-naphthalen]-2-one (**3aa**)

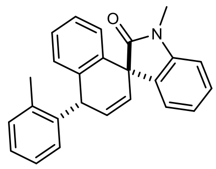

Purified by chromatography on silica gel, eluting with ethyl acetate/petroleum ether 1:15 (*v*/*v*); white solid, Mp = 153-155 ^{o}C, 57.2 mg, 81% yield; ^{1}H NMR (500 MHz, $CDCl_3$): δ 7.42-7.37 (td, *J* = 7.6, 1.3 Hz, 1H), 7.27-7.19 (m, 3H), 7.18-7.09 (m, 4H), 7.05-7.01 (m, *J* = 7.4 Hz, 1H), 6.99 (d, *J* = 8.0 Hz, 1H), 6.91 (d, *J* = 7.5 Hz, 1H), 6.60 (d, *J* = 7.5 Hz, 1H), 6.26 (dd, *J* = 10.0, 3.0 Hz, 1H), 5.63 (dd, *J* = 10.0, 3.0 Hz, 1H), 5.29 (s, 1H), 3.31 (s, 3H), 2.45 (s, 3H). ^{13}C NMR (125 MHz, $CDCl_3$): δ 177.9, 144.0, 138.0, 136.2, 135.6, 133.7, 132.6, 130.7, 129.8, 129.3, 128.6, 127.5, 126.75, 126.72, 126.5, 124.8, 123.7, 123.3, 108.1, 54.9, 26.7, 19.8. HRMS *m*/*z* (ESI+): Calculated for $C_{25}H_{22}NO$ ($[M+H]^+$): 352.1696, found 352.1699.

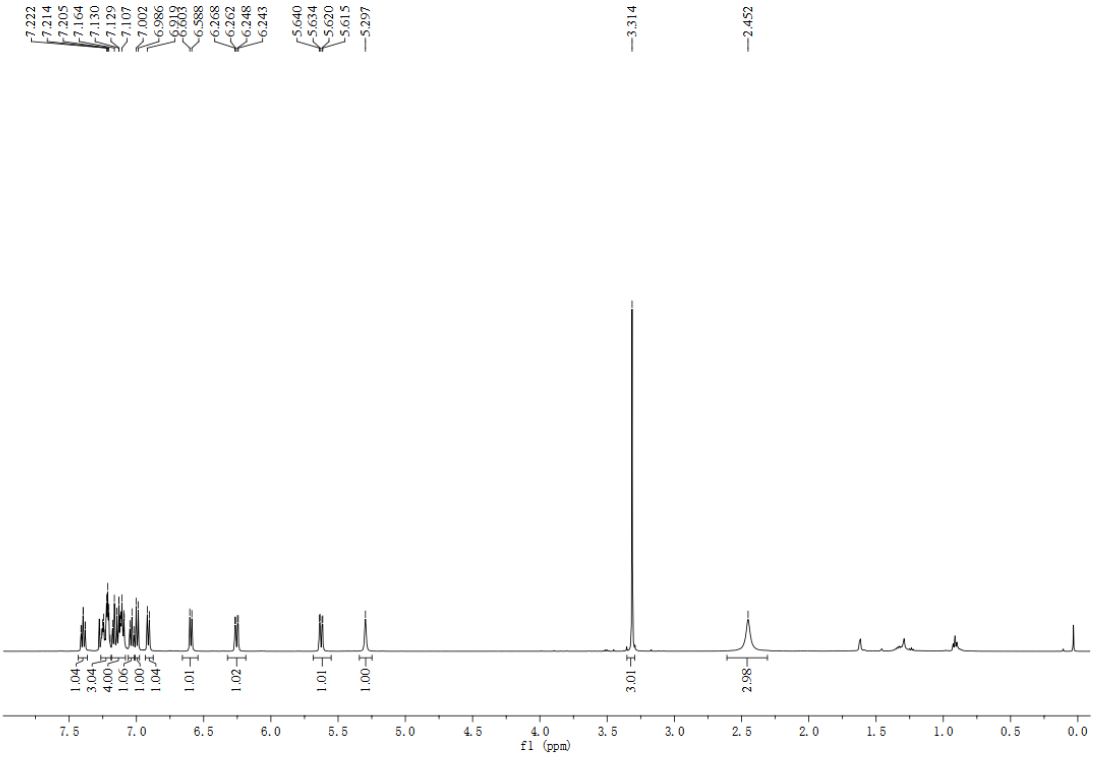

Supplementary Figure 129. ^{1}H NMR Spectra of compound **3aa**.

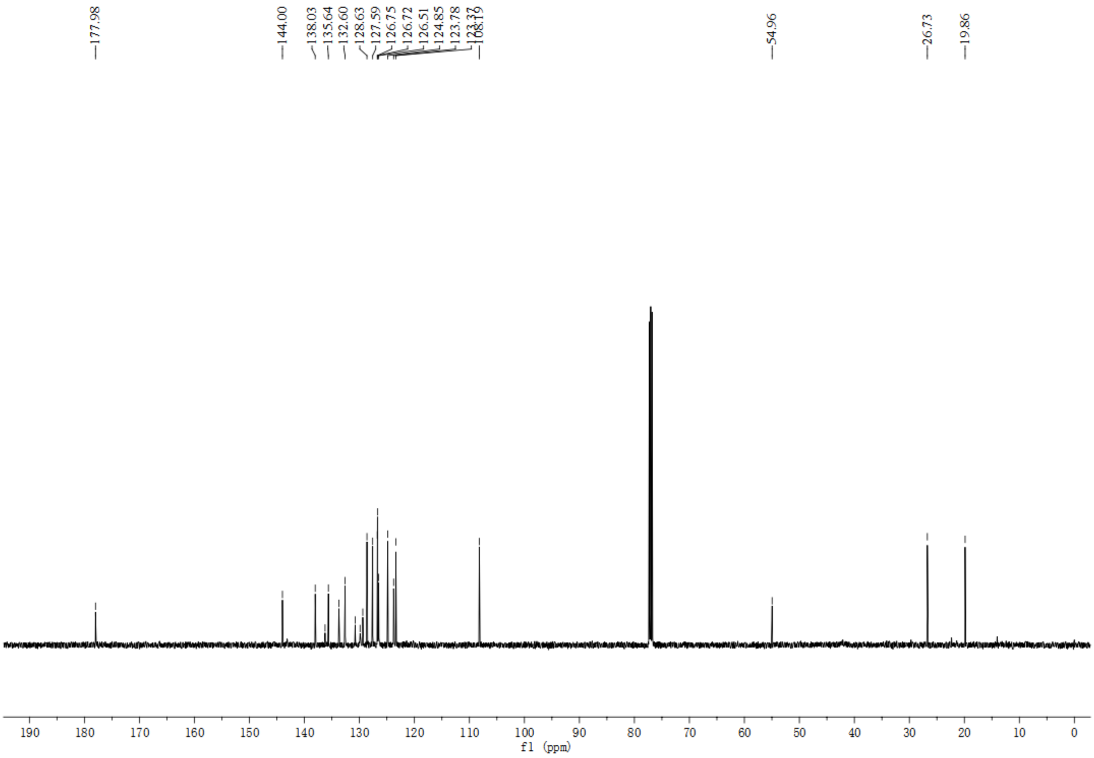

Supplementary Figure 130. ^{13}C NMR Spectra of compound **3aa**.

1-Methyl-4'-(*m*-tolyl)-4'*H*-spiro[indoline-3,1'-naphthalen]-2-one (**3ab**)

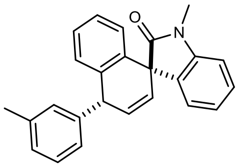

Purified by chromatography on silica gel, eluting with ethyl acetate/petroleum ether 1:15 (*v*/*v*); white solid, Mp = 129-131 °C, 52.5 mg, 75% yield; ^{1}H NMR (500 MHz, $CDCl_3$): δ 7.41-7.37 (m, 1H), 7.31-7.27 (m, 1H), 7.18 (d, *J* = 7.0 Hz, 1H), 7.14- 7.09 (m, 5H), 7.06-6.98 (m, 3H), 6.60 (d, *J* = 8.0 Hz, 1H), 6.27 (dd, *J* = 10.0, 3.0 Hz, 1H), 5.61 (dd, *J* = 10.0, 2.5 Hz, 1H), 4.98 (t, *J* = 2.5 Hz, 1H), 3.32 (s, 3H), 2.40 (s, 3H). ^{13}C NMR (125 MHz, $CDCl_3$): δ 178.0, 145.0, 143.9, 138.3, 137.7, 135.6, 133.5, 132.8, 130.0, 129.6, 128.6, 128.5, 127.47, 127.44, 126.8, 126.6, 125.9, 124.8, 123.5, 123.3, 108.2, 54.7, 45.1, 26.7, 21.4. HRMS *m*/*z* (ESI+): Calculated for $C_{25}H_{22}NO$ ($[M+H]^+$): 352.1696, found 352.1709.

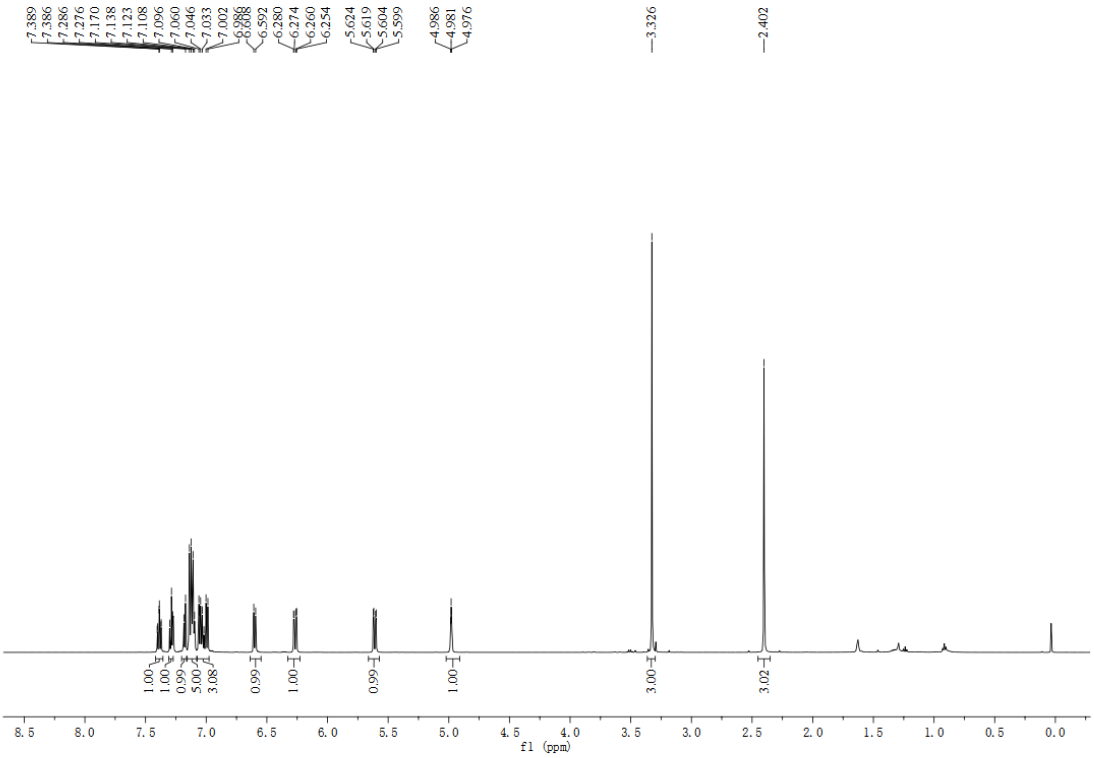

Supplementary Figure 131. ^{1}H NMR Spectra of compound **3ab**.

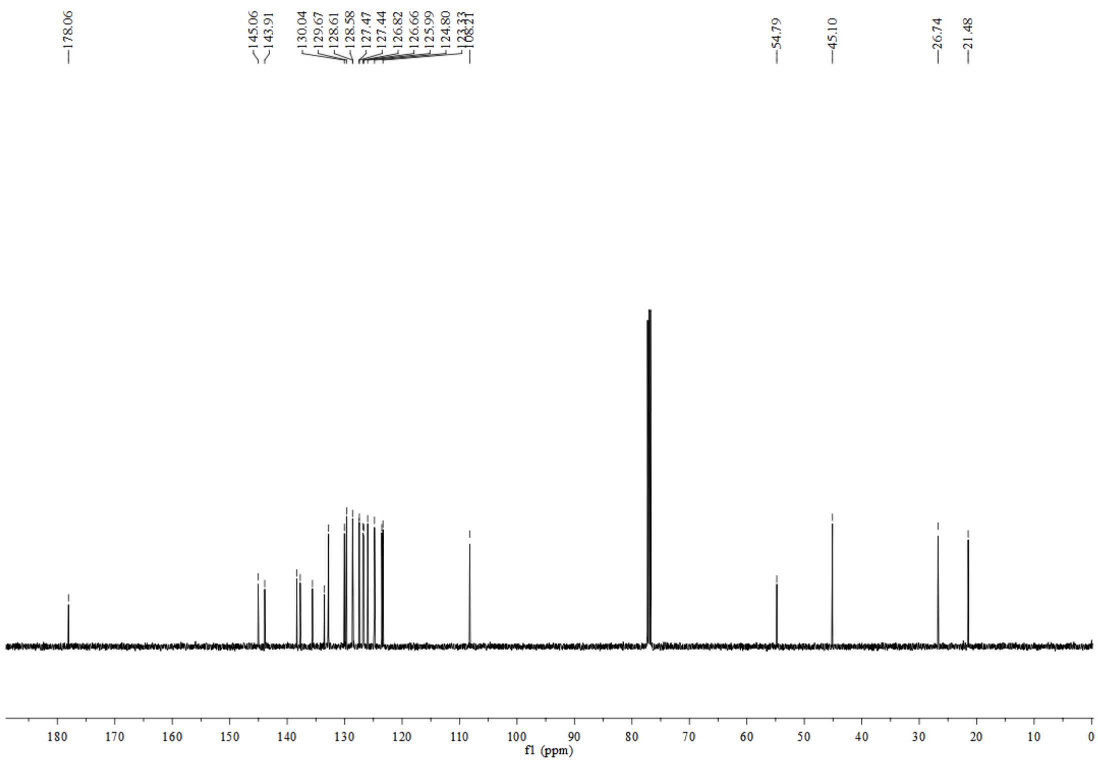

Supplementary Figure 132. ^{13}C NMR Spectra of compound **3ab**.

4'-(3-Fluorophenyl)-1-methyl-4'*H*-spiro[indoline-3,1'-naphthalen]-2-one (**3ac**)

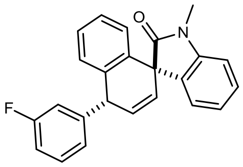

Purified by chromatography on silica gel, eluting with ethyl acetate/petroleum ether 1:15 (*v*/*v*); white solid, Mp = 164-166 °C, 54.9 mg, 77% yield; ^{1}H NMR (500 MHz, $CDCl_3$): δ 7.41-7.33 (m, 2H), 7.15-7.11 (m, 4H), 7.06-6.96 (m, 5H), 6.59 (dd, *J* = 7.5, 1.0 Hz, 1H), 6.23 (dd, *J* = 10.0, 3.0 Hz, 1H), 5.63 (dd, *J* = 9.5, 2.5 Hz, 1H), 5.01 (s, 1H), 3.31 (s, 3H). ^{13}C NMR (125 MHz, $CDCl_3$): δ 177.8, 163.2 (d, *J* = 245.0 Hz), 147.7 (d, *J* =6.3 Hz), 143.9, 136.9, 135.3, 133.5, 132.0, 130.2 (d, *J* = 7.5 Hz), 129.9, 128.7, 127.6, 127.1, 126.8, 124.7, 124.6 (d, *J* = 2.5 Hz), 124.2, 123.4, 115.8 (d, *J* = 21.2 Hz), 113.7 (d, *J* = 20.0 Hz), 108.2, 54.7, 44.8, 26.7. HRMS *m*/*z* (ESI+): Calculated for $C_{24}H_{19}FNO$ ($[M+H]^+$): 356.1445, found 356.1439.

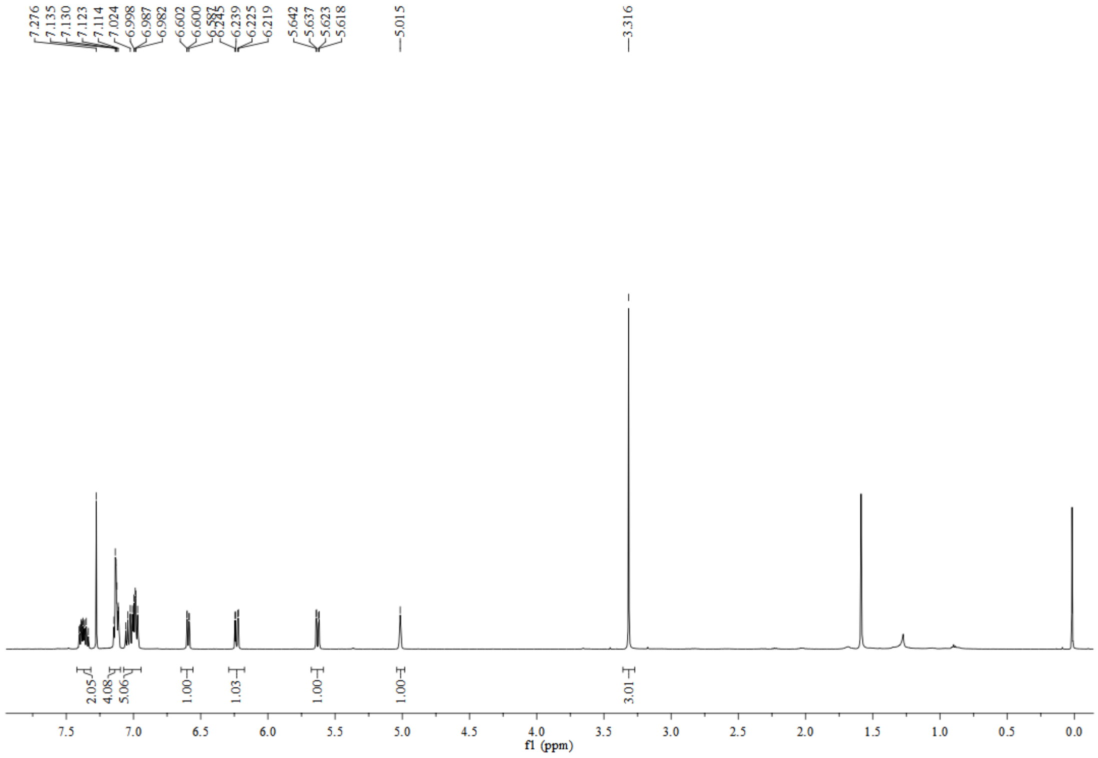

Supplementary Figure 133. ^{1}H NMR Spectra of compound **3ac**.

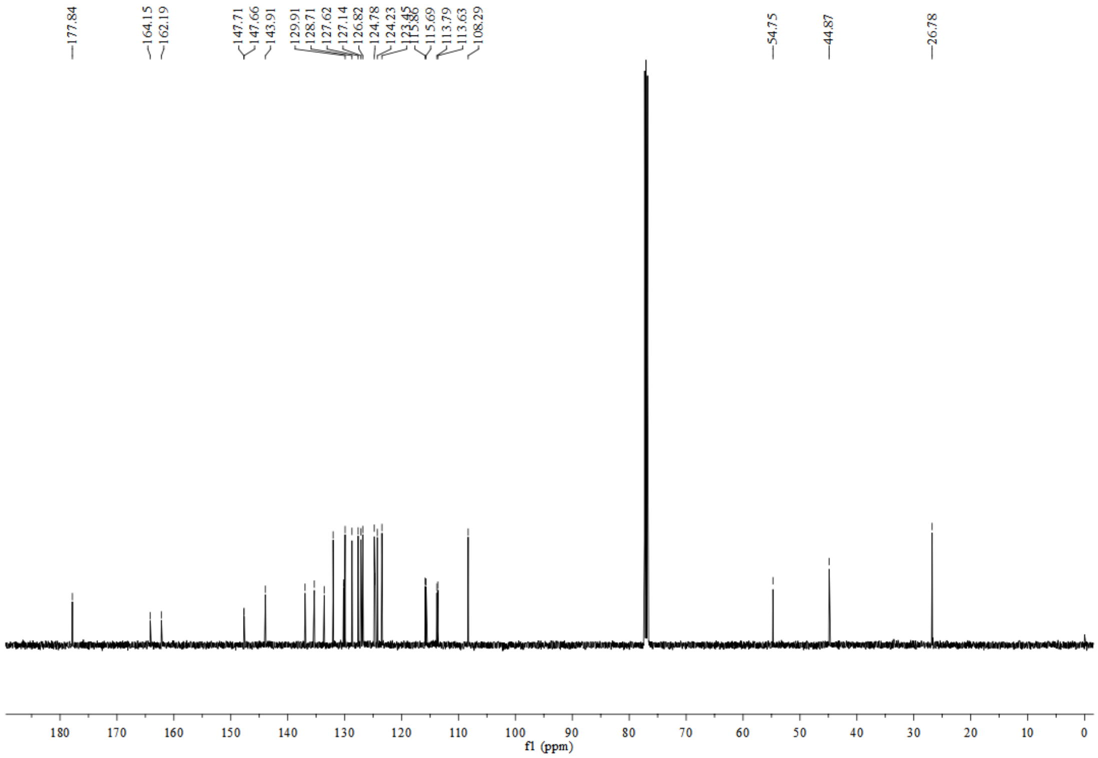

Supplementary Figure 134. ^{13}C NMR Spectra of compound **3ac**.

4'-(3-Chlorophenyl)-1-methyl-4'*H*-spiro[indoline-3,1'-naphthalen]-2-one (**3ad**)

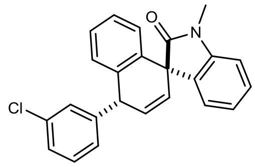

Purified by chromatography on silica gel, eluting with ethyl acetate/petroleum ether 1:15 (*v*/*v*); white solid, Mp = 153-155 °C, 45.3 mg, 61% yield; ^{1}H NMR (500 MHz, $CDCl_3$): δ 7.42-7.37 (m, 1H), 7.35-7.31 (m, 1H), 7.29-7.27 (m, 2H), 7.21 (d, *J* = 7.5 Hz, 1H), 7.16-7.12 (m, 3H), 7.07-7.03 (m, 1H), 7.02-6.98 (m, 2H), 6.61 (d, *J* = 7.5 Hz, 1H), 6.23 (dd, *J* = 9.5, 3.0 Hz, 1H), 5.65 (dd, *J* = 10.0, 2.5 Hz, 1H), 5.00 (t, *J* = 2.0 Hz, 1H), 3.31 (s, 3H). ^{13}C NMR (125 MHz, $CDCl_3$): δ 177.7, 147.1, 143.9, 136.8, 135.2, 134.5, 133.5, 131.9, 129.99, 129.91, 129.0, 128.7, 127.6, 127.17, 127.15, 126.9, 126.8, 124.7, 124.3, 123.4, 108.2, 54.7, 44.8, 26.7. HRMS *m*/*z* (ESI+): Calculated for $C_{24}H_{19}{}^{35}ClNO$ ([M+H]$^+$): 372.1150, found 372.1144.

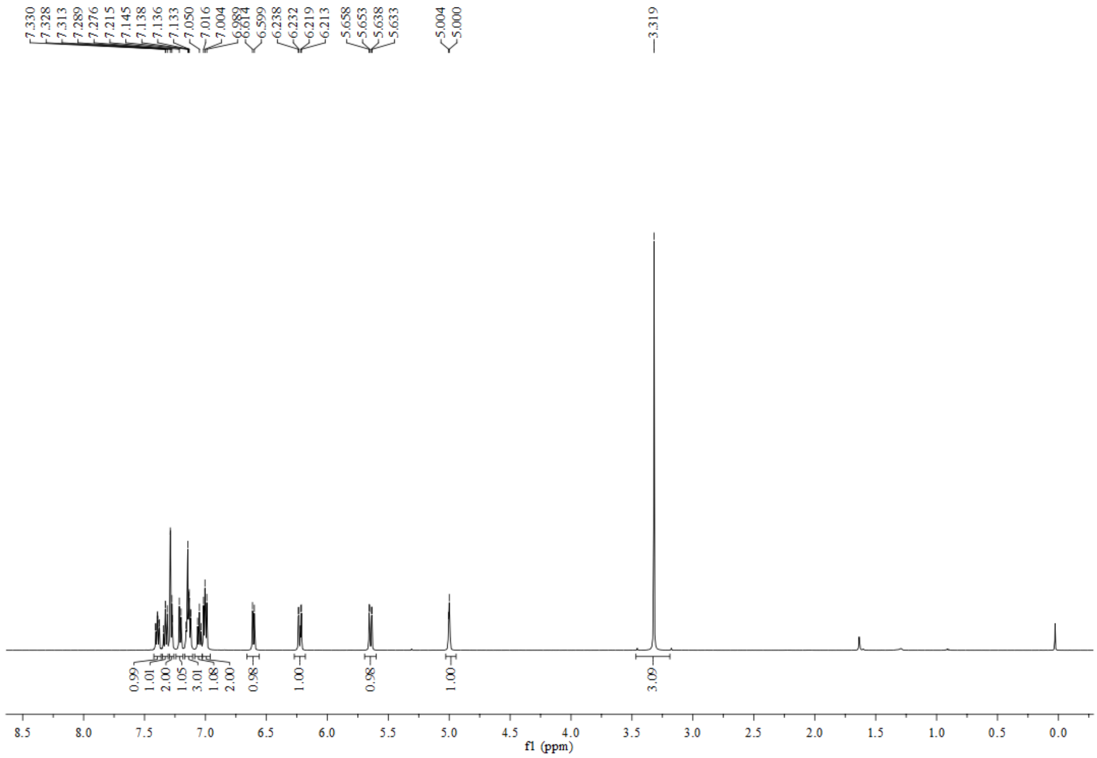

Supplementary Figure 135. ^{1}H NMR Spectra of compound **3ad**.

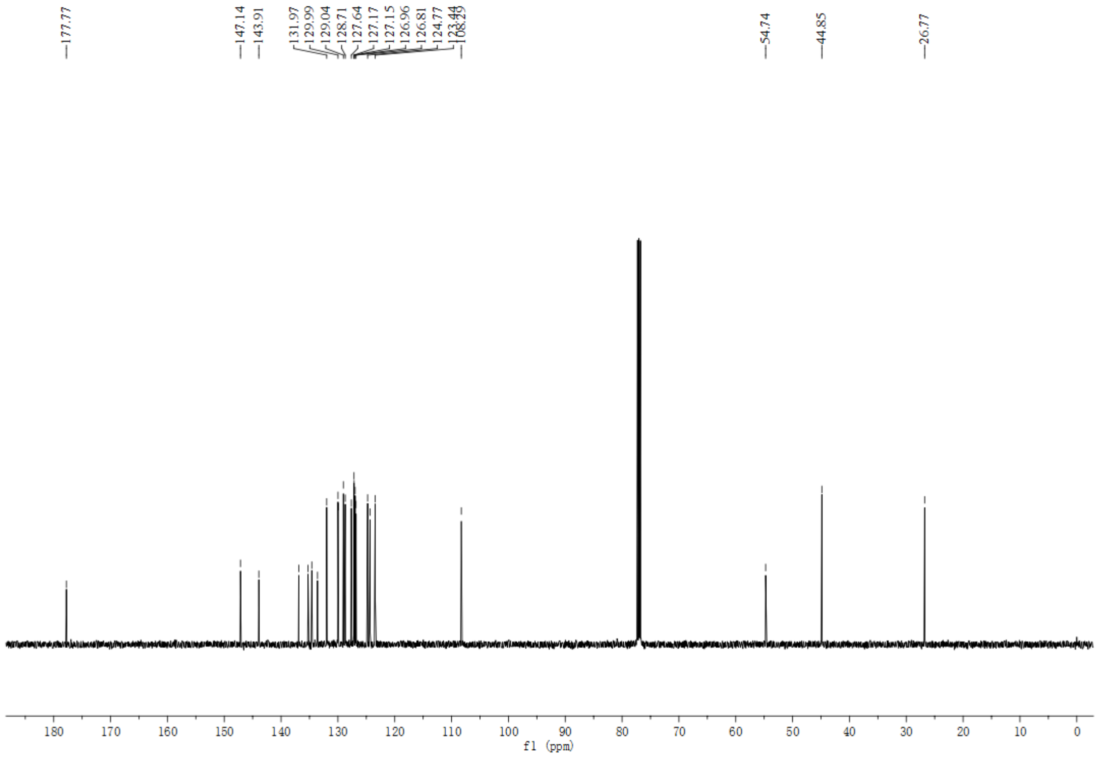

Supplementary Figure 136. ^{13}C NMR Spectra of compound **3ad**.

4'-(4-Fluorophenyl)-1-methyl-4'*H*-spiro[indoline-3,1'-naphthalen]-2-one (**3ae**)

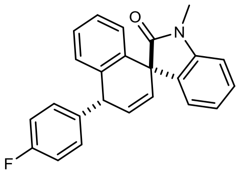

Purified by chromatography on silica gel, eluting with ethyl acetate/petroleum ether 1:15 (*v*/*v*); white solid, Mp = 186-188 °C, 55.5 mg, 78% yield; ^{1}H NMR (500 MHz, $CDCl_3$): δ 7.41-7.36 (m, 1H), 7.27-7.25 (m, 2H), 7.14-7.01 (m, 6H), 6.99 (d, *J* = 7.5 Hz, 2H), 6.59 (dd, *J* = 8.0, 1.0 Hz, 1H), 6.23 (dd, *J* = 9.5, 3.0 Hz, 1H), 5.62 (dd, *J* = 10.0, 2.5 Hz, 1H), 5.01 (t, *J* = 2.5 Hz, 1H), 3.31 (s, 3H). ^{13}C NMR (125 MHz, $CDCl_3$): δ 177.8, 161.7 (d, *J* = 243.8 Hz), 143.9, 140.9 (d, *J* = 2.5 Hz), 137.4, 135.3, 133.5, 132.5, 130.3 (d, *J* =8.8 Hz), 129.9, 128.6, 127.5, 126.9, 126.7, 124.7, 123.9, 123.3, 115.5 (d, *J* = 21.2 Hz), 108.2, 54.7, 44.3, 26.7. HRMS *m*/*z* (ESI+): Calculated for $C_{24}H_{19}FNO$ ($[M+H]^+$): 356.1445, found 356.1443.

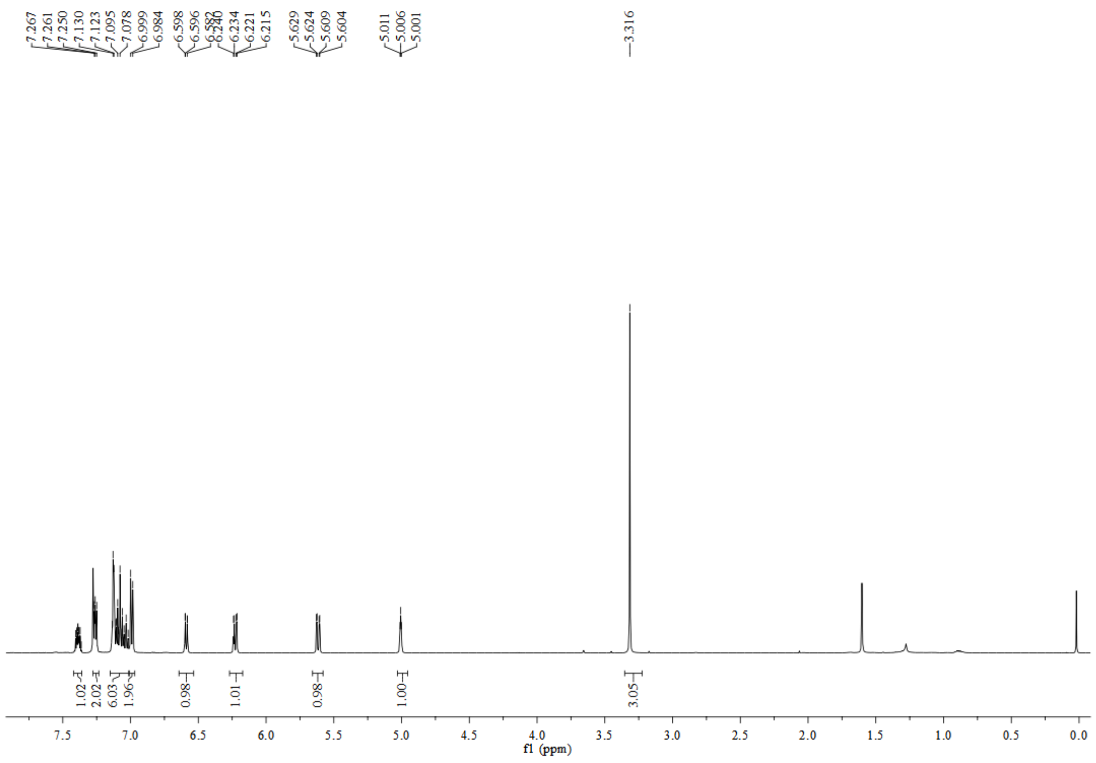

Supplementary Figure 137. ^{1}H NMR Spectra of compound **3ae**.

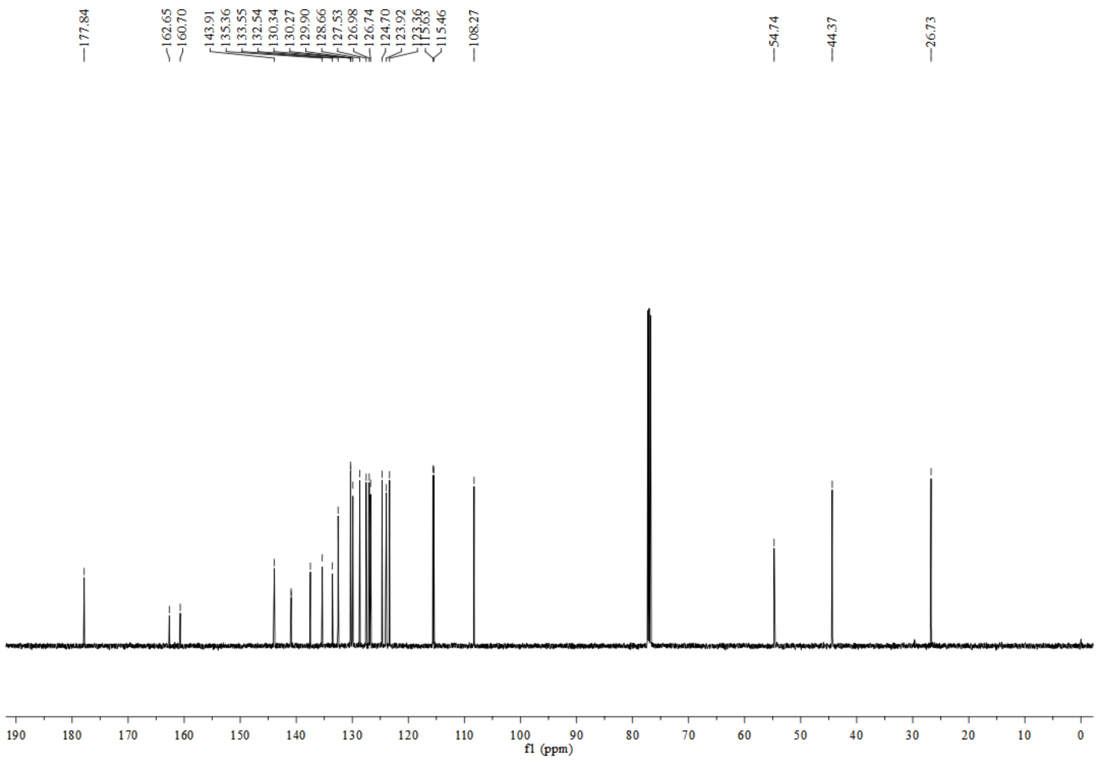

Supplementary Figure 138. ^{13}C NMR Spectra of compound **3ae**.

4'-(4-Chlorophenyl)-1-methyl-4'*H*-spiro[indoline-3,1'-naphthalen]-2-one (**3af**)

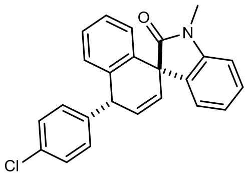

Purified by chromatography on silica gel, eluting with ethyl acetate/petroleum ether 1:15 (*v*/*v*); white solid, Mp = 188-190 °C, 37.1 mg, 50% yield; ^{1}H NMR (600 MHz, $CDCl_3$): δ 7.42-7.36 (m, 3H), 7.25 (d, *J* = 8.4 Hz, 2H), 7.15-7.11 (m, 3H), 7.05 (t, *J* = 7.2 Hz, 1H), 7.01-6.98 (m, 2H), 6.60 (d, *J* = 7.8 Hz, 1H), 6.22 (dd, *J* = 10.2, 3.0 Hz, 1H), 5.63 (dd, *J* = 9.6, 2.4 Hz, 1H), 5.00 (s, 1H), 3.32 (s, 3H). ^{13}C NMR (150 MHz, $CDCl_3$): δ 177.8, 143.9, 143.6, 137.1, 135.3, 133.6, 132.5, 132.2, 130.2, 129.9, 128.9, 128.7, 127.6, 127.1, 126.8, 124.7, 124.1, 123.4, 108.3, 54.7, 44.5, 26.8. HRMS *m*/*z* (ESI+): Calculated for $C_{24}H_{19}{}^{35}ClNO$ ([M+H]$^+$): 372.1150, found 372.1145.

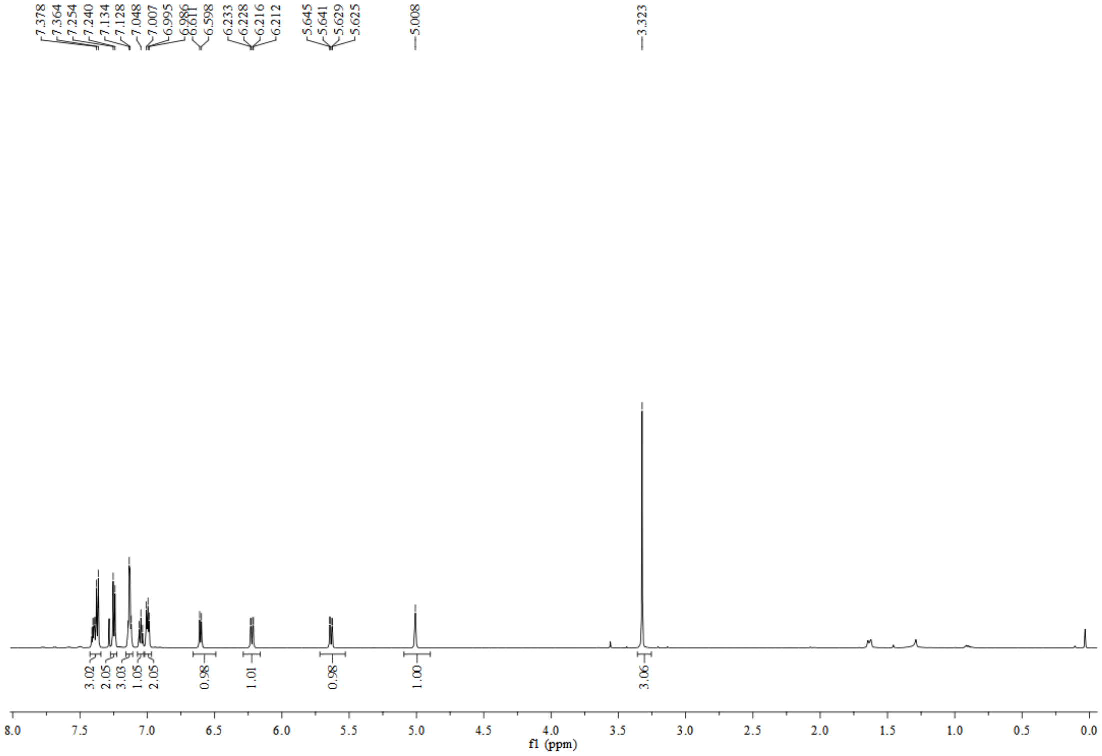

Supplementary Figure 139. ^{1}H NMR Spectra of compound **3af**.

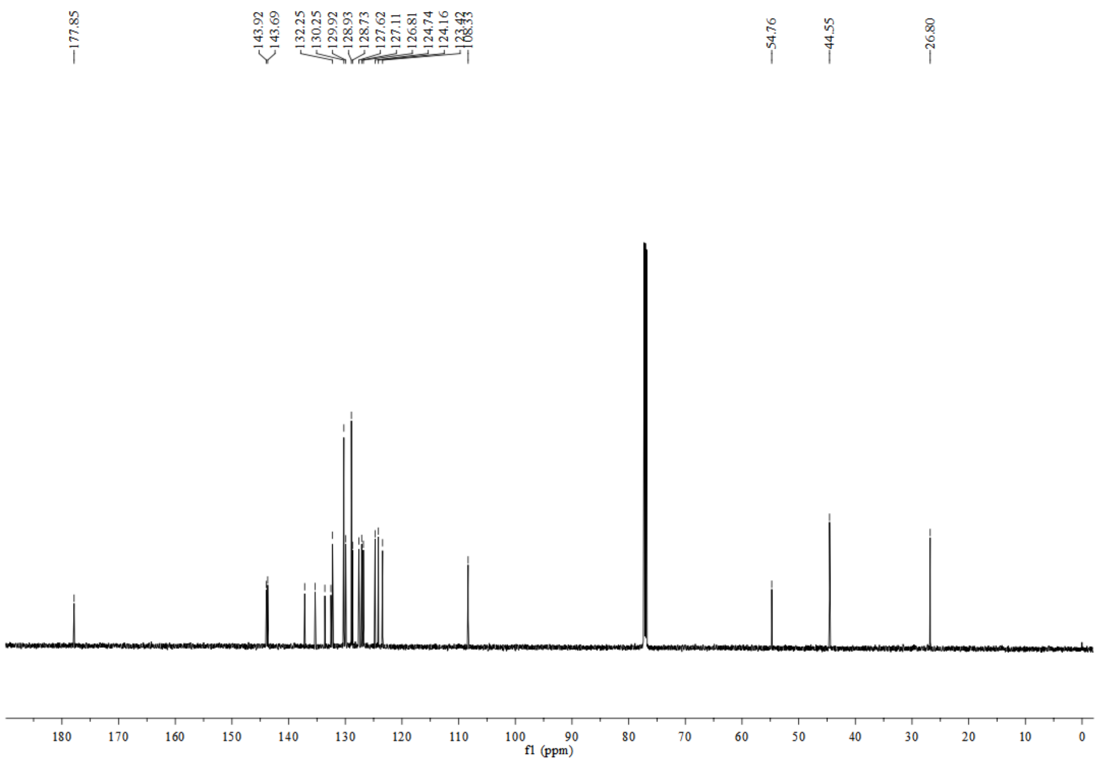

Supplementary Figure 140. ^{13}C NMR Spectra of compound **3af**.

1-Methyl-4'-(4-(trifluoromethyl)phenyl)-4'*H*-spiro[indoline-3,1'-naphthalen]-2-one (**3ag**)

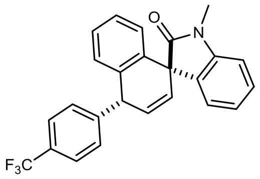

Purified by chromatography on silica gel, eluting with ethyl acetate/petroleum ether 1:15 (*v*/*v*); white solid, Mp = 161-163 °C, 44.3 mg, 55% yield; ^{1}H NMR (600 MHz, $CDCl_3$): δ 7.67 (d, *J* = 7.8 Hz, 2H), 7.45-7.40 (m, 3H), 7.15-7.13 (m, 3H), 7.07 (t, *J* = 7.8 Hz, 1H), 7.01 (d, *J* = 7.8 Hz, 1H), 6.97 (d, *J* = 7.8 Hz, 1H), 6.63 (d, *J* = 7.8 Hz, 1H), 6.23 (dd, *J* = 9.6, 3.0 Hz, 1H), 5.67 (dd, *J* = 9.6, 1.8 Hz, 1H), 5.10 (s, 1H), 3.33 (s, 3H). ^{13}C NMR (150 MHz, $CDCl_3$): δ 177.7, 149.1, 143.9, 136.7, 135.2, 133.7, 131.8, 129.9, 129.2, 129.1 (q, *J* = 33.0 Hz), 128.8, 127.7, 127.2, 126.9, 125.8 (q, *J* = 3.0 Hz), 124.7, 124.5, 124.2 (q, *J* = 271.5 Hz), 123.4, 108.3, 54.7, 45.0, 26.8. HRMS *m*/*z* (ESI+): Calculated for $C_{25}H_{19}F_3NO$ ($[M+H]^+$): 406.1413, found 406.1408.

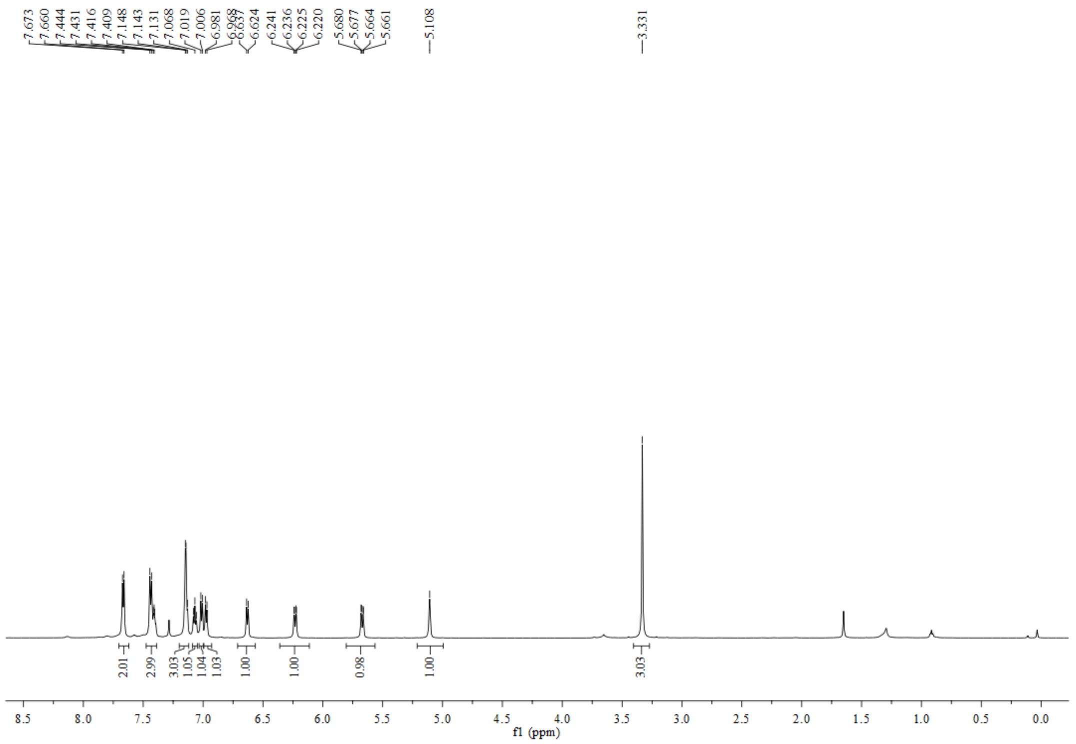

Supplementary Figure 141. ^{1}H NMR Spectra of compound **3ag**.

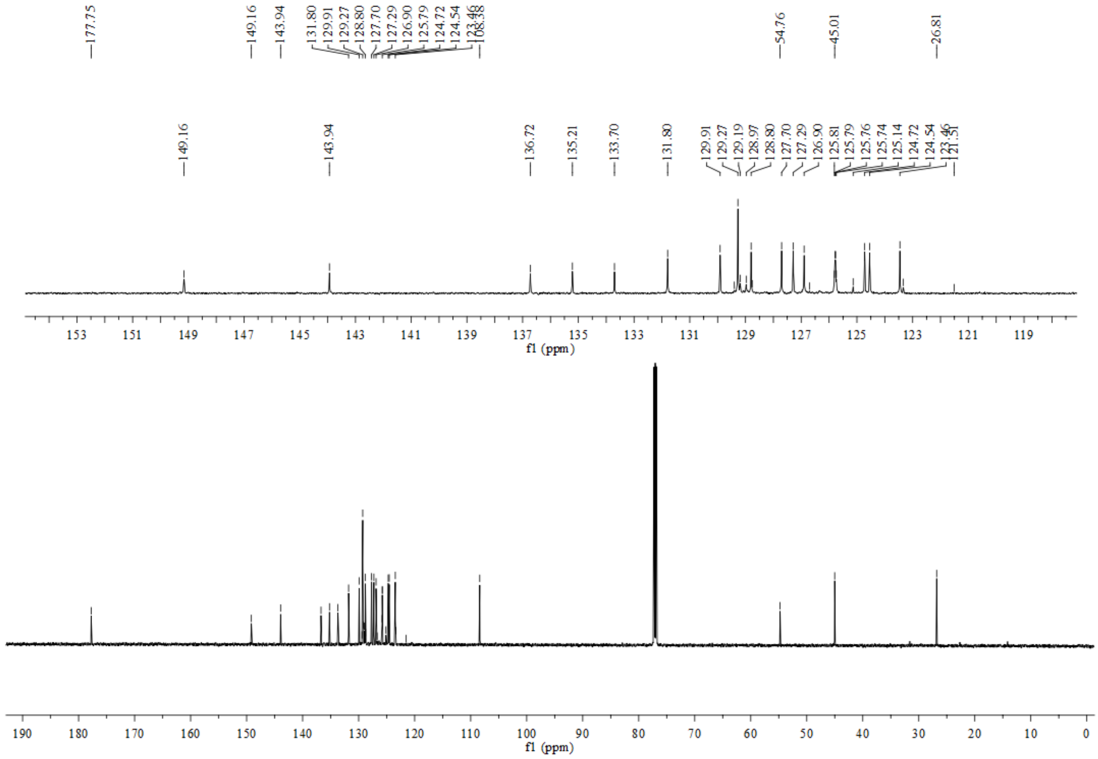

Supplementary Figure 142. ^{13}C NMR Spectra of compound **3ag**.

4'-([1,1'-Biphenyl]-4-yl)-1-methyl-4'*H*-spiro[indoline-3,1'-naphthalen]-2-one (**3ah**)

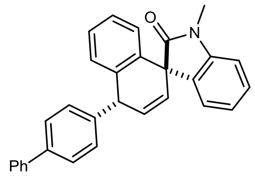

Purified by chromatography on silica gel, eluting with ethyl acetate/petroleum ether 1:15 (*v*/*v*); white solid, Mp = 180-182 °C, 41.2 mg, 50% yield; ^{1}H NMR (500 MHz, $CDCl_3$): δ 7.65-7.61 (m, 4H), 7.49-7.45 (m, 2H), 7.41-7.35 (m, 4H), 7.20-7.17 (m, 1H), 7.17-7.09 (m, 3H), 7.07-7.03 (m, 1H), 7.00 (d, *J* = 8.0 Hz, 1H), 6.62 (dd, *J* = 8.0, 1.0 Hz, 1H), 6.30 (dd, *J* = 10.0, 3.5 Hz, 1H), 5.64 (dd, *J* = 10.0, 2.5 Hz, 1H), 5.07 (t, *J* = 3.0 Hz, 1H), 3.33 (s, 3H). ^{13}C NMR (125 MHz, $CDCl_3$): δ 178.0, 144.2, 143.9, 140.8, 139.6, 137.5, 135.5, 133.6, 132.5, 130.1, 129.3, 128.7, 128.6, 127.57, 127.52, 127.2, 127.0, 126.9, 126.7, 124.8, 123.7, 123.3, 108.2, 54.8, 44.8, 26.7. HRMS *m*/*z* (ESI+): Calculated for $C_{30}H_{24}NO$ ([M+H]$^{+}$): 414.1852, found 414.1847.

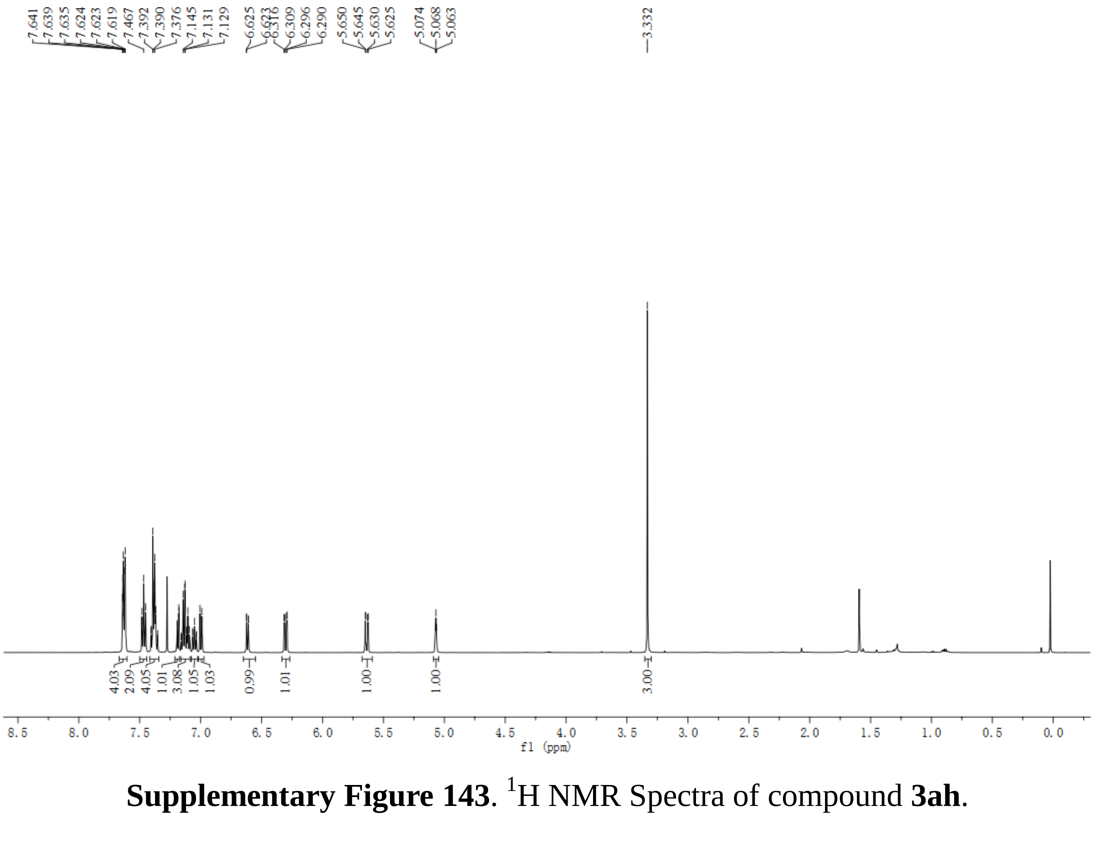

Supplementary Figure 143. ^{1}H NMR Spectra of compound **3ah**.

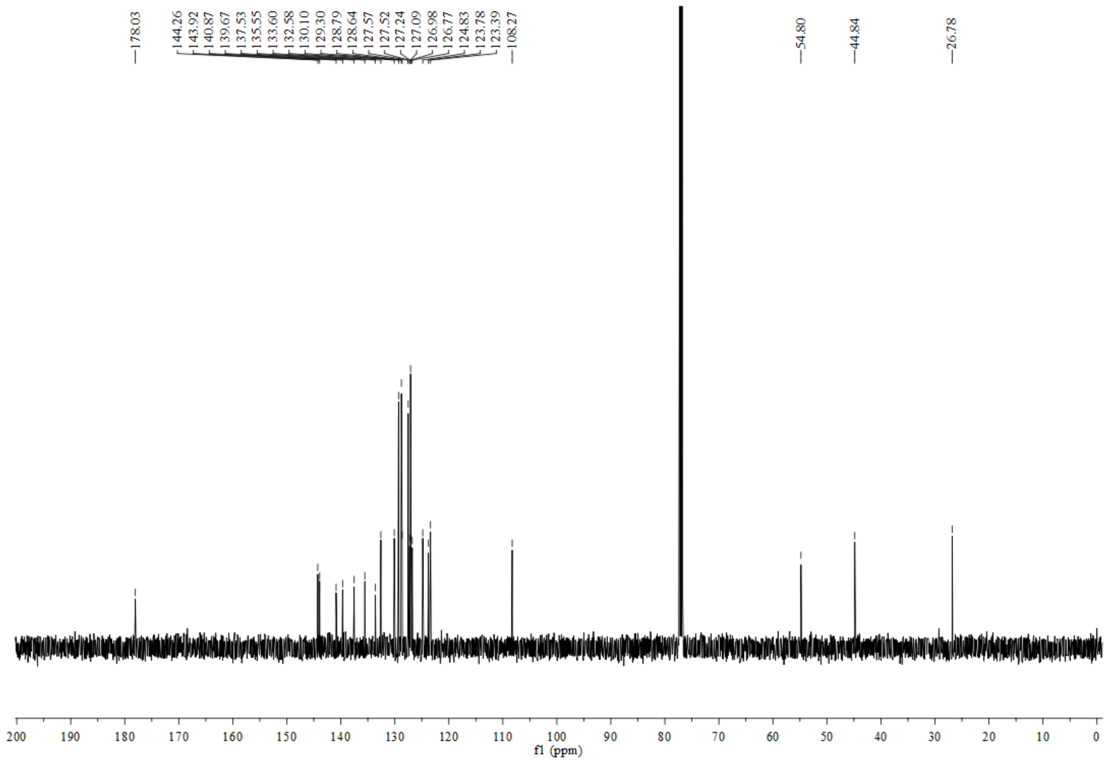

Supplementary Figure 144. ^{13}C NMR Spectra of compound **3ah**.

1-Methyl-4'-(4-vinylphenyl)-4'*H*-spiro[indoline-3,1'-naphthalen]-2-one (**3ai**)

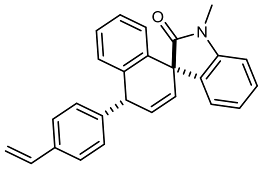

Purified by chromatography on silica gel, eluting with ethyl acetate/petroleum ether 1:15 (*v*/*v*); white solid, Mp = 171-173 °C, 33.6 mg, 46% yield; ^{1}H NMR (500 MHz, $CDCl_3$): δ 7.44 (d, *J* = 8.5 Hz, 2H), 7.40-7.36 (m, 1H), 7.28-7.26 (m, 2H), 7.17-7.09 (m, 3H), 7.05-6.98 (m, 3H), 6.78-6.72 (m, 1H), 6.61-6.58 (m, 1H), 6.25 (dd, *J* = 10.0, 3.0 Hz, 1H), 5.79-5.75 (m, 1H), 5.61 (dd, *J* = 10.0, 2.5 Hz, 1H), 5.28-5.25 (m, 1H), 5.01 (t, *J* = 2.5 Hz, 1H), 3.32 (s, 3H). ^{13}C NMR (125 MHz, $CDCl_3$): δ 178.0, 144.8, 143.9, 137.4, 136.5, 136.1, 135.5, 133.5, 132.5, 130.0, 129.0, 128.6, 127.5, 126.9, 126.7, 126.6, 124.8, 123.7, 123.3, 113.6, 108.2, 54.7, 44.8, 26.7. HRMS *m*/*z* (ESI+): Calculated for $C_{26}H_{22}NO$ ($[M+H]^+$): 364.1696, found 364.1707.

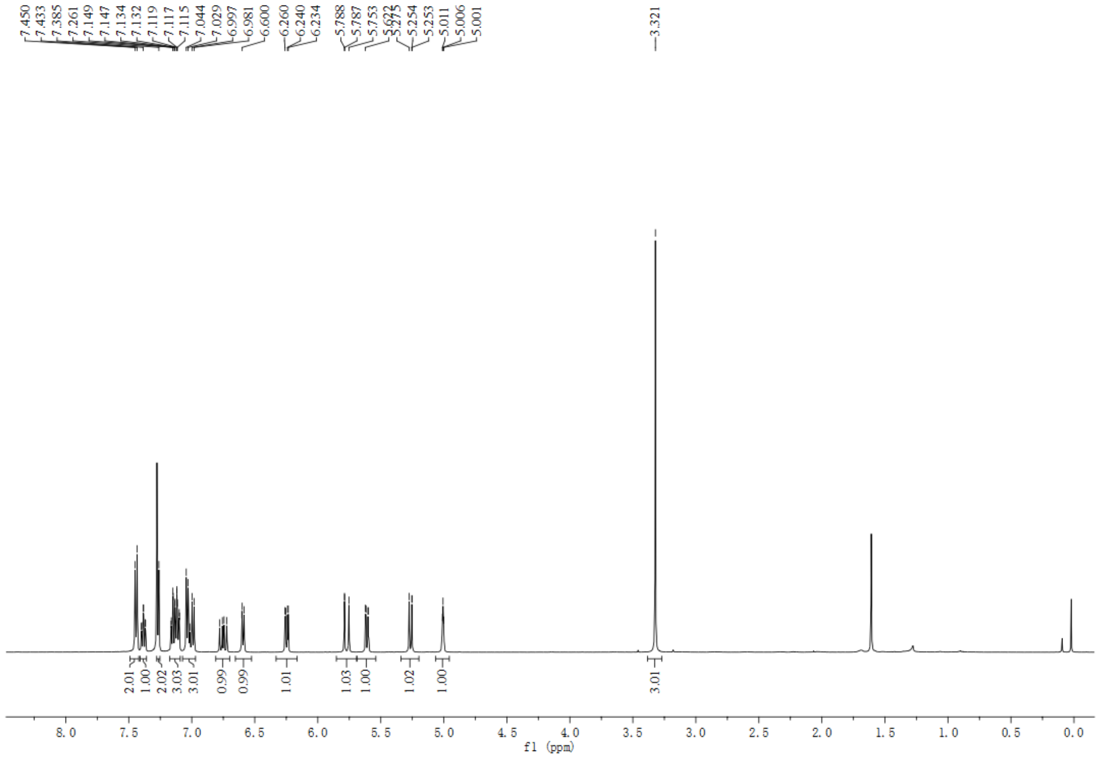

Supplementary Figure 145. ^{1}H NMR Spectra of compound **3ai**.

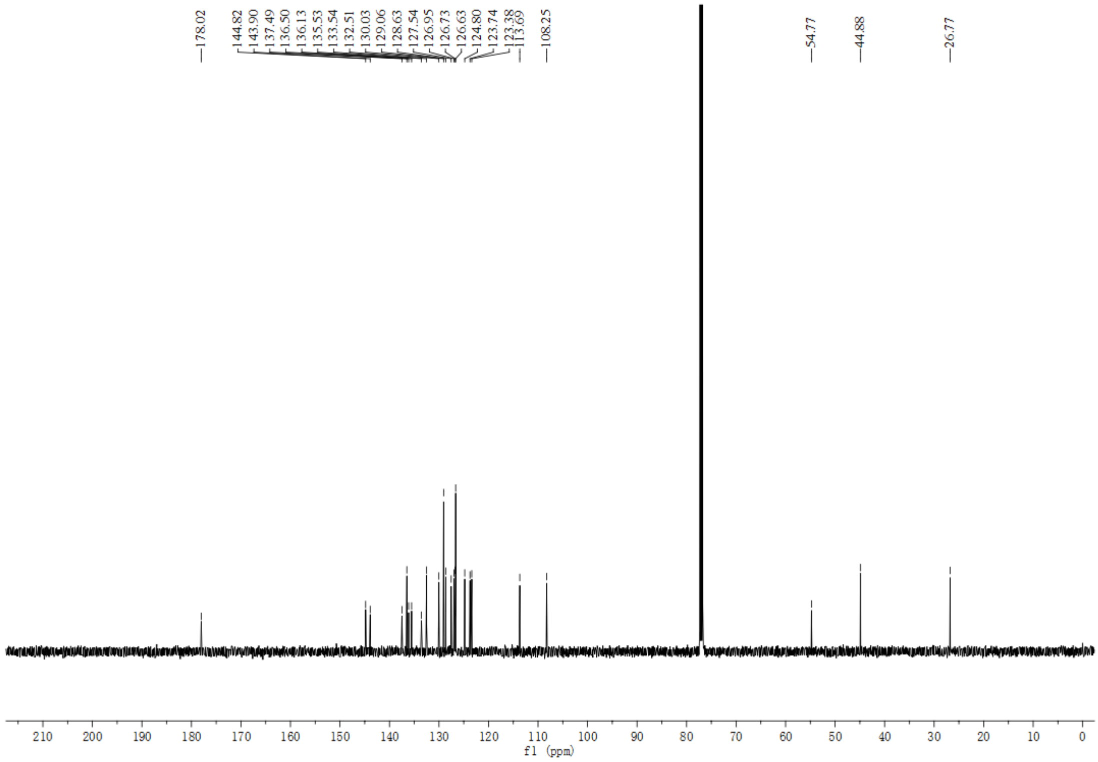

Supplementary Figure 146. ^{13}C NMR Spectra of compound **3ai**.

4'-(3,4-Difluorophenyl)-1-methyl-4'*H*-spiro[indoline-3,1'-naphthalen]-2-one (**3aj**)

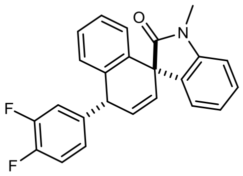

Purified by chromatography on silica gel, eluting with ethyl acetate/petroleum ether 1:15 (*v*/*v*); white solid, Mp = 150-152 °C, 67.4 mg, 90% yield; ^{1}H NMR (500 MHz, $CDCl_3$): δ 7.42-7.38 (m, 1H), 7.21-7.10 (m, 4H), 7.09-7.03 (m, 3H), 7.01-6.97 (m, 2H), 6.60 (d, *J* = 7.0 Hz, 1H), 6.20 (dd, *J* = 10.0, 3.0 Hz, 1H), 5.64 (dd, *J* = 10.0, 2.5 Hz, 1H), 4.98 (s, 1H), 3.31 (s, 3H). ^{13}C NMR (125 MHz, $CDCl_3$): δ 177.6, 150.5 (dd, *J* = 246.3, 12.5 Hz), 149.3 (dd, *J* = 246.3, 12.5 Hz), 143.9, 142.2 (dd, *J* = 3.8 Hz, 3.8 Hz), 136.7, 135.1, 133.5, 131.8, 129.7, 128.7, 127.6, 127.2, 126.8, 124.7 (dd, *J* = 6.3, 3.8 Hz), 124.6, 124.5, 123.4, 117.5 (dd, *J* = 35.0, 17.5 Hz), 108.3, 54.7, 44.3, 26.7. HRMS *m*/*z* (ESI+): Calculated for $C_{24}H_{18}F_2NO$ ([M+H]$^+$): 374.1351, found 374.1345.

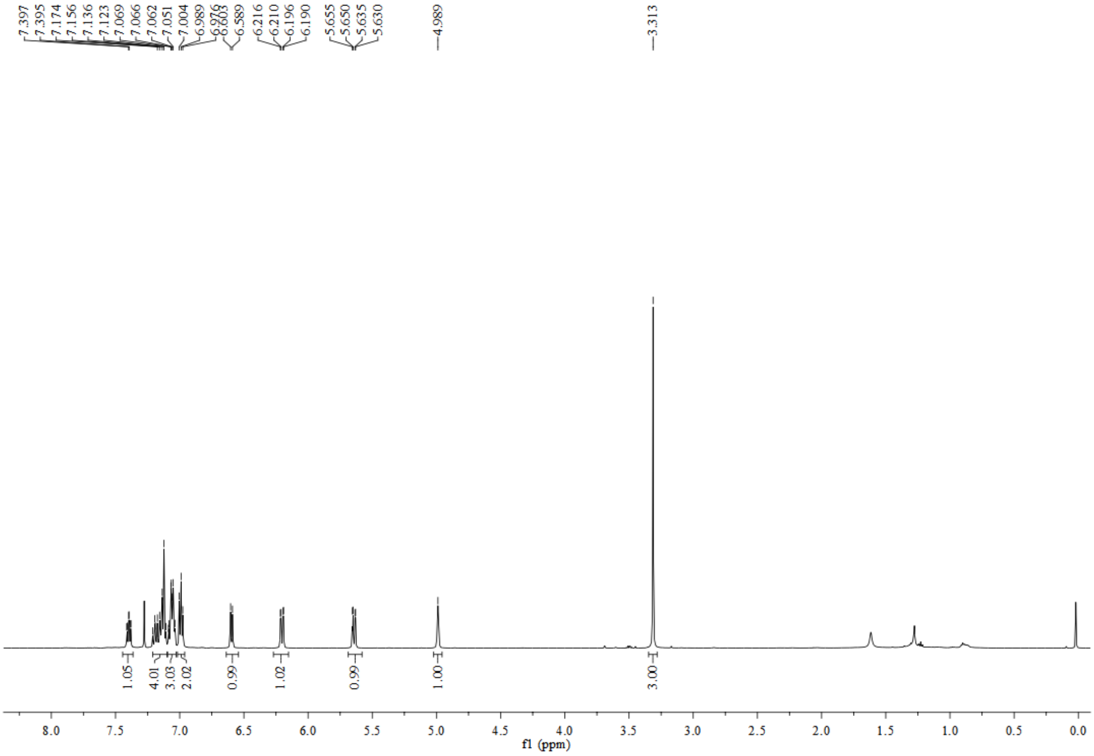

Supplementary Figure 147. ^{1}H NMR Spectra of compound **3aj**.

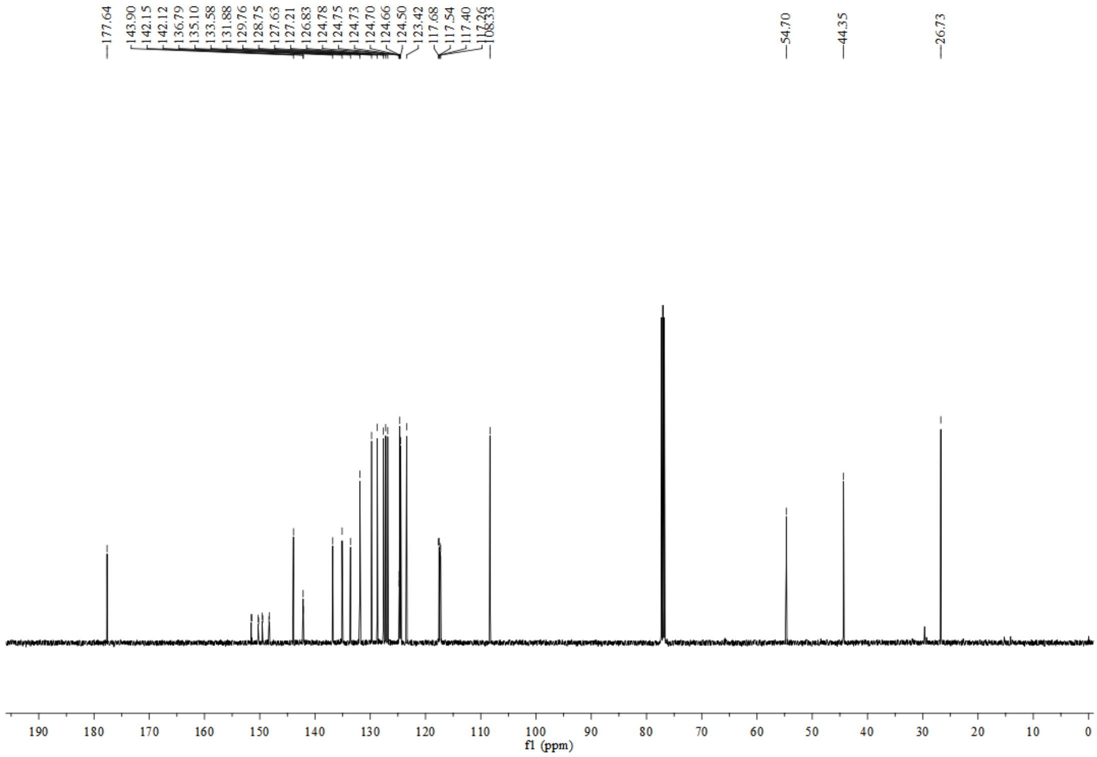

Supplementary Figure 148. ^{13}C NMR Spectra of compound **3aj**.

4'-(3-Chloro-4-fluorophenyl)-1-methyl-4'*H*-spiro[indoline-3,1'-naphthalen]-2-one (**3ak**)

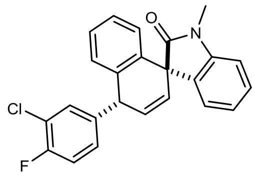

Purified by chromatography on silica gel, eluting with ethyl acetate/petroleum ether 1:15 (*v*/*v*); white solid, Mp = 160-162 ^{o}C, 49.0 mg, 63% yield; ^{1}H NMR (500 MHz, $CDCl_3$): δ 7.42-7.38 (m, 1H), 7.34-7.31 (m, 1H), 7.18-7.11 (m, 5H), 7.07-7.04 (m, 1H), 7.01-6.97 (m, 2H), 6.60 (d, *J* = 7.5 Hz, 1H), 6.20 (dd, *J* = 10.0, 3.0 Hz, 1H), 5.65 (dd, *J* = 10.0, 2.5 Hz, 1H), 4.98 (s, 1H), 3.31 (s, 3H). ^{13}C NMR (125 MHz, $CDCl_3$): δ 177.6, 157.0 (d, *J* = 246.3 Hz), 143.9, 142.2 (d, *J* = 3.8 Hz), 136.8, 135.1, 133.6, 131.9, 130.9, 129.8, 128.7, 128.5 (d, *J* =6.3 Hz), 127.7, 127.2, 126.8, 124.7 (d, *J* = 12.5 Hz), 123.4, 121.2 (d, *J* = 18.8 Hz), 116.8 (d, *J* = 21.3 Hz), 108.3, 54.7, 44.2, 26.7. HRMS *m*/*z* (ESI+): Calculated for $C_{24}H_{18}F^{35}ClNO$ ($[M+H]^+$): 390.1055, found 390.1051.

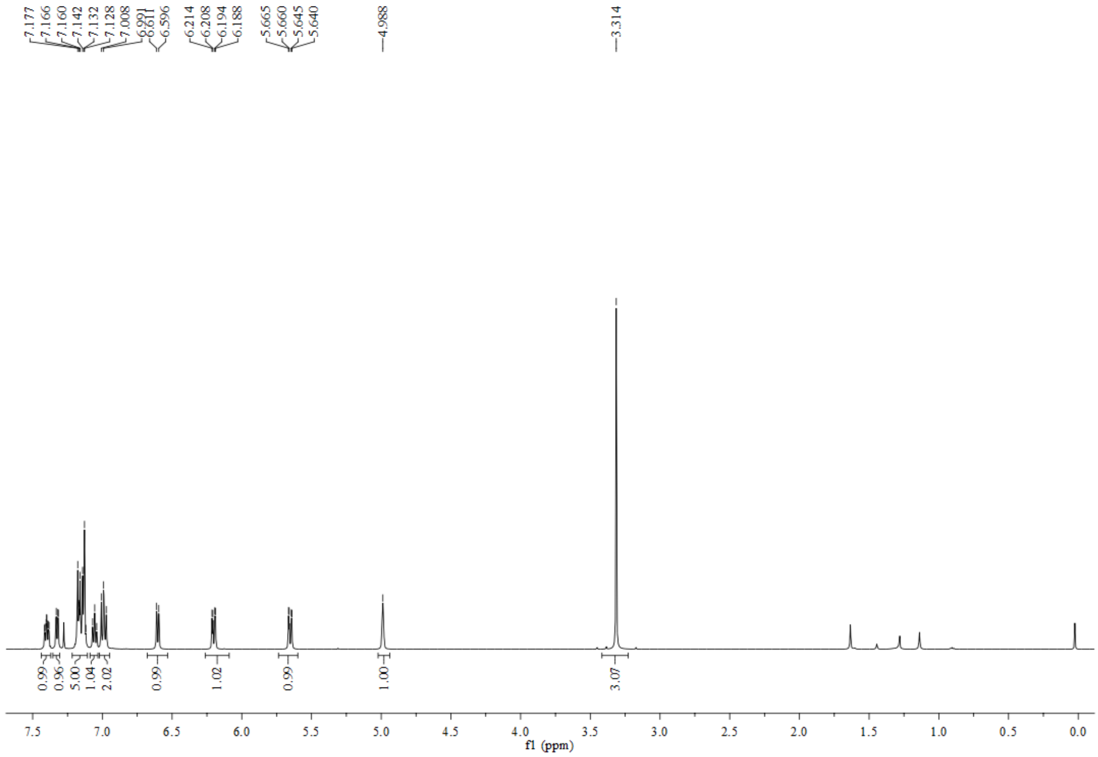

Supplementary Figure 149. ¹H NMR Spectra of compound **3ak**.

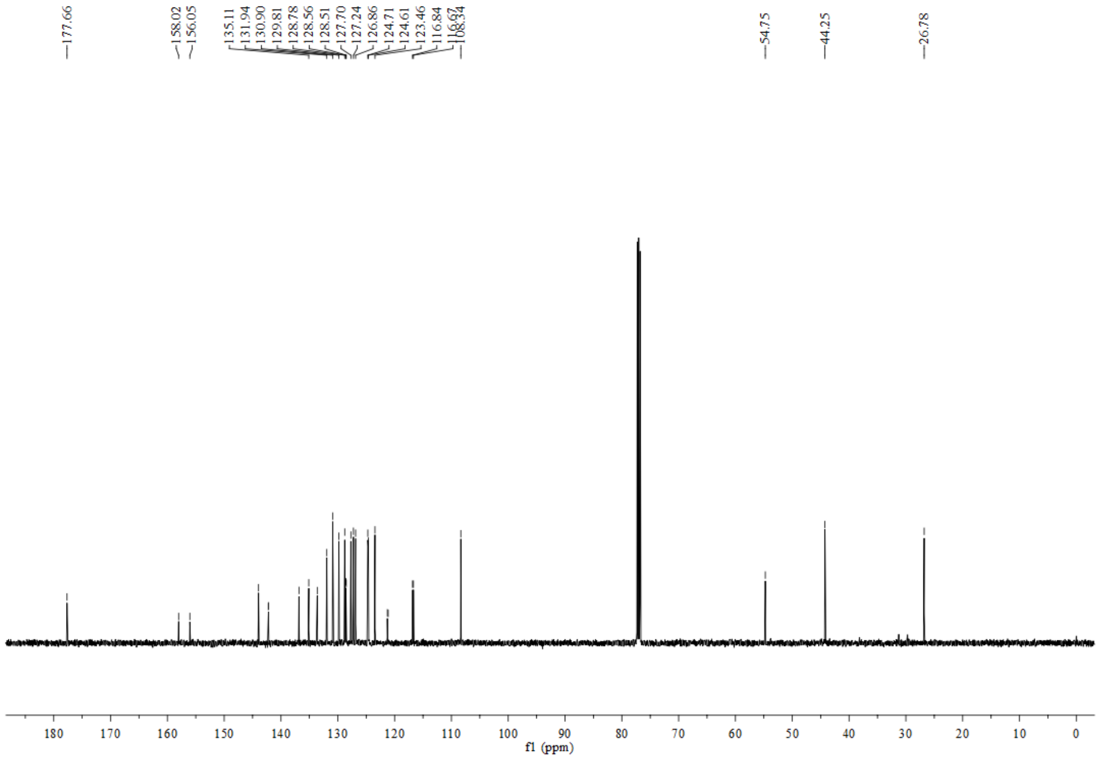

Supplementary Figure 150. ¹³C NMR Spectra of compound **3ak**.

Methyl 4'-phenyl-2*H*,4'*H*-spiro[benzofuran-3,1'-naphthalene]-6-carboxylate (**12**)

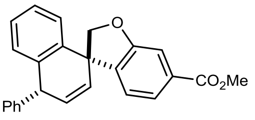

Purified by chromatography on silica gel, eluting with ethyl acetate/petroleum ether 1:30 (*v*/*v*); colourless oil, 40.7 mg, 55% yield; ^{1}H NMR (500 MHz, $CDCl_3$): δ 7.66-7.62 (m, 2H), 7.40-7.7 (m, 2H), 7.31-7.25 (m, 3H), 7.16-7.10 (m, 3H), 7.07 (d, *J* = 7.5 Hz, 1H), 7.02 (d, *J* = 7.5 Hz, 1H), 6.08-6.05 (m, 1H), 5.90-5.87 (m, 1H), 4.79 (d, *J* = 9.0 Hz, 1H), 4.75 (t, *J* = 2.5 Hz, 1H), 4.68 (d, *J* = 9.0 Hz, 1H), 3.93 (s, 3H). ^{13}C NMR (125 MHz, $CDCl_3$): δ 166.8, 159.6, 145.1, 141.1, 138.6, 135.8, 131.0, 129.5, 129.4, 128.8, 128.6, 127.73, 127.70, 127.4, 127.0, 126.7, 124.5, 123.3, 111.0, 86.6, 52.1, 50.2, 45.1. HRMS *m*/*z* (ESI+): Calculated for $C_{25}H_{20}NaO_3$ ($[M+Na]^+$): 391.1305, found 391.1315.

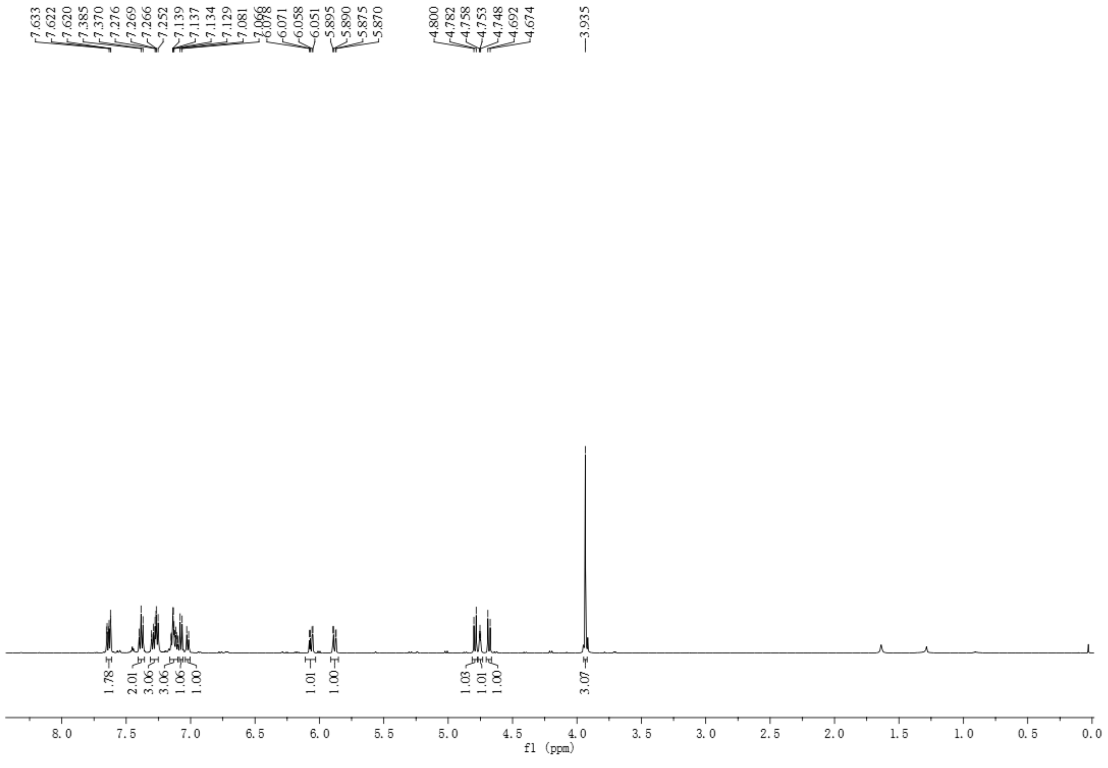

Supplementary Figure 151. ^{1}H NMR Spectra of compound **12**.

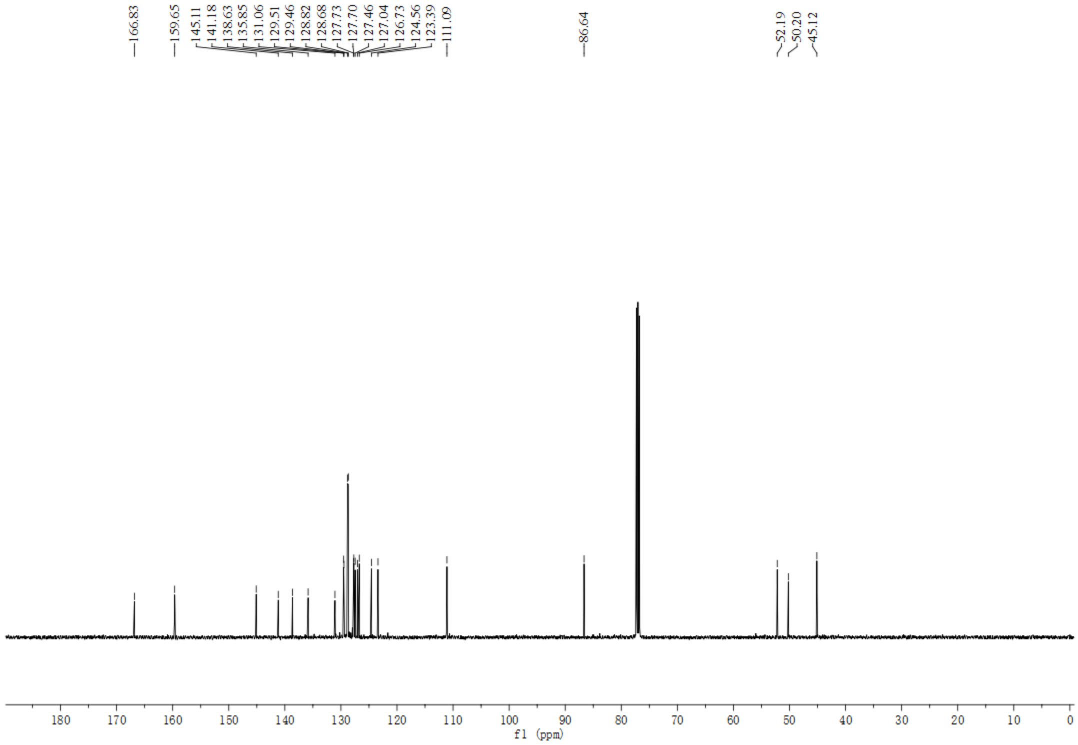

Supplementary Figure 152. ^{13}C NMR Spectra of compound **12**.

General procedure for three-component dearomative 1,4-vinylarylation reaction

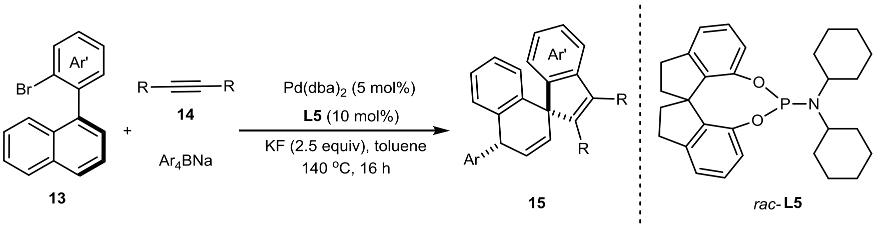

To a dried Schlenk tube were added **13** (0.20 mmol) and **14** (0.30 mmol), $Pd(dba)_2$ (5.8 mg, 0.010 mmol), ligand **L5** (9.2 mg, 0.02 mmol), Ar_4BNa (0.20 mmol), KF (29.1 mg, 0.50 mmol) under N_2. 2.0 mL toluene was then introduced via syringe and the tube was sealed using Teflon cap. The mixture was stirred at 140 °C for 16 h. The solvent was then removed under vacuum and the residue was purified by chromatography on silica gel, eluting with dichloromethane/petroleum ether (*v*/*v*) to afford the products **15**.

2,3-Diethyl-4'-phenyl-4'*H*-spiro[indene-1,1'-naphthalene] (**15a**)

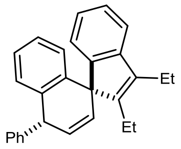

Purified by chromatography on silica gel, eluting with dichloromethane/petroleum ether 1:25 (*v*/*v*); colourless oil, 46.6 mg, 64% yield; ^1H NMR (600 MHz, $CDCl_3$): δ 7.43-7.40 (m, 2H), 7.39-7.35 (m, 3H), 7.34-7.30 (m, 2H), 7.15-7.08 (m, 4H), 6.98-6.95 (m, 1H), 6.59 (d, *J* = 7.8 Hz, 1H), 6.17 (dd, *J* = 9.6, 2.4 Hz, 1H), 5.35 (d, *J* = 9.6 Hz, 1H), 5.00 (s, 1H), 2.75-2.68 (m, 2H), 2.49-2.43 (m, 1H), 2.36-2.30 (m, 1H), 1.38 (t, *J* = 7.2 Hz, 3H), 1.06 (t, *J* = 7.8 Hz, 3H). ^{13}C NMR (150 MHz, $CDCl_3$): δ 154.3, 152.2, 146.2, 144.5, 139.7, 137.3, 135.2, 130.0, 129.5, 128.8, 128.7, 127.8, 127.1, 126.8, 126.55, 126.51, 126.4, 125.1, 123.8, 118.6, 59.2, 45.4, 19.7, 18.8, 14.9, 13.9. HRMS *m*/*z* (EI+): Calculated for $C_{28}H_{26}$ (M^+): 362.2035, found 362.2032.

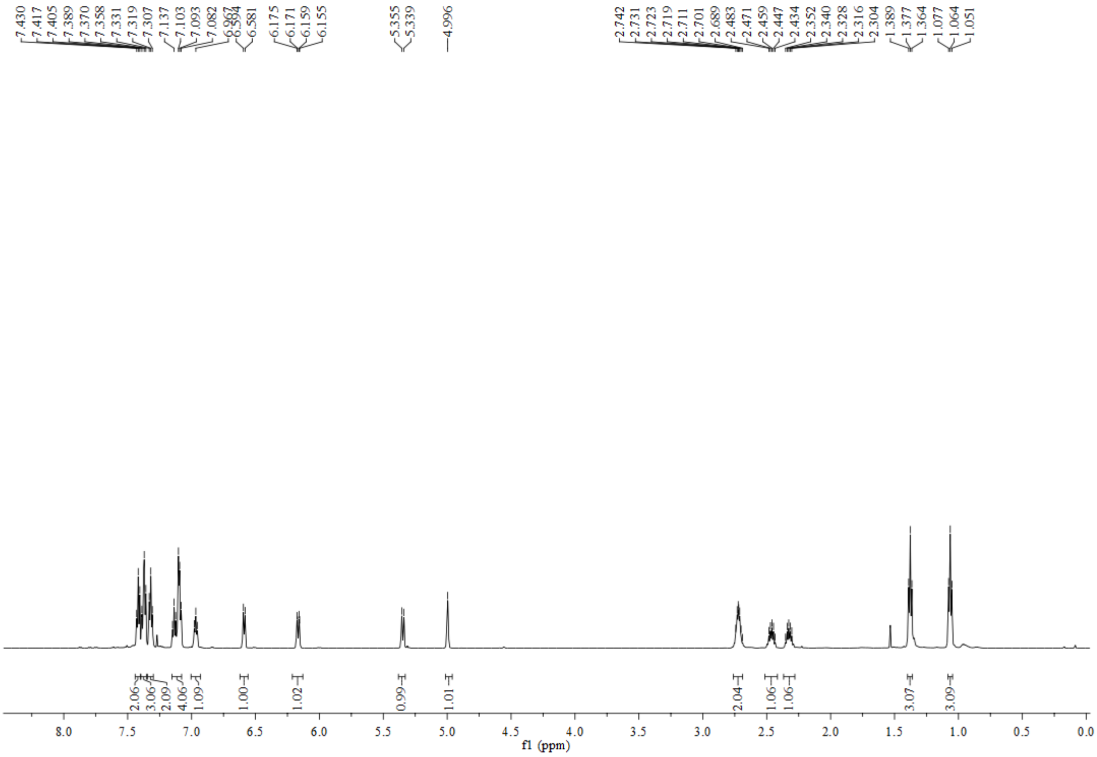

Supplementary Figure 153. ^{1}H NMR Spectra of compound **15a**.

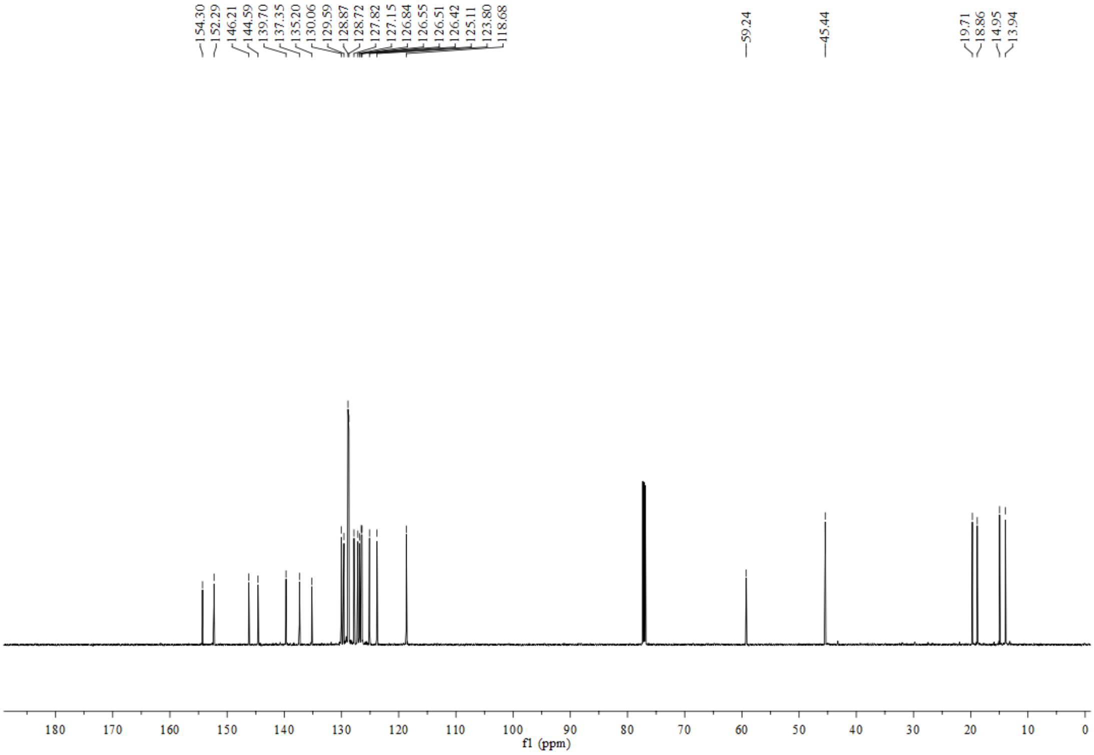

Supplementary Figure 154. ^{13}C NMR Spectra of compound **15a**.

4'-Phenyl-2,3-dipropyl-4'*H*-spiro[indene-1,1'-naphthalene] (**15b**)

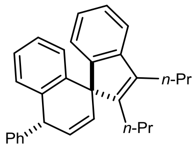

Purified by chromatography on silica gel, eluting with dichloromethane/petroleum ether 1:25 (*v*/*v*); colourless oil, Mp = 85-87 °C, 55.1 mg, 71% yield; ^{1}H NMR (500 MHz, $CDCl_3$): δ 7.41-7.27 (m, 7H), 7.11-7.05 (m, 4H), 6.95 (d, *J* = 6.0 Hz, 1H), 6.54 (d, *J* = 7.0 Hz, 1H), 6.13 (d, *J* = 10.0 Hz, 1H), 5.31-5.28 (m, 1H), 4.96 (s, 1H), 2.65 (s, 2H), 2.44-2.38 (m, 1H), 2.26-2.20 (m, 1H), 1.79 (d, *J* = 5.5 Hz, 2H), 1.49-1.46 (m, 1H), 1.35-1.32 (m, 1H), 1.13-1.10 (m, 3H), 0.93-0.90 (m, 3H). ^{13}C NMR (125 MHz, $CDCl_3$): δ 154.2, 151.5, 146.1, 144.7, 138.4, 137.2, 135.2, 130.0, 129.3, 128.7, 128.6, 127.8, 127.1, 126.7, 126.48, 126.42, 126.3, 125.0, 123.7, 118.8, 59.2, 45.3, 29.2, 27.7, 23.2, 22.2, 14.6, 14.4. HRMS *m*/*z* (EI+): Calculated for $C_{30}H_{30}$ (M^+): 390.2348, found 390.2346.

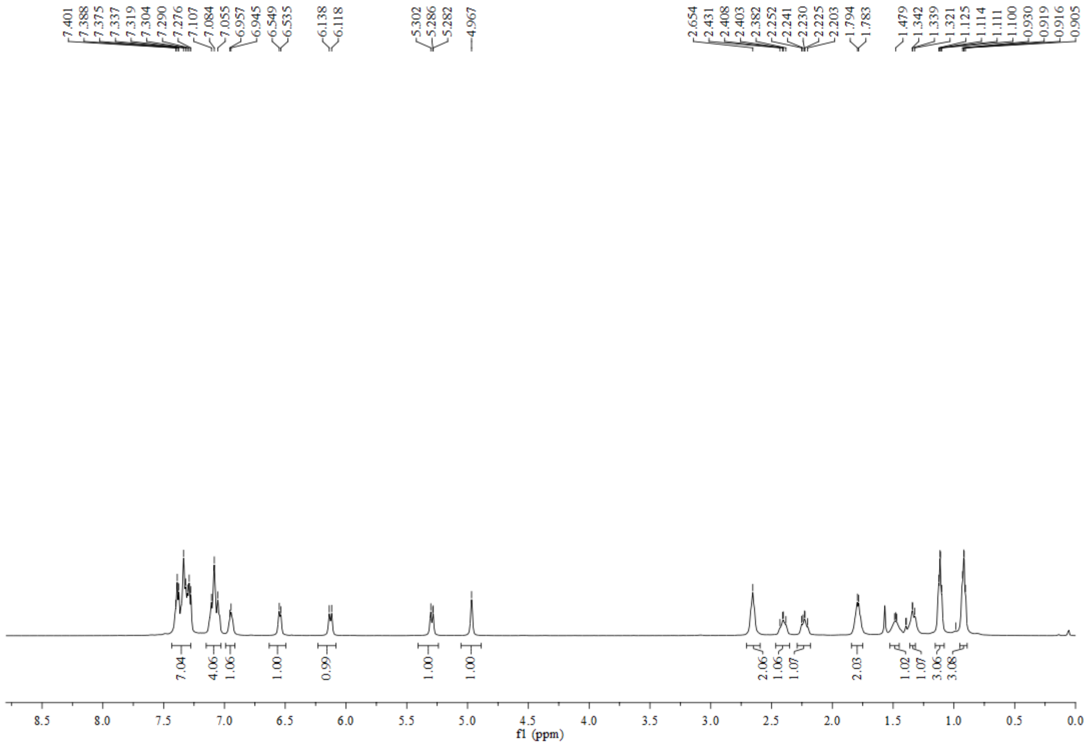

Supplementary Figure 155. ^{1}H NMR Spectra of compound **15b**.

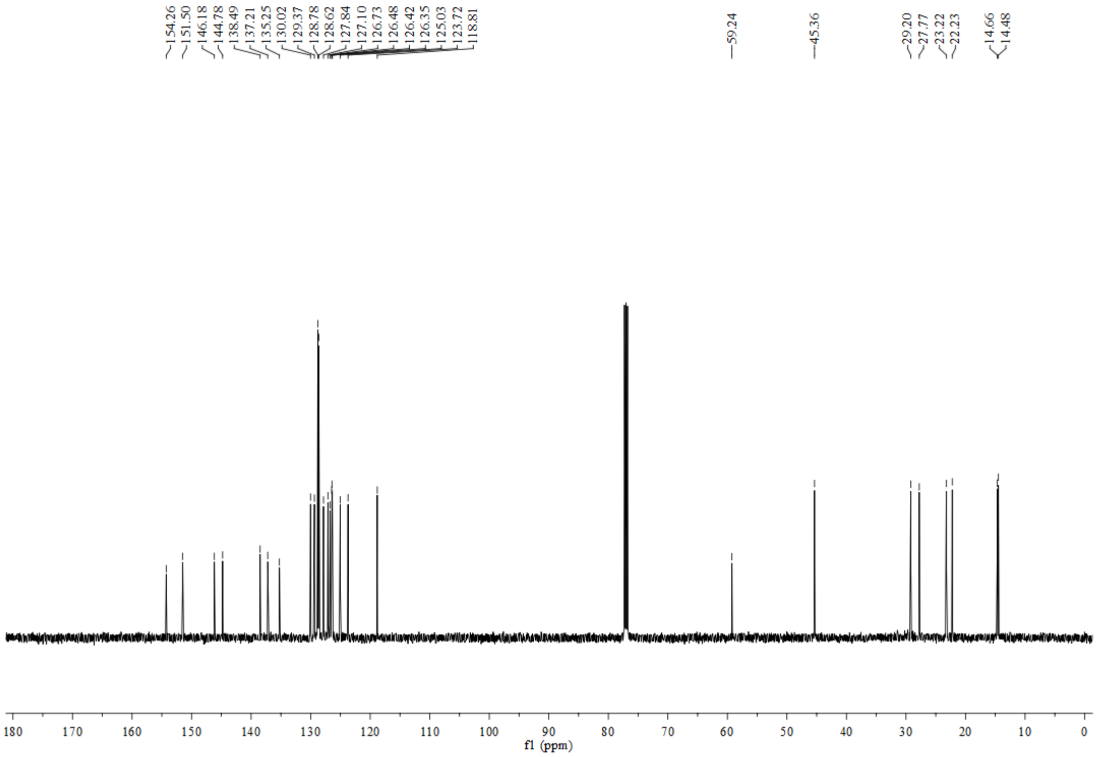

Supplementary Figure 156. ^{13}C NMR Spectra of compound **15b**.

2,3-Dipentyl-4'-phenyl-4'*H*-spiro[indene-1,1'-naphthalene] (**15c**)

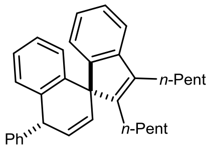

Purified by chromatography on silica gel, eluting with dichloromethane/petroleum ether 1:20 (*v*/*v*); colourless oil, 73.1 mg, 82% yield; ^{1}H NMR (600 MHz, $CDCl_3$): δ 7.35-7.32 (m, 2H), 7.31-7.27 (m, 3H), 7.26-7.22 (m, 2H), 7.08-6.99 (m, 4H), 6.91-6.88 (m, 1H), 6.50 (d, *J* = 7.8 Hz, 1H), 6.08 (dd, *J* = 10.2, 3.0 Hz, 1H), 5.25 (d, *J* = 10.2 Hz, 1H), 4.91 (s, 1H), 2.66-2.57 (m, 2H), 2.39-2.34 (m, 1H), 2.21-2.16 (m, 1H), 1.74-1.68 (dd, *J* = 14.6, 7.4 Hz, 2H), 1.48-1.39 (m, 5H), 1.39-1.21 (m, 5H), 0.95 (t, *J* = 7.2 Hz, 3H), 0.82 (t, *J* = 7.2 Hz, 3H). ^{13}C NMR (150 MHz, $CDCl_3$): δ 154.2, 151.5, 146.2, 144.8, 138.6, 137.2, 135.2, 130.0, 129.4, 128.8, 128.6, 127.8, 127.1, 126.7, 126.48, 126.43, 126.3, 125.0, 123.7, 118.7, 59.2, 45.4, 32.4, 32.2, 29.6, 28.7, 27.0, 25.7, 22.6, 22.4, 14.1, 14.0. HRMS *m*/*z* (EI+): Calculated for $C_{34}H_{38}$ (M^+): 446.2974, found 446.2971.

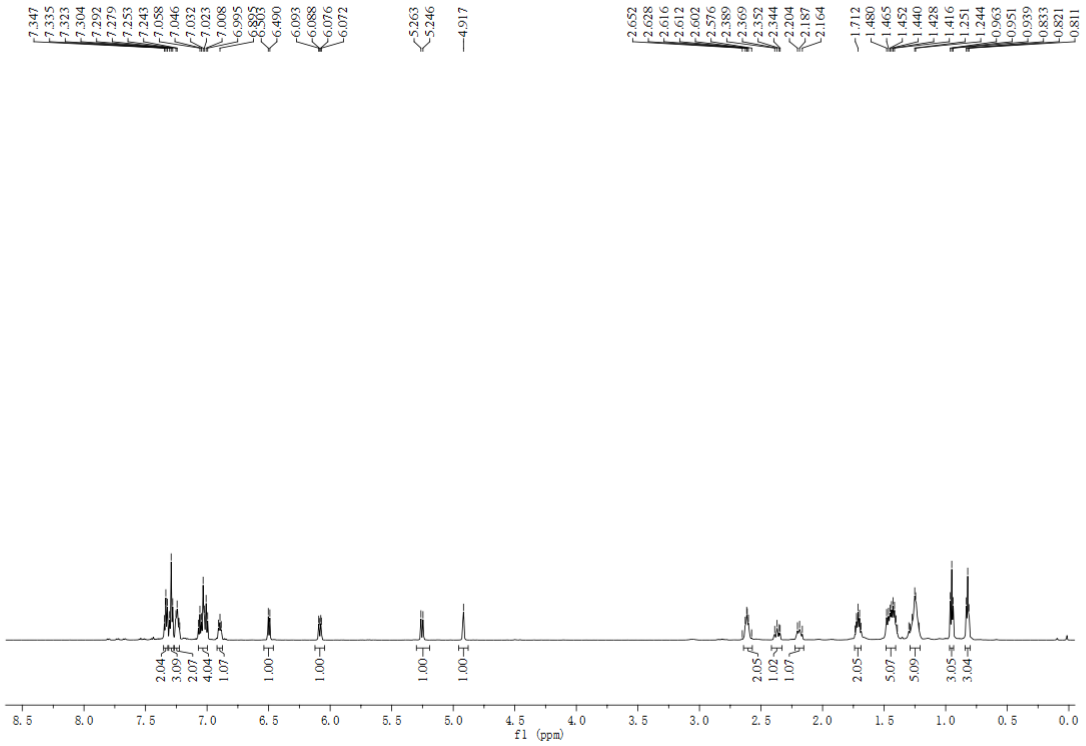

Supplementary Figure 157. ^{1}H NMR Spectra of compound **15c**.

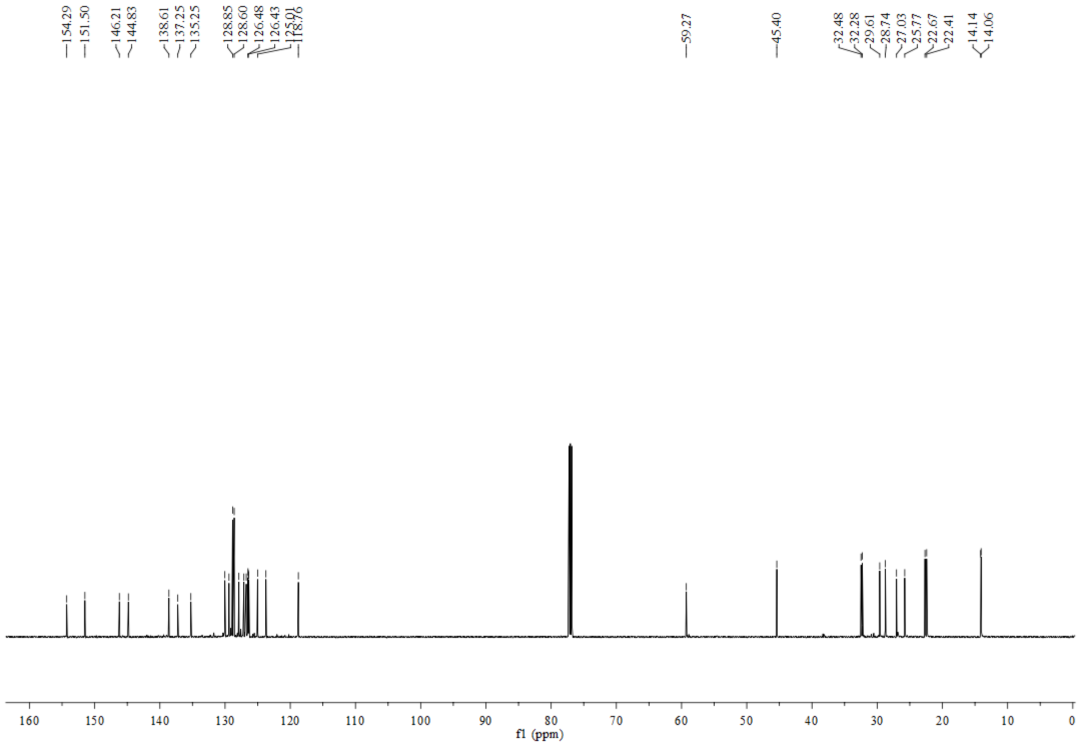

Supplementary Figure 158. ^{13}C NMR Spectra of compound **15c**.

2,3-Dipentyl-4'-(*m*-tolyl)-4'*H*-spiro[indene-1,1'-naphthalene] (**15d**)

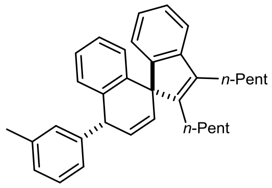

Purified by chromatography on silica gel, eluting with dichloromethane/petroleum ether 1:20 (*v*/*v*); colourless oil, 63.0 mg, 68% yield; ^{1}H NMR (500 MHz, $CDCl_3$): δ 7.35 (d, *J* = 7.5 Hz, 1H), 7.32-7.28 (m, 2H), 7.16-7.04 (m, 7H), 6.97-6.93 (m, 1H), 6.55 (d, *J* = 8.0 Hz, 1H), 6.13 (dd, *J* = 10.0, 3.5 Hz, 1H), 5.30 (dd, *J* = 10.0, 2.5 Hz, 1H), 4.93 (t, *J* = 3.0 Hz, 1H), 2.69-2.63 (m, 2H), 2.47-2.42 (m, 1H), 2.40 (s, 3H), 2.28-2.22 (m, 1H), 1.80-1.73 (m, 2H), 1.53-1.45 (m, 5H), 1.36-1.26 (m, 5H), 1.01 (t, *J* = 7.0 Hz, 3H), 0.87 (t, *J* = 7.0 Hz, 3H). ^{13}C NMR (125 MHz, $CDCl_3$): δ 154.3, 151.5, 146.1, 144.8, 138.5, 138.1, 137.3, 135.1, 130.0, 129.5, 129.4, 128.4, 127.7, 127.2, 127.0, 126.7, 126.4, 126.2, 126.0, 124.9, 123.7, 118.7, 59.2, 45.2, 32.4, 32.2, 29.6, 28.7, 27.0, 25.7, 22.6, 22.4, 21.4, 14.1, 14.0. HRMS *m*/*z* (EI+): Calculated for $C_{35}H_{40}$ (M^+): 460.3130, found 460.3116.

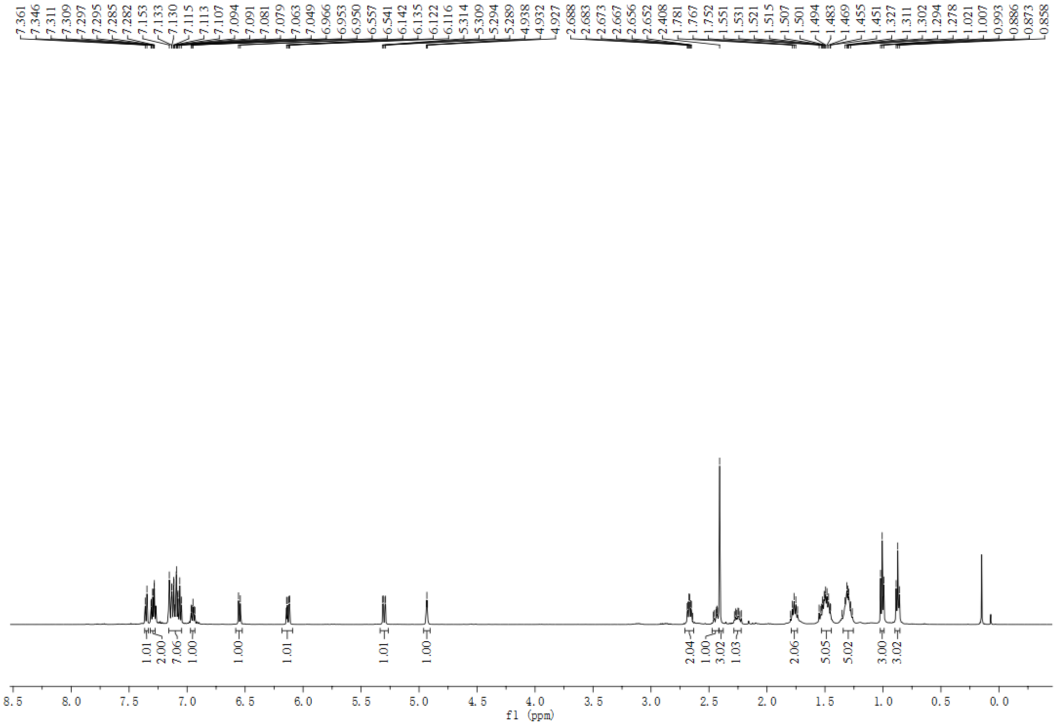

Supplementary Figure 159. ^{1}H NMR Spectra of compound **15d**.

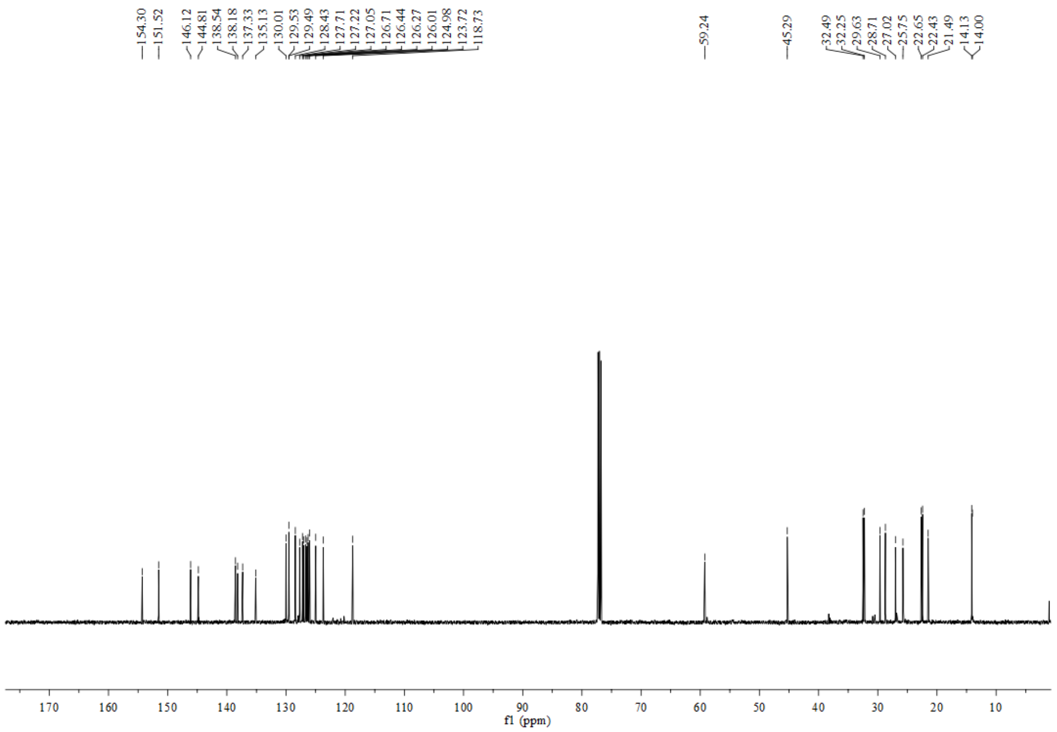

Supplementary Figure 160. ^{13}C NMR Spectra of compound **15d**.

2,3,4'-Triphenyl-4'*H*-spiro[indene-1,1'-naphthalene] (**15e**)

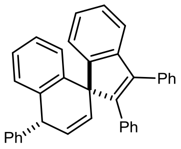

Purified by chromatography on silica gel, eluting with dichloromethane/petroleum ether 1:10 (*v*/*v*); pale yellow solid, Mp = 171-173 ^{o}C, 59.6 mg, 65% yield; ^{1}H NMR (600 MHz, $CDCl_3$): δ 7.44-7.40 (m, 3H), 7.36-7.32 (m, 2H), 7.30-7.26 (m, 2H), 7.21 – 7.17 (m, 1H), 7.16-7.11 (m, 5H), 7.09-7.01 (m, 3H), 6.97 (d, *J* = 7.2 Hz, 4H), 6.88 (d, *J* = 7.8 Hz, 1H), 6.72 (d, *J* = 4.8 Hz, 2H), 6.06 (dd, *J* = 10.2, 2.4 Hz, 1H), 5.57 (d, *J* = 9.6 Hz, 1H), 4.83 (s, 1H). ^{13}C NMR (150 MHz, $CDCl_3$): δ 154.4, 151.6, 145.3, 143.5, 140.9, 137.4, 136.0, 134.9, 134.0, 130.27, 130.21, 130.0, 129.5, 129.0, 128.4, 127.7, 127.4, 127.1, 127.05, 127.03, 126.8, 126.6, 126.4, 126.2, 124.2, 120.8, 60.5, 45.4. HRMS *m*/*z* (EI+): Calculated for $C_{36}H_{26}$ (M^{+}): 458.2035, found 458.2050.

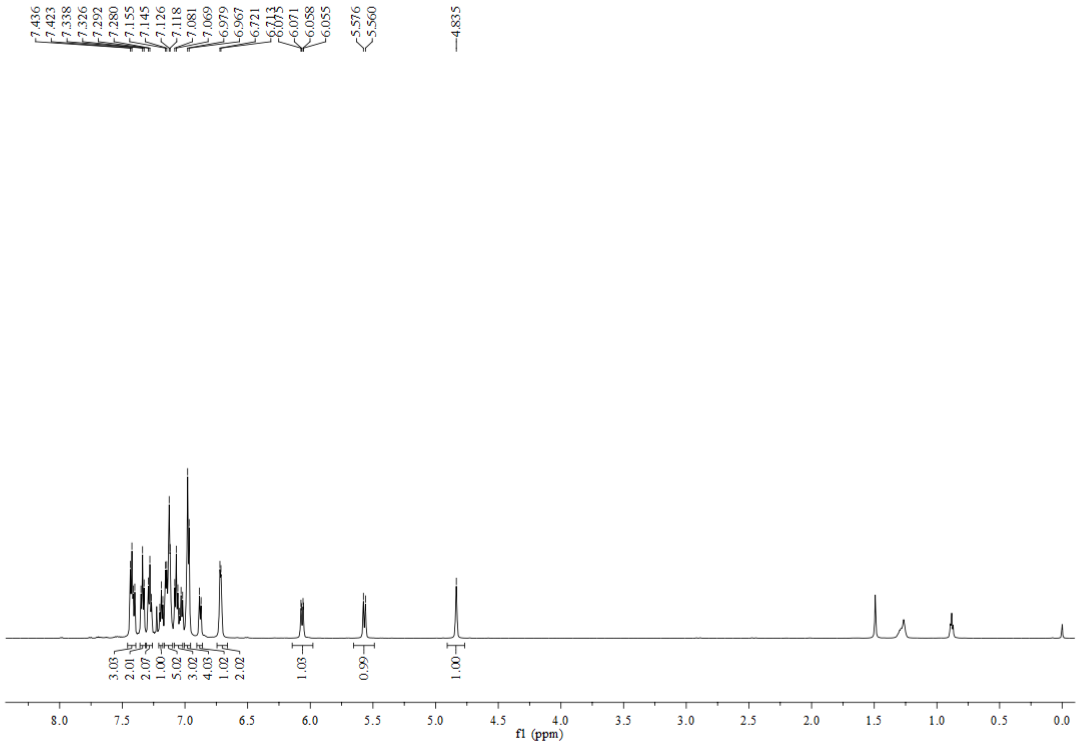

Supplementary Figure 161. ^{1}H NMR Spectra of compound **15e**.

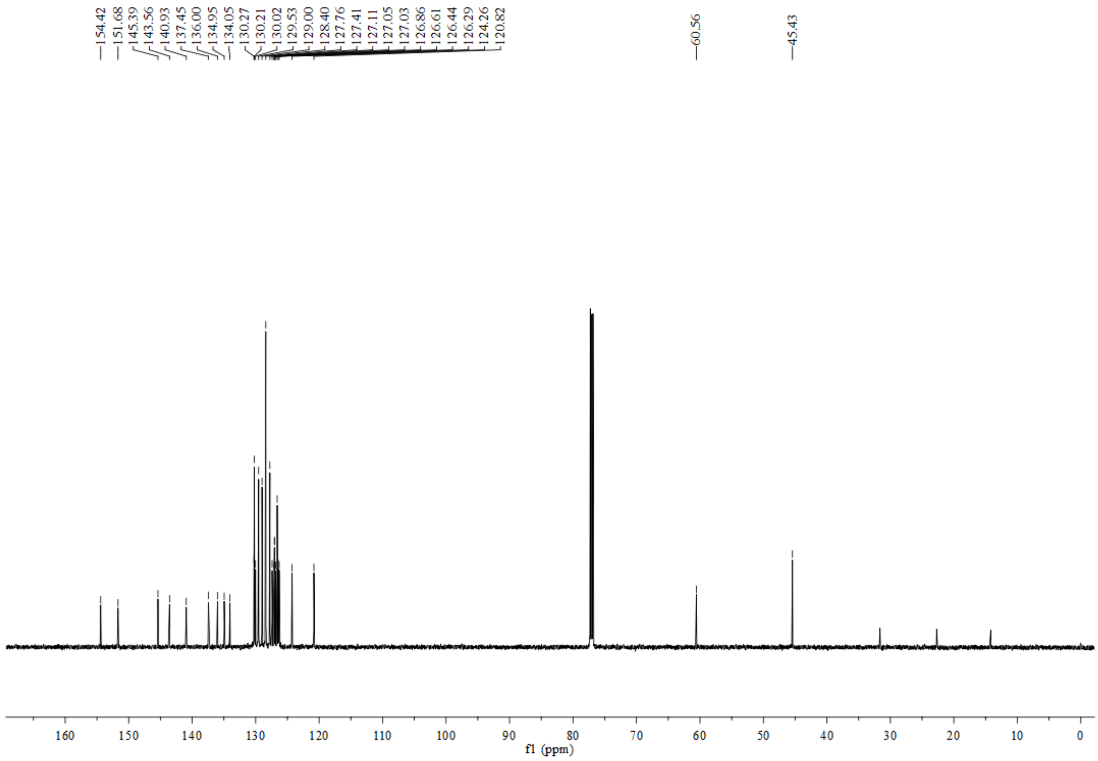

Supplementary Figure 162. ^{13}C NMR Spectra of compound **15e**.

2,3-Diphenyl-4'-(*m*-tolyl)-4'*H*-spiro[indene-1,1'-naphthalene] (**15f**)

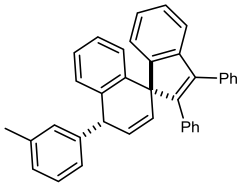

Purified by chromatography on silica gel, eluting with dichloromethane/petroleum ether 1:10 (*v*/*v*); pale yellow solid, Mp = 191-193 ^{o}C, 58.7 mg, 62% yield; ^{1}H NMR (600 MHz, $CDCl_3$): δ 7.43-7.38 (m, 3H), 7.35-7.32 (m, 2H), 7.30-7.25 (m, 7.7 Hz, 2H), 7.19-7.16 (m, 1H), 7.15-7.11 (m, 2H), 7.08-6.95 (m, 9H), 6.88 (d, *J* = 7.8 Hz, 1H), 6.66 (s, 1H), 6.58 (d, *J* = 7.8 Hz, 1H), 6.06 (dd, *J* = 10.0, 3.0 Hz, 1H), 5.57 (d, *J* = 9.6 Hz, 1H), 4.81 (s, 1H), 2.21 (s, 3H). ^{13}C NMR (150 MHz, $CDCl_3$): δ 154.4, 151.5, 145.4, 143.5, 141.0, 137.9, 137.4, 136.0, 135.0, 134.0, 130.2, 130.1, 130.0, 129.59, 129.55, 128.4, 128.2, 127.7, 127.3, 127.1, 127.08, 127.06, 126.9, 126.8, 126.6, 126.5, 126.4, 126.2, 124.2, 120.7, 60.4, 45.3, 21.4. HRMS *m*/*z* (EI+): Calculated for $C_{37}H_{28}$ (M^+): 472.2191, found 472.2192.

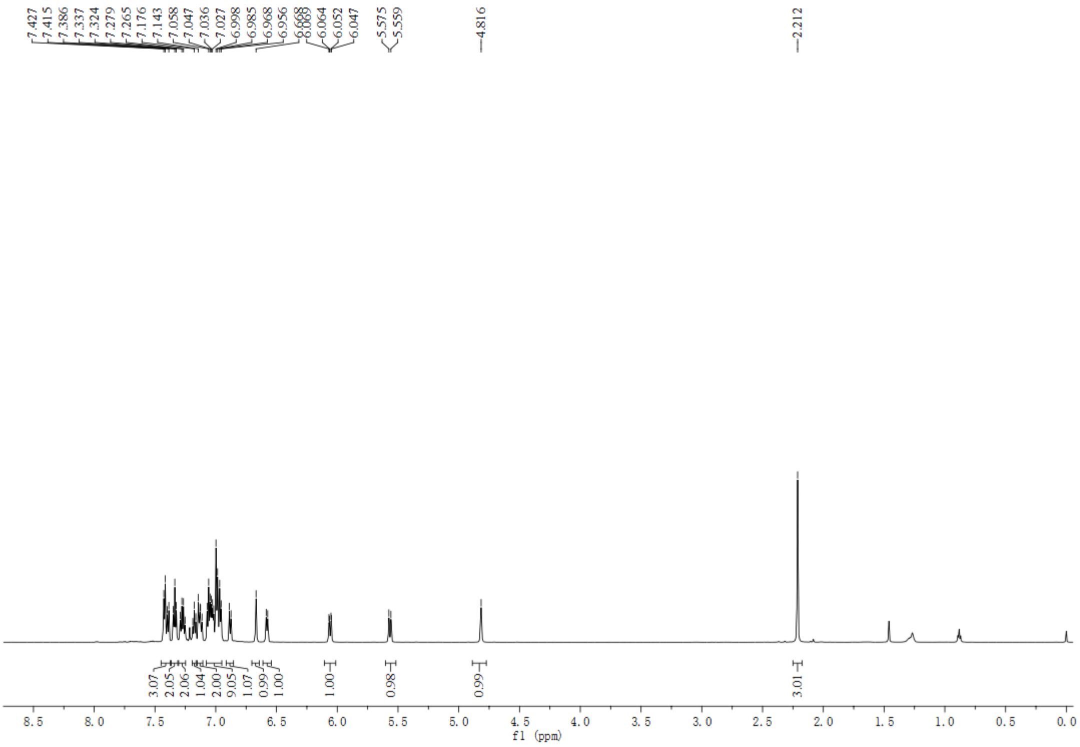

Supplementary Figure 163. ^{1}H NMR Spectra of compound **15f**.

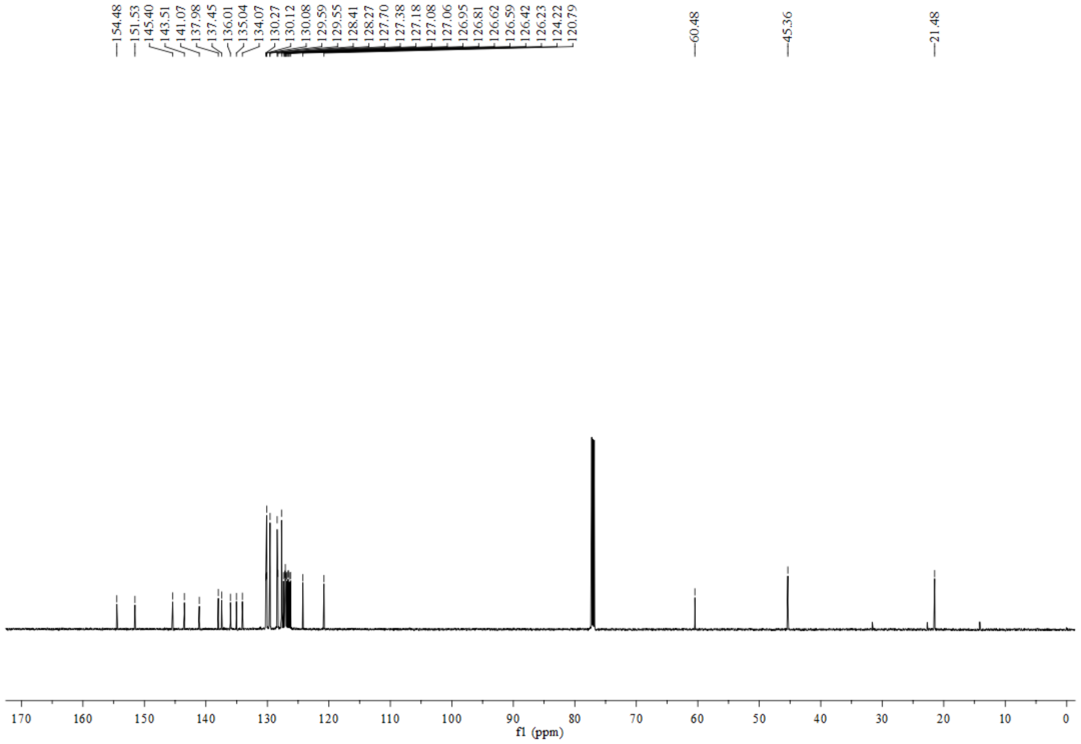

Supplementary Figure 164. ^{13}C NMR Spectra of compound **15f**.

4'-Phenyl-2,3-di-*p*-tolyl-4'*H*-spiro[indene-1,1'-naphthalene] (**15g**)

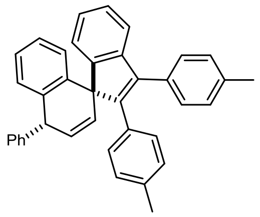

Purified by chromatography on silica gel, eluting with dichloromethane/petroleum ether 1:10 (*v*/*v*); pale yellow solid, Mp = 112-114 ^{o}C, 59.2 mg, 61% yield; ^{1}H NMR (500 MHz, $CDCl_3$): δ 7.39 (d, *J* = 7.5 Hz, 1H), 7.33 (d, *J* = 8.0 Hz, 2H), 7.28-7.24 (m, 1H), 7.18-7.10 (m, 7H), 7.04-7.00 (m, 1H), 6.98-6.95 (dd, J = 6.6, 3.8 Hz, 2H), 6.90-6.85 (m, 5H), 6.75-6.72 (m, 2H), 6.05 (dd, *J* = 10.0, 3.0 Hz, 1H), 5.55 (dd, *J* = 10.0, 2.5 Hz, 1H), 4.83 (s, 1H), 2.35 (s, 3H), 2.26 (s, 3H). ^{13}C NMR (125 MHz, $CDCl_3$): δ 154.3, 151.2, 145.5, 143.8, 140.4, 137.3, 136.9, 136.4, 134.3, 133.0, 132.0, 130.1, 130.0, 129.8, 129.3, 129.1, 129.0, 128.4, 128.2, 127.0, 126.9, 126.8, 126.7, 126.5, 126.2, 126.1, 124.1, 120.7, 60.4, 45.4, 21.3, 21.2. HRMS *m/z* (EI+): Calculated for $C_{38}H_{30}$ (M^+): 486.2348, found 486.2354.

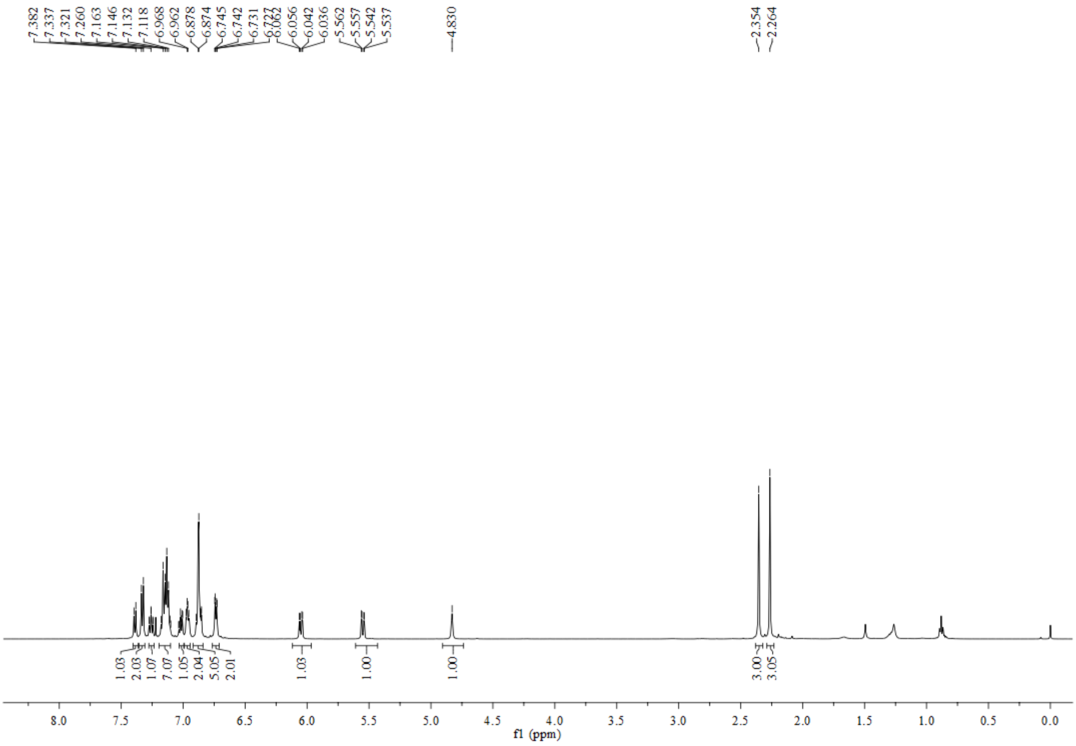

Supplementary Figure 165. ^{1}H NMR Spectra of compound **15g**.

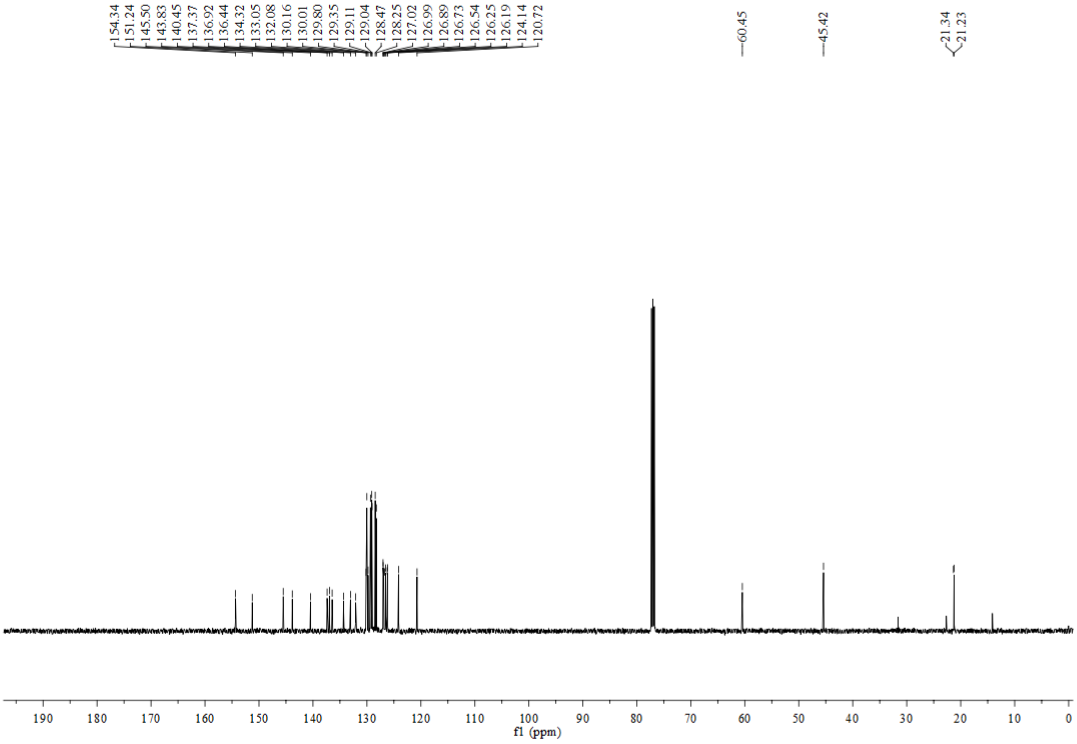

Supplementary Figure 166. ^{13}C NMR Spectra of compound **15g**.

2,3-Bis(4-(*tert*-butyl)phenyl)-4'-phenyl-4'*H*-spiro[indene-1,1'-naphthalene] (**15h**)

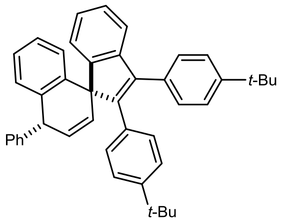

Purified by chromatography on silica gel, eluting with dichloromethane/petroleum ether 1:10 (*v*/*v*); white solid, Mp = 210-212 °C, 53.4 mg, 47% yield; ^{1}H NMR (500 MHz, $CDCl_3$): δ 7.42-7.36 (m, 5H), 7.27-7.23 (m, 1H), 7.18-7.07 (m, 7H), 7.06-6.88 (m, 8H), 6.05 (dd, *J* = 10.0, 3.0 Hz, 1H), 5.56 (dd, *J* = 10.0, 2.5 Hz, 1H), 4.86 (s, 1H), 1.34 (s, 9H), 1.25 (s, 9H). ^{13}C NMR (125 MHz, $CDCl_3$): δ 154.4, 150.8, 150.1, 149.6, 145.6, 143.8, 140.6, 137.2, 134.6, 132.9, 132.1, 130.2, 129.6, 129.18, 129.14, 128.3, 127.2, 126.97, 126.94, 126.7, 126.6, 126.3, 126.1, 125.2, 124.4, 124.0, 120.8, 60.2, 45.4, 34.6, 34.4, 31.4, 31.3. HRMS *m*/*z* (EI+): Calculated for $C_{44}H_{42}$ (M^+): 570.3287, found 570.3289.

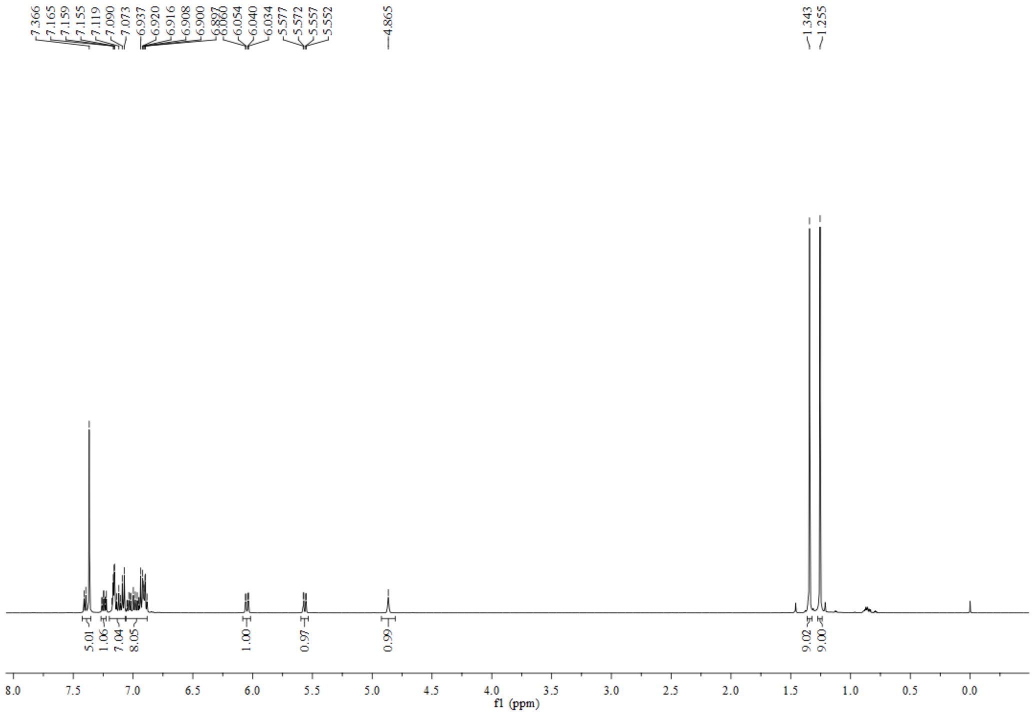

Supplementary Figure 167. ^{1}H NMR Spectra of compound **15h**.

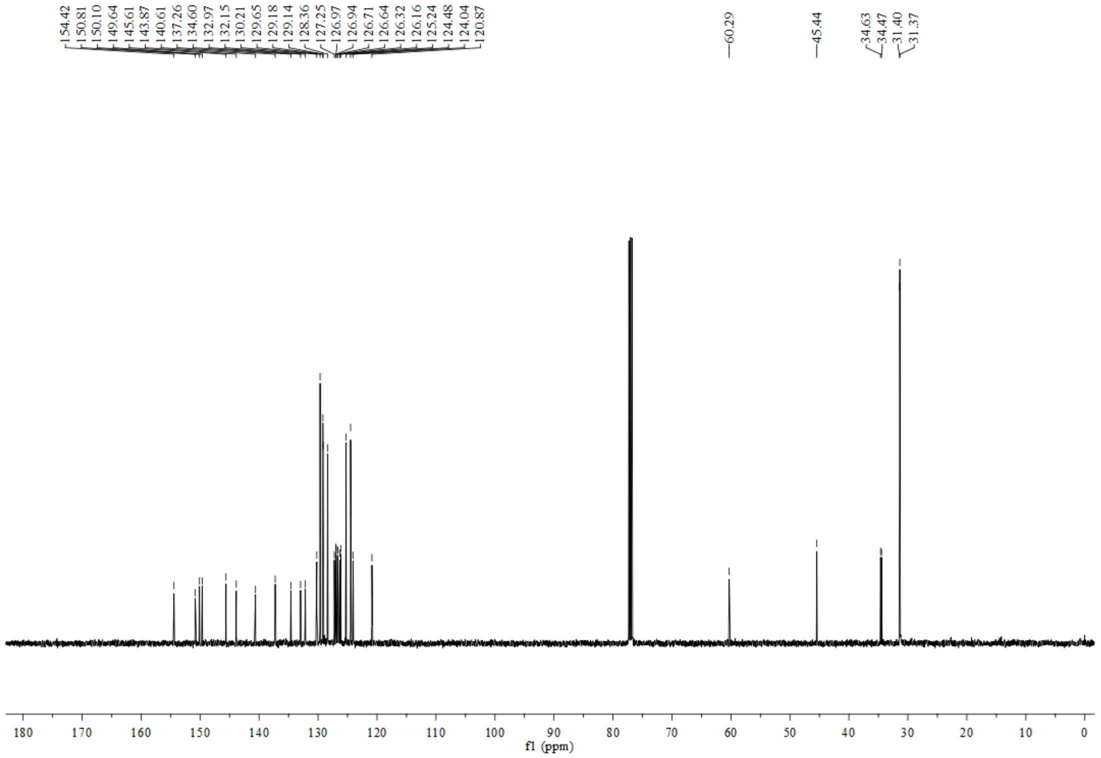

Supplementary Figure 168. ^{13}C NMR Spectra of compound **15h**.

4'-(Naphthalen-2-yl)-2,3-dipentyl-4'*H*-spiro[indene-1,1'-naphthalene] (**15i**)

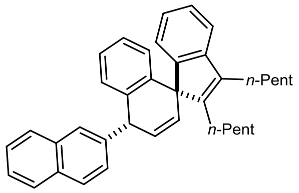

Purified by chromatography on silica gel, eluting with dichloromethane/petroleum ether 1:15 (*v*/*v*); colourless oil, 59.1 mg, 59% yield; ^{1}H NMR (500 MHz, $CDCl_3$): δ 7.89-7.84 (m, 4H), 7.56-7.49 (m, 2H), 7.41 (dd, *J* = 8.0, 1.5 Hz, 1H), 7.37 (d, *J* = 7.0 Hz, 1H), 7.33-7.29 (m, 1H), 7.15-7.04 (m, 4H), 6.99-6.95 (m, 1H), 6.60-6.58 (m, 1H), 6.19 (dd, *J* = 10.0, 3.0 Hz, 1H), 5.36 (dd, *J* = 10.0, 2.5 Hz, 1H), 5.14 (t, *J* = 2.5 Hz, 1H), 2.74-2.64 (m, 2H), 2.52-2.45 (m, 1H), 2.34-2.27 (m, 1H), 1.81-1.74 (m, 2H), 1.55-1.47 (m, 5H), 1.38-1.29 (m, 5H), 1.01 (t, *J* = 7.0 Hz, 3H), 0.88 (t, *J* = 7.5 Hz, 3H). ^{13}C NMR (125 MHz, $CDCl_3$): δ 154.2, 151.4, 144.8, 143.4, 138.7, 137.0, 135.3, 133.6, 132.3, 130.1, 129.2, 128.3, 128.2, 127.7, 127.69, 127.19, 127.12, 126.7, 126.5, 126.4, 126.1, 125.5, 125.0, 123.7, 118.7, 59.2, 45.5, 32.5, 32.2, 29.6, 28.7, 27.1, 25.7, 22.6, 22.4, 14.1, 14.0. HRMS *m*/*z* (EI+): Calculated for $C_{38}H_{40}$ (M^+): 496.3130, found

496.3144.

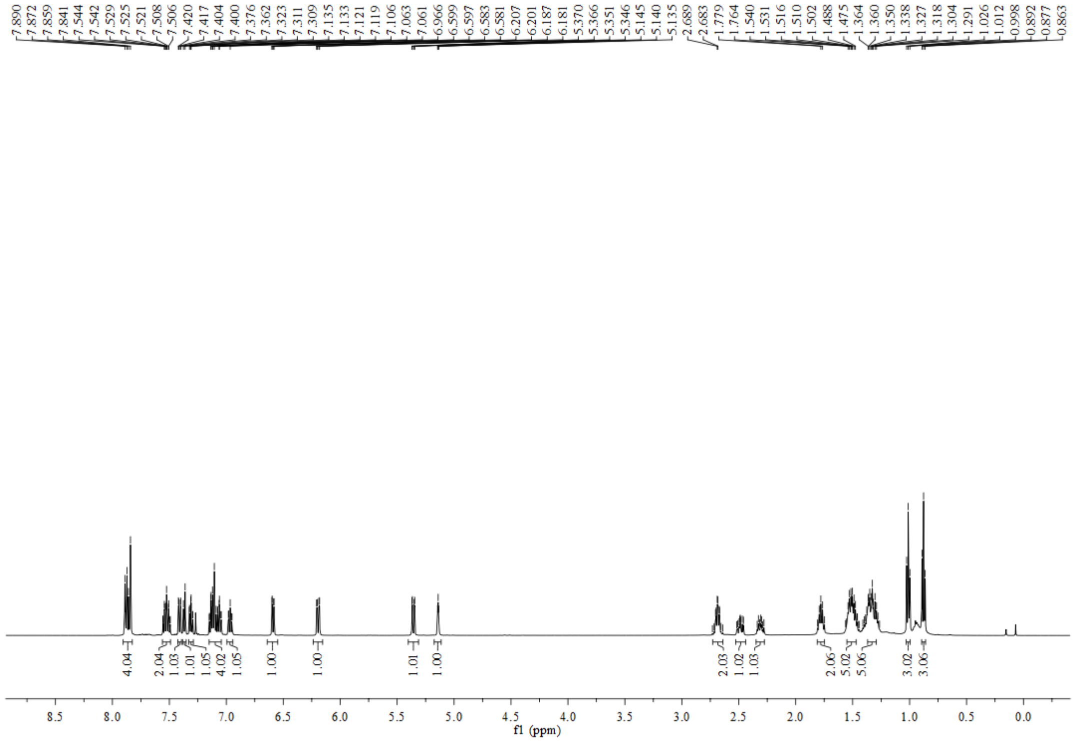

Supplementary Figure 169. ^{1}H NMR Spectra of compound **15i**.

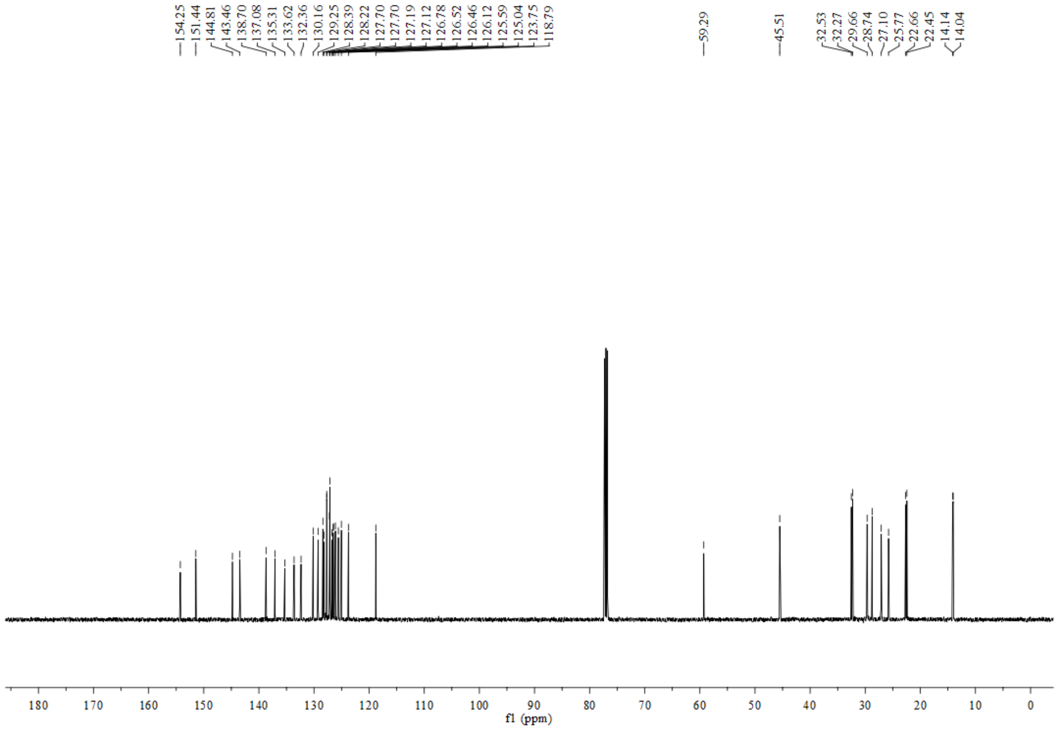

Supplementary Figure 170. ^{13}C NMR Spectra of compound **15i**.

5-Methyl-2,3-dipentyl-4'-phenyl-4'*H*-spiro[indene-1,1'-naphthalene] (**15j**)

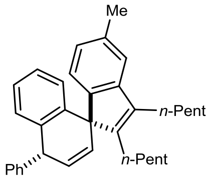

Purified by chromatography on silica gel, eluting with dichloromethane/petroleum ether 1:20 (*v*/*v*); colourless oil, 76.3 mg, 83% yield; ^{1}H NMR (600 MHz, $CDCl_3$): δ 7.39-7.35 (m, 2H), 7.33-7.31 (m, 2H), 7.30-7.26 (m, 1H), 7.14 (s, 1H), 7.07-7.03 (m, 2H), 6.95-6.91 (m, 3H), 6.54 (d, J = 7.8 Hz, 1H), 6.10 (dd, J = 9.6, 3.0 Hz, 1H), 5.28 (dd, J = 9.6, 2.4 Hz, 1H), 4.94 (t, J = 2.4 Hz, 1H), 2.67-2.59 (m, 2H), 2.43 (s, 3H), 2.42-2.36 (m, 1H), 2.24-2.18 (m, 1H), 1.77-1.71 (m, 2H), 1.54-1.42 (m, 5H), 1.34-1.24 (m, 5H), 0.99 (t, J = 7.2 Hz, 3H), 0.85 (t, J = 7.2 Hz, 3H). ^{13}C NMR (150 MHz, $CDCl_3$): δ 151.8, 151.6, 146.2, 145.0, 138.5, 137.2, 136.4, 135.5, 130.0, 129.1, 128.8, 128.6, 128.1, 127.1, 126.43, 126.41, 126.3, 125.7, 123.4, 119.5, 58.8, 45.3, 32.4, 32.3, 29.6, 28.7, 27.0, 25.7, 22.7, 22.4, 21.7, 14.1, 14.0. HRMS *m*/*z* (EI+): Calculated for $C_{35}H_{40}$ (M^+): 460.3130, found 460.3112.

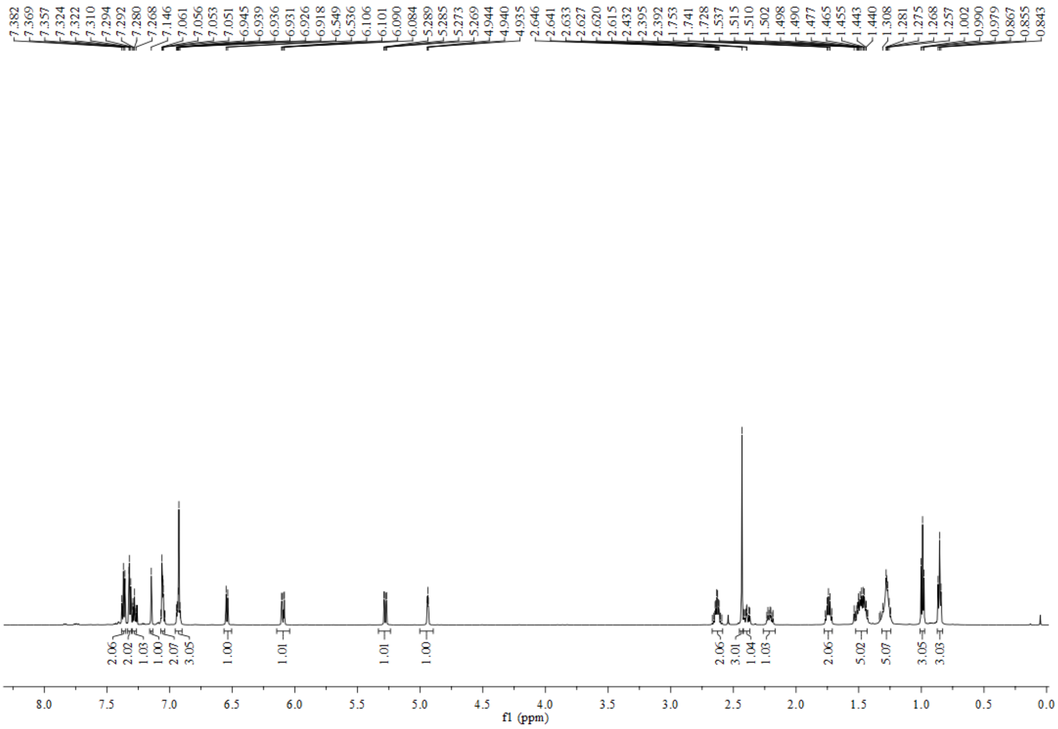

Supplementary Figure 171. ^{1}H NMR Spectra of compound **15j**.

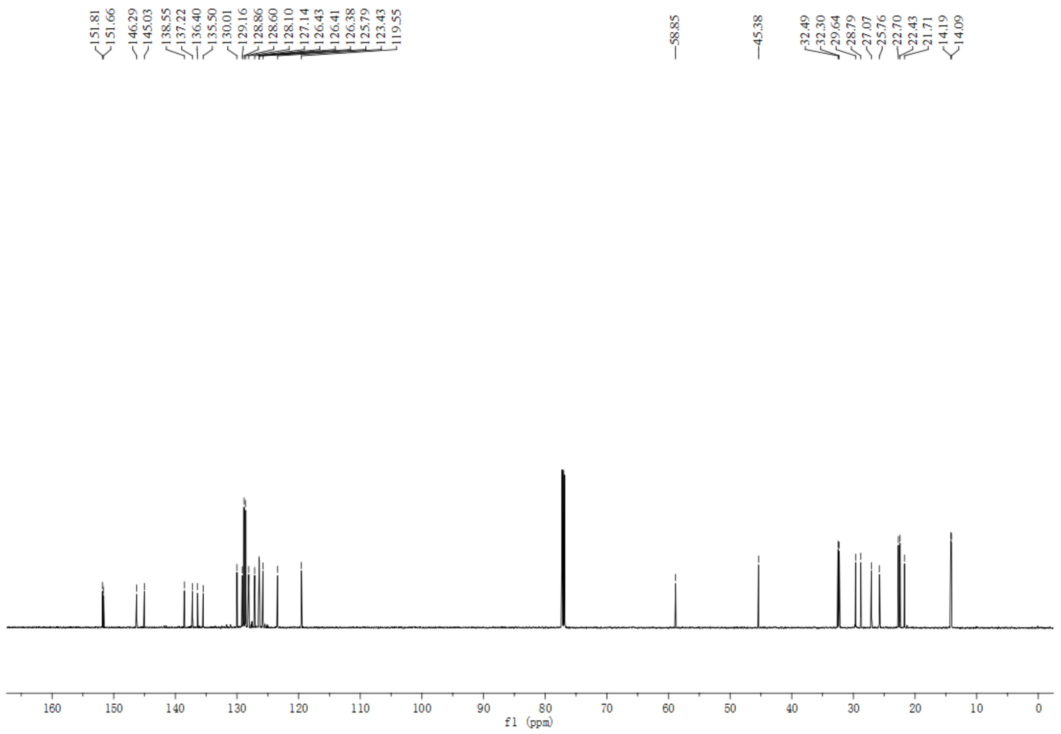

Supplementary Figure 172. ^{13}C NMR Spectra of compound **15j**.

5-Methoxy-2,3-dipentyl-4'-phenyl-4'*H*-spiro[indene-1,1'-naphthalene] (**15k**)

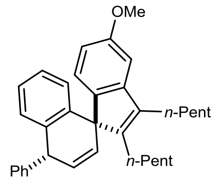

Purified by chromatography on silica gel, eluting with dichloromethane/petroleum ether 1:5 (*v*/*v*); colourless oil, 63.5 mg, 67% yield; ^{1}H NMR (600 MHz, $CDCl_3$): δ 7.34-7.30 (m, 2H), 7.28-7.22 (m, 3H), 7.03-7.00 (m, 2H), 6.91-6.87 (m, 2H), 6.84 (d, *J* = 1.8 Hz, 1H), 6.61-6.58 (dd, *J* = 8.4, 2.4 Hz, 1H), 6.49 (d, *J* = 7.8 Hz, 1H), 6.04 (dd, *J* = 9.6, 3.0 Hz, 1H), 5.23 (dd, J = 10.2, 2.4 Hz, 1H), 4.88 (t, *J* = 2.4 Hz, 1H), 3.81 (s, 3H), 2.60-2.52 (m, 2H), 2.37-2.31 (m, 1H), 2.18-2.12 (m, 1H), 1.71-1.65 (m, 2H), 1.47-1.37 (m, 5H), 1.28-1.19 (m, 5H), 0.94 (t, *J* = 7.2 Hz, 3H), 0.80 (t, *J* = 7.2 Hz, 3H). ^{13}C NMR (150 MHz, $CDCl_3$): δ 159.2, 152.9, 146.8, 146.3, 146.2, 138.3, 137.1, 135.5, 129.9, 129.0, 128.8, 128.5, 128.0, 127.1, 126.4, 126.3, 126.3, 124.1, 109.7, 105.2, 58.4, 55.4, 45.3, 32.4, 32.2, 29.5, 28.7, 27.1, 25.7, 22.6, 22.4, 14.1, 14.0. HRMS *m*/*z* (EI+): Calculated for $C_{35}H_{40}O$ (M^+): 476.3079, found 476.3068.

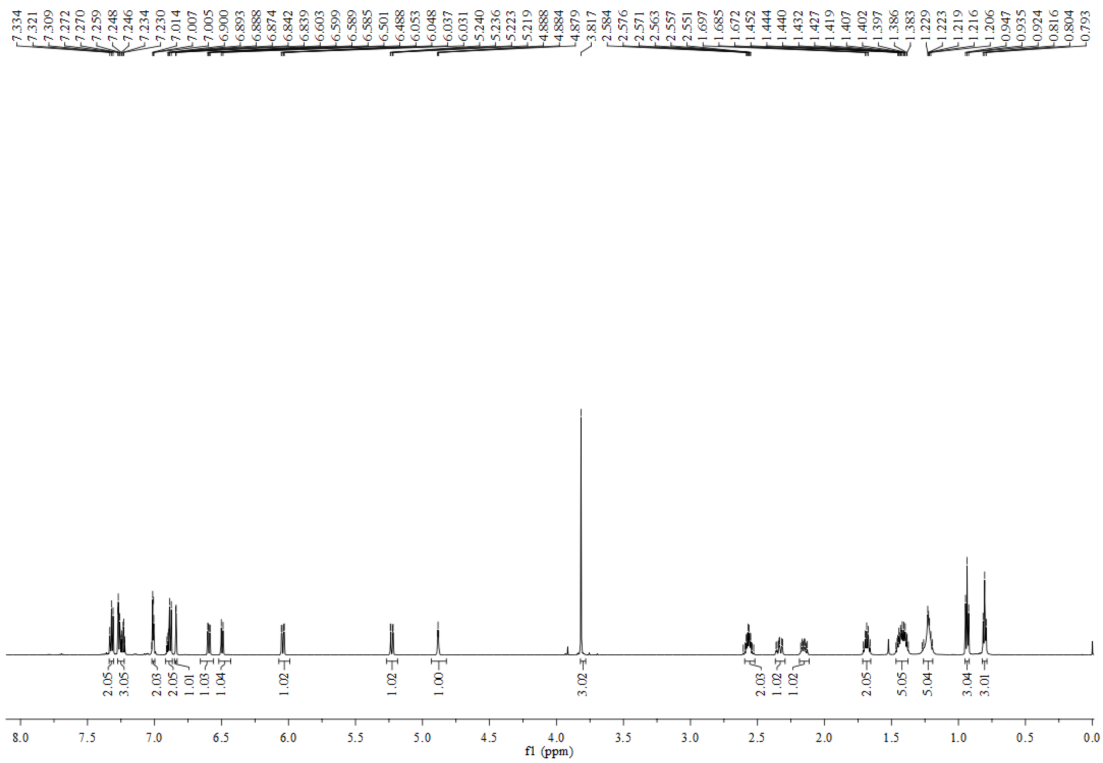

Supplementary Figure 173. ^{1}H NMR Spectra of compound **15k**.

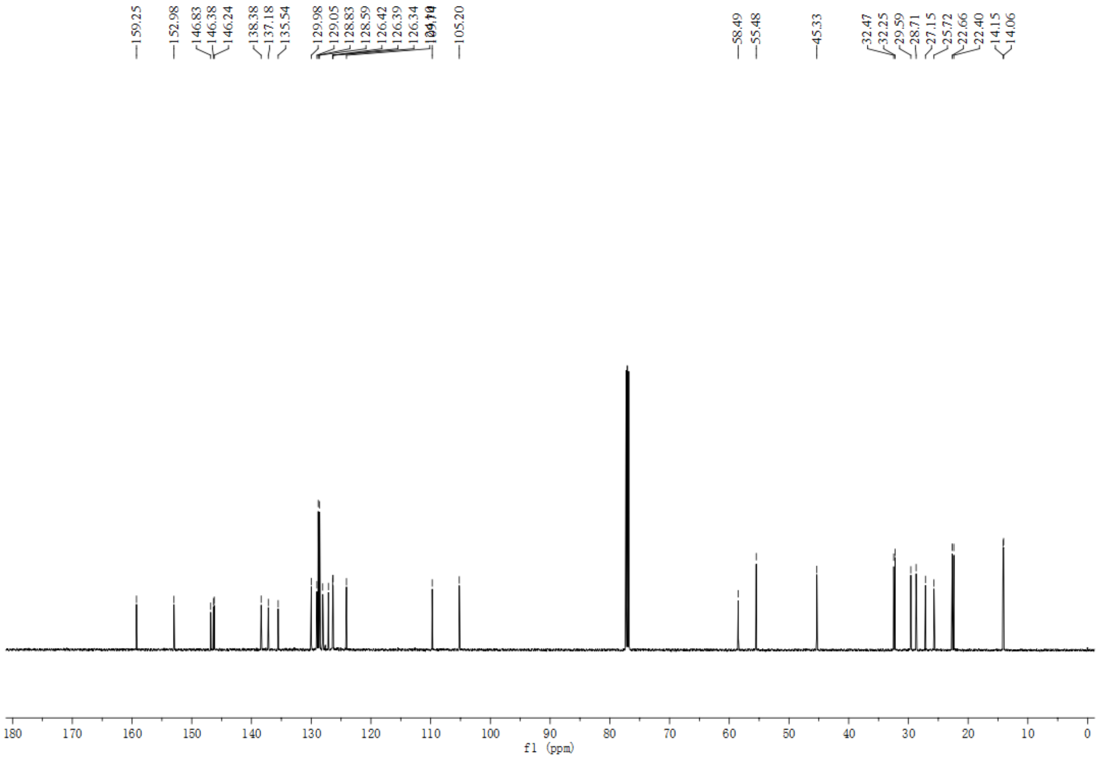

Supplementary Figure 174. ^{13}C NMR Spectra of compound **15k**.

5-Fluoro-2,3-dipentyl-4'-phenyl-4'*H*-spiro[indene-1,1'-naphthalene] (**15l**)

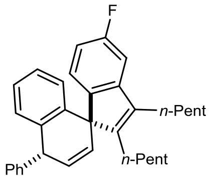

Purified by chromatography on silica gel, eluting with dichloromethane/petroleum ether 1:25 (*v*/*v*); colourless oil, 74.4 mg, 80% yield; ^{1}H NMR (600 MHz, $CDCl_3$): δ 7.34-7.31 (m, 2H), 7.27-7.22 (m, 3H), 7.03 (d, *J* = 3.6 Hz, 2H), 6.97-6.94 (m, 1H), 6.92-6.88 (m, 2H), 6.74-6.70 (m, 1H), 6.47 (d, *J* = 7.8 Hz, 1H), 6.07 (dd, *J* = 9.6, 3.0 Hz, 1H), 5.21 (dd, *J* = 9.6, 2.4 Hz, 1H), 4.89 (t, *J* = 3.0 Hz, 1H), 2.59-2.53 (m, 2H), 2.37-2.31 (m, 1H), 2.19-2.13 (m, 1H), 1.70-1.64 (m, 2H), 1.45-1.40 (m, 5H), 1.29-1.19 (m, 5H), 0.94 (t, *J* = 7.2 Hz, 3H), 0.80 (t, *J* = 6.6 Hz, 3H). ^{13}C NMR (150 MHz, $CDCl_3$): δ 162.7 (d, *J* = 241.5 Hz), 153.8, 149.6 (d, *J* = 1.5 Hz), 146.9 (d, *J* = 7.5 Hz), 146.0, 138.1 (d, *J* = 1.5 Hz), 137.2, 134.8, 130.1, 129.5, 128.8, 128.6, 127.4, 126.9, 126.6, 126.5, 126.4, 124.5 (d, *J* = 9.0 Hz), 111.4 (d, *J* = 22.5 Hz), 106.0 (d, *J* = 22.5 Hz), 58.6, 45.2, 32.4, 32.2, 29.4, 28.6, 27.1, 25.7, 22.6, 22.3, 14.1, 14.0. HRMS *m*/*z* (EI+): Calculated for $C_{34}H_{37}F$ (M^+): 464.2879, found 464.2869.

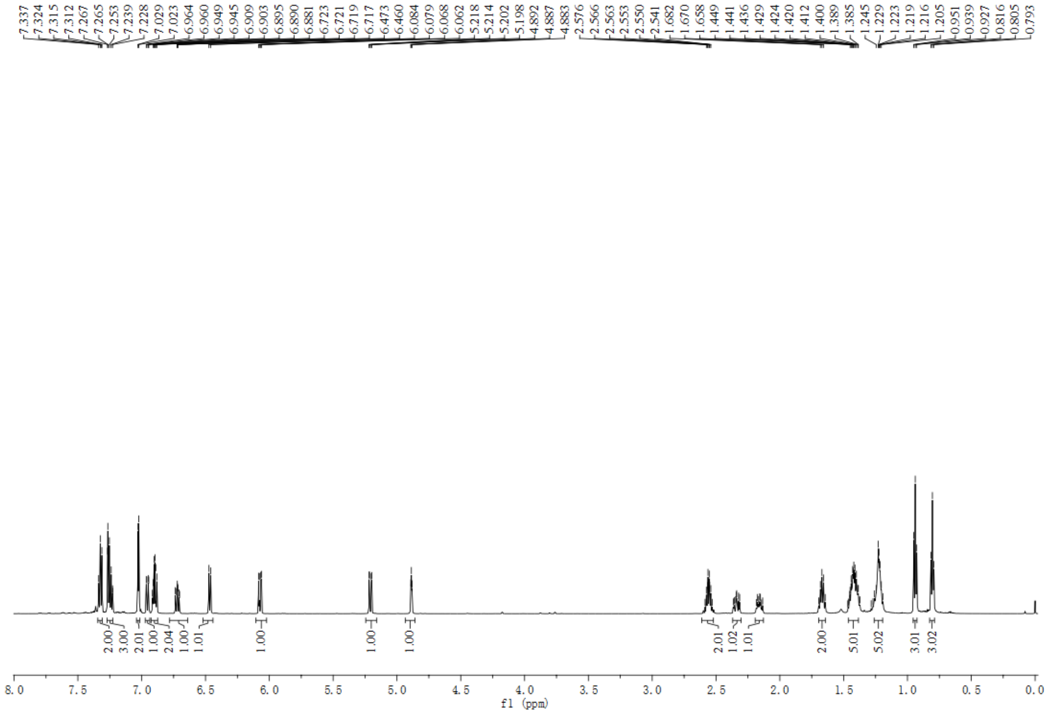

Supplementary Figure 175. ^{1}H NMR Spectra of compound **15l**.

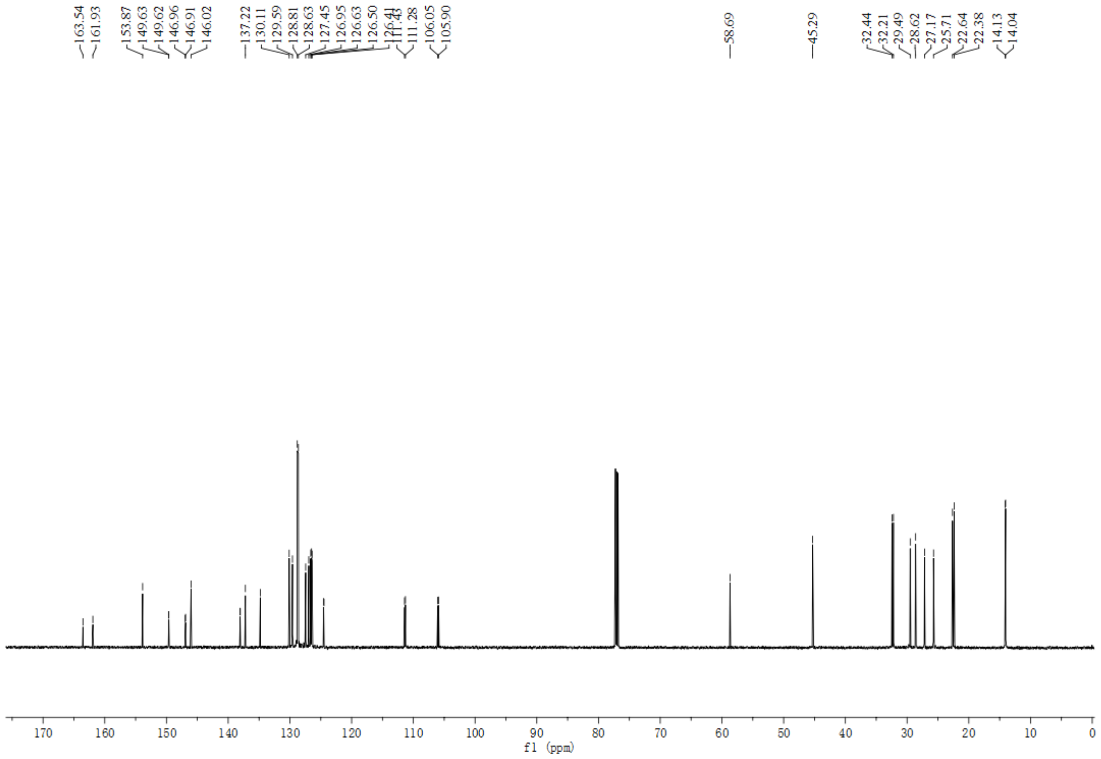

Supplementary Figure 176. ^{13}C NMR Spectra of compound **15l**.

6-Methyl-2,3-dipentyl-4'-phenyl-4'*H*-spiro[indene-1,1'-naphthalene] (**15m**)

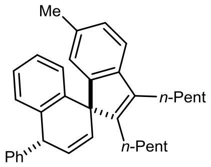

Purified by chromatography on silica gel, eluting with dichloromethane/petroleum ether 1:25 (*v*/*v*); colourless oil, 51.0 mg, 55% yield; ^{1}H NMR (600 MHz, $CDCl_3$): δ 7.34-7.31 (m, 2H), 7.29-7.26 (m, 2H), 7.25-7.22 (m, 1H), 7.17 (d, *J* = 7.2 Hz, 1H), 7.07-7.01 (m, 3H), 6.91-6.88 (m, 1H), 6.81 (s, 1H), 6.50 (d, *J* = 7.8 Hz, 1H), 6.06 (dd, *J* = 11.4, 3.6 Hz, 1H), 5.24 (dd, *J* = 9.6, 2.4 Hz, 1H), 4.91 (t, *J* = 3.0 Hz, 1H), 2.62-2.53 (m, 2H), 2.36-2.30 (m, 1H), 2.25 (s, 3H), 2.17-2.12 (m, 1H), 1.71-1.65 (m, 2H), 1.47-1.36 (m, 5H), 1.27-1.19 (m, 5H), 0.93 (t, *J* = 7.2 Hz, 3H), 0.80 (t, *J* = 5.5 Hz, 3H). ^{13}C NMR (150 MHz, $CDCl_3$): δ 154.5, 150.4, 146.2, 142.2, 138.4, 137.2, 135.5, 134.6, 129.9, 129.1, 128.8, 128.6, 128.1, 127.4, 127.2, 126.43, 126.41, 126.3,

124.5, 118.4, 59.0, 45.3, 32.4, 32.2, 29.6, 28.7, 26.9, 25.8, 22.6, 22.4, 21.4, 14.1, 14.0.

HRMS *m/z* (EI+): Calculated for $C_{35}H_{40}$ (M^+): 460.3130, found 460.3132.

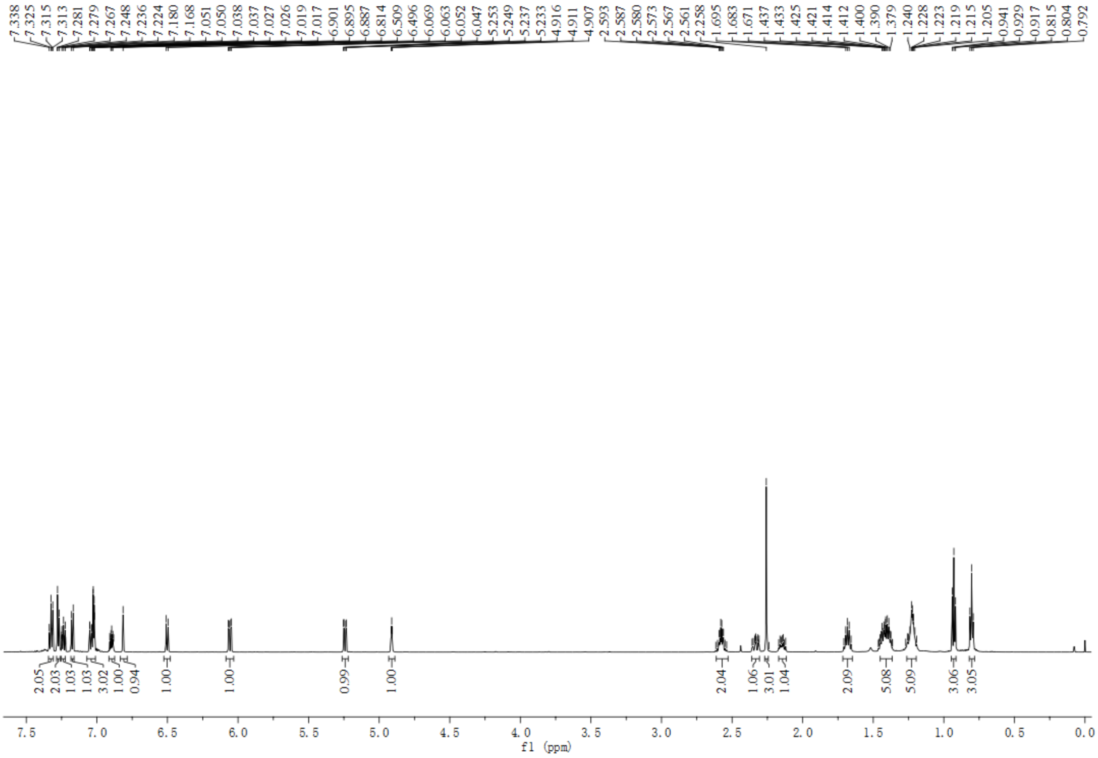

Supplementary Figure 177. ^{1}H NMR Spectra of compound **15m**.

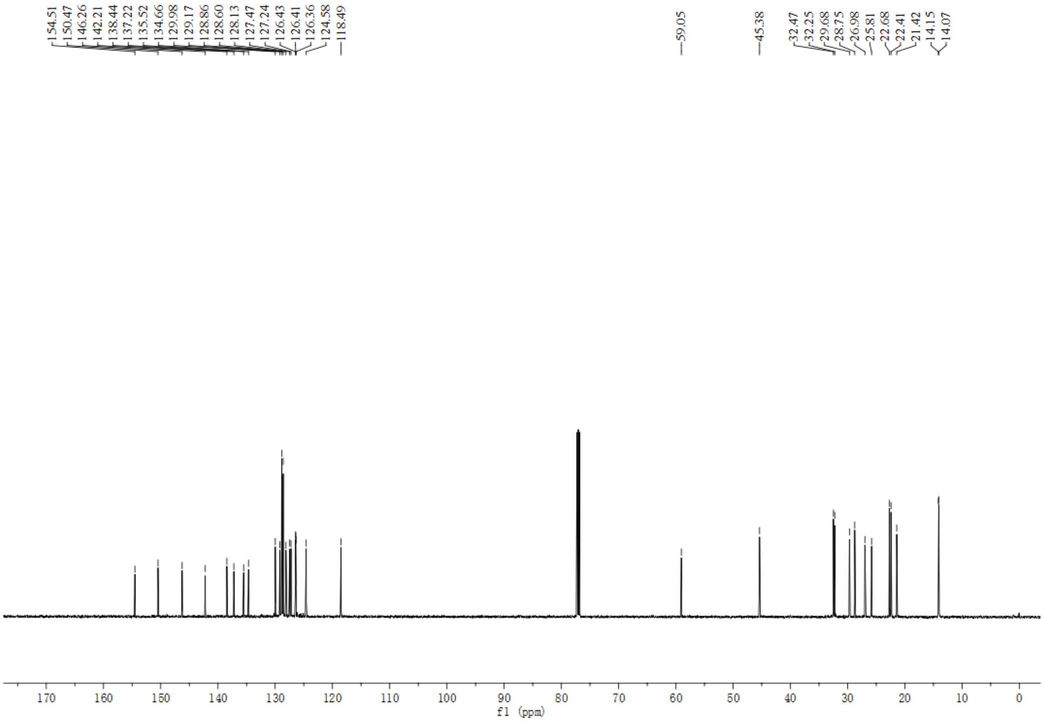

Supplementary Figure 178. ^{13}C NMR Spectra of compound **15m**.

6-Fluoro-2,3-dipentyl-4'-phenyl-4'*H*-spiro[indene-1,1'-naphthalene] (**15n**)

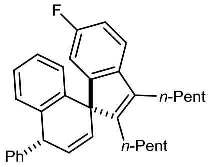

Purified by chromatography on silica gel, eluting with dichloromethane/petroleum ether 1:25 (*v*/*v*); colourless oil, 76.7 mg, 83% yield; ^{1}H NMR (600 MHz, $CDCl_3$): δ 7.34-7.31 (m, 2H), 7.27-7.22 (m, 3H), 7.20-7.17 (m, 1H), 7.04-7.02 (m, 2H), 6.94-6.89 (m, 2H), 6.70 (dd, *J* = 9.0, 2.4 Hz, 1H), 6.48 (d, *J* = 7.8 Hz, 1H), 6.09 (dd, J = 9.6, 3.0 Hz, 1H), 5.23 (dd, J = 10.2, 2.4 Hz, 1H), 4.89 (t, *J* = 3.0 Hz, 1H), 2.62-2.53 (m, 2H), 2.36-2.30 (m, 1H), 2.18-2.12 (m, 1H), 1.70-1.64 (m, 2H), 1.47-1.37 (m, 5H), 1.27-1.19 (m, 5H), 0.94 (t, *J* = 6.6 Hz, 3H), 0.80 (t, *J* = 7.2 Hz, 3H). ^{13}C NMR (150 MHz, $CDCl_3$): δ 161.5 (d, *J* = 241.5 Hz), 156.2 (d, *J* = 6.0 Hz), 151.2 (d, *J* = 1.5 Hz), 145.9, 140.6 (d, *J* = 1.5 Hz), 137.8, 137.2, 134.6, 130.1, 129.8, 128.8, 128.6, 127.3, 127.0, 126.7, 126.5, 126.4, 119.3 (d, *J* = 9.0 Hz), 113.5 (d, *J* = 22.5 Hz), 111.5 (d, *J* = 24.0 Hz), 59.2 (d, *J* = 3.0 Hz), 45.2, 32.4, 32.2, 29.6, 28.6, 27.0, 25.8, 22.6, 22.4, 14.1, 14.0. HRMS *m*/*z* (EI+): Calculated for $C_{34}H_{37}F$ (M^+): 464.2879, found 464.2901.

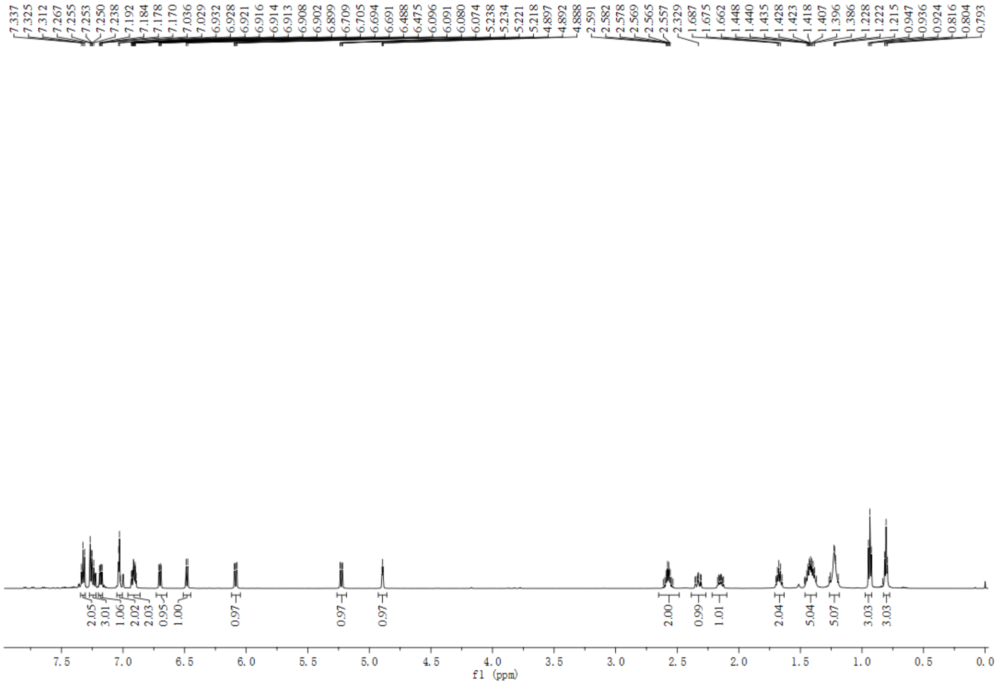

Supplementary Figure 179. ^{1}H NMR Spectra of compound **15n**.

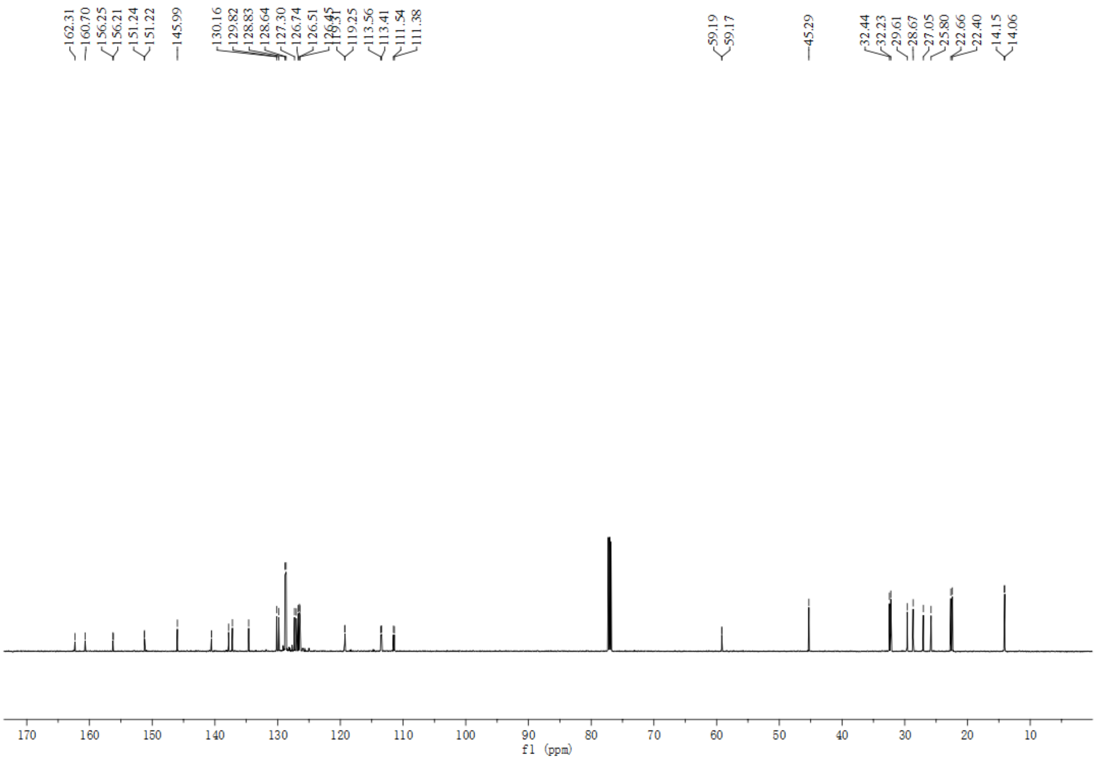

Supplementary Figure 180. ^{13}C NMR Spectra of compound **15n**.

Synthetic transformation of product 3a to 16

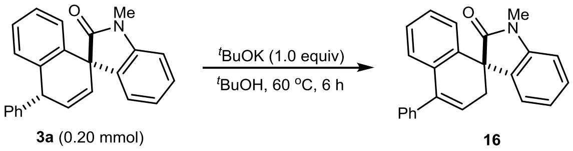

To a solution of **3a** (67.5 mg, 0.20 mmol) in ^{t}BuOH (2.0 mL) was added ^{t}BuOK (14.8 mg, 1.0 equiv.) and the mixture was stirred at 60 °C for 6 h. The solvent was removed under vacuum, the residue was purified by flash column chromatography on silica gel, eluting with ethyl acetate/petroleum ether 1:15 (*v*/*v*). giving the desired product **16**, white solid, Mp = 166-168 °C, 61.6 mg, 91% yield; ^{1}H NMR (500 MHz, $CDCl_3$): δ 7.52-7.45 (m, 4H), 7.43-7.40 (m, 2H), 7.30-7.26 (m, 1H), 7.18-7.11 (m, 3H), 6.96-6.92 (m, 2H), 6.88 (d, *J* = 7.5 Hz, 1H), 6.10-6.07 (m, 1H), 3.39 (s, 3H), 3.23 (dd, *J* = 16.5, 3.0 Hz, 1H), 2.59 (dd, *J* = 16.5, 6.0 Hz, 1H). ^{13}C NMR (125 MHz, $CDCl_3$): δ 179.8, 141.6, 140.6, 140.2, 136.0, 134.2, 133.8, 128.6, 128.4, 128.3, 128.2, 127.58, 127.52, 126.8, 125.7, 123.6, 123.5, 122.7, 108.4, 52.3, 33.8, 26.5. HRMS *m*/*z* (ESI+): Calculated for $C_{24}H_{20}NO$ ([M+H]$^+$): 338.1539, found 338.1548.

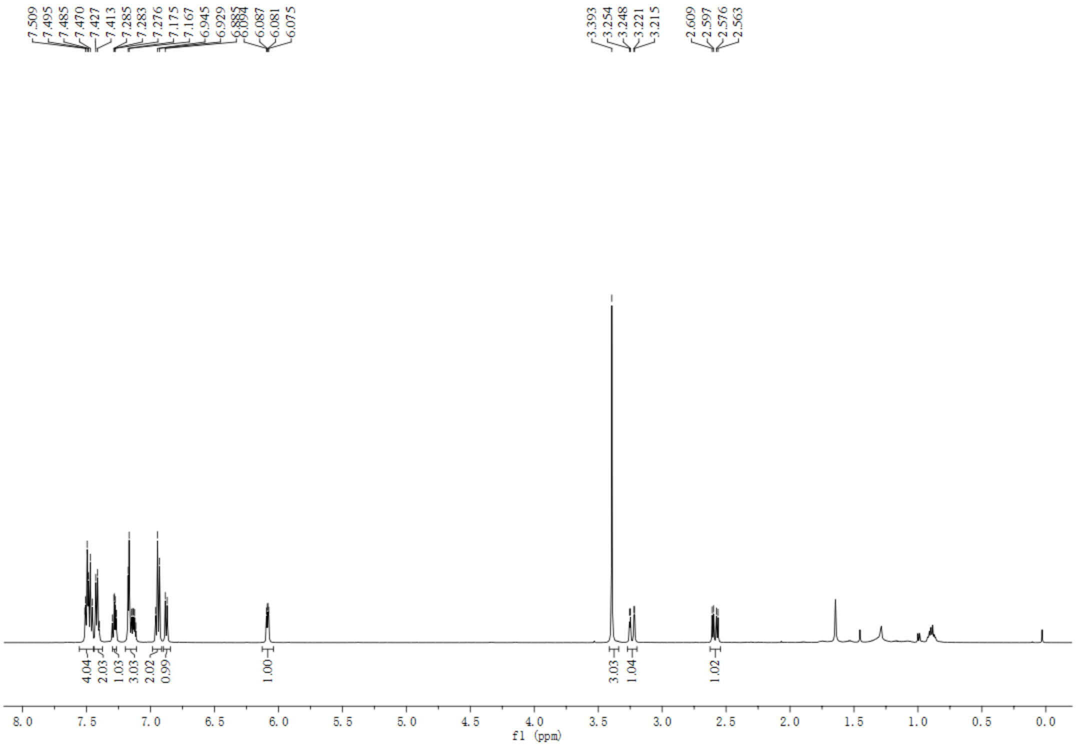

Supplementary Figure 181. ^{1}H NMR Spectra of compound **16**.

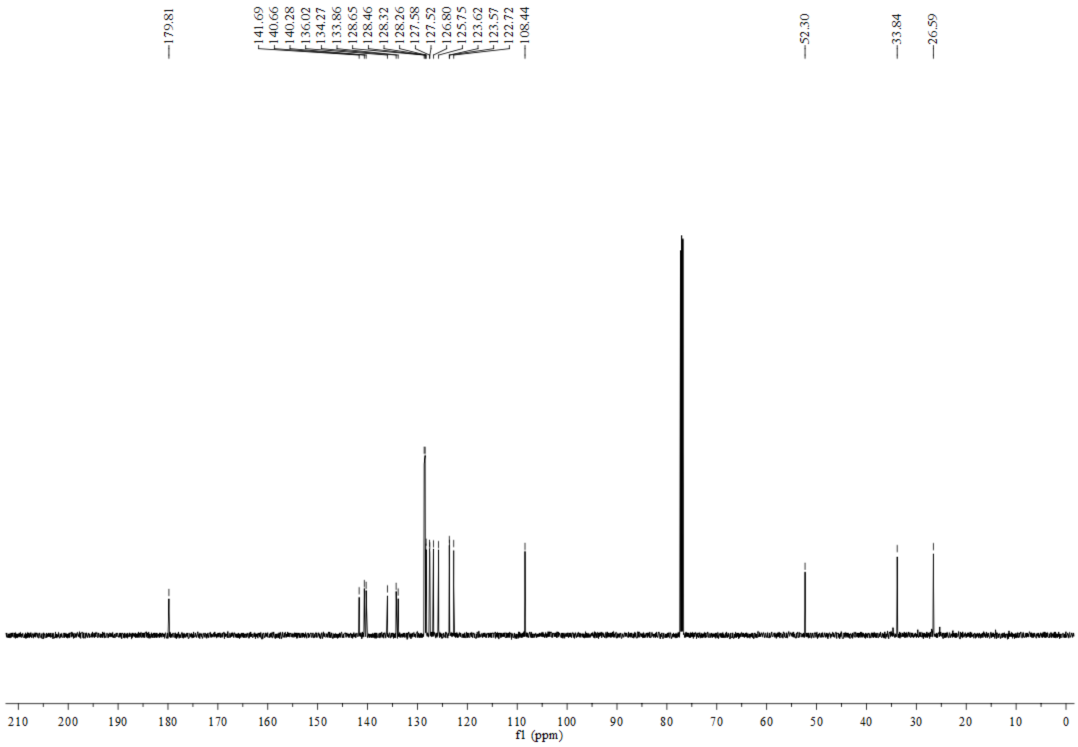

Supplementary Figure 182. ^{13}C NMR Spectra of compound **16**.

Synthetic transformation of product 3a to 17

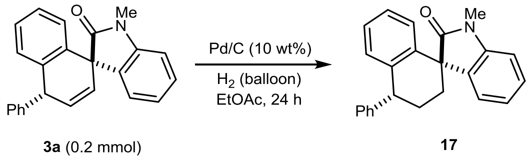

To a solution of **3a** (67.5 mg, 0.2 mmol) in ethyl acetate (2.0 mL) was added Pd/C (6.8 mg, 10 wt%) and the mixture was stirred with a hydrogen balloon at room temperature for 24 h. The solvent was removed under vacuum, the residue was purified by flash column chromatography on silica gel, eluting with ethyl acetate/petroleum ether 1:15 (*v*/*v*), giving the desired product **17**, white solid, Mp = 138-140 ^{o}C, 62.7 mg, 92% yield; ^{1}H NMR (500 MHz, $CDCl_3$): δ 7.40-7.32 (m, 3H), 7.30-7.26 (m, 1H), 7.25-7.23 (m, 2H), 7.21-7.19 (m, 1H), 7.13-7.02 (m, 3H), 7.01-6.96 (m, 2H), 6.59-6.57 (m, 1H), 4.41 (t, *J* = 6.5 Hz, 1H), 3.33 (s, 3H), 2.80-2.72 (m, 1H), 2.20-2.12 (m, 2H), 2.07-1.99 (m, 1H). ^{13}C NMR (150 MHz, $CDCl_3$): δ 180.3, 146.8, 143.2, 139.8, 137.1, 136.0, 130.7, 128.8, 128.4, 128.0, 127.8, 127.3, 126.9, 126.2, 124.0, 122.9, 108.1, 52.3, 44.9, 31.0, 28.3, 26.5. HRMS *m*/*z* (ESI+): Calculated

for $C_{24}H_{21}NNaO$ ([M+Na]$^+$): 362.1515, found 362.1517.

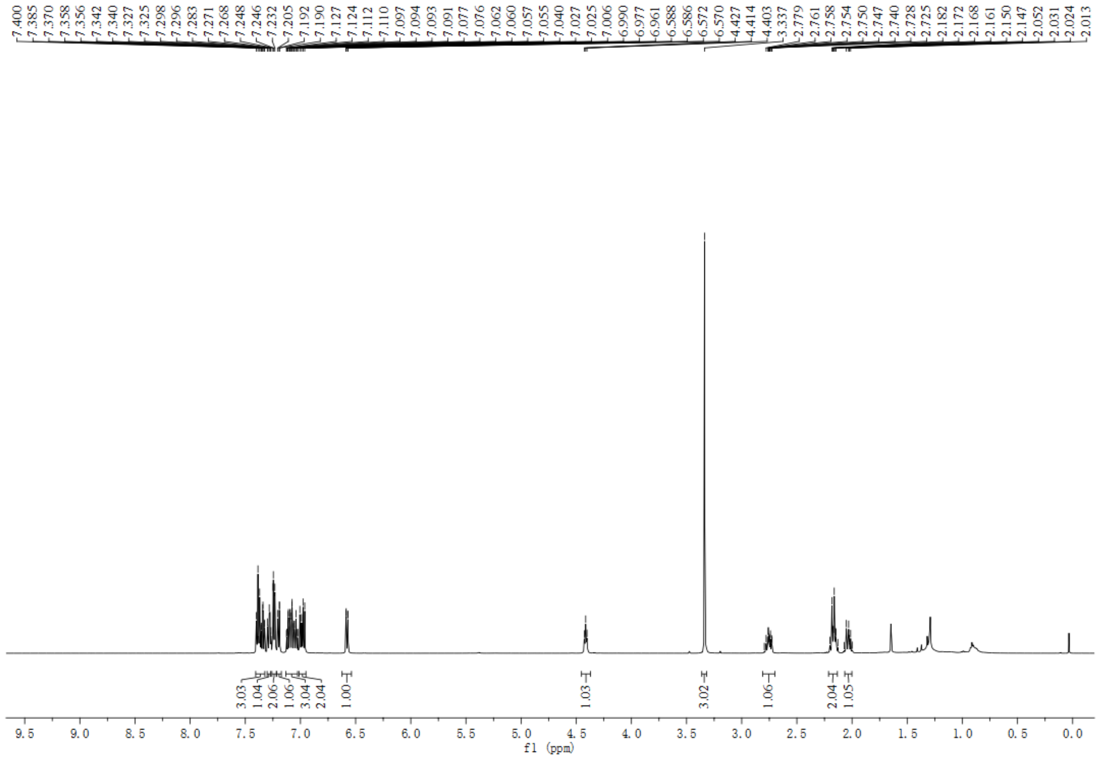

Supplementary Figure 183. ^{1}H NMR Spectra of compound **17**.

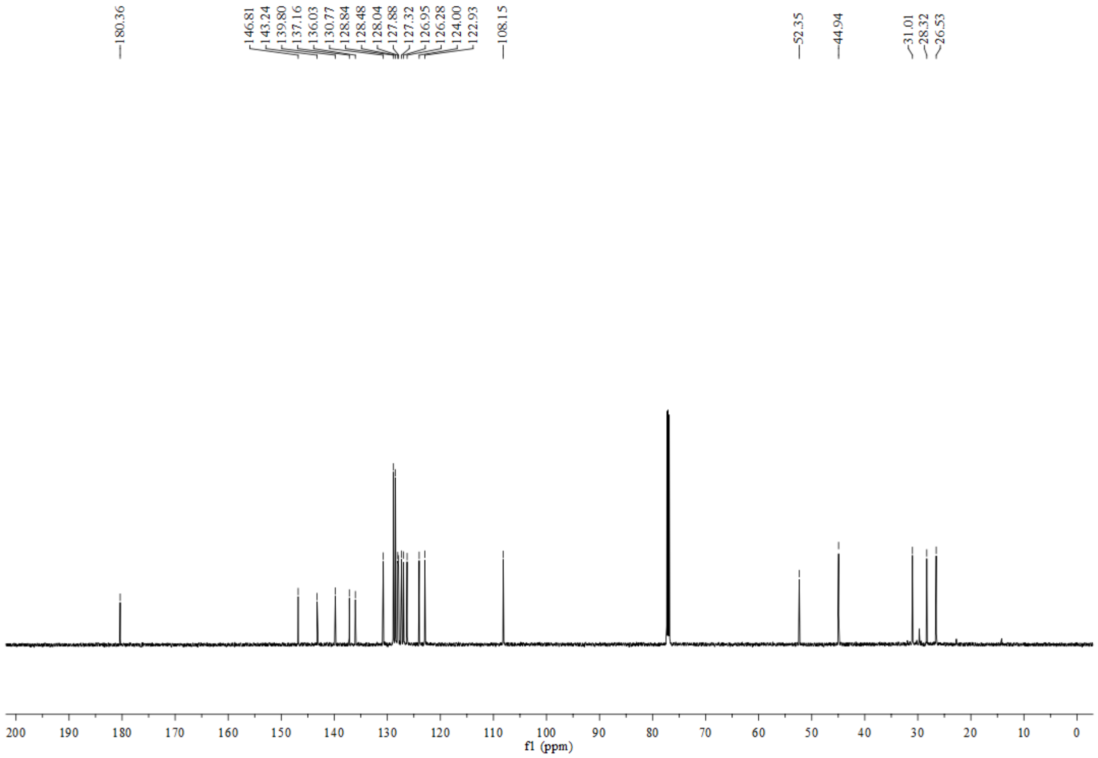

Supplementary Figure 184. ^{13}C NMR Spectra of compound **17**.

Crystal report of compound 3a (CCDC number: 1969160):

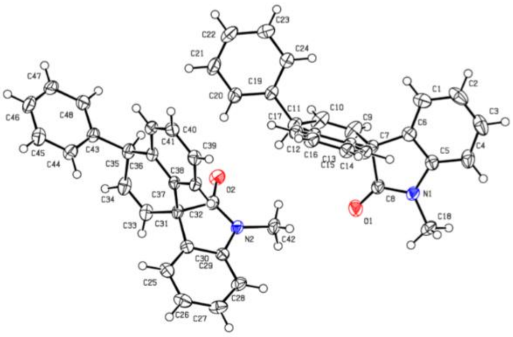

checkCIF/PLATON report

Structure factors have been supplied for datablock(s) 190718_zb_12_52b_0m

THIS REPORT IS FOR GUIDANCE ONLY. IF USED AS PART OF A REVIEW PROCEDURE FOR PUBLICATION, IT SHOULD NOT REPLACE THE EXPERTISE OF AN EXPERIENCED CRYSTALLOGRAPHIC REFEREE.

No syntax errors found.   CIF dictionary   Interpreting this report

Datablock: 190718_zb_12_52b_0m

```
Bond precision:   C-C = 0.0031 A          Wavelength=0.71073

Cell:          a=7.8502(4)       b=10.4336(7)      c=23.1499(15)
               alpha=93.066(2)   beta=91.965(2)    gamma=111.358(2)
Temperature:   170 K
```

	Calculated	Reported
Volume	1760.42(19)	1760.41(19)
Space group	P -1	P -1
Hall group	-P 1	-P 1
Moiety formula	C24 H19 N O	C24 H19 N O
Sum formula	C24 H19 N O	C24 H19 N O
Mr	337.40	337.40
Dx,g cm-3	1.273	1.273
Z	4	4
Mu (mm-1)	0.077	0.077
F000	712.0	712.0
F000'	712.28	
h,k,lmax	10,13,29	10,13,29
Nref	7799	7748
Tmin,Tmax	0.976,0.985	0.675,0.746
Tmin'	0.973	

```
Correction method= # Reported T Limits: Tmin=0.675 Tmax=0.746
AbsCorr = MULTI-SCAN

Data completeness= 0.993           Theta(max)= 27.132

R(reflections)= 0.0543( 6913)      wR2(reflections)= 0.1577( 7748)

S = 1.073                  Npar= 472
```

The following ALERTS were generated. Each ALERT has the format
test-name_ALERT_alert-type_alert-level.
Click on the hyperlinks for more details of the test.

Alert level C

```
PLAT911_ALERT_3_C Missing FCF Refl Between Thmin & STh/L=    0.600          27 Report
```

Alert level G

```
PLAT154_ALERT_1_G The s.u.'s on the Cell Angles are Equal ..(Note)      0.002 Degree
PLAT793_ALERT_4_G Model has Chirality at C7            (Centro SPGR)        S Verify
PLAT793_ALERT_4_G Model has Chirality at C11           (Centro SPGR)        R Verify
PLAT793_ALERT_4_G Model has Chirality at C31           (Centro SPGR)        R Verify
PLAT793_ALERT_4_G Model has Chirality at C35           (Centro SPGR)        S Verify
PLAT870_ALERT_4_G ALERTS Related to Twinning Effects Suppressed ..          ! Info
PLAT910_ALERT_3_G Missing # of FCF Reflection(s) Below Theta(Min).          4 Note
PLAT912_ALERT_4_G Missing # of FCF Reflections Above STh/L=  0.600         21 Note
PLAT913_ALERT_3_G Missing # of Very Strong Reflections in FCF ....          1 Note
PLAT931_ALERT_5_G CIFcalcFCF  Twin Law ( 0 0 1)          Est.d BASF      0.10 Check
PLAT933_ALERT_2_G Number of OMIT Records in Embedded .res File ...          5 Note
```

```
   0 ALERT level A = Most likely a serious problem - resolve or explain
   0 ALERT level B = A potentially serious problem, consider carefully
   1 ALERT level C = Check. Ensure it is not caused by an omission or oversight
  11 ALERT level G = General information/check it is not something unexpected

   1 ALERT type 1 CIF construction/syntax error, inconsistent or missing data
   1 ALERT type 2 Indicator that the structure model may be wrong or deficient
   3 ALERT type 3 Indicator that the structure quality may be low
   6 ALERT type 4 Improvement, methodology, query or suggestion
   1 ALERT type 5 Informative message, check
```

It is advisable to attempt to resolve as many as possible of the alerts in all categories. Often the minor alerts point to easily fixed oversights, errors and omissions in your CIF or refinement strategy, so attention to these fine details can be worthwhile. In order to resolve some of the more serious problems it may be necessary to carry out additional measurements or structure refinements. However, the purpose of your study may justify the reported deviations and the more serious of these should normally be commented upon in the discussion or experimental section of a paper or in the "special_details" fields of the CIF. checkCIF was carefully designed to identify outliers and unusual parameters, but every test has its limitations and alerts that are not important in a particular case may appear. Conversely, the absence of alerts does not guarantee there are no aspects of the results needing attention. It is up to the individual to critically assess their own results and, if necessary, seek expert advice.

Publication of your CIF in IUCr journals

A basic structural check has been run on your CIF. These basic checks will be run on all CIFs submitted for publication in IUCr journals (*Acta Crystallographica*, *Journal of Applied Crystallography*, *Journal of Synchrotron Radiation*); however, if you intend to submit to *Acta Crystallographica Section C* or *E* or *IUCrData*, you should make sure that full publication checks are run on the final version of your CIF prior to submission.

Publication of your CIF in other journals

Please refer to the *Notes for Authors* of the relevant journal for any special instructions relating to CIF submission.

DFT Calculations

Computational Details

All DFT calculations were performed using Gaussian 09 program.[4] Geometry optimizations were carried out using B3LYP[5-7]-D3 (Becke-Johnson damping function)[8,9] functional, with def2-SVP basis set[10] for all elements. The vibrational frequency was calculated at the same level of theory to identify each optimized stationary point as an energy minimum or a transition state, and to evaluate the zero-point vibrational energy and thermal corrections at 298 K. Based on the gas-phase optimized structures, the single-point energies and solvent effects of toluene were calculated using M06-L functional[11] and def2-TZVP basis set[12] for all elements, using the SMD solvent model.[13] For comparison, we also verified the free energy profile using wB97XD functional[14]. The 3D diagrams of computed species were generated by CYLView.[15] In order to adjust the Gibbs free energies from 1 atm to 1 mol/L, a correction of $RT\ln(c_s/c_g)$ (1.9 kcal/mol) is added to energies of all species. c_s is the standard molar con-centration in solution (1 mol/L), c_g is standard molar con-centration in gas phase (0.0446 mol/L), and R is the gas constant.

Alternative Reaction Pathways

$$PhB(OH)_2 + F^- \xrightarrow{\Delta G = -29.6 \text{ kcal/mol}} PhB(OH)_2F^-$$

Supplementary Figure 185. The DFT-computed reaction free energy of phenylboronic acid with fluoride anion.

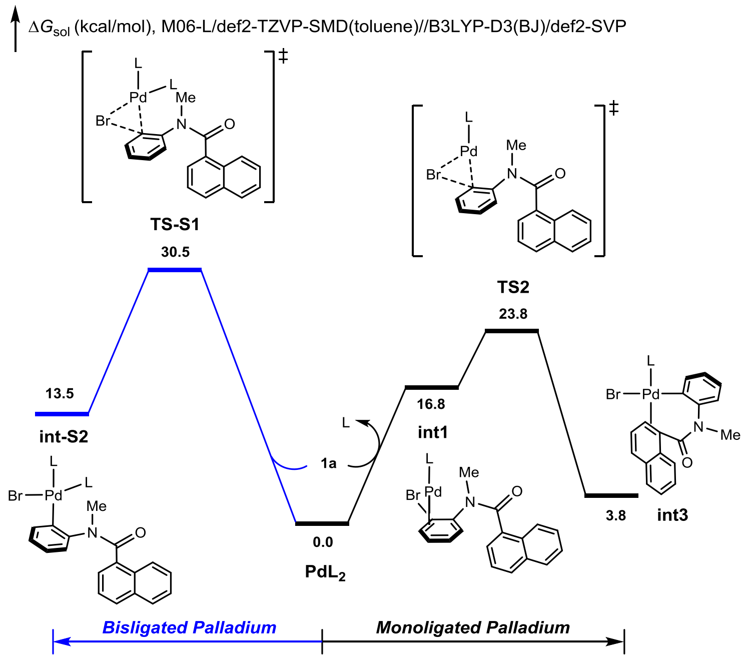

Supplementary Figure 186. Oxidative addition of **1a** with bisligated *vs* monoligated palladium.

The oxidative addition of **1a** with bisligated palladium is also considered, which is less favorable by 6.7 kcal/mol than that with monoligated palladium.

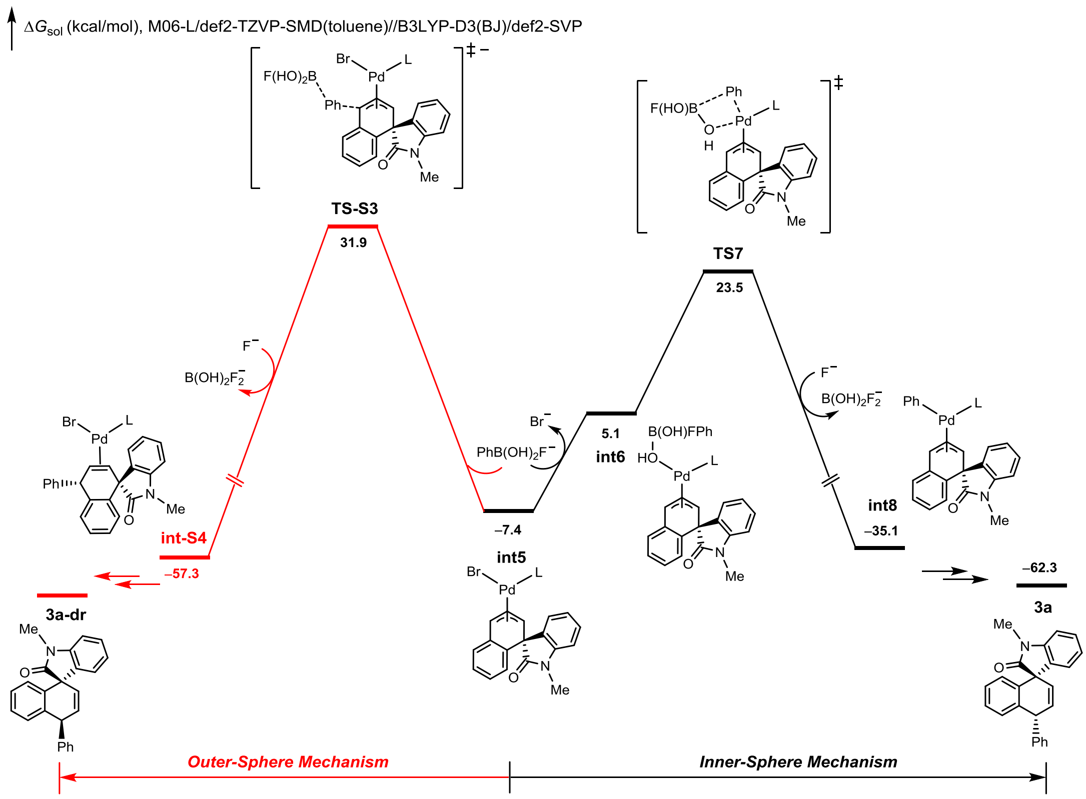

Supplementary Figure 187. Outer-sphere transmetallation *vs* inner-sphere transmetallation.

For the transmetallation, both inner-sphere and outer-sphere mechanism are considered. The outer-sphere pathway, which leads to the diastereoisomer **3a-dr**, requires a barrier of 31.9 kcal/mol. This controls the diastereoselectivity and favors the formation of **3a**.

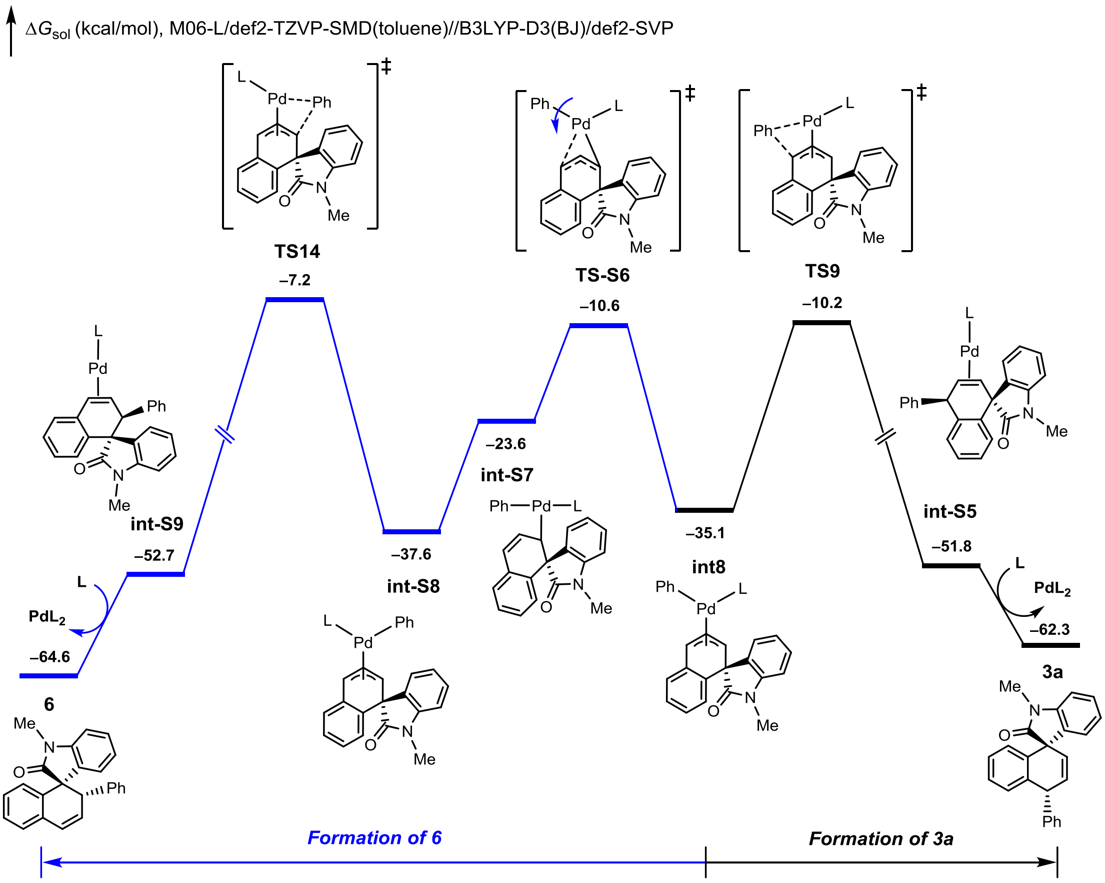

Supplementary Figure 188. DFT-computed free energy profiles for the formation of regio-divergent products (**3a** *vs* **6**).

From **int8**, direct reductive elimination via **TS9** leads to the formation of 1,4-dicarbofunctionalization product **3a**. Alternatively, isomerization via **TS-S6** and subsequent reductive elimination via **TS14** generates the 1,2-dicarbofunctionalization product **6** (the blue pathway). Comparing the two reductive elimination transition states, the 1,4-dicarbofunctionalization is more favorable than the 1,2-dicarbofunctionalization.

We calculated the reductive elimination transition states **TS-S10** and **TS-S11**, where the spiro scaffold is replaced by methylene. The regioselectivity is reversed with 4.9 kcal/mol preference for the 1,2-dicarbofunctionalization. This is because the

1,2-dicarbofunctionalization product has the conjugation between the phenyl group and the forming double bond. Therefore, the intrinsic regioselectivity favors the 1,2-dicarbofunctionalization, and the steric repulsions of spiro scaffold overrules the intrinsic selectivity and favors the 1,4-dicarbofunctionalization.

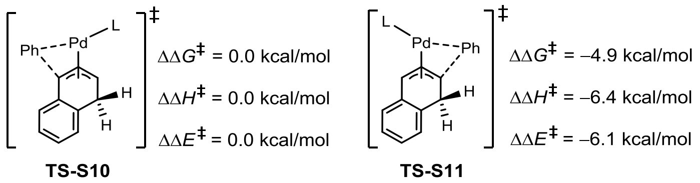

Supplementary Figure 189. Regioselectivity of truncated model.

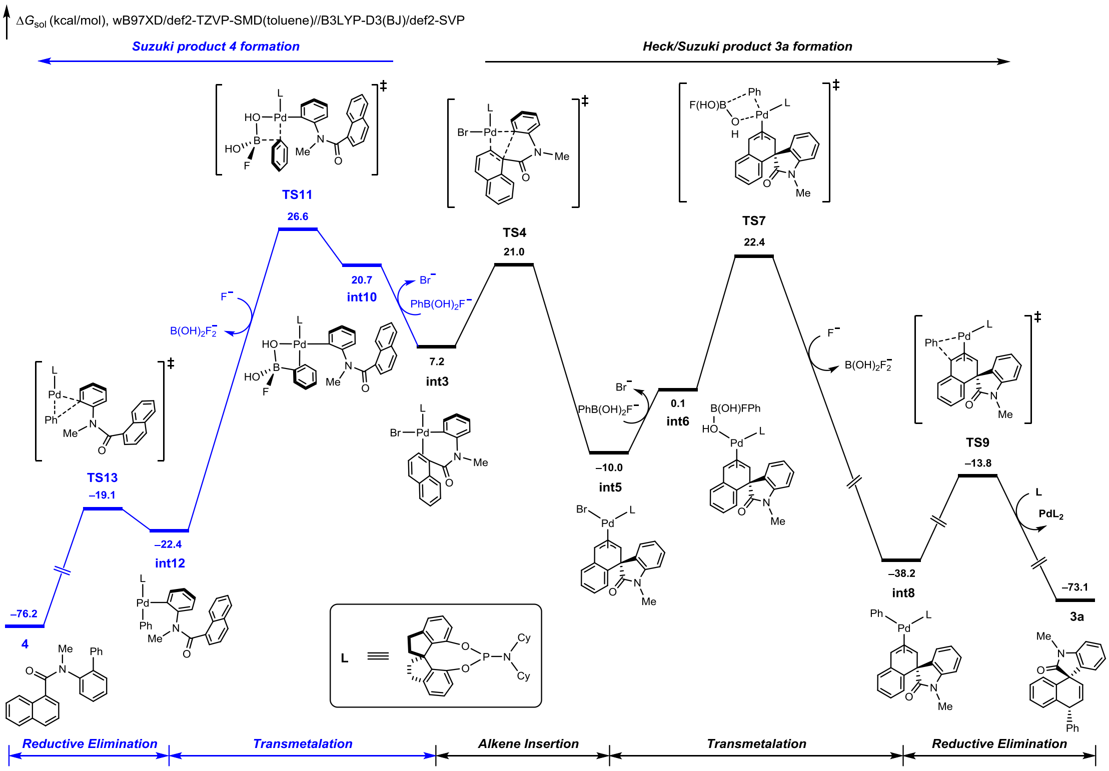

Supplementary Figure 190. DFT-computed free energy profiles of the most favorable pathway for the formation of **3a** and by-product **4** (**L** = **L5**). The single-point energies and solvent effects of toluene were calculated using wB97XD functional.

The same chemoselectivity was identified using wB97XD functional and M06-L functional.

Supplementary Table 1. Zero-point correction (*ZPE*), thermal correction to enthalpy

(*TCH*), thermal correction to Gibbs free energy (*TCG*), energies(*E*), enthalpies (*H*), and Gibbs free energies (*G*) (in Hartree) of the structures for all the figures calculated at the M06-L/def2-TZVP-SMD(toluene)//B3LYP-D3(BJ)/def2-SVP level of theory.

Structures	*ZPE*	*TCH*	*TCG*	*E*	*H*	*G*	Imaginary Frequency
**PdL2**	1.199813	1.259801	1.105707	-3475.535752	-3474.27595	-3474.430045	
**L**	0.598555	0.627209	0.540963	-1673.717849	-1673.09064	-1673.176886	
**1a**	0.273505	0.29204	0.225621	-3398.607028	-3398.31499	-3398.381407	
**Int1**	0.873436	0.923642	0.78642	-5200.394134	-5199.47049	-5199.607714	
**TS2**	0.873052	0.922629	0.787027	-5200.38365	-5199.46102	-5199.596623	-121.79*i*
**Int3**	0.875768	0.925179	0.793782	-5200.422335	-5199.49716	-5199.628553	
**TS4**	0.873611	0.922529	0.792061	-5200.39736	-5199.47483	-5199.605299	-376.75*i*
**Int5**	0.876568	0.925536	0.794558	-5200.440856	-5199.51532	-5199.646298	
**Int6**	1.005104	1.062253	0.915873	-3134.610961	-3133.54871	-3133.695088	
**TS7**	1.004284	1.061025	0.916686	-3134.582514	-3133.52149	-3133.665828	-203.79*i*
**Int8**	0.965616	1.018011	0.880244	-2858.008838	-2856.99083	-2857.128594	
**TS9**	0.964808	1.016616	0.879841	-2857.968731	-2856.95212	-2857.08889	-277.15*i*
**Int10**	1.004594	1.061987	0.913625	-3134.587391	-3133.5254	-3133.673766	
**TS11**	1.002505	1.059833	0.912032	-3134.575419	-3133.51559	-3133.663387	-284.88*i*
**Int12**	0.964571	1.01763	0.877162	-2857.987659	-2856.97003	-2857.110497	
**TS13**	0.963482	1.016081	0.875169	-2857.982134	-2856.96605	-2857.106965	-216.48*i*
**3a**	0.365374	0.386434	0.315589	-1056.234375	-1055.84794	-1055.918786	
**F-anion**	0	0.00236	-0.014159	-99.935922	-99.933562	-99.950081	
**Br-anion**	0	0.00236	-0.016176	-2574.187237	-2574.18488	-2574.203413	
**PhB(OH)$_2$F-anion**	0.125247	0.135575	0.090212	-508.362278	-508.226703	-508.272066	
**PhB(OH)$_2$**	0.125476	0.13422	0.092679	-408.370448	-408.236228	-408.277769	
**B(OH)$_2$F$_2$-anion**	0.03644	0.041831	0.008859	-376.589492	-376.547661	-376.580633	
**4**	0.365052	0.386468	0.315695	-1056.252954	-1055.86649	-1055.937259	
**TS14**	0.964355	1.016298	0.880381	-2857.964477	-2856.94818	-2857.084096	-283.76*i*
**TS-S1**	1.475732	1.55412	1.362074	-6874.121822	-6872.5677	-6872.759748	-145.40*i*
**Int-S2**	1.479145	1.557178	1.367928	-6874.154904	-6872.59773	-6872.786976	
**TS-S3**	1.002296	1.061431	0.910047	-5708.762756	-5707.70133	-5707.852709	-407.04*i*
**Int-S4**	0.966716	1.020905	0.877768	-5432.242178	-5431.22127	-5431.36441	
**Int-S5**	0.966504	1.01867	0.880112	-2858.035269	-2857.0166	-2857.155157	
**TS-S6**	0.964938	1.016843	0.881203	-2857.970833	-2856.95399	-2857.08963	-199.44*i*

**Int-S7**	0.965167	1.017728	0.879841	-2857.990172	-2856.97244	-2857.110331	
**Int-S8**	0.965486	1.017926	0.880274	-2858.012879	-2856.99495	-2857.132605	
**Int-S9**	0.966102	1.018211	0.880543	-2858.037199	-2857.01899	-2857.156656	
**TS-S10**	0.850026	0.893977	0.772322	-2419.995656	-2419.10168	-2419.223334	-280.85*i*
**TS-S11**	0.849567	0.893478	0.774202	-2420.005409	-2419.11193	-2419.231207	-292.90*i*

Supplementary References

(1) Zhou, H., Wang, W.-H., Fu, Y., Xie, J.-H., Shi, W.-J., Wang, L.-X. & Zhou, Q.-L. Highly enantioselective copper-catalyzed conjugate addition of diethylzinc to enones using chiral spiro phosphoramidites as ligands. *J. Org. Chem.* **68**, 1582-1584 (2003).

(2) Iwasaki, H., Eguchi, T., Tsutsui, N., Ohno, H. & Tanaka, T. Samarium(II)-mediated spirocyclization by intramolecular aryl radical addition onto an aromatic ring. *J. Org. Chem.* **73**, 7145-7152 (2008).

(3) Yang, L., Zheng, H., Luo, L., Nan, J., Liu, J., Wang, Y. & Luan, X. Palladium-catalyzed dynamic kinetic asymmetric transformation of racemic biaryls: axial-to-central chirality transfer. *J. Am. Chem. Soc.* **137**, 4876-4879 (2015).

(4) Frisch, M. J., Trucks, G. W., Schlegel, H. B., Scuseria, G. E., Robb, M. A., Cheeseman, J. R., Scalmani, G., Barone, V., Petersson, G. A., Nakatsuji, H., Li, X., Caricato, M., Marenich, A. V., Bloino, J., Janesko, B. G., Gomperts, R., Mennucci, B., Hratchian, H. P., Ortiz, J. V., Izmaylov, A. F., Sonnenberg, J. L., Williams-Young, D., Ding, F., Lipparini, F., Egidi, F., Goings, J., Peng, B., Petrone, A., Henderson, T., Ranasinghe, D., Zakrzewski, V. G., Gao, J., Rega, N., Zheng, G., Liang, W., Hada, M., Ehara, M., Toyota, K., Fukuda, R., Hasegawa, J., Ishida, M., Nakajima, T., Honda, Y., Kitao, O., Nakai, H., Vreven, T., Throssell, K., Montgomery, J. A. Jr., Peralta, J. E., Ogliaro, F., Bearpark, M. J., Heyd, J. J., Brothers, E. N., Kudin, K. N., Staroverov, V. N., Keith, T. A., Kobayashi, R., Normand, J., Raghavachari, K., Rendell, A. P., Burant, J. C., Iyengar, S. S., Tomasi, J., Cossi, M., Millam, J. M., Klene, M., Adamo, C., Cammi, R., Ochterski, J. W., Martin, R. L., Morokuma, K., Farkas, O., Foresman, J. B., & Fox, D. J., Gaussian 09, revision C.01; Gaussian Inc.: Wallingford, CT, **2016**.

[5] Lee, C., Yang, W. & Parr, R. G. Development of the Colle-Salvetti correlation-energy formula into a functional of the electron density. *Phys. Rev. B.* **37**, 785-789 (1988).

[6] Becke, A. D. Density‐functional thermochemistry. III. The role of exact exchange. *J. Chem. Phys.* **98**, 5648-5652 (1993).

[7] Stephens, P. J., Devlin, F. J., Chabalowski, C. F. & Frisch, M. J. Ab Initio calculation of vibrational absorption and circular dichroism spectra using density functional force fields. *J. Phys. Chem.* **98**, 11623-11627 (1994).

[8] Grimme, S., Antony, J., Ehrlich, S. & Krieg, H. A consistent and accurate ab initio parametrization of density functional dispersion correction (DFT-D) for the 94 elements H-Pu. *J. Chem. Phys.* **132**, 154104 (2010).

[9] Grimme, S., Ehrlich, S. & Goerigk, L. Effect of the damping function in dispersion corrected density functional theory. *J. Comput. Chem.* **32**, 1456-1465 (2011).

[10] Schäfer, A., Horn, H. & Ahlrichs, R. Fully optimized contracted Gaussian basis sets for atoms Li to Kr. *J. Chem. Phys.* **97**, 2571-2577 (1992).

[11] Zhao, Y. & Truhlar, D. G. A new local density functional for main-group thermochemistry, transition metal bonding, thermochemical kinetics, and noncovalent interactions. *J. Chem. Phys.* **125**, 194101 (2006).

[12] Weigend, F. & Ahlrichs, R. Balanced basis sets of split valence, triple zeta valence and quadruple zeta valence quality for H to Rn: Design and assessment of accuracy. *Phys. Chem. Chem. Phys.* **7**, 3297-3305 (2005).

[13] Marenich, A. V., Cramer, C. J. & Truhlar, D. G. Universal solvation model based on solute electron density and on a continuum model of the solvent defined by the bulk dielectric constant and atomic surface tensions. *J. Phys. Chem. B* **113**, 6378-6396 (2009).

[14] Chai, J.-D. & Head-Gordon, M. Long-range corrected hybrid density functionals with damped atom–atom dispersion corrections. *Phys. Chem. Chem. Phys.* **10**, 6615-6620 (2008).

[15] Legault, C. Y., CYLview, 1.0b, Université de Sherbrooke, **2009** (http://www.cylview.org)
