# Supplementary material for: Dearomative 1,4-difunctionalization of naphthalenes via palladium-catalyzed tandem Heck/Suzuki coupling reaction
Source: Nat Commun. 2020 Sep 1;11:4380. doi: 10.1038/s41467-020-18137-w (PMC7463262; doi:10.1038/s41467-020-18137-w)
Supplement: Supplementary file 3 — Description of Additional Supplementary Files [file 41467_2020_18137_MOESM3_ESM.pdf]

Description of Additional Supplementary Files

File Name: Supplementary Data 1

Description: Cartesian coordinates of the calculated species in DFT calculation study
