# Supplementary material for: Dearomative 1,4-difunctionalization of naphthalenes via palladium-catalyzed tandem Heck/Suzuki coupling reaction
Source: Nat Commun. 2020 Sep 1;11:4380. doi: 10.1038/s41467-020-18137-w (PMC7463262; doi:10.1038/s41467-020-18137-w)
Supplement: Supplementary file 4 — Supplementary Data 1 [file 41467_2020_18137_MOESM4_ESM.zip › 251193_2_supp_4810474_qlccqz.docx]

**Cartesian coordinates of the calculated species**

**1a**

0 1

C -1.19147000 1.41176500 -2.14292800

C -2.49103900 1.66377000 -1.76297100

C -3.15091700 0.82303900 -0.82550000

C -2.45437700 -0.30800100 -0.28070300

C -1.09692100 -0.52928900 -0.67781800

C -0.49012200 0.31704300 -1.58795300

H -5.00936900 1.94111300 -0.83254600

H -0.69194600 2.05808900 -2.86805500

H -3.03627700 2.51559000 -2.17754600

C -4.48773100 1.07755200 -0.41173900

C -3.13474300 -1.14085000 0.65269200

H 0.54488200 0.13674400 -1.88356000

C -4.42985500 -0.86149400 1.03478700

C -5.11500000 0.25737100 0.50020200

H -2.62466600 -2.02128200 1.04289700

H -4.93673300 -1.51384400 1.74987100

H -6.14209900 0.46403800 0.81012100

C -0.33566000 -1.70801400 -0.13415000

N 0.87922600 -1.41744900 0.46531400

O -0.74538800 -2.85184400 -0.23829800

C 1.68480600 -2.52657200 0.96809000

H 1.43223500 -3.42503100 0.39345700

H 2.75212300 -2.29573600 0.85195700

H 1.47189100 -2.71858400 2.03409600

C 1.18627600 -0.11388500 0.94265300

C 2.29052500 0.61185200 0.46346300

C 0.38229700 0.47754200 1.92826400

C 2.58663700 1.88443000 0.95651400

C 0.65902500 1.75452000 2.41358700

C 1.76677000 2.45626900 1.93127200

H 3.44753700 2.42662800 0.56323900

H 0.01151600 2.19919900 3.17203300

H 1.99582800 3.45526000 2.30861800

H -0.47890100 -0.08232800 2.29779700

Br 3.37147100 -0.10529300 -0.93199300

**3a**

0 1

C -1.03796900 -0.90349600 -1.44308600

C -1.81531500 -0.77947400 -0.15458100

C -1.13732100 0.18364300 0.81791500

C 0.23897900 0.45373200 0.72497400

C 1.09002000 -0.25693200 -0.32679700

C 0.26844900 -0.65226800 -1.53213700

H -2.95131200 0.60302600 1.90337600

H -1.59301200 -1.23783400 -2.32370300

C -1.88042700 0.80134200 1.83419900

C 0.83958100 1.32528600 1.64440600

H 0.80615400 -0.78409600 -2.47417900

C 0.09036200 1.93041800 2.65255600

C -1.27742100 1.66461000 2.74843500

H 1.90803300 1.53338300 1.56404200

H 0.57430000 2.60848700 3.35944100

H -1.87763600 2.13268400 3.53232300

C 1.65045300 -1.56039200 0.33721000

N 3.03278500 -1.47473400 0.32234800

O 1.00313000 -2.47501200 0.79125800

C 3.89554300 -2.49217800 0.87194300

H 3.25488400 -3.28741600 1.27473200

H 4.55493200 -2.91424500 0.09527100

H 4.52260800 -2.08396800 1.68196500

C 3.46882900 -0.29426200 -0.29199500

C 2.36302800 0.47139000 -0.70042900

C 4.77618100 0.13462300 -0.50985900

C 2.55576500 1.68349400 -1.34475500

C 4.95812700 1.36503600 -1.16033900

C 3.86700500 2.13297800 -1.57589400

H 1.69643700 2.28077400 -1.65852900

H 5.97346600 1.72470100 -1.34362700

H 4.03417500 3.08670400 -2.08076300

C -3.26693700 -0.42205500 -0.41883700

C -3.58774400 0.75156000 -1.11792400

C -4.30745000 -1.24462100 0.02664900

C -4.91745700 1.09360900 -1.36449600

H -2.78082300 1.40257600 -1.46445800

C -5.64272100 -0.90534000 -0.21729100

H -4.06848500 -2.16114600 0.57303200

C -5.95109400 0.26480100 -0.91368200

H -5.15098800 2.01147600 -1.90987600

H -6.44302600 -1.55890300 0.13859000

H -6.99304100 0.53216400 -1.10556400

H 5.63148000 -0.46205100 -0.18911900

H -1.79547900 -1.77611500 0.32343100

**4**

0 1

C -0.27739500 -3.02978500 -0.00323400

C -1.58941800 -2.85250600 -0.37928800

C -2.30423700 -1.68506700 0.00392900

C -1.64753400 -0.67669600 0.78770000

C -0.26282400 -0.86601600 1.12121500

C 0.38473200 -2.02868600 0.74311900

H -4.14465300 -2.27193900 -0.97984300

H 0.26263700 -3.93652800 -0.28451900

H -2.10598800 -3.61270200 -0.97113900

C -3.66057000 -1.49798400 -0.37841500

C -2.39560400 0.47205800 1.16853700

H 1.42859700 -2.17949800 1.01675900

C -3.71243800 0.61720900 0.78756500

C -4.35378500 -0.37040300 0.00329000

H -1.91213900 1.23241400 1.77680500

H -4.26542600 1.50955900 1.09040300

H -5.39706400 -0.23855900 -0.29365800

C 0.46567500 0.18435600 1.91038700

N 1.74318700 0.53962000 1.51360600

O -0.02857400 0.71531500 2.89524600

C 2.41837900 1.50537000 2.37908500

H 1.80931400 2.41815300 2.47574400

H 2.55900400 1.10144600 3.39327500

H 3.39108700 1.75768600 1.94139400

C 2.40087600 0.11302400 0.32238000

C 3.63361900 -0.54469800 0.44879200

C 1.84842900 0.33142500 -0.95972300

C 4.31866200 -1.01193900 -0.67092200

H 4.03697400 -0.71031000 1.45010200

C 2.54316100 -0.17351400 -2.07283700

C 3.76223800 -0.83613700 -1.94060800

H 5.27585900 -1.52426500 -0.55031300

H 2.12084400 -0.00815900 -3.06612400

H 4.28276700 -1.20318900 -2.82793200

C 0.56866900 1.05342600 -1.17013300

C -0.42469200 0.49618200 -1.99219900

C 0.32685500 2.30340800 -0.57641100

C -1.62787300 1.16591400 -2.21304500

H -0.26477700 -0.49324400 -2.42518900

C -0.87429600 2.97633900 -0.80261700

H 1.09073500 2.75536200 0.05826300

C -1.85589300 2.40887300 -1.61933200

H -2.40049100 0.70283600 -2.83023900

H -1.04599800 3.94836400 -0.33429100

H -2.80340400 2.92778100 -1.78012100

**6**

0 1

C 1.17212500 -1.18455000 -2.14990600

C 2.35482700 -1.29101100 -1.52936400

C 2.54655200 -0.84107100 -0.14915800

C 1.54929100 -0.06112100 0.48083000

C 0.37942000 0.43900200 -0.35678500

C -0.03869600 -0.59628600 -1.47017300

H 4.45205000 -1.81932600 0.08995400

H 1.03296400 -1.57869200 -3.15957900

C 3.68478500 -1.21446600 0.58042600

C 1.71409600 0.31597700 1.81435900

C 2.85105100 -0.06954100 2.53428900

C 3.84073400 -0.83354600 1.91460500

H 0.94735800 0.92234800 2.29915700

H 2.96110800 0.23463600 3.57777400

H 4.73317400 -1.13442000 2.46842900

C 0.82540200 1.74313200 -1.09787600

N -0.10888800 2.72599700 -0.80589500

O 1.78843200 1.89352500 -1.81277700

C -0.02920500 4.06930600 -1.32496900

H 0.85251900 4.11980200 -1.97692900

H -0.93016400 4.32361600 -1.90777900

H 0.07932800 4.80328600 -0.50886700

C -1.06725500 2.27102100 0.10437100

C -0.81649100 0.92745600 0.43481700

C -2.12896700 2.98228100 0.65955400

C -1.63177400 0.28320100 1.35660900

C -2.94950800 2.31438600 1.58071100

C -2.70430300 0.98447800 1.93144900

H -1.44052400 -0.75399800 1.63331800

H -3.78893700 2.84857300 2.03237800

H -3.35106700 0.48360600 2.65467600

H -2.31456700 4.02398400 0.39352600

H 3.20418300 -1.76172900 -2.03144700

C -0.95830700 -1.68640800 -0.93925100

C -0.45879500 -2.75049100 -0.17404600

C -2.33962000 -1.60735100 -1.15901100

C -1.32534500 -3.69610700 0.37813000

H 0.61536800 -2.83395300 -0.00478600

C -3.20860100 -2.55737700 -0.61763100

H -2.74059900 -0.77687700 -1.74572800

C -2.70365000 -3.60238500 0.15981000

H -0.92122700 -4.51407000 0.97968200

H -4.28318600 -2.47676700 -0.79856700

H -3.38016600 -4.34493700 0.58987400

H -0.61944500 -0.02642300 -2.21234800

**B(OH)_2_F_2_-anion**

-1 1

B -0.03388000 -0.00000300 0.00003400

O 0.78514700 1.22281400 0.00049800

H 1.71391800 0.97782700 -0.00097900

O 0.78512300 -1.22282800 0.00039000

H 1.71389800 -0.97785200 0.00029800

F -0.87941600 0.00001700 1.14417800

F -0.87842500 -0.00000100 -1.14491200

**Br-anion**

-1 1

Br 0.00000000 0.00000000 0.00000000

**F-anion**

-1 1

F 0.00000000 0.00000000 0.00000000

**int-S2**

0 1

C -0.06933100 2.32412000 1.87065400

C 0.36524300 1.40559600 2.83138900

H 1.12635900 0.67960800 2.55289000

C -0.19992400 1.40863900 4.10677800

H 0.13293400 0.67192300 4.83492800

C -1.22101900 2.30832800 4.42418800

H -1.70167600 2.27760900 5.40473800

C -1.64485900 3.21722200 3.45937300

C -2.78870300 4.19720800 3.55247200

H -3.65461800 3.78120600 4.08953600

H -2.48421800 5.11151500 4.09256300

C -3.07930700 4.49157000 2.07456600

H -3.57186500 5.45794300 1.89847900

H -3.72958900 3.70504300 1.66097900

C -1.68385900 4.39179800 1.36902000

C -1.05646600 3.26391500 2.17911700

C -0.93818700 5.75448900 1.49763000

H 0.12579200 5.59955800 1.25642600

H -0.99133500 6.17044100 2.51384200

C -1.60164600 6.64918300 0.42709500

H -0.91818200 7.41234500 0.02233400

H -2.46929900 7.19251100 0.83906000

C -2.03395100 5.65209900 -0.62805500

C -2.50059700 5.88450900 -1.92161400

H -2.61456400 6.90422700 -2.29718700

C -2.82043200 4.78819200 -2.73420300

H -3.21095300 4.94997400 -3.74150500

C -2.58570700 3.48440200 -2.28951200

H -2.77185900 2.61672500 -2.92251400

C -2.08177600 3.26799100 -1.00174300

C -1.89758200 4.34287300 -0.13372500

O -1.73557200 1.99131600 -0.62579200

O 0.49182900 2.26407800 0.61143300

P -0.16244100 1.41822800 -0.66065000

N 0.67462500 2.25127300 -1.85289600

C 1.00737600 1.68192700 -3.17318500

C 1.64436000 0.29701000 -3.10757000

C -0.17517500 1.70345500 -4.15056500

H 1.78066500 2.34907700 -3.58256800

C 2.07613600 -0.17012400 -4.49833300

H 0.92509600 -0.41880100 -2.68805000

H 2.49348800 0.31041100 -2.41382600

C 0.22650600 1.19310700 -5.53786700

H -0.98191800 1.07770700 -3.73912800

H -0.57537500 2.72406600 -4.21829600

C 0.89146300 -0.18482800 -5.46821100

H 2.54127100 -1.16556800 -4.43360000

H 2.85798600 0.50924400 -4.88536300

H -0.65617500 1.16530000 -6.19745800

H 0.93370500 1.90921200 -5.99597700

H 1.21597300 -0.50731500 -6.47100300

H 0.15334600 -0.93136700 -5.12294900

C 1.16325200 3.63771800 -1.60223900

C 2.65414700 3.65264300 -1.25192500

C 0.84418700 4.62583000 -2.73042400

H 0.62808100 4.00405400 -0.72261300

C 3.12206500 5.06472700 -0.88728600

H 3.23927700 3.28258700 -2.11321600

H 2.82804200 2.96104700 -0.41794000

C 1.30216900 6.03638900 -2.34157700

H 1.35859200 4.33372300 -3.66145700

H -0.23303000 4.61909300 -2.93380700

C 2.79276900 6.07283700 -1.99215100

H 4.20279700 5.06031800 -0.67045700

H 2.61930300 5.37482500 0.04720000

H 1.07628700 6.73974000 -3.15964400

H 0.71444600 6.37577100 -1.47126100

H 3.09295600 7.08953700 -1.68884400

H 3.38271800 5.82845300 -2.89477800

Pd -0.39310300 -0.90519500 -0.28740300

C -5.68489700 -4.06604100 -0.84902700

C -7.05535300 -3.93233500 -0.86057200

C -7.69252800 -2.94525400 -0.06027800

C -6.89408600 -2.09147600 0.77377100

C -5.46935500 -2.23797600 0.74034100

C -4.88979000 -3.20849500 -0.05493300

H -9.70272900 -3.43682900 -0.70829900

H -5.19841500 -4.82799000 -1.46259100

H -7.67488900 -4.58166300 -1.48497600

C -9.10548400 -2.78027300 -0.06976100

C -7.55502900 -1.11078000 1.56774400

H -3.80359500 -3.30483400 -0.07636400

C -8.92673800 -0.97460500 1.52956100

C -9.71280900 -1.81560000 0.70374400

H -6.95829800 -0.48263500 2.22848400

H -9.41378200 -0.21683300 2.14839100

H -10.79922400 -1.69896400 0.68578200

C -4.61231100 -1.36029700 1.61747400

N -3.62194100 -0.60768400 1.01388300

O -4.79744100 -1.33310900 2.82717700

C -2.71061700 0.11961500 1.88303000

H -3.10543200 0.07127500 2.90245300

H -1.70842500 -0.33531900 1.87360800

H -2.63206500 1.16155600 1.55595400

C -3.48598600 -0.49459700 -0.39813300

C -2.26826900 -0.70969300 -1.07090300

C -4.62716000 -0.16300800 -1.14998100

C -2.27411800 -0.75218200 -2.47020700

C -4.61056700 -0.16398800 -2.54298200

C -3.43217300 -0.49532500 -3.21243100

H -1.35257600 -0.98826300 -3.00538400

H -5.52013400 0.07846800 -3.09698600

H -3.40001500 -0.53795500 -4.30430000

H -5.54870100 0.07695800 -0.61860100

Br -1.23597800 -3.09198200 0.59217500

C 2.22969100 -1.96885600 2.74449600

C 1.12749300 -2.60266400 3.33010000

H 0.54841300 -3.30628900 2.73250500

C 0.76897900 -2.29751400 4.64383400

H -0.09163300 -2.79820900 5.09253000

C 1.47303300 -1.32451300 5.36405800

H 1.16409200 -1.05433400 6.37709600

C 2.54265400 -0.67228800 4.75391300

C 3.35546100 0.49372100 5.26867500

H 2.72385300 1.26074600 5.74318700

H 4.08941400 0.16921300 6.02704500

C 4.05093700 1.00281600 3.99192200

H 4.99660000 1.52980000 4.18150700

H 3.38294100 1.69452800 3.45608000

C 4.24142000 -0.27921200 3.11553400

C 2.96136900 -1.02143900 3.45651700

C 5.49831300 -1.06112500 3.60798100

H 5.45163200 -2.08354700 3.20074900

H 5.54191500 -1.13837700 4.70324900

C 6.68305200 -0.30005300 2.98909000

H 7.56204200 -0.93828500 2.80895800

H 7.01842800 0.52440800 3.64210800

C 6.09573500 0.23312600 1.70191300

C 6.77903400 0.76929100 0.61363500

H 7.86634900 0.87275800 0.63820800

C 6.04996300 1.16191100 -0.51482900

H 6.55836400 1.59958800 -1.37665400

C 4.67684900 0.93540800 -0.56894600

H 4.10964800 1.14984000 -1.47189400

C 4.00164700 0.36129200 0.51845400

C 4.69005000 0.09341900 1.70612900

O 2.67012700 0.04083500 0.33701900

O 2.58032500 -2.27602800 1.44572800

P 1.95346900 -1.45542500 0.12596600

N 2.77366600 -2.29967500 -1.07948800

C 2.12345700 -3.47798000 -1.73193400

C 1.76135700 -4.60389000 -0.75067100

C 0.93121400 -3.12243800 -2.62521600

H 2.90013800 -3.86773900 -2.40836800

C 1.21578900 -5.83177000 -1.48569700

H 0.99309600 -4.23651300 -0.05581100

H 2.63514200 -4.87504300 -0.14515600

C 0.37743100 -4.35621400 -3.34469500

H 0.12965000 -2.68006700 -2.01439300

H 1.24130100 -2.36956100 -3.35794600

C 0.01332200 -5.47051200 -2.36123100

H 0.93971000 -6.60736500 -0.75296100

H 2.01207600 -6.26920200 -2.11835000

H -0.50067900 -4.06520600 -3.94449200

H 1.13476400 -4.73070800 -4.05954600

H -0.34979700 -6.35807100 -2.90551000

H -0.81063400 -5.12480600 -1.71481500

C 4.25917100 -2.33551000 -1.07826800

C 4.88838300 -3.60837200 -0.49138300

C 4.81806200 -2.02565300 -2.47394000

H 4.59513200 -1.52710400 -0.42576500

C 6.41563400 -3.48125300 -0.46141300

H 4.61657400 -4.48775100 -1.09828500

H 4.48959600 -3.76982500 0.51997100

C 6.34939200 -1.95753500 -2.46912800

H 4.48860700 -2.80192800 -3.18633600

H 4.39298700 -1.07431900 -2.82630400

C 6.97768800 -3.21375100 -1.86039800

H 6.85942900 -4.39469100 -0.03285800

H 6.69724200 -2.64871800 0.20937900

H 6.71952200 -1.79629200 -3.49517100

H 6.66402700 -1.08066200 -1.87920300

H 8.07501000 -3.11252700 -1.82425800

H 6.76405000 -4.08405900 -2.50787800

**int-S4**

-1 1

C 1.07867900 -0.94200800 -2.31104800

C 0.11380700 -0.43441700 -3.19355900

H 0.14782900 0.62777300 -3.43992900

C -0.90752600 -1.25827100 -3.67155500

H -1.66579100 -0.84066200 -4.33817400

C -1.01745900 -2.58279900 -3.23058400

H -1.87001900 -3.19738000 -3.52936800

C -0.05017700 -3.08317300 -2.36088100

C -0.01735100 -4.42285600 -1.65738300

H -1.00788400 -4.69704700 -1.26080200

H 0.29652800 -5.23319000 -2.34017400

C 1.02332000 -4.18814800 -0.54365400

H 1.53864100 -5.10249600 -0.21175600

H 0.52996900 -3.73717500 0.32972400

C 2.00394100 -3.12576400 -1.12953600

C 1.03361200 -2.28930100 -1.94460700

C 3.06472700 -3.81111700 -2.04930400

H 3.47474600 -3.04513300 -2.72629200

H 2.62929100 -4.60820300 -2.66995400

C 4.16472600 -4.29892100 -1.08841700

H 5.16033200 -4.34781300 -1.55832900

H 3.94221500 -5.31318400 -0.70941600

C 4.09775500 -3.28478600 0.03213400

C 5.02963300 -3.04343000 1.04163000

H 5.95574700 -3.62194500 1.09599400

C 4.75540500 -2.04418400 1.98471200

H 5.46757700 -1.84161000 2.78914700

C 3.59567200 -1.27501900 1.88461300

H 3.37800500 -0.47127500 2.58647700

C 2.67640800 -1.49937600 0.84741000

C 2.90504100 -2.54670900 -0.05310900

O 1.58543000 -0.70501100 0.73008000

O 1.97355600 -0.08791200 -1.72286600

P 1.42801300 0.68162700 -0.27175800

N 2.86609700 1.55467800 -0.02331600

C 2.74300000 2.98030200 0.35177700

C 2.13416500 3.86534800 -0.74687100

C 1.99630200 3.16808700 1.68064700

H 3.77413700 3.33109800 0.52012900

C 2.10490800 5.33727700 -0.32464800

H 1.10270600 3.53387200 -0.94328500

H 2.70496000 3.73901100 -1.68012600

C 1.96499800 4.64036000 2.09998300

H 0.96357700 2.80453100 1.55798600

H 2.47378500 2.54088100 2.45194600

C 1.35792200 5.51793500 1.00048100

H 1.62166800 5.93639500 -1.11373700

H 3.14080300 5.71915100 -0.22152300

H 1.39508000 4.75038800 3.03846400

H 2.99655600 4.98149700 2.31792300

H 1.36870300 6.57843700 1.30812500

H 0.30479100 5.22932000 0.84298700

C 4.17844300 1.07584700 -0.48084900

C 4.60600600 1.63847300 -1.84768500

C 5.30394900 1.28374600 0.54473600

H 4.07726300 -0.00799900 -0.61842000

C 5.89171300 0.95755800 -2.32942900

H 4.77390600 2.72687900 -1.76501100

H 3.78792000 1.48393200 -2.56463000

C 6.58962000 0.59313100 0.07699800

H 5.50480100 2.36113000 0.67419200

H 4.98834200 0.89631900 1.52239200

C 7.02299100 1.09581800 -1.30449400

H 6.20388200 1.36968900 -3.30428600

H 5.68380900 -0.11544700 -2.49541500

H 7.39693100 0.75099500 0.81298900

H 6.41253600 -0.49586700 0.03474600

H 7.92264800 0.55519000 -1.64636800

H 7.30862400 2.16157500 -1.22650400

Pd -0.73332200 1.23910300 -0.38753100

C -2.61750600 0.19246400 -0.90326300

C -3.92923400 0.78566700 -0.46124600

C -4.22423000 0.56399400 1.00668500

C -3.46868600 -0.29825400 1.81206000

C -2.21631600 -1.01132700 1.30807500

C -1.83047000 -0.65163200 -0.12622000

H -5.91223500 1.89598000 0.93408400

H -2.49175300 0.14306000 -1.98649600

H -3.89309300 1.87354200 -0.63829700

C -5.33362900 1.22016500 1.56894000

C -3.85604200 -0.50261600 3.14908900

C -4.96613200 0.13707200 3.68980300

C -5.70809000 1.01723600 2.89240000

H -3.25843400 -1.16798300 3.77694600

H -5.24641500 -0.03905400 4.73168100

H -6.57453700 1.54140100 3.30452800

C -2.41591000 -2.55490900 1.32165100

N -1.42898700 -3.10294700 2.12876400

O -3.24639000 -3.20067600 0.71413600

C -1.31489400 -4.51225900 2.39546000

H -2.08640400 -5.02400600 1.80479700

H -0.32124700 -4.89380800 2.10738700

H -1.47350700 -4.72747300 3.46678400

C -0.64320600 -2.10315900 2.72999100

C -1.07719100 -0.84327600 2.29972300

C 0.40003200 -2.25089800 3.63960200

C -0.49401900 0.30709700 2.80321200

C 0.99654000 -1.07971300 4.13304700

C 0.55399700 0.18257800 3.72899600

H -0.84261300 1.28529300 2.46540600

H 1.82350200 -1.16393900 4.84276700

H 1.03385400 1.08064100 4.12396400

H 0.74658900 -3.23588400 3.95718000

Br -1.63382700 3.68242400 -0.73400000

H -1.18988500 -1.38558200 -0.61366300

C -5.05465100 0.20736500 -1.31478300

C -5.79782800 1.02888600 -2.17078700

C -5.34331100 -1.16725800 -1.27381600

C -6.81786900 0.49671200 -2.96818900

H -5.56556200 2.09666300 -2.21397800

C -6.36061400 -1.69775800 -2.06749700

H -4.75974400 -1.82185500 -0.62000900

C -7.10408500 -0.86896300 -2.91765200

H -7.38746900 1.15327600 -3.63220400

H -6.57499500 -2.76939500 -2.02409300

H -7.89994000 -1.28872100 -3.53939600

**int-S5**

0 1

C -1.70020900 -1.03972600 1.90671400

C -0.68468600 -1.14323100 2.86453300

H -0.22160100 -0.22861400 3.23703200

C -0.25308100 -2.40313400 3.28933600

H 0.54589300 -2.47534900 4.03113900

C -0.80463700 -3.56629200 2.73724200

H -0.43172400 -4.54992600 3.03289700

C -1.83106400 -3.44956900 1.80027800

C -2.56322000 -4.54014900 1.05244800

H -1.87700100 -5.30940700 0.66551300

H -3.28456200 -5.05198400 1.71437800

C -3.27497800 -3.76608600 -0.07404100

H -4.20531600 -4.24111700 -0.41731300

H -2.60001900 -3.68061800 -0.93468800

C -3.51638700 -2.33333300 0.50194900

C -2.31055500 -2.18882800 1.40846200

C -4.85678400 -2.30196700 1.29993800

H -4.84500300 -1.42657300 1.96856200

H -4.99388000 -3.19890000 1.92131600

C -5.94045800 -2.12168800 0.21883400

H -6.84096900 -1.60830900 0.59055700

H -6.27310400 -3.09546900 -0.18183200

C -5.21856200 -1.32229200 -0.84380400

C -5.76043400 -0.61872900 -1.91943200

H -6.83875800 -0.61623300 -2.09609600

C -4.89976200 0.08783000 -2.76739300

H -5.30173800 0.63476900 -3.62353300

C -3.53145700 0.14559400 -2.49427500

H -2.85444500 0.74315200 -3.10631500

C -2.99965700 -0.53619500 -1.39151500

C -3.83285100 -1.33743000 -0.60333300

O -1.67240800 -0.35822900 -1.08091200

O -2.04533900 0.19603700 1.39951000

P -1.13860800 0.75237300 0.08275500

N -1.98926000 2.18064900 -0.14769400

C -1.23045900 3.37980000 -0.54846200

C -0.18641700 3.83152200 0.48435000

C -0.60114800 3.23402100 -1.94143800

H -1.97100800 4.18998100 -0.61691200

C 0.50530400 5.12349500 0.04387800

H 0.57288400 3.03300500 0.60886900

H -0.66838800 3.95252400 1.46666300

C 0.09751000 4.52422200 -2.37949300

H 0.13043500 2.40628200 -1.91508700

H -1.38380400 2.94501300 -2.66097200

C 1.13344000 4.97795900 -1.34634100

H 1.26412200 5.41404200 0.78745900

H -0.23671800 5.94331800 0.02125100

H 0.57242300 4.38159900 -3.36375200

H -0.65888700 5.32024500 -2.51023000

H 1.59859900 5.92719500 -1.65915300

H 1.94511800 4.23151800 -1.30384400

C -3.40575500 2.33596500 0.24024600

C -3.57397300 2.98634000 1.62157400

C -4.25540800 3.07219100 -0.80409400

H -3.81247700 1.31946000 0.31545300

C -5.04818200 2.98731400 2.04139800

H -3.19699100 4.02465300 1.58641500

H -2.96110600 2.43799500 2.35124100

C -5.73245700 3.04742400 -0.39579400

H -3.93030600 4.12349100 -0.88887600

H -4.11599500 2.60811800 -1.78911200

C -5.93721500 3.66383900 0.99237400

H -5.16609700 3.47782800 3.02168400

H -5.37793600 1.94066000 2.17503100

H -6.34078600 3.57854700 -1.14647200

H -6.08273700 2.00016100 -0.39299900

H -6.99739800 3.60036100 1.28895600

H -5.68895700 4.74042800 0.94855500

Pd 1.09636000 0.65208700 0.45830600

C 3.00210200 -0.02635800 1.37171900

C 4.29529100 0.39812400 0.69996000

C 4.28636000 0.05808600 -0.78549200

C 3.55950900 -1.05507900 -1.25176600

C 2.78013700 -1.92333600 -0.27100100

C 2.29004400 -1.12579500 0.92768600

H 5.58608500 1.68222300 -1.34656100

H 2.87257700 0.31345600 2.40469400

H 5.08614100 -0.22806300 1.15168700

C 5.02540800 0.81687800 -1.70287700

C 3.59638100 -1.38251900 -2.61340400

C 4.33647200 -0.61915900 -3.51686600

C 5.05432900 0.48646200 -3.05840500

H 3.03086600 -2.24451300 -2.97017400

H 4.34945800 -0.88897700 -4.57554700

H 5.63775400 1.09448000 -3.75412200

C 3.72316000 -3.02497000 0.31950300

N 3.12356000 -4.25182200 0.09405800

O 4.77348800 -2.85013700 0.89679100

C 3.69738600 -5.50435500 0.51987800

H 4.63739900 -5.27895200 1.04019800

H 3.01820700 -6.03640200 1.20682700

H 3.90673200 -6.15849500 -0.34307500

C 1.92752800 -4.11614000 -0.62420100

C 1.68041000 -2.75866700 -0.88824100

C 1.06667700 -5.11740900 -1.06764900

C 0.56734100 -2.38156500 -1.61967800

C -0.05748600 -4.72370400 -1.81196400

C -0.30453100 -3.37666800 -2.08998900

H 0.36984100 -1.33049600 -1.82288700

H -0.74626600 -5.48826100 -2.17998600

H -1.17898000 -3.08839600 -2.67636700

H 1.26143800 -6.17044600 -0.85855700

H 1.61501500 -1.66630500 1.59367800

C 4.63524200 1.84356700 0.99964400

C 5.78262100 2.17115400 1.73050900

C 3.80317800 2.88246400 0.55673600

C 6.09584600 3.50433200 2.01734400

H 6.44025600 1.36961100 2.07764700

C 4.11310600 4.21396800 0.83419600

H 2.91079000 2.63184500 -0.02459400

C 5.26178000 4.52972000 1.56894400

H 6.99597300 3.73982800 2.59062200

H 3.46249600 5.01125200 0.47054700

H 5.50452500 5.57260900 1.78680500

**int-S7**

0 1

C 1.51342000 -1.33154400 -2.33017000

C 0.77204800 -1.23289200 -3.51338300

H 0.68433400 -0.25927800 -3.99790100

C 0.13801900 -2.36392400 -4.03394600

H -0.43658600 -2.27913300 -4.95896600

C 0.17534600 -3.57917100 -3.33880000

H -0.38543300 -4.44263100 -3.70399700

C 0.90633000 -3.66064000 -2.15456400

C 1.01576100 -4.81637700 -1.18541200

H 0.03131100 -5.25532800 -0.96398900

H 1.65245600 -5.62133900 -1.59255200

C 1.64695700 -4.15739600 0.05755900

H 2.23478100 -4.84951300 0.67781300

H 0.84586300 -3.73767400 0.68131500

C 2.50824400 -2.98602900 -0.50881000

C 1.63778400 -2.55729200 -1.67813600

C 3.88569900 -3.52612800 -1.01387300

H 4.30202100 -2.79828700 -1.72813200

H 3.79135300 -4.48949600 -1.53465600

C 4.76899800 -3.58300100 0.24600800

H 5.84246500 -3.47148200 0.02783500

H 4.64991800 -4.54527300 0.77470000

C 4.22413500 -2.44685400 1.08151200

C 4.80315200 -1.82256000 2.18652300

H 5.77457600 -2.15111700 2.56381000

C 4.12133700 -0.76734800 2.80552900

H 4.55367700 -0.27381200 3.67917000

C 2.90357300 -0.31558000 2.29154900

H 2.36550800 0.52069000 2.73809600

C 2.34746800 -0.93359800 1.16431000

C 2.97446700 -2.03651400 0.58285500

O 1.18542700 -0.43258800 0.63642700

O 2.05373600 -0.18487500 -1.77230000

P 1.09362800 0.63476800 -0.66172900

N 2.12503700 1.92041100 -0.40513400

C 1.56063000 3.24358200 -0.07170800

C 0.46004000 3.70810700 -1.03635900

C 1.08086100 3.30518800 1.38201500

H 2.39063400 3.95736500 -0.19101400

C -0.07053300 5.09474500 -0.66616000

H -0.39538900 2.99933800 -0.98606100

H 0.83323300 3.67810500 -2.07171100

C 0.54935800 4.69042800 1.75379700

H 0.28474900 2.55612100 1.51638300

H 1.90309900 3.00931400 2.05099200

C -0.54960700 5.14127200 0.78849400

H -0.88769200 5.37274300 -1.35068900

H 0.73168300 5.84171800 -0.81156600

H 0.17295000 4.67966100 2.78950600

H 1.37891900 5.42131700 1.73050300

H -0.89752500 6.15545700 1.04320900

H -1.42092200 4.47315400 0.90155400

C 3.59235400 1.78666000 -0.52999400

C 4.10015100 2.34960600 -1.86487800

C 4.37038500 2.38372300 0.64960300

H 3.80427100 0.70887200 -0.53477800

C 5.60471700 2.10752300 -2.02648300

H 3.89030700 3.43396400 -1.90749500

H 3.53807100 1.87615800 -2.68318000

C 5.87025200 2.11330000 0.48944700

H 4.20630400 3.47378300 0.70128100

H 4.00165600 1.95395500 1.59008800

C 6.39930200 2.66211600 -0.83970600

H 5.96161200 2.54874100 -2.97147500

H 5.78285500 1.01950800 -2.10390900

H 6.42348000 2.55245400 1.33579700

H 6.04278700 1.02293000 0.53568700

H 7.47094700 2.42919200 -0.95281200

H 6.31799700 3.76470900 -0.83218000

Pd -1.21295000 0.95753600 -1.01266800

C -2.32038800 -1.75700600 -1.44329100

C -3.60383500 -2.17035900 -1.35332100

C -4.42011300 -1.86207900 -0.19139900

C -3.82155100 -1.30339400 0.96227000

C -2.30854100 -1.14558300 1.01620700

C -1.65312200 -1.01972400 -0.38065500

H -6.26614100 -2.49677300 -1.10692500

H -1.74005000 -1.95669900 -2.34276200

H -4.07177800 -2.71095600 -2.18013000

C -5.80940900 -2.07504200 -0.20771200

C -4.62473400 -0.98447500 2.06029600

C -6.00555500 -1.20094400 2.03099600

C -6.60063800 -1.74712400 0.89100100

H -4.16721000 -0.55983200 2.95521800

H -6.61377200 -0.94314700 2.90121200

H -7.67972600 -1.91583200 0.85928500

C -1.63513900 -2.44613600 1.59301700

N -0.80716400 -2.05683600 2.63488000

O -1.74170100 -3.57858100 1.17825500

C 0.03866900 -2.96917300 3.36538700

H -0.12728600 -3.97450600 2.95819000

H 1.10041700 -2.69661000 3.25167000

H -0.21595600 -2.96791100 4.43847600

C -0.93349800 -0.69322200 2.91152100

C -1.83166700 -0.10316700 2.00930900

C -0.32539800 0.03669200 3.93132200

C -2.17995300 1.23116700 2.15762800

C -0.67071100 1.39068400 4.05864200

C -1.59790900 1.97957300 3.19436600

H -2.91178600 1.67975400 1.48788000

H -0.21609900 1.98588800 4.85447400

H -1.87102300 3.02912900 3.32219600

H 0.38404100 -0.43010400 4.61591200

H -0.61290200 -1.33625100 -0.29854900

C -3.14869100 1.51349300 -1.29837900

C -4.06681700 0.92681000 -2.17993600

C -3.50516400 2.75513500 -0.73026800

C -5.28365900 1.54411900 -2.48824400

H -3.84203000 -0.04107700 -2.63171700

C -4.72332300 3.37785500 -1.02902900

H -2.82770400 3.26189600 -0.03776100

C -5.62118400 2.77084100 -1.90968600

H -5.97649000 1.05871500 -3.18199600

H -4.97086800 4.33693400 -0.56456900

H -6.57732700 3.24756800 -2.14057600

**int-S8**

0 1

C -0.91292200 -1.44265700 1.58919500

C 0.28000300 -1.29708800 2.30609000

H 0.59837600 -0.30051600 2.60632300

C 1.05197100 -2.42129600 2.60764100

H 1.99117700 -2.29362400 3.14986500

C 0.65455900 -3.69143700 2.17813000

H 1.28418300 -4.56293600 2.37061800

C -0.53749000 -3.82230300 1.47002200

C -1.13491100 -5.06175400 0.84598600

H -0.37501100 -5.69946800 0.36893900

H -1.63945300 -5.68293500 1.60748100

C -2.14572500 -4.47541200 -0.15636100

H -2.98125700 -5.14954900 -0.39334100

H -1.63290200 -4.23118900 -1.09889900

C -2.62195600 -3.13751500 0.49943500

C -1.34437100 -2.70551700 1.19262900

C -3.78034800 -3.42180900 1.50429400

H -3.87037200 -2.55929200 2.18369300

H -3.59260000 -4.31426200 2.11827100

C -5.03725500 -3.52821500 0.61822600

H -5.96066500 -3.23381900 1.14116100

H -5.19125200 -4.56189400 0.26194000

C -4.71113400 -2.60419900 -0.53509500

C -5.56809700 -2.09201500 -1.50894000

H -6.62625400 -2.36416100 -1.51776800

C -5.05007100 -1.22155700 -2.47458300

H -5.69957400 -0.82159100 -3.25667500

C -3.71588400 -0.81288800 -2.41262400

H -3.31413100 -0.08521600 -3.11833700

C -2.87029700 -1.30579100 -1.41066100

C -3.34562100 -2.26705600 -0.51401400

O -1.59709700 -0.79018500 -1.30105900

O -1.64417500 -0.31905100 1.22365300

P -1.23215900 0.44700000 -0.20491100

N -2.47915600 1.55992500 -0.24439200

C -2.28149500 2.85162300 -0.94375400

C -1.37557800 3.83464000 -0.19242500

C -1.81082900 2.66189600 -2.39397200

H -3.28030300 3.30642800 -0.99838500

C -1.26490700 5.17086700 -0.93036000

H -0.37394600 3.39426300 -0.09028700

H -1.75171200 3.97779100 0.83145300

C -1.67834800 4.00045500 -3.12592000

H -0.83055100 2.15463800 -2.38970800

H -2.51333200 1.99672000 -2.91934800

C -0.76753400 4.96786800 -2.36437900

H -0.58953600 5.84432300 -0.37858600

H -2.25257300 5.66888200 -0.95625500

H -1.30125400 3.83219800 -4.14820100

H -2.67995000 4.45604200 -3.23467800

H -0.70335600 5.93214800 -2.89483100

H 0.25682200 4.55370600 -2.33350500

C -3.74813900 1.31897400 0.48221700

C -3.78293200 1.98286900 1.86632000

C -5.00718300 1.68942700 -0.31179500

H -3.79792400 0.23573500 0.64732400

C -5.03241500 1.54265700 2.63760200

H -3.78264100 3.08087700 1.74616900

H -2.87229600 1.71703100 2.42137100

C -6.25479000 1.21603800 0.44286100

H -5.07236800 2.78307000 -0.44139400

H -4.96547100 1.24072500 -1.31251900

C -6.31686100 1.82167900 1.84946500

H -5.06782000 2.03907800 3.62115300

H -4.95863100 0.45811400 2.83871100

H -7.16115900 1.47586300 -0.12852500

H -6.23443300 0.11393200 0.51105000

H -7.19376800 1.43672500 2.39601600

H -6.45758000 2.91480100 1.76352600

Pd 0.99300700 0.96537400 -0.50631200

C 2.09197700 0.87372700 -2.39987100

C 1.29492600 -0.27292400 -2.46744900

C 1.72473800 -1.52020700 -1.82902000

C 2.90165200 -1.54950000 -1.04423700

C 3.75447200 -0.29023400 -0.88617100

C 3.01477800 0.97133500 -1.32081300

H 0.06119600 -2.66614600 -2.57671200

H 1.86474400 1.74391100 -3.02086500

H 0.46661000 -0.32410900 -3.17466900

C 0.98245600 -2.70277800 -1.99239500

C 3.31102000 -2.75401500 -0.46751800

C 2.57821800 -3.92715300 -0.65682500

C 1.41012800 -3.89901400 -1.42321700

H 4.21538000 -2.77122400 0.14429300

H 2.91510100 -4.85962100 -0.19856700

H 0.82966800 -4.81163100 -1.57437700

C 5.00531400 -0.42451500 -1.82364400

N 6.13544600 -0.35065500 -1.02783700

O 4.99603600 -0.54938800 -3.02672700

C 7.47799200 -0.43159400 -1.54810300

H 7.40480800 -0.54624900 -2.63747900

H 8.04920400 0.48253200 -1.31476600

H 8.01535100 -1.29723500 -1.12574400

C 5.80338300 -0.20210500 0.32591000

C 4.40693100 -0.16551100 0.47762900

C 6.66303500 -0.11110900 1.41854800

C 3.84955100 -0.05296900 1.74092800

C 6.08536100 0.01570100 2.69174800

C 4.69807800 0.04260200 2.85574800

H 2.76841800 -0.02736300 1.85598700

H 6.73629200 0.09359000 3.56604800

H 4.26782200 0.14846700 3.85368600

H 7.74668500 -0.13861000 1.29371500

H 3.59593200 1.89053500 -1.22239100

C 1.31112200 2.10413300 1.19743200

C 0.38114200 2.17727500 2.25422500

C 2.46595200 2.90138300 1.33123900

C 0.59261700 2.97812200 3.38199300

H -0.54752100 1.60743000 2.21006000

C 2.68493300 3.71053100 2.45195500

H 3.23161000 2.89473800 0.55544100

C 1.75111400 3.75078500 3.48943200

H -0.15846000 2.99843700 4.17731000

H 3.59896100 4.30829500 2.51374500

H 1.92166300 4.37882500 4.36763500

**int-S9**

0 1

C -1.62925600 -1.64815200 1.37037800

C -0.42710800 -1.73028900 2.08489700

H 0.01898100 -0.80789300 2.45173200

C 0.18308400 -2.96745400 2.29339300

H 1.12907000 -3.01732800 2.83641900

C -0.37998400 -4.13227300 1.75937000

H 0.12550100 -5.09334200 1.87802400

C -1.56882600 -4.03958400 1.03932300

C -2.31800000 -5.12574400 0.29966600

H -1.64516500 -5.79930300 -0.25424600

H -2.89199600 -5.75789500 1.00041900

C -3.25350100 -4.31857000 -0.62210000

H -4.16822300 -4.85712200 -0.90899000

H -2.72085000 -4.04514300 -1.54630300

C -3.55493700 -3.00846400 0.17517700

C -2.22275800 -2.80590400 0.87212700

C -4.71208400 -3.26158100 1.19211200

H -4.67326400 -2.47466500 1.96197100

H -4.62102900 -4.23284500 1.69933600

C -5.99785600 -3.11241400 0.35640700

H -6.86335600 -2.78040200 0.95089900

H -6.28445200 -4.07002200 -0.11308600

C -5.59420500 -2.09914400 -0.69173200

C -6.41275600 -1.35343700 -1.53967600

H -7.49807300 -1.47851400 -1.51664900

C -5.82137800 -0.43745700 -2.41770500

H -6.44389900 0.14902900 -3.09757500

C -4.44117200 -0.22859400 -2.39432600

H -3.96817500 0.52353800 -3.02713200

C -3.62910300 -0.96000300 -1.51729700

C -4.19550800 -1.95054400 -0.70904900

O -2.29225400 -0.65159100 -1.43895900

O -2.18610700 -0.41438000 1.11169900

P -1.65390800 0.39980700 -0.27531600

N -2.68131400 1.72487400 -0.13841600

C -2.10508100 3.06697000 -0.37593100

C -0.95771800 3.42418800 0.58414300

C -1.69172700 3.26203800 -1.84213400

H -2.91778000 3.77996700 -0.17256900

C -0.40574900 4.82442000 0.31752300

H -0.13340800 2.69398800 0.45527100

H -1.29791400 3.31423800 1.62506400

C -1.10721700 4.65332600 -2.09841700

H -0.93359500 2.49576100 -2.09384000

H -2.56294900 3.06976900 -2.48893900

C 0.04640500 4.96202000 -1.13830400

H 0.42985900 5.02910500 1.00437800

H -1.18616600 5.57914700 0.53044900

H -0.77252900 4.73175500 -3.14593000

H -1.89815800 5.41515600 -1.96662300

H 0.44491900 5.97240900 -1.32748200

H 0.87391000 4.25206400 -1.32097900

C -4.01445300 1.63599000 0.48602300

C -4.01447800 2.04704700 1.96692300

C -5.09844000 2.40509800 -0.28161400

H -4.29659100 0.57536400 0.45385200

C -5.39030400 1.79806300 2.59461400

H -3.75775400 3.11854400 2.05230400

H -3.23567200 1.47876000 2.49510500

C -6.47710200 2.14419600 0.33476600

H -4.89211400 3.48876700 -0.24447200

H -5.08292100 2.10924000 -1.33898000

C -6.50364000 2.51325400 1.82192500

H -5.39057900 2.11257600 3.65140400

H -5.58864600 0.71058200 2.59301100

H -7.24941200 2.70716800 -0.21520900

H -6.72385500 1.07476000 0.21404800

H -7.48807300 2.27748000 2.25935900

H -6.36923300 3.60591600 1.92481400

Pd 0.50878700 0.82071000 -0.73128800

C 2.49895900 0.88491100 -1.72486200

C 1.90129500 -0.33544500 -1.99895600

C 2.27554200 -1.54890200 -1.25695100

C 3.43722900 -1.55262000 -0.45064000

C 4.37030300 -0.34650000 -0.50647100

C 3.60667100 1.01088500 -0.68342000

H 0.57743200 -2.68791300 -1.93307700

H 2.40432500 1.70590000 -2.44023400

H 1.31236900 -0.46696300 -2.90964000

C 1.48797300 -2.70874100 -1.32983600

C 3.77415200 -2.70819700 0.26143000

C 2.98381300 -3.85788400 0.17675800

C 1.84141500 -3.85793500 -0.62789000

H 4.67495700 -2.71359400 0.87767300

H 3.26383200 -4.75230500 0.73833400

H 1.21671200 -4.75019300 -0.69743600

C 5.26333500 -0.51341100 -1.78181600

N 6.58684900 -0.44644800 -1.37810600

O 4.89531800 -0.66100000 -2.92453000

C 7.69757600 -0.56344400 -2.29056800

H 7.28423800 -0.68814600 -3.29982200

H 8.33021600 0.33942900 -2.26368000

H 8.32265900 -1.43755700 -2.04241700

C 6.69566200 -0.31520000 0.00859000

C 5.41526700 -0.27172000 0.59214200

C 7.86112200 -0.24691500 0.76861400

C 5.30278500 -0.17338200 1.97377800

C 7.72745300 -0.13779000 2.16031300

C 6.46610600 -0.10544400 2.75849700

H 4.32379600 -0.15335900 2.45032600

H 8.62507400 -0.08255800 2.78094700

H 6.37974700 -0.02676300 3.84420700

H 8.84557100 -0.28158500 0.29959800

H 4.34889800 1.72921700 -1.06337800

C 3.04638900 1.61386000 0.59554300

C 2.20162000 0.87940700 1.45306100

C 3.29070200 2.96009600 0.89234800

C 1.63546900 1.48714800 2.57806200

H 2.03122500 -0.18237600 1.26752200

C 2.71062100 3.57106000 2.00859800

H 3.94234600 3.54062800 0.23416300

C 1.87833400 2.83540000 2.85461100

H 0.99349600 0.90518000 3.24284600

H 2.91415400 4.62400300 2.21779700

H 1.42293500 3.30790500 3.72786800

**int1**

0 1

C -2.07953900 1.45099200 -1.78988000

C -1.39198900 1.20691700 -2.98593800

H -1.45318900 0.21400800 -3.43370800

C -0.61560300 2.21540900 -3.56305400

H -0.07505900 2.01162900 -4.48979100

C -0.48097600 3.45734200 -2.93083200

H 0.17338500 4.22448300 -3.35164300

C -1.16581200 3.68647500 -1.73780100

C -1.11585800 4.89519200 -0.83014700

H -0.09458500 5.28936400 -0.71272000

H -1.73106900 5.71818800 -1.23510200

C -1.70420100 4.35059200 0.48694100

H -2.17266800 5.12167000 1.11558900

H -0.90772900 3.87371600 1.07942300

C -2.71545000 3.24576300 0.04086300

C -2.00009100 2.70185400 -1.18124300

C -4.08713400 3.89046500 -0.33493700

H -4.63542300 3.18473300 -0.97870700

H -3.96981900 4.83093700 -0.89238600

C -4.82239600 4.05020800 1.00896700

H -5.91856700 4.02087000 0.90826300

H -4.57348800 5.01281700 1.48978400

C -4.28259900 2.89443300 1.82131000

C -4.78454700 2.35437700 3.00535000

H -5.67787600 2.77482900 3.47356100

C -4.12441000 1.26480500 3.58559300

H -4.49355000 0.83538700 4.51995200

C -3.01720000 0.69284200 2.95516700

H -2.51659200 -0.18369900 3.36840000

C -2.54209000 1.21976000 1.74737700

C -3.13540100 2.36349100 1.20569600

O -1.51557500 0.57855900 1.09508000

O -2.74110200 0.41545600 -1.15743500

P -1.76207300 -0.53638700 -0.15887700

N -2.93090400 -1.62817800 0.33671000

C -2.55571000 -3.05530200 0.44286100

C -2.14249300 -3.68606600 -0.89746800

C -1.49130400 -3.29466800 1.52467500

H -3.46800900 -3.57478600 0.77169900

C -1.81015400 -5.17189300 -0.73857400

H -1.25467100 -3.15058400 -1.27887700

H -2.94643800 -3.53566400 -1.63482800

C -1.15251600 -4.78068700 1.66805300

H -0.57762400 -2.73827300 1.25212900

H -1.84912400 -2.87561900 2.47898900

C -0.73009100 -5.38737100 0.32615400

H -1.48895200 -5.59116000 -1.70633400

H -2.72214100 -5.72616200 -0.44707100

H -0.35733000 -4.91335600 2.42006800

H -2.03645000 -5.32544700 2.04905900

H -0.51056300 -6.46174800 0.44144000

H 0.20473400 -4.90344800 -0.00764800

C -4.35669300 -1.25746400 0.43631500

C -5.16779600 -1.66092700 -0.80411100

C -5.03659500 -1.77267100 1.71167800

H -4.38261600 -0.16068000 0.48729300

C -6.59922900 -1.12060600 -0.70994900

H -5.19111300 -2.76266300 -0.88570000

H -4.66300600 -1.27470000 -1.70125400

C -6.45814100 -1.21054000 1.81705400

H -5.09090900 -2.87478700 1.69575100

H -4.44060400 -1.49087800 2.58987600

C -7.29197800 -1.56841200 0.58174700

H -7.18422400 -1.43400200 -1.59029900

H -6.56240600 -0.01627500 -0.73677500

H -6.94572200 -1.58521500 2.73219700

H -6.39926600 -0.11258400 1.91981900

H -8.29703400 -1.12047800 0.65322200

H -7.43959500 -2.66379800 0.55170800

Pd 0.28281100 -1.02919900 -0.91329800

C 6.80248500 -0.58416400 -0.77866800

C 7.50964300 -0.69489500 0.39805000

C 7.09148100 0.00077100 1.56541100

C 5.92449100 0.83416500 1.50760700

C 5.19515900 0.90440700 0.27887400

C 5.63408300 0.21040600 -0.83326400

H 8.68654500 -0.74982000 2.82932500

H 7.13210700 -1.11659300 -1.67371200

H 8.40477000 -1.31986500 0.45227000

C 7.79895300 -0.11264200 2.79419000

C 5.52162400 1.52596900 2.68496500

H 5.06771900 0.27198500 -1.76461300

C 6.22861200 1.38911700 3.86088900

C 7.37762700 0.56217400 3.91907500

H 4.65623100 2.18728700 2.63760700

H 5.90575200 1.92924300 4.75411200

H 7.92958000 0.46518700 4.85701800

C 3.96198400 1.76497700 0.18569700

N 2.79309400 1.11604800 -0.17510400

O 4.00655400 2.96655200 0.39251100

C 1.58177500 1.91608800 -0.37936100

H 1.16140800 1.73433600 -1.37691900

H 0.82144900 1.66101900 0.37326000

H 1.86314400 2.97103600 -0.28630400

C 2.68688600 -0.30035000 -0.12871700

C 2.40591500 -1.07683900 -1.30056100

C 2.95021600 -0.98447300 1.06947900

C 2.38395000 -2.49442100 -1.22320100

C 2.97150700 -2.37850900 1.12838400

C 2.67122000 -3.13165100 -0.00908700

H 2.22829300 -3.07464400 -2.13338400

H 3.20533700 -2.87524900 2.07171300

H 2.67060100 -4.22264400 0.03205700

H 3.16238100 -0.39097000 1.95969500

Br 2.51725000 -0.25040600 -3.04671200

**int10**

0 1

C 1.77146500 1.36275500 2.09524500

C 1.15469800 0.87202700 3.25079200

H 1.22526100 -0.19486200 3.46413400

C 0.41497900 1.74058400 4.05658500

H -0.07617400 1.35369800 4.95229500

C 0.25607700 3.08402900 3.69515000

H -0.37061500 3.74783400 4.29518100

C 0.88512700 3.55518000 2.54434800

C 0.80518700 4.92879100 1.91883600

H -0.21663700 5.33792500 1.93598800

H 1.44519300 5.64681800 2.46122700

C 1.32645100 4.67816400 0.49134400

H 1.77322500 5.56431500 0.01877000

H 0.49997000 4.33875800 -0.15201300

C 2.34788700 3.50331000 0.64198300

C 1.68399200 2.70906800 1.75357200

C 3.73980700 4.06005300 1.07528500

H 4.33143200 3.22958100 1.49272000

H 3.65884600 4.83475400 1.85088700

C 4.38166000 4.55563500 -0.23510500

H 5.48177400 4.51079400 -0.22007200

H 4.11071100 5.60516900 -0.44442700

C 3.78361200 3.62880000 -1.27026400

C 4.18678500 3.42348200 -2.58965000

H 5.02580500 3.98546200 -3.00660300

C 3.50201400 2.48617500 -3.37192000

H 3.79213400 2.32384300 -4.41239400

C 2.48151100 1.71328500 -2.81325600

H 1.97666000 0.93370600 -3.38456800

C 2.10839300 1.90933300 -1.47953300

C 2.70107300 2.91889000 -0.71831500

O 1.18004400 1.04888200 -0.91996800

O 2.43495800 0.47728200 1.25722800

P 1.63980000 -0.25858100 -0.00036800

N 2.96753600 -1.01100400 -0.66678700

C 2.89385500 -2.30738600 -1.38235300

C 2.17943300 -3.41863000 -0.60925200

C 2.34969900 -2.15167500 -2.80740700

H 3.94004900 -2.63479700 -1.46873800

C 2.22375900 -4.74268800 -1.37443300

H 1.12714600 -3.15699200 -0.42610100

H 2.63741500 -3.53271800 0.38448300

C 2.35641200 -3.48465400 -3.56433000

H 1.32545800 -1.75125000 -2.75736900

H 2.95166300 -1.40288000 -3.34374600

C 1.64205400 -4.59316000 -2.78344700

H 1.67554200 -5.50481700 -0.80004300

H 3.27065700 -5.09133900 -1.44950400

H 1.89915600 -3.35217900 -4.55861200

H 3.40354900 -3.79128200 -3.74351700

H 1.71016900 -5.54781700 -3.32995300

H 0.56568700 -4.35670900 -2.70936400

C 4.32353800 -0.41913300 -0.49111200

C 5.10204000 -1.11465100 0.63286000

C 5.14878400 -0.36775000 -1.78287600

H 4.17337900 0.62188700 -0.18407500

C 6.44215000 -0.41189800 0.87889200

H 5.27767000 -2.17052500 0.35911100

H 4.49030100 -1.11061000 1.54681700

C 6.47281200 0.36223900 -1.52883500

H 5.36899100 -1.38668500 -2.14247100

H 4.57643600 0.14145500 -2.56794100

C 7.27185900 -0.30389500 -0.40425800

H 7.00834800 -0.93960100 1.66337900

H 6.24326100 0.60332700 1.26861300

H 7.06509900 0.39560200 -2.45777300

H 6.25629000 1.41161400 -1.25978800

H 8.20436600 0.25186300 -0.21220500

H 7.57239600 -1.31733200 -0.72752300

Pd -0.15389500 -1.37559600 0.76488900

C -5.81316800 -1.38542700 -1.48935100

C -6.91592600 -0.67183600 -1.90075300

C -7.03731500 0.71041700 -1.59499900

C -6.00677700 1.36313600 -0.83372600

C -4.86119400 0.59564900 -0.43090600

C -4.78371600 -0.74444400 -0.76693500

H -8.93897800 0.94811500 -2.60832800

H -5.71744000 -2.44751900 -1.72586400

H -7.71303300 -1.15527200 -2.47159900

C -8.16460500 1.46065200 -2.03147900

C -6.16480400 2.75004100 -0.54496600

H -3.91161600 -1.32377500 -0.47702600

C -7.26840600 3.44705900 -0.98888000

C -8.28107300 2.80101400 -1.73912100

H -5.40840000 3.24965600 0.05714300

H -7.36621500 4.50959900 -0.75290800

H -9.15134500 3.36647900 -2.08083200

C -3.79197700 1.25392300 0.40675800

N -2.46717900 1.09070400 0.05753800

O -4.10148200 1.93386900 1.38025500

C -1.47551700 1.76798000 0.88075900

H -1.94056300 2.64925800 1.33599200

H -1.10469200 1.12673400 1.69398000

H -0.63013200 2.05680200 0.25051600

C -2.03527600 0.28549200 -1.03722300

C -1.20946800 -0.84490900 -0.86273700

C -2.53088900 0.59489200 -2.31398700

C -1.05325500 -1.71097500 -1.95474100

C -2.32613800 -0.25577000 -3.39768400

C -1.61746600 -1.44215900 -3.20597800

H -0.47625600 -2.62666900 -1.82703900

H -2.73955600 -0.00289900 -4.37639100

H -1.47729300 -2.14911100 -4.02763500

H -3.12865800 1.50051900 -2.42947900

B -0.04551500 -3.41117500 2.66011500

O 0.90617100 -2.20048300 2.45685600

H 1.80068200 -2.53858300 2.31097300

O 0.06746900 -3.95203000 3.96521400

H -0.37982900 -3.39979000 4.61408600

C -1.51768400 -2.77653600 2.27395000

C -2.47842700 -3.57118600 1.59827300

C -1.95724800 -1.52015800 2.77022800

C -3.80940800 -3.17834100 1.51116300

H -2.15335100 -4.52788400 1.18234100

C -3.29296300 -1.10442400 2.65474000

H -1.25375500 -0.88421900 3.31542800

C -4.22136100 -1.94958400 2.05364300

H -4.54117000 -3.82106200 1.01450600

H -3.60190100 -0.12517100 3.02498600

H -5.26498900 -1.64014300 1.97278500

F 0.34106300 -4.38576300 1.71445400

**int12**

0 1

C -2.01664700 -1.44366300 1.79462000

C -1.26818900 -1.05957600 2.92252400

H -1.47152900 -0.09313400 3.39110200

C -0.30913100 -1.93622300 3.44732600

H 0.28217600 -1.62773600 4.31042900

C -0.05477200 -3.16148000 2.82440500

H 0.74484300 -3.80804700 3.19168200

C -0.79817600 -3.52049900 1.69876200

C -0.63288000 -4.72037700 0.79442800

H 0.42579000 -4.94836100 0.59806200

H -1.07576000 -5.62038400 1.25626400

C -1.40164600 -4.29935700 -0.47602500

H -1.80526200 -5.14635300 -1.04897900

H -0.73288400 -3.73448700 -1.14278400

C -2.52371600 -3.33769900 0.03290600

C -1.81235000 -2.68502400 1.20161300

C -3.76713900 -4.15531300 0.50555600

H -4.35492800 -3.52666600 1.19285900

H -3.48494200 -5.07002000 1.04662700

C -4.57377000 -4.41627600 -0.78001900

H -5.65321800 -4.53110000 -0.59632000

H -4.23695700 -5.33969000 -1.28339100

C -4.25251800 -3.20468300 -1.62586800

C -4.90982700 -2.74290100 -2.76637700

H -5.77669700 -3.27756400 -3.16189400

C -4.43934400 -1.58634400 -3.39940800

H -4.92992500 -1.21782600 -4.30320500

C -3.36110000 -0.87868600 -2.86399900

H -2.99707600 0.04015300 -3.32488400

C -2.73409700 -1.32947900 -1.69694100

C -3.13729200 -2.52775800 -1.10068600

O -1.72927700 -0.56581900 -1.14284200

O -2.83504400 -0.51472300 1.17858400

P -1.92546200 0.48220600 0.15438500

N -3.11931100 1.58043000 -0.22659900

C -2.80139600 3.02069900 -0.28684300

C -2.27633900 3.59511900 1.03643100

C -1.86237500 3.36270200 -1.44845900

H -3.76186600 3.51902600 -0.48605700

C -2.09469100 5.11326500 0.92833000

H -1.31444900 3.11444400 1.28422200

H -2.97598800 3.33999900 1.84807900

C -1.65452700 4.87686200 -1.56289600

H -0.89229100 2.86334200 -1.28611100

H -2.28006600 2.95467800 -2.38269800

C -1.18214700 5.49666200 -0.24234600

H -1.69606500 5.51574900 1.87383800

H -3.08616500 5.58180400 0.78512300

H -0.93282200 5.09622000 -2.36610800

H -2.60927400 5.34789900 -1.86206700

H -1.12796600 6.59361700 -0.33706900

H -0.15759300 5.15251500 -0.03034300

C -4.53598000 1.16554500 -0.32285500

C -5.34079900 1.53301300 0.93185000

C -5.23517100 1.68026600 -1.58719500

H -4.53239600 0.06889000 -0.38764000

C -6.76548300 0.97420900 0.84161300

H -5.37890000 2.63231200 1.03566300

H -4.82127600 1.13758400 1.81675700

C -6.65000400 1.10001300 -1.68419800

H -5.30063400 2.78139200 -1.56307100

H -4.64358600 1.41325100 -2.47310000

C -7.47562900 1.43275300 -0.43660700

H -7.34545800 1.26647400 1.73225200

H -6.71395600 -0.12970000 0.85092800

H -7.15107300 1.47840500 -2.59019500

H -6.57823500 0.00383600 -1.79951900

H -8.47607500 0.97462000 -0.50438000

H -7.63488800 2.52583800 -0.39041200

Pd 0.23038300 0.52072700 1.25967900

C 6.12594600 1.37417500 0.52359600

C 7.20799200 1.16192900 -0.30045800

C 7.16679900 0.15442200 -1.30221300

C 5.98847700 -0.65562400 -1.44095800

C 4.87180100 -0.39667200 -0.57919300

C 4.95321300 0.60061600 0.37478800

H 9.16020200 0.55479200 -2.05555700

H 6.15724900 2.14459500 1.29707600

H 8.11467100 1.76429200 -0.19888100

C 8.26983100 -0.06890500 -2.17250200

C 5.97690500 -1.66122400 -2.45030400

H 4.10374000 0.80330200 1.02149900

C 7.06242200 -1.84693900 -3.27994500

C 8.22208000 -1.04502800 -3.14314600

H 5.10204800 -2.30360800 -2.54164900

H 7.03132700 -2.62562100 -4.04611500

H 9.07612500 -1.20534300 -3.80573900

C 3.64554200 -1.26688000 -0.68996700

N 2.42474900 -0.66438400 -0.89832000

O 3.73448300 -2.48732800 -0.60839200

C 1.27042900 -1.54739100 -0.99768500

H 1.57571400 -2.47354500 -1.50250900

H 0.87313700 -1.81972300 -0.00514700

H 0.47520400 -1.04466000 -1.55499300

C 2.23954200 0.74963100 -0.98032000

C 1.43614700 1.44385900 -0.05269200

C 2.91575900 1.45516300 -1.98793700

C 1.44016900 2.84415700 -0.09487700

C 2.87934200 2.84746900 -2.03721200

C 2.15323600 3.54708000 -1.07143600

H 0.88099100 3.40391500 0.65642900

H 3.42857700 3.38061900 -2.81635200

H 2.13424400 4.64022500 -1.07639100

H 3.50183400 0.89070000 -2.71531500

C 1.94731500 0.31818000 2.29013500

C 2.59824600 -0.92524100 2.34998800

C 2.47096700 1.38198700 3.04214900

C 3.72249100 -1.10777900 3.16044500

H 2.24470300 -1.76777600 1.75489200

C 3.59204700 1.19846500 3.86237500

H 2.01471400 2.37337400 2.98017000

C 4.22260600 -0.04707500 3.92214100

H 4.22265100 -2.07963100 3.17494400

H 3.98264000 2.03886300 4.44314700

H 5.10958300 -0.18652400 4.54496700

**int3**

0 1

C -1.37366500 1.81565300 -1.48849000

C -0.44832900 2.14012900 -2.48481500

H 0.01453800 1.33069000 -3.04978100

C -0.13595200 3.48073800 -2.72188900

H 0.60780100 3.72854600 -3.48065500

C -0.73060300 4.49552900 -1.96334800

H -0.45489900 5.54018200 -2.12652800

C -1.64786900 4.15215300 -0.97209900

C -2.35114700 5.04893100 0.02057300

H -1.69924200 5.84705200 0.40847700

H -3.22035300 5.54646000 -0.44541400

C -2.79527700 4.05386700 1.10763500

H -3.67080700 4.38460500 1.68408000

H -1.97217600 3.89020300 1.82076200

C -3.05621600 2.71292700 0.34019700

C -1.99457100 2.81012700 -0.73801100

C -4.51224900 2.70082000 -0.21406000

H -4.58642900 1.91893000 -0.98661000

H -4.79343800 3.65727200 -0.67748500

C -5.37823100 2.32463600 1.00730400

H -6.29613300 1.78189900 0.73177300

H -5.69765000 3.22082500 1.56700900

C -4.44173400 1.47060800 1.83597100

C -4.73523500 0.67087100 2.93963200

H -5.75361000 0.61609300 3.33177900

C -3.70256600 -0.06261200 3.53767200

H -3.90469000 -0.67668600 4.41820700

C -2.42451500 -0.06817700 2.97425800

H -1.62436000 -0.68455400 3.38175400

C -2.15175000 0.70591800 1.84018700

C -3.13213500 1.55832700 1.32732400

O -0.92415400 0.56071900 1.21897700

O -1.67029900 0.48513000 -1.22686600

P -0.77876800 -0.39243400 -0.15600200

N -1.76192400 -1.72615200 -0.03624400

C -1.26188900 -3.05012800 0.40788300

C -0.04360300 -3.57932700 -0.35653600

C -1.04476100 -3.10884300 1.92478400

H -2.08201800 -3.74448200 0.17450800

C 0.31340100 -4.99537900 0.10185100

H 0.82513700 -2.92140200 -0.18634200

H -0.23602200 -3.53892700 -1.43720100

C -0.64511200 -4.51424400 2.38594300

H -0.26070200 -2.38483700 2.20188400

H -1.96554000 -2.78836800 2.43172600

C 0.56284800 -5.05045800 1.61190700

H 1.19747000 -5.35088400 -0.45145600

H -0.51209300 -5.68513200 -0.15501600

H -0.44297700 -4.51048300 3.46974700

H -1.50036300 -5.19798000 2.23237900

H 0.79525800 -6.08009900 1.92993000

H 1.45258400 -4.44134300 1.85456100

C -3.17474800 -1.66690500 -0.49698400

C -3.34388100 -2.33000800 -1.86862800

C -4.17706100 -2.21113800 0.52656000

H -3.41387000 -0.60572700 -0.62064800

C -4.77878800 -2.15594300 -2.37784100

H -3.10882300 -3.40675000 -1.78969800

H -2.61641200 -1.89470600 -2.56984400

C -5.60711900 -2.01418400 0.00894400

H -4.00666000 -3.28717200 0.70249800

H -4.04229600 -1.69683600 1.48673800

C -5.80565700 -2.66832700 -1.36228100

H -4.90256700 -2.66750000 -3.34610600

H -4.96198000 -1.08251400 -2.56809000

H -6.32904200 -2.41497000 0.73934100

H -5.81208000 -0.93067100 -0.06775400

H -6.83072000 -2.49098900 -1.72776100

H -5.69584800 -3.76342500 -1.25704500

Pd 1.35933000 -0.51994300 -0.77684200

C 4.13721500 -0.96487500 -2.13810000

C 5.11539500 -1.37390400 -1.26331300

C 5.47648900 -0.56836800 -0.14480300

C 4.77004400 0.65439900 0.10657300

C 3.70012400 1.02128000 -0.77124400

C 3.44060300 0.25605000 -1.90857100

H 7.05487100 -1.88589800 0.54124900

H 3.86331100 -1.56337500 -3.00783400

H 5.64552300 -2.31558000 -1.42465600

C 6.52743100 -0.94803400 0.73206200

C 5.17230300 1.46134400 1.20398800

H 2.74332000 0.62886500 -2.65971100

C 6.20682700 1.07229300 2.02771800

C 6.88446300 -0.14856300 1.79713300

H 4.65713400 2.40409400 1.38631300

H 6.50400400 1.70779400 2.86481400

H 7.69682100 -0.45148800 2.46190800

C 2.84611000 2.25528800 -0.55909400

N 2.11555100 2.30284800 0.61048900

O 2.80266600 3.13232300 -1.40690000

C 1.28459800 3.47004600 0.86892500

H 1.21522500 4.04923100 -0.05753800

H 0.28177900 3.13792000 1.16857200

H 1.70930700 4.10905900 1.66136700

C 2.10914300 1.23164800 1.55114800

C 1.90589500 -0.08976100 1.12070100

C 2.35135200 1.50104900 2.90421900

C 2.06998400 -1.12760100 2.04241800

C 2.45275000 0.45849900 3.82665500

C 2.34606200 -0.86271800 3.38949600

H 1.96245000 -2.16407500 1.71799400

H 2.64517700 0.67995800 4.87888600

H 2.45850500 -1.69029900 4.09448900

H 2.49348600 2.53365100 3.23001400

Br 0.65460200 -1.30961000 -3.11267300

**int5**

0 1

C 1.34968800 0.02990200 -2.60687500

C 0.48611600 0.59659900 -3.55299700

H 0.20620600 1.64604000 -3.45571200

C -0.05692300 -0.20146700 -4.56276600

H -0.74141400 0.24743000 -5.28581500

C 0.22284000 -1.57313600 -4.61294800

H -0.24257400 -2.20623800 -5.37254100

C 1.09218600 -2.12169200 -3.66994800

C 1.50808400 -3.56410000 -3.48878300

H 0.66763700 -4.26533700 -3.60746500

H 2.26858700 -3.85003800 -4.23683800

C 2.09821700 -3.56497900 -2.06591700

H 2.84223900 -4.35557300 -1.89288300

H 1.29099700 -3.70166300 -1.32850900

C 2.69485800 -2.13065300 -1.88387800

C 1.69322900 -1.31842300 -2.68397000

C 4.12265000 -2.06620600 -2.51092700

H 4.37892600 -1.00783400 -2.67478500

H 4.17852100 -2.57915800 -3.48166700

C 5.04381900 -2.67066100 -1.43402900

H 6.06827000 -2.26833800 -1.46800900

H 5.13039800 -3.76567800 -1.54687600

C 4.33029800 -2.31929000 -0.14712000

C 4.80826700 -2.40134200 1.15991500

H 5.82153000 -2.75830300 1.35947600

C 3.96395500 -2.02910600 2.21393400

H 4.30657100 -2.11455100 3.24769300

C 2.69730500 -1.50612700 1.95223800

H 2.03964800 -1.16826700 2.75183100

C 2.25211800 -1.37426700 0.63087600

C 3.02615100 -1.86554000 -0.42292400

O 1.04194400 -0.76193500 0.40555100

O 1.75532800 0.78595900 -1.51860000

P 0.69982500 0.75588000 -0.20444600

N 1.43669600 1.86534500 0.79737500

C 0.81982400 3.19622400 1.03235600

C 0.70876900 4.04639400 -0.23942600

C -0.51937800 3.11119100 1.77216000

H 1.51755100 3.70808900 1.71372000

C 0.14881500 5.43849500 0.06955300

H 0.03617800 3.54292200 -0.95000600

H 1.69324300 4.12287700 -0.72209700

C -1.07158900 4.50812100 2.07552900

H -1.24727100 2.56625300 1.14927900

H -0.37809900 2.53769600 2.70171000

C -1.19287100 5.35192700 0.80320800

H 0.03616900 6.00733300 -0.86743900

H 0.87319000 5.99815700 0.69215200

H -2.04974400 4.41963700 2.57596300

H -0.39815700 5.01596200 2.79188300

H -1.56133000 6.36146700 1.05044100

H -1.93467800 4.88793900 0.13245000

C 2.88333700 1.74091700 1.09105200

C 3.77120200 2.71506800 0.30312500

C 3.16206900 1.82149500 2.59782700

H 3.18494100 0.73859000 0.76588300

C 5.25060000 2.45037800 0.60428700

H 3.52747100 3.75550400 0.57650500

H 3.56274900 2.59939500 -0.77036200

C 4.64593600 1.58652200 2.89928300

H 2.86763200 2.81540300 2.97699000

H 2.53280200 1.08485800 3.11907200

C 5.54088400 2.54306700 2.10538800

H 5.88160500 3.15893300 0.04327100

H 5.51756200 1.43972400 0.24475100

H 4.83116300 1.69063000 3.98119600

H 4.90333200 0.54688700 2.63379700

H 6.60343100 2.32842800 2.30665200

H 5.36023700 3.57972100 2.44460400

Pd -1.49550300 0.51877500 -0.92942900

C -2.31565300 -1.33615400 -1.66334000

C -3.41495700 -0.46271000 -1.56444100

C -4.18494600 -0.36165700 -0.31995500

C -3.73503300 -1.01740200 0.84777200

C -2.42378900 -1.79226800 0.83695500

C -1.63493700 -1.60451200 -0.45285400

H -5.69587700 0.91607400 -1.17537100

H -1.86919300 -1.58192500 -2.62932300

H -3.85894000 -0.04667600 -2.46923100

C -5.37238300 0.38796400 -0.27598900

C -4.49580400 -0.93439900 2.01596200

C -5.68442300 -0.20132900 2.04550200

C -6.11921300 0.46786200 0.89702400

H -4.14363500 -1.43502000 2.92068300

H -6.26444900 -0.14326900 2.96927300

H -7.03993100 1.05517000 0.91825700

C -2.69243300 -3.33745700 0.91098900

N -1.97573700 -3.83107400 1.98828300

O -3.36267900 -3.99020100 0.14663200

C -1.95389900 -5.22790800 2.34999200

H -2.55967500 -5.77113700 1.61314600

H -0.92402500 -5.62136600 2.33895200

H -2.37896800 -5.38425400 3.35554500

C -1.33907100 -2.80481600 2.69960000

C -1.58716000 -1.56877700 2.08222800

C -0.58651000 -2.89994100 3.86856200

C -1.11452700 -0.39933600 2.65104900

C -0.09871200 -1.70833900 4.42769700

C -0.36224000 -0.47040600 3.83387800

H -1.32902700 0.56019600 2.18405300

H 0.49074500 -1.75465100 5.34656400

H 0.01872700 0.44658200 4.28829000

H -0.39063800 -3.86309500 4.34192500

Br -2.24391900 2.72432500 -1.90776300

H -0.68972400 -2.14558200 -0.49034600

**int6**

0 1

C 0.95789300 1.01627600 2.06442900

C 0.21390700 0.39860100 3.07423500

H 0.29831400 -0.67909700 3.20845200

C -0.65103500 1.16131900 3.86269300

H -1.23204500 0.66804100 4.64526200

C -0.81995600 2.52979600 3.61429400

H -1.54775100 3.11044000 4.18616500

C -0.05575600 3.13534600 2.61642000

C -0.09245500 4.56764100 2.12990500

H -1.12285200 4.93407100 1.99701700

H 0.39424800 5.24371600 2.85458000

C 0.68951600 4.49848500 0.80288300

H 1.18371900 5.44239800 0.53076500

H 0.00949900 4.22801300 -0.01814500

C 1.70642100 3.33325500 1.00630900

C 0.88140700 2.39611000 1.87139000

C 2.96776200 3.83910400 1.77957000

H 3.46296100 2.96927800 2.23948300

H 2.71168800 4.54223100 2.58498200

C 3.87876300 4.44132200 0.69295700

H 4.94788700 4.39545700 0.95189700

H 3.64014100 5.50468600 0.51391800

C 3.53789800 3.60771900 -0.52147400

C 4.24725000 3.46820800 -1.71471300

H 5.17819000 4.01706300 -1.87550900

C 3.75188100 2.60752300 -2.70147200

H 4.29077200 2.48889700 -3.64430500

C 2.59151700 1.86304100 -2.47420700

H 2.22332000 1.15062600 -3.21268500

C 1.90095900 1.99545000 -1.26411500

C 2.34303000 2.90215700 -0.30139500

O 0.81619100 1.18983700 -1.00405600

O 1.70563900 0.22990700 1.20396300

P 0.98700200 -0.28160400 -0.21515300

N 2.31138800 -1.05431100 -0.87661400

C 2.14711600 -2.08375900 -1.93317500

C 2.12506300 -3.51229500 -1.37561400

C 0.94465600 -1.81760500 -2.85346800

H 3.03137200 -1.98459800 -2.57976100

C 2.00236300 -4.56631800 -2.47831000

H 1.26739100 -3.60414500 -0.69939500

H 3.03710600 -3.69025400 -0.78749500

C 0.85216200 -2.87509400 -3.95841300

H 0.01260100 -1.83415100 -2.26974300

H 1.02871400 -0.80914600 -3.28953100

C 0.79901100 -4.29385600 -3.38373800

H 1.90669100 -5.55955900 -2.01371100

H 2.92316100 -4.57991100 -3.09210500

H -0.03020300 -2.67437500 -4.58828900

H 1.73398200 -2.78518900 -4.61972100

H 0.75366500 -5.03259600 -4.20074500

H -0.12122200 -4.42693000 -2.79027600

C 3.67823700 -0.73597100 -0.37813100

C 4.11364700 -1.58550400 0.82624600

C 4.75808600 -0.76826800 -1.46398400

H 3.63679500 0.30374200 -0.02844700

C 5.42368800 -1.03963800 1.40421100

H 4.25468300 -2.63209300 0.51004800

H 3.31934200 -1.57799700 1.58384900

C 6.06780300 -0.20286300 -0.90121900

H 4.93350900 -1.80374300 -1.80161900

H 4.43472500 -0.18419100 -2.33746700

C 6.52594800 -0.97606800 0.34093700

H 5.74877600 -1.65534700 2.25872900

H 5.24119800 -0.02473400 1.80299800

H 6.85000100 -0.22611600 -1.67771400

H 5.91423500 0.85998300 -0.64218300

H 7.43985800 -0.52236600 0.75911700

H 6.79669400 -2.00540200 0.04254800

Pd -1.13525600 -1.13626800 0.14019400

C -2.67159800 -0.46277400 1.48885600

C -3.27878700 -1.47907800 0.74007900

C -3.89576100 -1.20686300 -0.56039300

C -3.82844200 0.08985300 -1.11903700

C -3.07696100 1.18657400 -0.37880000

C -2.18364500 0.64467300 0.73982000

H -4.53631800 -3.24349300 -0.83321700

H -2.36586400 -0.63681800 2.52030300

H -3.45112100 -2.45989700 1.18217000

C -4.53692400 -2.23998200 -1.26479300

C -4.44893400 0.33495300 -2.34526000

C -5.10745300 -0.69146100 -3.02994000

C -5.14218600 -1.98274900 -2.49422600

H -4.40074100 1.33491100 -2.78120100

H -5.58348700 -0.48242700 -3.99069800

H -5.64138800 -2.78928800 -3.03603500

C -4.07077200 2.12397200 0.40326400

N -3.75579700 3.42756600 0.06432800

O -4.91475200 1.78050900 1.19597300

C -4.42620300 4.57498100 0.62640400

H -5.14966000 4.20891000 1.36633500

H -3.70524300 5.24684500 1.12134700

H -4.95954400 5.14501600 -0.15247500

C -2.78327500 3.47877200 -0.94396300

C -2.37086000 2.17803100 -1.27947100

C -2.27658200 4.60041000 -1.59562600

C -1.47882000 1.97984100 -2.31905100

C -1.34546500 4.38687100 -2.62479400

C -0.95823900 3.09630200 -2.99318500

H -1.18031900 0.96964400 -2.59656000

H -0.92689800 5.24786300 -3.15112500

H -0.23871800 2.95474000 -3.80144600

H -2.59552600 5.60823900 -1.32613100

H -1.60662700 1.41288200 1.25392700

B -1.14485500 -4.27223300 0.63338400

O -0.51532100 -5.45089800 0.10645500

H -0.77448700 -6.22226500 0.62262600

C -0.57202200 -3.71979900 2.05371100

C 0.80357200 -3.47633000 2.23506600

C -1.40767100 -3.49423200 3.16167100

C 1.32129200 -3.03699800 3.45663700

H 1.48761100 -3.64813800 1.40165300

C -0.90471400 -3.04803200 4.38987000

H -2.47891300 -3.68867200 3.05964400

C 0.46648800 -2.82197400 4.54378100

H 2.39532700 -2.86052400 3.56361700

H -1.58192100 -2.88921600 5.23434900

H 0.86646200 -2.47786100 5.50118000

O -0.87554300 -3.19847000 -0.40414800

H -1.31042600 -3.46836900 -1.22480200

F -2.57423300 -4.43439300 0.71505800

**int8**

0 1

C -1.64941400 0.78370700 -2.34843700

C -0.94165300 0.40968700 -3.49738100

H -0.65482500 -0.63282500 -3.62854400

C -0.58061000 1.37831100 -4.43754300

H -0.02588600 1.07733800 -5.32930400

C -0.88664700 2.72771300 -4.22217400

H -0.56446200 3.48977600 -4.93591600

C -1.59831900 3.08417700 -3.07758400

C -2.00984200 4.46123300 -2.60929700

H -1.21572600 5.21078700 -2.74955700

H -2.88874100 4.81940600 -3.17425500

C -2.35726300 4.21959200 -1.12890900

H -3.08098100 4.93792600 -0.71803400

H -1.44310400 4.27894100 -0.51752800

C -2.88348400 2.74737500 -1.07772400

C -2.01736900 2.11297100 -2.15042600

C -4.40072400 2.70842500 -1.43921900

H -4.66360500 1.67635100 -1.72072500

H -4.64818800 3.36002000 -2.28965700

C -5.11789900 3.09732200 -0.13005900

H -6.11899400 2.64724100 -0.03897600

H -5.25510200 4.19040300 -0.05584700

C -4.16561800 2.60045100 0.93615300

C -4.38128500 2.46907600 2.30800600

H -5.34141500 2.74676400 2.74952700

C -3.34332200 1.98170100 3.11152800

H -3.48193000 1.89531600 4.19177800

C -2.14516300 1.55077000 2.53638700

H -1.34654400 1.12287600 3.14005300

C -1.95666800 1.64419300 1.15210600

C -2.93206500 2.25222800 0.35810400

O -0.82560300 1.09866400 0.58668400

O -1.94492000 -0.16051700 -1.38368500

P -0.83738000 -0.42591100 -0.14874200

N -1.80568200 -1.45556500 0.75158700

C -1.24923100 -2.41003000 1.74054200

C -0.02869900 -3.18947600 1.24793300

C -0.96837800 -1.74373900 3.09384200

H -2.04398600 -3.15614900 1.89534900

C 0.46012100 -4.19254800 2.29386700

H 0.79257500 -2.49398000 1.01544000

H -0.27241300 -3.69462000 0.30574800

C -0.45291200 -2.74027700 4.13580400

H -0.22008800 -0.95438000 2.93342800

H -1.87515600 -1.24220300 3.45755300

C 0.77043500 -3.50756200 3.62688300

H 1.34768200 -4.71635200 1.90495600

H -0.31400100 -4.96609100 2.45356800

H -0.22061100 -2.21288900 5.07632800

H -1.25702800 -3.46018300 4.37602700

H 1.10189900 -4.24485100 4.37651000

H 1.61005500 -2.80234100 3.48660400

C -3.27997700 -1.48137600 0.56173300

C -3.70869300 -2.67327200 -0.30418400

C -4.08564000 -1.42541900 1.86562700

H -3.53850200 -0.57216800 0.00897000

C -5.21249900 -2.62739900 -0.59612300

H -3.45800200 -3.61587500 0.21455700

H -3.12961600 -2.66258200 -1.23843200

C -5.58494400 -1.35395800 1.55414200

H -3.89235700 -2.32144600 2.47965400

H -3.77897400 -0.54953000 2.45093500

C -6.03798700 -2.53538500 0.69142600

H -5.51179200 -3.50863400 -1.18695600

H -5.42597000 -1.74374300 -1.22511900

H -6.16076100 -1.31615700 2.49346600

H -5.79423100 -0.40742800 1.02330200

H -7.11182700 -2.45211200 0.45518800

H -5.91534200 -3.47055600 1.26814600

Pd 1.20727500 -0.67145100 -1.15025300

C 2.31591300 0.92332200 -2.13009200

C 3.08354600 -0.26498000 -2.25148900

C 4.06041100 -0.60712100 -1.21001900

C 4.10901600 0.13388500 -0.00445500

C 3.18564200 1.33450100 0.20642600

C 2.08803200 1.42741300 -0.84421400

H 4.89808500 -2.26211700 -2.30851200

H 1.73476600 1.29080900 -2.97811000

H 3.19900700 -0.73642600 -3.22816800

C 4.94178300 -1.69004000 -1.37907400

C 5.02849200 -0.22439600 0.98395900

C 5.89995300 -1.30227600 0.80401600

C 5.85503300 -2.03616900 -0.38412400

H 5.05852300 0.34502700 1.91578100

H 6.61036600 -1.56619700 1.59061300

H 6.53178100 -2.88036500 -0.53609800

C 4.02143200 2.65492200 0.06555700

N 3.87088900 3.37360800 1.24158600

O 4.66048400 3.00717600 -0.89757000

C 4.48553300 4.65825000 1.46908900

H 5.03313300 4.92877000 0.55684200

H 3.72519100 5.42830600 1.68127300

H 5.18960600 4.61763400 2.31720900

C 3.09010500 2.67800400 2.17234800

C 2.66586100 1.45529500 1.62510700

C 2.75617600 3.05518000 3.47164600

C 1.92062800 0.57601100 2.39074400

C 1.99179500 2.15870800 4.23368000

C 1.58400400 0.92978000 3.70778800

H 1.61416800 -0.38075600 1.96724100

H 1.72104300 2.42600500 5.25792200

H 1.00718500 0.24003200 4.32677300

H 3.08382800 4.00853200 3.88910600

H 1.37857400 2.24553700 -0.70985000

C 0.93320000 -2.62506500 -1.75849600

C -0.25474000 -3.09967800 -2.34164100

C 1.93924400 -3.56950300 -1.48805800

C -0.43922100 -4.45787700 -2.62992400

H -1.07806000 -2.41298900 -2.55257800

C 1.76029500 -4.92948000 -1.76649200

H 2.87991400 -3.25197100 -1.02903400

C 0.56669400 -5.38230800 -2.33721200

H -1.38037100 -4.79415100 -3.07537800

H 2.55915000 -5.64015300 -1.53369300

H 0.42347600 -6.44425100 -2.55300500

**L**

0 1

C -1.69088700 -1.74054000 0.87013100

C -1.78730800 -3.03211600 1.40528500

H -0.90196800 -3.47270900 1.86768700

C -2.99019400 -3.73776700 1.32297100

H -3.05305400 -4.74501200 1.74186300

C -4.09848300 -3.17725400 0.67613600

H -5.02640700 -3.74552400 0.57463900

C -3.99073900 -1.89337100 0.14316800

C -5.00950600 -1.10771300 -0.65199600

H -5.55324900 -1.73233600 -1.37758000

H -5.76839500 -0.66132700 0.01543200

C -4.14395100 -0.02167000 -1.31900800

H -4.69687800 0.89415500 -1.57311400

H -3.70341000 -0.41693300 -2.24752100

C -2.98544800 0.24085900 -0.30250800

C -2.80160900 -1.15426200 0.26382200

C -3.44894200 1.26237900 0.78174900

H -2.77948100 1.17115900 1.65179500

H -4.47575000 1.07320100 1.12690600

C -3.25977200 2.63981700 0.11688700

H -3.06054900 3.44480000 0.84160800

H -4.15929700 2.93921800 -0.44943500

C -2.09141100 2.40124400 -0.81439900

C -1.29347500 3.34245200 -1.46493400

H -1.49050100 4.41149600 -1.35382400

C -0.23255300 2.89314400 -2.25967000

H 0.39825300 3.61230900 -2.78772500

C 0.06246200 1.53058500 -2.33812600

H 0.92464900 1.16770200 -2.89937100

C -0.72574200 0.59097000 -1.65868100

C -1.85284800 1.02203400 -0.94943400

O -0.33463700 -0.72408800 -1.65442600

O -0.48496700 -1.07782900 0.88980900

P 0.59068900 -1.40292500 -0.39109600

N 1.74638600 -0.26145600 0.07023200

C 3.16254400 -0.62366300 -0.12521000

C 3.56758500 -1.93539100 0.56753800

C 3.55719600 -0.64180100 -1.60929500

H 3.74826900 0.17474600 0.35393200

C 5.06172700 -2.22039900 0.38700500

H 2.98269200 -2.76672200 0.13828500

H 3.30063900 -1.87728300 1.63485000

C 5.04712300 -0.94394600 -1.79626000

H 2.95296500 -1.40916500 -2.12684100

H 3.29036700 0.32600500 -2.06288800

C 5.44974500 -2.24572800 -1.09528500

H 5.32671500 -3.17425400 0.87186400

H 5.64742100 -1.43654100 0.90232700

H 5.29403100 -0.99008800 -2.86963300

H 5.63926000 -0.10961000 -1.37671900

H 6.53218200 -2.42280500 -1.20690900

H 4.93984800 -3.09421900 -1.58689000

C 1.43464700 0.95704300 0.84145200

C 1.66112100 0.76984100 2.34965900

C 2.16561700 2.21121200 0.34259200

H 0.36153200 1.13968900 0.70168000

C 1.19069000 2.00493000 3.12554800

H 2.73636300 0.59771400 2.54098400

H 1.12023500 -0.12837100 2.68001200

C 1.67827000 3.44912600 1.10372500

H 3.25327700 2.10557100 0.49860100

H 2.00203200 2.33091000 -0.73611300

C 1.86200200 3.28484400 2.61626300

H 1.37772100 1.87399700 4.20423800

H 0.09549300 2.09925200 3.00842500

H 2.21121700 4.34593500 0.74653200

H 0.60991300 3.60881800 0.87460800

H 1.46568200 4.16497800 3.14961800

H 2.94282400 3.24150200 2.84567000

**PdL2**

0 1

C -3.00991200 -1.05653900 1.84863100

C -1.97468000 -1.21487500 2.77914900

H -1.38400500 -0.34142500 3.05886800

C -1.69350600 -2.47991600 3.30100400

H -0.88097100 -2.59536900 4.02198700

C -2.41175500 -3.60187900 2.86858400

H -2.15802300 -4.59729800 3.24107600

C -3.43260600 -3.43332200 1.93356600

C -4.27961400 -4.48259200 1.24842300

H -3.69376900 -5.36661600 0.95170100

H -5.08163500 -4.84142400 1.91768200

C -4.85620300 -3.71405900 0.04229900

H -5.81531200 -4.11157700 -0.32021600

H -4.14030500 -3.74769300 -0.79264600

C -4.96983700 -2.23437800 0.53197900

C -3.76199400 -2.15752400 1.44587000

C -6.30384600 -2.02700700 1.31565300

H -6.19796200 -1.13104200 1.94732700

H -6.54028400 -2.87550200 1.97402600

C -7.35997700 -1.77311400 0.22338200

H -8.19846600 -1.14936300 0.57056600

H -7.79812100 -2.71996700 -0.13889200

C -6.55392200 -1.10524500 -0.86842800

C -7.01378000 -0.39153600 -1.97507200

H -8.08515500 -0.26621700 -2.14984500

C -6.08009500 0.16351300 -2.85833000

H -6.41967400 0.71628100 -3.73742300

C -4.71215500 0.05465300 -2.59936900

H -3.97054100 0.52236500 -3.24816800

C -4.26035100 -0.63405500 -1.46632700

C -5.17874300 -1.27382700 -0.62827200

O -2.91609800 -0.63253600 -1.17618500

O -3.20860000 0.17026500 1.24505000

P -2.20755000 0.45568100 -0.08918800

N -2.73178900 2.00055000 -0.46263100

C -1.70509100 3.01644600 -0.80750700

C -0.74528300 3.33760300 0.35114600

C -0.94148200 2.64045500 -2.08697900

H -2.26616400 3.93773100 -1.02480800

C 0.28919500 4.39238200 -0.04673700

H -0.21012400 2.41348100 0.63914400

H -1.32545700 3.65815700 1.23058000

C 0.11912600 3.68120900 -2.45706000

H -0.44383700 1.66727300 -1.92334600

H -1.66455100 2.49874000 -2.90709100

C 1.06987000 3.94699800 -1.28576200

H 0.97311400 4.57121400 0.79634200

H -0.21638500 5.35411800 -0.25802000

H 0.68072800 3.33911300 -3.34175800

H -0.37556300 4.62800800 -2.74575900

H 1.81761800 4.70837200 -1.56509300

H 1.62365800 3.02149200 -1.05146300

C -4.11350000 2.44420400 -0.19669400

C -4.25523300 3.21164300 1.12622100

C -4.72981500 3.24679000 -1.34988800

H -4.71270900 1.52899000 -0.09481800

C -5.72950400 3.51262800 1.41795100

H -3.68858300 4.15797100 1.06451600

H -3.81079700 2.61448100 1.93532800

C -6.20965900 3.52880500 -1.06922000

H -4.20024300 4.20761100 -1.46829800

H -4.60888700 2.69404900 -2.29112200

C -6.39418600 4.26712600 0.26130300

H -5.82371100 4.08481200 2.35570100

H -6.26092000 2.55719300 1.58108800

H -6.64701800 4.11208900 -1.89644600

H -6.75494000 2.56886200 -1.03953200

H -7.46597100 4.42221300 0.46937600

H -5.94285800 5.27328100 0.18061600

Pd -0.00002400 -0.00011700 0.09099000

C 3.00992200 1.05656600 1.84862200

C 1.97472600 1.21490200 2.77918000

H 1.38406400 0.34144900 3.05891300

C 1.69359100 2.47994300 3.30105200

H 0.88108900 2.59541200 4.02207000

C 2.41183800 3.60189900 2.86860200

H 2.15812900 4.59732200 3.24109800

C 3.43263200 3.43333700 1.93352900

C 4.27960200 4.48260400 1.24833400

H 3.69373800 5.36662400 0.95163200

H 5.08165200 4.84144500 1.91755400

C 4.85614300 3.71406900 0.04219200

H 5.81524200 4.11158200 -0.32035600

H 4.14021700 3.74770000 -0.79272900

C 4.96980000 2.23439100 0.53187600

C 3.76199400 2.15753400 1.44581400

C 6.30384200 2.02703200 1.31549900

H 6.19798900 1.13107400 1.94718800

H 6.54031000 2.87553200 1.97385500

C 7.35993100 1.77312500 0.22318800

H 8.19843300 1.14937900 0.57035000

H 7.79806200 2.71997100 -0.13912100

C 6.55383100 1.10523400 -0.86857500

C 7.01363500 0.39149800 -1.97522900

H 8.08500100 0.26616800 -2.15005600

C 6.07990700 -0.16356500 -2.85842700

H 6.41944300 -0.71635100 -3.73752500

C 4.71197500 -0.05469200 -2.59941100

H 3.97034200 -0.52242000 -3.24817700

C 4.26022400 0.63404300 -1.46636700

C 5.17866700 1.27382300 -0.62836900

O 2.91598800 0.63255900 -1.17615700

O 3.20858700 -0.17025600 1.24505200

P 2.20751000 -0.45572400 -0.08914600

N 2.73181400 -2.00057600 -0.46257200

C 1.70514600 -3.01643100 -0.80761600

C 0.74523500 -3.33767200 0.35094200

C 0.94162100 -2.64030800 -2.08709300

H 2.26620800 -3.93770500 -1.02497400

C -0.28921100 -4.39242200 -0.04708800

H 0.21006100 -2.41355900 0.63895800

H 1.32534700 -3.65827700 1.23039600

C -0.11893900 -3.68105100 -2.45734400

H 0.44395100 -1.66715000 -1.92339300

H 1.66473800 -2.49850400 -2.90714700

C -1.06977800 -3.94695500 -1.28615200

H -0.97320700 -4.57130500 0.79592200

H 0.21637900 -5.35414800 -0.25838700

H -0.68047200 -3.33888400 -3.34205800

H 0.37578300 -4.62782000 -2.74608100

H -1.81748600 -4.70831800 -1.56562100

H -1.62360600 -3.02148000 -1.05182200

C 4.11353500 -2.44421000 -0.19660100

C 4.25527500 -3.21159900 1.12634900

C 4.72993900 -3.24678900 -1.34975200

H 4.71270300 -1.52897000 -0.09473700

C 5.72955100 -3.51249400 1.41813600

H 3.68867900 -4.15795900 1.06465200

H 3.81077600 -2.61443500 1.93542100

C 6.20979400 -3.52869400 -1.06902000

H 4.20044900 -4.20765400 -1.46814800

H 4.60901000 -2.69408900 -2.29100900

C 6.39431900 -4.26697400 0.26152600

H 5.82376300 -4.08465500 2.35590000

H 6.26090800 -2.55702600 1.58127600

H 6.64722200 -4.11196800 -1.89621700

H 6.75500600 -2.56871000 -1.03934100

H 7.46610700 -4.42198900 0.46963800

H 5.94305700 -5.27315800 0.18084500

**PhB(OH)_2_**

0 1

B 1.77722000 0.00013900 0.00004100

O 2.49848300 -1.08420300 -0.40303400

H 1.94484400 -1.78363900 -0.76721300

O 2.49829900 1.08423500 0.40317400

H 1.94414600 1.78352600 0.76698500

C 0.19845600 0.00009300 -0.00008700

C -0.53102000 -1.18362400 0.21780000

C -0.53123200 1.18372000 -0.21798100

C -1.92759500 -1.18843100 0.22422800

H 0.00216800 -2.12152800 0.40457700

C -1.92770800 1.18830200 -0.22420700

H 0.00188900 2.12161300 -0.40504900

C -2.62904400 -0.00014500 0.00009900

H -2.47118100 -2.11896800 0.40571100

H -2.47152500 2.11870400 -0.40566300

H -3.72184300 -0.00016000 0.00021500

**PhB(OH)_2_F-anion**

-1 1

B -1.57476700 0.00947400 -0.02950100

O -2.03387500 0.17685300 -1.39387400

H -2.96889700 -0.06364400 -1.37517600

O -2.13522600 -1.23480000 0.52214600

H -1.95652200 -1.22171100 1.46936700

C 0.07070200 0.00437100 -0.00820200

C 0.82139100 1.19430500 0.01427800

C 0.80022000 -1.19774900 -0.01845200

C 2.22057200 1.19162400 0.00707200

H 0.27945500 2.14458300 0.04846400

C 2.19995300 -1.22077800 -0.02359000

H 0.23240200 -2.13381800 -0.01118800

C 2.92040800 -0.02104000 -0.01416200

H 2.77385600 2.13805900 0.02270700

H 2.73598600 -2.17723200 -0.03287100

H 4.01574400 -0.03023600 -0.01867700

F -2.00942700 1.11731300 0.80856000

**TS-S1**

0 1

C -0.34103500 2.36842700 1.67477100

C 0.36805500 1.63076700 2.62977000

H 1.24272200 1.06616700 2.30936100

C -0.07431300 1.60197000 3.95380900

H 0.47283500 1.00625400 4.68029700

C -1.23539100 2.28285500 4.33179700

H -1.60648100 2.21841900 5.35741100

C -1.93367400 3.00995600 3.37202900

C -3.25275600 3.72811400 3.52432000

H -3.95997200 3.17489900 4.16084600

H -3.11085000 4.72197800 3.98540400

C -3.72415000 3.84899700 2.06697500

H -4.41877600 4.68172000 1.88751900

H -4.23362000 2.92004800 1.76597500

C -2.40591100 3.97945000 1.22968300

C -1.48227900 3.08568200 2.04083300

C -1.95566500 5.47027700 1.18746000

H -0.90524600 5.51024900 0.85716900

H -2.01496500 5.95698800 2.17168000

C -2.87084700 6.11182100 0.12250100

H -2.39280200 6.95399300 -0.40277000

H -3.79726700 6.50761000 0.57354200

C -3.17003100 4.95543900 -0.80887900

C -3.78410700 4.97681800 -2.06059200

H -4.13684800 5.91655500 -2.49234000

C -3.94052000 3.77044500 -2.75514600

H -4.43947500 3.75925700 -3.72706200

C -3.40595300 2.58515400 -2.24407400

H -3.46533000 1.64839200 -2.79689800

C -2.75627700 2.57970400 -1.00108300

C -2.72550600 3.74948200 -0.23777100

O -2.12160700 1.43760400 -0.58312300

O 0.08551100 2.35609900 0.36847200

P -0.44269700 1.21049700 -0.73815100

N 0.06782500 2.08212200 -2.08782900

C 0.39223000 1.39694200 -3.35130900

C 1.59649400 0.45585700 -3.26729300

C -0.82206000 0.68790700 -3.95797000

H 0.67380400 2.19716700 -4.05057300

C 1.92786600 -0.12551700 -4.64404400

H 1.37187300 -0.35574400 -2.56156900

H 2.45797800 0.99599800 -2.85041600

C -0.49820600 0.07830100 -5.32368700

H -1.15983300 -0.10090500 -3.26632100

H -1.64825500 1.40756500 -4.04354500

C 0.72065300 -0.84485700 -5.25569600

H 2.79146900 -0.80618700 -4.56918700

H 2.24099600 0.69193300 -5.31992100

H -1.37500800 -0.46805000 -5.70916900

H -0.29752500 0.89256700 -6.04447800

H 0.96968200 -1.22995600 -6.25840000

H 0.46820200 -1.72204100 -4.63604600

C 0.32587800 3.54012100 -2.03168200

C 1.82286600 3.85202600 -1.91129500

C -0.31854100 4.33700900 -3.17237200

H -0.14420000 3.89963400 -1.11204800

C 2.05207100 5.35399700 -1.71391600

H 2.34588600 3.51387400 -2.82478100

H 2.23618700 3.28156100 -1.06753400

C -0.10280100 5.83815000 -2.94836500

H 0.12758700 4.06140000 -4.14331900

H -1.38924900 4.10433600 -3.22699600

C 1.38557200 6.17936400 -2.81979200

H 3.13150900 5.57226200 -1.66216100

H 1.62780800 5.65021000 -0.73697600

H -0.56276900 6.41149200 -3.77002100

H -0.62949000 6.14160200 -2.02631800

H 1.51866800 7.25720000 -2.62800300

H 1.88809300 5.96884400 -3.78196200

Pd 0.04665400 -1.04332400 -0.18756700

C -5.81303700 -4.56863900 0.12562300

C -7.14195900 -4.31034900 -0.12891800

C -7.72662200 -3.07165500 0.25088100

C -6.92117800 -2.08603300 0.91580700

C -5.53746600 -2.37417500 1.14238700

C -5.00853500 -3.59230400 0.75456800

H -9.69771800 -3.54043200 -0.52321800

H -5.36955800 -5.52367800 -0.16509100

H -7.76806900 -5.05469700 -0.62786100

C -9.09353700 -2.78288200 -0.01697100

C -7.52712900 -0.85179500 1.28707500

H -3.95101900 -3.79797100 0.93050800

C -8.85431400 -0.60382900 1.00696000

C -9.64768200 -1.57606500 0.34919300

H -6.92929900 -0.11317500 1.81992500

H -9.30144000 0.34878100 1.30136800

H -10.69882000 -1.36650800 0.13659800

C -4.65568800 -1.37822100 1.84692800

N -3.43888100 -1.08776500 1.25820200

O -4.96664700 -0.89077700 2.92442400

C -2.49988700 -0.33255000 2.08203900

H -2.46966000 -0.76115600 3.09043900

H -1.50054600 -0.38069500 1.63696900

H -2.80011600 0.71999600 2.15983800

C -3.20243400 -1.30505400 -0.12075500

C -2.02986200 -1.93987800 -0.59529400

C -4.16234100 -0.91531900 -1.06200600

C -1.94517600 -2.34753000 -1.94100500

C -4.04587200 -1.24679400 -2.41199600

C -2.94099800 -1.98505900 -2.84672300

H -1.09728700 -2.94543100 -2.27031500

H -4.82684000 -0.94767100 -3.11372500

H -2.84668900 -2.28422800 -3.89324000

H -5.02649300 -0.34996200 -0.71101800

Br -1.08937000 -3.25390800 0.78898900

C 2.79637100 -1.43740700 2.76607000

C 1.79278600 -2.18320000 3.39876200

H 1.32614800 -3.00513300 2.85413700

C 1.40331300 -1.86055300 4.70007800

H 0.62742800 -2.45229300 5.19087900

C 1.97109000 -0.76295700 5.35971600

H 1.63590400 -0.48811700 6.36295900

C 2.92787000 0.00531700 4.69707500

C 3.56785100 1.30569300 5.12789300

H 2.83914800 1.99706400 5.57893600

H 4.35616600 1.13406500 5.88167100

C 4.15529700 1.84035500 3.80653300

H 5.01519000 2.51114700 3.94232200

H 3.38035700 2.39440500 3.25455400

C 4.51929000 0.55519600 2.99098700

C 3.37788200 -0.35102700 3.41432000

C 5.89606900 0.00434800 3.47459600

H 5.99503900 -1.03522200 3.12407000

H 5.98575600 0.00007300 4.57005500

C 6.92989400 0.89837000 2.76858700

H 7.89081400 0.39130200 2.58959000

H 7.15330600 1.80215700 3.36172300

C 6.22895800 1.25670100 1.47746600

C 6.79024000 1.81820800 0.33298800

H 7.84999400 2.08295400 0.30850100

C 5.97590400 2.03027500 -0.78569000

H 6.38655900 2.48702000 -1.68885900

C 4.65076200 1.60032600 -0.77292200

H 4.02397600 1.67477400 -1.66045800

C 4.10672900 0.99206100 0.36893500

C 4.86261400 0.90690100 1.54465600

O 2.84512100 0.45158800 0.24956400

O 3.20202300 -1.76470200 1.49896500

P 2.40153500 -1.16447700 0.12837600

N 3.39062700 -1.92997900 -1.00764800

C 2.90391000 -3.23674300 -1.52937000

C 2.48064400 -4.22572300 -0.42994400

C 1.81132100 -3.07897000 -2.58511000

H 3.77075600 -3.67975600 -2.04262300

C 2.05325700 -5.57197400 -1.02045400

H 1.63124500 -3.79952900 0.12685500

H 3.29946400 -4.35286500 0.29205400

C 1.35444500 -4.42509800 -3.15298600

H 0.95667500 -2.55310200 -2.12931900

H 2.18977500 -2.43145900 -3.38421500

C 0.93204400 -5.39781900 -2.04833900

H 1.73330400 -6.24903300 -0.21181200

H 2.92276700 -6.05369600 -1.50576800

H 0.53073000 -4.26724400 -3.86969900

H 2.18326400 -4.87325900 -3.73191600

H 0.65069500 -6.37121800 -2.48265300

H 0.03539300 -5.00750300 -1.53698000

C 4.85871200 -1.75284100 -1.01396900

C 5.65191200 -2.85354300 -0.29143800

C 5.37464100 -1.52653900 -2.44104400

H 5.06871500 -0.83475800 -0.46064900

C 7.14743800 -2.51705700 -0.29343300

H 5.50291200 -3.82719200 -0.78931300

H 5.27314600 -2.94939500 0.73576100

C 6.87977000 -1.23902000 -2.45565300

H 5.16403800 -2.41832200 -3.05801200

H 4.81359000 -0.69181700 -2.88960000

C 7.67324800 -2.32259000 -1.71897700

H 7.71367800 -3.30927500 0.22340600

H 7.30765100 -1.58745900 0.28302900

H 7.23466400 -1.13831800 -3.49474900

H 7.05997000 -0.26717200 -1.96538500

H 8.74576000 -2.06702800 -1.70392300

H 7.58606000 -3.27832200 -2.26815500

**TS-S10**

0 1

C 1.48437300 -1.38735200 -1.70133200

C 0.58754000 -1.71401200 -2.72716000

H 0.03260200 -0.90881600 -3.21083900

C 0.39285900 -3.04994100 -3.08609400

H -0.30981000 -3.29551000 -3.88613500

C 1.05599200 -4.07420000 -2.39769800

H 0.86448700 -5.12143600 -2.64453400

C 1.95279900 -3.73621400 -1.38437200

C 2.74292900 -4.64623100 -0.46993900

H 2.15100600 -5.50232600 -0.11019400

H 3.61904700 -5.06540400 -0.99577000

C 3.17365000 -3.69247500 0.66202400

H 4.09362100 -4.00294600 1.17823700

H 2.37281500 -3.62293300 1.41487200

C 3.31544200 -2.29863300 -0.02893400

C 2.20420000 -2.39224700 -1.05801100

C 4.71519800 -2.17455300 -0.71030500

H 4.65740000 -1.38131400 -1.47231500

H 5.02232500 -3.10199600 -1.21500300

C 5.66207400 -1.73969000 0.42366700

H 6.52366900 -1.15469100 0.06575400

H 6.07287900 -2.61316400 0.96037300

C 4.75055800 -0.93836000 1.32605900

C 5.09781500 -0.05826100 2.35116800

H 6.14676700 0.11750400 2.60180600

C 4.07972200 0.59720100 3.05295800

H 4.32970500 1.28225000 3.86661400

C 2.74311400 0.41868000 2.68931500

H 1.94005700 0.96114100 3.18962400

C 2.40418100 -0.44213400 1.63729200

C 3.40584500 -1.17360400 0.99026700

O 1.09538600 -0.52580900 1.23391800

O 1.57856500 -0.08458700 -1.26575300

P 0.46604600 0.37753900 -0.06938600

N 1.02367500 1.95067700 0.09836500

C 0.06135500 3.02562000 0.41586300

C -0.98743200 3.27966600 -0.67599700

C -0.60619500 2.82510500 1.78313600

H 0.66568000 3.94089200 0.49284400

C -1.87273600 4.47763800 -0.31445500

H -1.60958000 2.38133900 -0.79652400

H -0.47966900 3.44803900 -1.63872400

C -1.47412500 4.03153500 2.15075800

H -1.22867300 1.91346000 1.74499200

H 0.17127900 2.65128500 2.54424600

C -2.51790400 4.31447000 1.06645800

H -2.65008400 4.60835400 -1.08391600

H -1.26269400 5.40071300 -0.32074600

H -1.96478800 3.86491200 3.12396500

H -0.82480100 4.91798900 2.27646800

H -3.10465900 5.21248300 1.32301700

H -3.22751800 3.47260700 1.03152900

C 2.41536100 2.33036600 -0.22767500

C 2.55035900 2.91422400 -1.64181400

C 3.06543200 3.26821300 0.79877900

H 2.99767300 1.40077300 -0.21028500

C 4.02536200 3.13856900 -1.99308200

H 2.00268600 3.87231200 -1.69546700

H 2.07897200 2.22621900 -2.35801600

C 4.54820500 3.46546400 0.46378400

H 2.56912700 4.25352300 0.78806900

H 2.94670500 2.85511200 1.80877800

C 4.73001600 4.01792600 -0.95438900

H 4.11639700 3.58131500 -2.99873100

H 4.53002100 2.15608400 -2.03771900

H 5.01516700 4.13901000 1.20142400

H 5.06706000 2.49475000 0.55452400

H 5.80186100 4.11339900 -1.19566900

H 4.30766200 5.03872000 -0.99939800

Pd -1.54064900 -0.55314500 -0.41170400

C -2.50572000 -2.60006700 -0.86956400

C -3.59846100 -1.65675800 -0.55834300

C -4.08058400 -1.68545100 0.85104300

C -3.31394900 -2.29773300 1.86744600

C -1.99988200 -2.99262900 1.56684500

C -1.70116100 -3.12888000 0.09799300

H -5.93001900 -0.68250600 0.38043500

H -2.30586200 -2.83126100 -1.91860300

H -4.40901400 -1.67700200 -1.28735800

C -5.32964200 -1.12896500 1.17573500

C -3.80326600 -2.29440800 3.17991800

C -5.03013100 -1.71058700 3.49764200

C -5.80406600 -1.13478000 2.48587300

H -3.20930100 -2.76942000 3.96612700

H -5.38835100 -1.71808100 4.52988700

H -6.77638500 -0.69360200 2.71751200

H -0.86209600 -3.76164400 -0.20015200

C -3.38127700 0.30758300 -1.19483900

C -3.34144800 0.43579200 -2.59492200

C -4.07425100 1.26853100 -0.44883200

C -3.93239400 1.53309300 -3.22610100

H -2.83740400 -0.32539200 -3.19823800

C -4.68415000 2.35630300 -1.08387000

H -4.12828400 1.17686400 0.63791600

C -4.61028800 2.49851800 -2.47214000

H -3.87241300 1.62986500 -4.31365900

H -5.21003200 3.10507500 -0.48540700

H -5.08356300 3.35079600 -2.96532600

H -1.17130200 -2.46591800 2.07354200

H -2.02045100 -3.99941500 2.02652600

**TS-S11**

0 1

C -0.66544600 -1.31883600 1.66875000

C 0.46215300 -1.53394500 2.46829800

H 1.02449400 -0.66932200 2.81930300

C 0.86549900 -2.83634500 2.77082900

H 1.75071800 -2.99361900 3.39200800

C 0.17246700 -3.93380400 2.24669500

H 0.52134500 -4.95158900 2.43721400

C -0.95347400 -3.70677200 1.45762000

C -1.81619600 -4.70949300 0.72514500

H -1.21722900 -5.48922400 0.22934500

H -2.50195300 -5.22639800 1.41963400

C -2.58765200 -3.82410700 -0.27250100

H -3.56001400 -4.23856800 -0.57578100

H -1.98214300 -3.67689200 -1.17904400

C -2.72225500 -2.44386000 0.44580300

C -1.40601400 -2.40180900 1.19843400

C -3.94360200 -2.46148700 1.41783500

H -3.81282200 -1.65012600 2.15140800

H -4.02451300 -3.40639600 1.97442200

C -5.15748100 -2.16237500 0.51754500

H -5.98255300 -1.66852700 1.05434700

H -5.57185600 -3.08902800 0.08233400

C -4.56097900 -1.28456600 -0.56049600

C -5.21873700 -0.47545700 -1.48677500

H -6.31024500 -0.42908400 -1.50936800

C -4.45639000 0.27969500 -2.38592100

H -4.95184400 0.91194400 -3.12660800

C -3.06193600 0.26999800 -2.31152000

H -2.45305800 0.89475000 -2.96658600

C -2.40942800 -0.52107500 -1.35623100

C -3.15715100 -1.35497200 -0.52001600

O -1.04540200 -0.43259500 -1.23697100

O -0.99458500 -0.03226200 1.29388500

P -0.26336900 0.56636200 -0.10772000

N -1.03779700 2.05756000 -0.12087800

C -0.25816500 3.26527700 -0.46557600

C 0.88239200 3.55692100 0.52178200

C 0.24309900 3.23565400 -1.91658400

H -0.96707100 4.10331200 -0.39503500

C 1.65920200 4.81975400 0.14339300

H 1.56950900 2.69758600 0.53021000

H 0.47209000 3.63839900 1.54013000

C 1.01019100 4.50935900 -2.27721900

H 0.90698500 2.36121100 -2.04243200

H -0.61577600 3.08278600 -2.58990800

C 2.16499200 4.75525400 -1.30112800

H 2.50061500 4.95982200 0.84185400

H 1.00822300 5.70680600 0.25709300

H 1.38395000 4.44524700 -3.31253200

H 0.32183200 5.37463800 -2.24790600

H 2.70304900 5.68102000 -1.56398600

H 2.89345400 3.92884800 -1.39308900

C -2.38416000 2.24039800 0.45998300

C -2.35163200 2.76860600 1.90225500

C -3.31079300 3.10658500 -0.40443000

H -2.83667100 1.24172200 0.49953400

C -3.76369500 2.79075300 2.49768400

H -1.92965000 3.78959700 1.91012400

H -1.68541400 2.13240000 2.50187000

C -4.72792900 3.11350000 0.17871600

H -2.93874100 4.14495800 -0.43943900

H -3.31608100 2.72882400 -1.43542300

C -4.73227100 3.60282700 1.63125200

H -3.73868100 3.18907200 3.52542700

H -4.13121300 1.75100700 2.57506400

H -5.38807700 3.74255500 -0.44129000

H -5.13754300 2.08930400 0.13215000

H -5.75193900 3.55770800 2.04888300

H -4.43209000 4.66673900 1.65479100

Pd 1.92032900 0.30179200 -0.60775200

C 3.43852800 -0.01342000 -2.22502800

C 2.47542300 -0.85407500 -2.74997800

C 2.19492100 -2.15771500 -2.13831000

C 3.04109400 -2.63359400 -1.10320000

C 4.27267100 -1.83666800 -0.73414900

C 4.11558200 -0.34348800 -0.95922200

H 0.42875500 -2.55173800 -3.31342100

H 3.67698800 0.92742600 -2.72848300

H 1.95387900 -0.58477400 -3.67053100

C 1.08937600 -2.93076600 -2.53031200

C 2.74420200 -3.84733600 -0.48333900

C 1.63965500 -4.60803400 -0.87955000

C 0.81643700 -4.14972300 -1.91093800

H 3.38758600 -4.20386500 0.32571300

H 1.42100200 -5.55356000 -0.37876300

H -0.04632100 -4.74023600 -2.22770100

H 5.04908200 0.20409100 -0.82602700

C 3.52588200 0.50510600 0.82724700

C 3.55291400 -0.35086600 1.93823600

C 3.99758800 1.82075000 0.98815500

C 3.97939200 0.11192200 3.18799300

H 3.20776800 -1.38069200 1.83955000

C 4.41577000 2.28677500 2.23658400

H 4.03567200 2.49423900 0.12721900

C 4.40385000 1.43419300 3.34645600

H 3.97151100 -0.56700900 4.04560000

H 4.76058400 3.31924700 2.34090600

H 4.73507200 1.79469300 4.32317300

H 5.11251900 -2.17172800 -1.37665100

H 4.57935400 -2.05196200 0.29835700

**TS-S3**

-1 1

C 1.26417100 -0.86484400 -2.46267200

C 0.57016900 -0.10863700 -3.41605400

H 0.89249800 0.91468100 -3.61149700

C -0.56221500 -0.64246100 -4.03858800

H -1.10621600 -0.03457000 -4.76559300

C -1.05383000 -1.90131600 -3.66937700

H -1.99717200 -2.27237300 -4.07437600

C -0.35796800 -2.64025900 -2.71218300

C -0.75486600 -3.94486600 -2.06289600

H -1.81637700 -3.91407700 -1.77588700

H -0.60787400 -4.79927100 -2.74778400

C 0.19739600 -4.02247000 -0.84988700

H 0.43046300 -5.04962700 -0.53072400

H -0.25691500 -3.50413800 0.00915000

C 1.47248500 -3.23319200 -1.29170800

C 0.83600800 -2.15513900 -2.15144800

C 2.38496300 -4.15147300 -2.16658200

H 3.05311000 -3.50623000 -2.75866200

H 1.80444500 -4.76766100 -2.86779700

C 3.20867500 -4.96503400 -1.15455900

H 4.18917200 -5.28191900 -1.54481400

H 2.67465400 -5.88345300 -0.85134200

C 3.33106100 -4.01036100 0.01163400

C 4.20375300 -4.08942800 1.09566500

H 4.91020600 -4.91944200 1.17963100

C 4.15660400 -3.08852200 2.07447000

H 4.81808900 -3.13646800 2.94333600

C 3.28951900 -2.00680200 1.92905500

H 3.25182500 -1.19950000 2.65818900

C 2.44389100 -1.91158900 0.81257700

C 2.40987200 -2.95222100 -0.12396800

O 1.65595400 -0.80660200 0.69508500

O 2.28297300 -0.27710500 -1.73760500

P 1.78511800 0.53910700 -0.32362200

N 3.27095500 1.23588800 0.05971200

C 3.39162900 2.71213800 0.03928000

C 3.13531700 3.33399900 -1.34188600

C 2.52845000 3.39080900 1.10938700

H 4.44186400 2.91714400 0.30674400

C 3.36682100 4.84834100 -1.32049300

H 2.08977600 3.14389200 -1.63234200

H 3.77908100 2.85261400 -2.09285900

C 2.76252100 4.90439100 1.13146300

H 1.46792500 3.19515800 0.88581500

H 2.75193400 2.93718000 2.08734600

C 2.51069400 5.52580900 -0.24587500

H 3.13444000 5.27070000 -2.31188300

H 4.43783500 5.05856000 -1.12801000

H 2.11021800 5.37141400 1.88863800

H 3.80482100 5.10973800 1.44585200

H 2.71251800 6.61078600 -0.21924800

H 1.44887100 5.39203600 -0.51232800

C 4.51653400 0.45673900 -0.03553100

C 5.33819000 0.72537500 -1.30659800

C 5.38918100 0.60443500 1.21944800

H 4.22134700 -0.59838800 -0.09295100

C 6.54950000 -0.21142200 -1.37067300

H 5.68695800 1.77257500 -1.31461100

H 4.68846200 0.58684700 -2.18213900

C 6.60753000 -0.32176100 1.15426200

H 5.73069500 1.65013300 1.31327000

H 4.77939400 0.38994800 2.10912700

C 7.42570000 -0.08593600 -0.11988500

H 7.14170000 -0.00614700 -2.27844800

H 6.19268600 -1.25385600 -1.45877200

H 7.23885200 -0.18431400 2.04878400

H 6.25838000 -1.36893400 1.17272900

H 8.27247400 -0.79179900 -0.16920700

H 7.86448800 0.92893100 -0.08747800

Pd -0.32535500 1.34345000 -0.55230600

C -2.18158700 0.02913300 -0.77072700

C -3.34436400 0.79100600 -0.48877800

C -3.54366300 1.25282200 0.89549300

C -2.82661300 0.66245900 1.95647800

C -1.69127900 -0.32599800 1.69911700

C -1.28134800 -0.40208600 0.22496100

H -5.04475900 2.69553300 0.35252500

H -2.01003200 -0.31931900 -1.78319900

H -3.71148000 1.43685100 -1.28363000

C -4.49903400 2.24305600 1.18183700

C -3.13432500 1.02801500 3.27309200

C -4.11441900 1.98108000 3.54829700

C -4.78580300 2.60711500 2.49245800

H -2.57930400 0.57120500 4.09567200

H -4.34152000 2.24623700 4.58402500

H -5.54233600 3.36948600 2.69392300

C -2.05332700 -1.80047600 2.06355700

N -1.09692800 -2.26271000 2.94664800

O -2.95430200 -2.48735900 1.61874300

C -1.02158000 -3.63153300 3.39080800

H -1.83213400 -4.18445500 2.89858000

H -0.05102400 -4.07535300 3.11296300

H -1.14300100 -3.70438200 4.48505600

C -0.20669100 -1.24627900 3.32398900

C -0.51638100 -0.07051300 2.62833500

C 0.82733600 -1.30455400 4.25318600

C 0.20695500 1.08622800 2.86184900

C 1.55873800 -0.12773700 4.48035500

C 1.25516900 1.05244600 3.79628500

H -0.03851800 1.99969800 2.31831600

H 2.37835800 -0.13991700 5.20360100

H 1.83539100 1.95675700 3.98888700

H 1.06743300 -2.22868600 4.78111900

Br -0.56545600 3.62251100 -1.73465100

H -0.63763200 -1.24925200 -0.00291600

B -4.55285700 -1.70557100 -1.87473600

O -4.23918400 -1.05527300 -3.09251000

H -3.32612300 -0.75348800 -3.07632900

O -3.47306400 -2.38454500 -1.26069700

H -3.57232600 -2.51959700 -0.30783700

C -5.19423200 -0.33701600 -0.64865800

C -6.09437900 0.53494000 -1.29880200

C -5.57777000 -0.81650500 0.62263900

C -7.31161100 0.90082600 -0.72796700

H -5.82477300 0.90615000 -2.29285500

C -6.79633900 -0.46924800 1.20200800

H -4.89469300 -1.46566300 1.17515300

C -7.66471900 0.40135600 0.53256700

H -7.99252700 1.57229800 -1.26033900

H -7.06830000 -0.86211500 2.18623900

H -8.61401500 0.69219000 0.99219300

F -5.73629400 -2.42687700 -1.97281800

**TS-S6**

0 1

C 1.65133600 -0.48129100 -2.44576600

C 0.83999200 -0.42632900 -3.58573000

H 0.36763700 0.52324000 -3.84216600

C 0.61213100 -1.58136000 -4.33838600

H -0.02336400 -1.52809000 -5.22531500

C 1.13068800 -2.81294800 -3.91924300

H 0.88875900 -3.72949100 -4.46228300

C 1.94220600 -2.85383900 -2.78601300

C 2.55320700 -4.05694300 -2.10129800

H 1.83018600 -4.88035200 -1.99510000

H 3.40843500 -4.45154900 -2.67725400

C 3.00462400 -3.47975100 -0.74420300

H 3.85112600 -4.01620200 -0.29163300

H 2.16637800 -3.50821600 -0.03024000

C 3.33587600 -1.98445900 -1.04584600

C 2.26759500 -1.67971300 -2.08293700

C 4.76247300 -1.86627700 -1.67481500

H 4.82200700 -0.90794100 -2.21416900

H 4.97069400 -2.66892800 -2.39634600

C 5.72178700 -1.84676300 -0.47077300

H 6.65585400 -1.29968200 -0.67208100

H 6.01161600 -2.86992900 -0.17247600

C 4.88597000 -1.19442400 0.60702700

C 5.30067900 -0.65065000 1.82230500

H 6.35578900 -0.66524800 2.10601200

C 4.34342400 -0.08530200 2.67440800

H 4.64706700 0.33471900 3.63611400

C 3.00398800 -0.01958600 2.28820000

H 2.24461500 0.43955200 2.91944500

C 2.60710900 -0.53644000 1.05002900

C 3.53165000 -1.17927000 0.22674100

O 1.30041900 -0.39579400 0.65132800

O 1.76508100 0.63858900 -1.65022200

P 0.68988700 0.79681000 -0.35220600

N 1.21975200 2.28215400 0.19539700

C 0.27675000 3.41055800 0.40626200

C -0.44485100 3.86253600 -0.87087500

C -0.71389900 3.15249000 1.54540800

H 0.91517200 4.24739200 0.72620900

C -1.33336100 5.08249800 -0.60635400

H -1.07344700 3.03364900 -1.23300900

H 0.29546700 4.07887400 -1.65665300

C -1.58904600 4.38089100 1.80957000

H -1.35921600 2.30880200 1.25858000

H -0.15639500 2.85146000 2.44697700

C -2.31898200 4.82844200 0.53915800

H -1.88024500 5.34643000 -1.52587300

H -0.69565800 5.95241700 -0.35745600

H -2.31511700 4.15459700 2.60757800

H -0.95711800 5.20817700 2.18557400

H -2.91008600 5.73722700 0.73952200

H -3.03086800 4.04563100 0.23509800

C 2.66331700 2.61480800 0.14647600

C 3.04750000 3.48528200 -1.05900200

C 3.17453300 3.24066700 1.45057100

H 3.19715700 1.66390600 0.02405200

C 4.56791400 3.67063300 -1.11908600

H 2.56035400 4.47229500 -0.97554400

H 2.67466200 3.01082300 -1.97756800

C 4.69569800 3.41853700 1.39588600

H 2.70436900 4.22627600 1.60744600

H 2.88495400 2.60881400 2.30088200

C 5.11732800 4.25794800 0.18510400

H 4.83872600 4.31041300 -1.97496200

H 5.03985300 2.68780400 -1.30184400

H 5.05448400 3.88177900 2.32983500

H 5.16923700 2.42282400 1.33723900

H 6.21598600 4.33857400 0.13773700

H 4.73295400 5.28725800 0.30709900

Pd -1.46882900 0.44028200 -0.76210000

C -1.89317900 -1.84420500 -1.82764800

C -3.24846700 -1.98889800 -1.77908000

C -3.97814800 -2.04627900 -0.53035400

C -3.27087400 -2.10665000 0.69252000

C -1.74999100 -2.14405600 0.68419800

C -1.12541000 -1.62110400 -0.62243900

H -5.91778100 -1.92815500 -1.46524600

H -1.37274900 -1.81269200 -2.78261400

H -3.82259500 -2.04649100 -2.70689600

C -5.38263000 -2.00511600 -0.51598500

C -3.98701900 -2.15669300 1.88954200

C -5.38535200 -2.12549400 1.89126100

C -6.08573200 -2.04025500 0.68562400

H -3.44824000 -2.20644100 2.83700800

H -5.92567100 -2.15927800 2.84012000

H -7.17698400 -1.99706200 0.68328500

C -1.21818800 -3.62756000 0.73594900

N -0.29454800 -3.69075700 1.76702300

O -1.50361600 -4.53220200 -0.01132900

C 0.44135300 -4.88747200 2.09584000

H 0.19034000 -5.64912800 1.34644500

H 1.52668900 -4.69609400 2.07636100

H 0.16788300 -5.26044300 3.09722800

C -0.26090400 -2.50073900 2.50715500

C -1.12533100 -1.55699800 1.93330700

C 0.46249000 -2.22568700 3.66400300

C -1.31164800 -0.32580500 2.53960400

C 0.28145700 -0.96767900 4.25919800

C -0.59718600 -0.02941900 3.71186800

H -2.00928900 0.39482300 2.11349100

H 0.83510900 -0.72605000 5.16970700

H -0.73559300 0.93836400 4.19790600

H 1.14112400 -2.96008100 4.09968600

H -0.06548300 -1.86659000 -0.68621100

C -3.33511000 1.50341300 -0.83229900

C -3.73701900 2.32740200 -1.90918300

C -4.24837800 1.42400900 0.24498600

C -4.94375200 3.03558200 -1.90987200

H -3.07927700 2.43771200 -2.77903100

C -5.46407800 2.11788800 0.25863900

H -4.01808400 0.79495000 1.11186000

C -5.81466400 2.93569300 -0.81934900

H -5.20794600 3.67028500 -2.76178800

H -6.13849200 2.02343900 1.11555600

H -6.75790100 3.48908200 -0.81157600

**TS11**

0 1

C 1.84364500 -1.43459600 -2.05467700

C 1.14332800 -1.05388100 -3.20513800

H 1.14042600 -0.00376400 -3.49493200

C 0.41179600 -2.00654900 -3.91771000

H -0.14251700 -1.70138400 -4.80806500

C 0.33967000 -3.32957600 -3.46623900

H -0.28210000 -4.05969800 -3.98931800

C 1.04589600 -3.69239900 -2.32123500

C 1.06133400 -5.02355300 -1.60563900

H 0.05997600 -5.47663300 -1.54448300

H 1.70769400 -5.74738100 -2.13269300

C 1.63935300 -4.65502300 -0.22648900

H 2.14689700 -5.48788900 0.28057400

H 0.83090500 -4.30427900 0.43365700

C 2.59883900 -3.45317000 -0.50835900

C 1.84026500 -2.76035100 -1.62823800

C 3.98732000 -3.97948100 -0.98961500

H 4.51483300 -3.15308800 -1.49236300

H 3.89750100 -4.80554000 -1.70927100

C 4.72579100 -4.36123800 0.30769600

H 5.82037800 -4.27715500 0.22188900

H 4.50995600 -5.40347700 0.60103500

C 4.15090400 -3.39008000 1.31469600

C 4.62495500 -3.07546900 2.58833700

H 5.51465400 -3.56901500 2.98653700

C 3.94417100 -2.11792100 3.34931400

H 4.29057000 -1.87007400 4.35522000

C 2.85173100 -1.43512100 2.80957600

H 2.34296900 -0.64290500 3.36005000

C 2.40571700 -1.73907500 1.51887900

C 3.00387500 -2.76822800 0.78842100

O 1.40015100 -0.97017800 0.96815500

O 2.49857700 -0.46594700 -1.31108600

P 1.71098500 0.30927500 -0.05781900

N 3.03190100 1.17799000 0.48314600

C 2.90522700 2.53103800 1.06972400

C 2.09807000 3.51497300 0.21617400

C 2.40535200 2.49095800 2.51898500

H 3.93150500 2.92630800 1.09005600

C 2.08342500 4.91248900 0.83692900

H 1.05779200 3.16872700 0.11217500

H 2.52202600 3.54478600 -0.80095600

C 2.34358300 3.89230300 3.13705800

H 1.40918800 2.02278200 2.53626200

H 3.06637500 1.83954300 3.11043700

C 1.54458600 4.87182200 2.27030400

H 1.47430700 5.58130300 0.20936100

H 3.10870000 5.32699500 0.84469600

H 1.91640100 3.83532200 4.15160800

H 3.37280800 4.27864100 3.25571500

H 1.56485500 5.87918500 2.71698200

H 0.48386900 4.56500300 2.24807800

C 4.40910600 0.64589600 0.31076500

C 5.11413800 1.27343300 -0.89983100

C 5.27916300 0.76025900 1.56921700

H 4.30530600 -0.42536900 0.10347500

C 6.48271200 0.62224600 -1.12954700

H 5.24128200 2.35780300 -0.72881400

H 4.47470200 1.15350500 -1.78638000

C 6.63416400 0.08298700 1.33647900

H 5.45049400 1.81978100 1.82350800

H 4.76269900 0.29854100 2.41969300

C 7.35819400 0.68122100 0.12615900

H 6.99316400 1.10157100 -1.98075300

H 6.32863900 -0.43473000 -1.41435600

H 7.25664900 0.17051500 2.24190800

H 6.46974500 -0.99721400 1.17439200

H 8.31401500 0.16016200 -0.04806600

H 7.61142800 1.73544100 0.34215600

Pd -0.23821300 1.29592300 -0.75252200

C -5.76698800 1.25209600 1.42625400

C -6.85964500 0.52745200 1.84490400

C -6.94331500 -0.86789700 1.58733800

C -5.88259700 -1.52155700 0.87043700

C -4.74758100 -0.74135500 0.46461400

C -4.70818700 0.61066800 0.74775600

H -8.85846200 -1.11880100 2.57208100

H -5.70197200 2.32508000 1.61945700

H -7.67870500 1.01179200 2.38318300

C -8.06043200 -1.63080900 2.02772600

C -6.00140100 -2.92024300 0.62427200

H -3.84528000 1.19849300 0.44932200

C -7.09678100 -3.62922700 1.07009000

C -8.13866400 -2.98306600 1.77919100

H -5.22037100 -3.41878400 0.05301100

H -7.16512700 -4.70111400 0.86817000

H -9.00159400 -3.55796700 2.12401400

C -3.63782000 -1.39895400 -0.31418400

N -2.33631400 -1.20169900 0.09025900

O -3.88789200 -2.11359000 -1.28013400

C -1.30704300 -1.90950800 -0.65723000

H -1.69676200 -2.88711100 -0.96683000

H -1.01326700 -1.37027700 -1.56972900

H -0.42850800 -2.02890300 -0.01832900

C -1.95516800 -0.34215300 1.16616500

C -1.18500900 0.81913300 0.95104700

C -2.43059000 -0.64408400 2.45159100

C -1.04263400 1.71028500 2.02460900

C -2.24037000 0.23459500 3.51575100

C -1.56804400 1.43717200 3.29179000

H -0.51938200 2.65334100 1.86660200

H -2.63551100 -0.00929700 4.50433900

H -1.44003800 2.16188900 4.10018900

H -2.99466500 -1.56820600 2.59074900

B -0.59611400 3.07361000 -2.66920800

O 0.49223800 2.06789100 -2.65278300

H 1.34100800 2.52783300 -2.57864400

O -1.14070900 3.38213800 -3.90731100

H -1.32729000 2.59403500 -4.42812400

C -2.00050800 2.17306000 -1.67014400

C -2.77624700 3.18885900 -1.06607400

C -2.69877100 1.16052200 -2.37655400

C -4.15748000 3.25228000 -1.24461500

H -2.26584100 3.97251800 -0.50218900

C -4.08390800 1.19140700 -2.52468700

H -2.14161300 0.33542700 -2.83090900

C -4.81029700 2.25552500 -1.97849900

H -4.73272400 4.07041700 -0.80410600

H -4.59769800 0.38078000 -3.04515400

H -5.89545800 2.29174000 -2.10080100

F -0.24491800 4.18954300 -1.93997900

**TS13**

0 1

C -1.90088100 1.35744700 -2.00312600

C -1.17024700 0.90877000 -3.11097900

H -1.30865000 -0.11820400 -3.45395400

C -0.25705200 1.76211400 -3.73676600

H 0.31901300 1.39806000 -4.59018500

C -0.03881000 3.05311400 -3.24118400

H 0.72135400 3.69594000 -3.69055500

C -0.77405400 3.48786900 -2.13906800

C -0.65259000 4.78319700 -1.36859400

H 0.39546200 5.08972700 -1.22920800

H -1.15811500 5.60762300 -1.90202200

C -1.36401200 4.45250600 -0.04114500

H -1.78648000 5.33059100 0.46813600

H -0.65266600 3.97399500 0.64943100

C -2.45550200 3.40264000 -0.42681000

C -1.73482900 2.65870600 -1.53529000

C -3.73517800 4.12411200 -0.95436000

H -4.31806000 3.40367600 -1.54961700

H -3.49639800 4.97908000 -1.60303100

C -4.51754300 4.50523300 0.31625700

H -5.60455100 4.56750800 0.15219500

H -4.19815800 5.48889600 0.70351800

C -4.13776600 3.40485200 1.28178200

C -4.75120600 3.05403100 2.48438900

H -5.62009800 3.60841900 2.84719100

C -4.23591200 1.98089200 3.22097300

H -4.69472800 1.69877100 4.17145900

C -3.16161000 1.23777800 2.72716900

H -2.77483700 0.36858500 3.26061200

C -2.57407300 1.57820300 1.50287800

C -3.01916700 2.70130500 0.79989300

O -1.58425200 0.76729800 0.99161200

O -2.70037000 0.46979000 -1.30546800

P -1.89213800 -0.44495100 -0.13853900

N -3.19385700 -1.35241300 0.38783600

C -2.97097000 -2.78732800 0.67068600

C -2.53162300 -3.59435300 -0.56226400

C -2.00960000 -2.99174900 1.84951900

H -3.95038800 -3.18387700 0.97703400

C -2.34578800 -5.07568700 -0.22703500

H -1.57034100 -3.18684200 -0.93053800

H -3.26641500 -3.45748300 -1.37076300

C -1.79192300 -4.47467200 2.16393000

H -1.04159100 -2.52597700 1.60310900

H -2.40198700 -2.45624600 2.72881800

C -1.35223900 -5.25921900 0.92357100

H -2.00899000 -5.62458300 -1.12147700

H -3.31968700 -5.51446400 0.06006100

H -1.04827300 -4.57848000 2.97100200

H -2.73253800 -4.91041500 2.54899900

H -1.23538000 -6.32784700 1.16706000

H -0.35911500 -4.90101100 0.59712400

C -4.57883400 -0.84046200 0.34229600

C -5.34454800 -1.31786900 -0.90024700

C -5.38389500 -1.13440600 1.61463100

H -4.49749400 0.25213400 0.26619000

C -6.72067500 -0.64792800 -0.97734400

H -5.46790500 -2.41502200 -0.85537500

H -4.74994400 -1.09052400 -1.79678400

C -6.75112200 -0.44455500 1.54645500

H -5.53900100 -2.22159100 1.72356600

H -4.82165000 -0.79749300 2.49566400

C -7.53404300 -0.87537200 0.30135800

H -7.27424200 -1.01429200 -1.85742500

H -6.57786700 0.43763600 -1.12833500

H -7.32934000 -0.66286400 2.45939700

H -6.59658100 0.64892500 1.52761300

H -8.49463200 -0.33691200 0.24668100

H -7.78238800 -1.94947300 0.38530000

Pd 0.13294500 -1.31595000 -0.83381200

C 6.36008700 -1.44258200 0.11387800

C 7.34654900 -0.82923400 0.85272400

C 7.21970100 0.53277900 1.23897600

C 6.05468400 1.27275200 0.84223200

C 5.03457900 0.59885700 0.09424800

C 5.19778900 -0.72831900 -0.25626900

H 9.10419600 0.60630200 2.30936000

H 6.45931000 -2.48840300 -0.18522100

H 8.24170100 -1.37936200 1.15458000

C 8.22180800 1.18015400 2.01382500

C 5.95265200 2.63717700 1.23855400

H 4.42065900 -1.23829300 -0.82095200

C 6.94100200 3.23163100 1.99412100

C 8.08780000 2.49907300 2.38780700

H 5.08937500 3.21627400 0.91226400

H 6.84350400 4.28032600 2.28543300

H 8.86471500 2.98388400 2.98391500

C 3.81119800 1.37296400 -0.33021600

N 2.58375000 0.91057300 0.09273200

O 3.91349000 2.38554200 -1.01135600

C 1.40126500 1.68356800 -0.27098100

H 1.72033800 2.70157700 -0.52187500

H 0.88887900 1.25845400 -1.14696300

H 0.69078700 1.68571200 0.56397400

C 2.42465100 -0.22547700 0.94035200

C 1.71771400 -1.37138300 0.51811400

C 2.99168400 -0.18433700 2.22375800

C 1.67033300 -2.47513300 1.39115800

C 2.91220000 -1.27655900 3.08326100

C 2.24852200 -2.43197200 2.65927900

H 1.17031900 -3.39056700 1.07220500

H 3.36960000 -1.22771800 4.07369700

H 2.17913600 -3.30387700 3.31460500

H 3.51449500 0.72367400 2.52912100

C 1.98109200 -1.87581500 -1.48820700

C 2.48542400 -0.94477300 -2.42086800

C 2.37129600 -3.22278400 -1.59858100

C 3.33084300 -1.36552900 -3.45330100

H 2.22301200 0.11001400 -2.35215200

C 3.21925600 -3.63555200 -2.62837400

H 2.02395000 -3.95355900 -0.86583700

C 3.70454900 -2.70747300 -3.55748900

H 3.70964200 -0.63106600 -4.16832600

H 3.51036600 -4.68671400 -2.70098100

H 4.37920700 -3.02999400 -4.35404300

**TS14**

0 1

C -1.07497500 -1.49680800 1.60879800

C 0.03184800 -1.41772700 2.46348000

H 0.30264400 -0.44025200 2.86458300

C 0.75912500 -2.56800500 2.77958200

H 1.61307700 -2.50050900 3.45676800

C 0.42837000 -3.79657100 2.19758900

H 1.03511900 -4.68346800 2.39454000

C -0.67886400 -3.86623400 1.35499500

C -1.20220700 -5.05387300 0.57951400

H -0.39415300 -5.60446100 0.07341900

H -1.70893800 -5.77257300 1.24756700

C -2.19185200 -4.40481000 -0.40699200

H -3.00153800 -5.07485400 -0.73022800

H -1.65223600 -4.06544000 -1.30348700

C -2.72248100 -3.14280500 0.34269500

C -1.47255300 -2.73411500 1.10125100

C -3.87220800 -3.54746300 1.31999700

H -3.98497700 -2.74941400 2.07114500

H -3.65877000 -4.48336700 1.85602400

C -5.12914700 -3.61080500 0.43141200

H -6.05875600 -3.39863300 0.98238000

H -5.24990100 -4.61085500 -0.02136900

C -4.83592900 -2.57244000 -0.62859800

C -5.71572000 -1.97835600 -1.53302200

H -6.77024200 -2.26398900 -1.55314800

C -5.22618100 -1.00429100 -2.41144400

H -5.89690500 -0.53294200 -3.13375600

C -3.89447700 -0.59052000 -2.33684400

H -3.51029500 0.20915100 -2.97154300

C -3.02441000 -1.17310900 -1.40547400

C -3.47636000 -2.21552100 -0.59297600

O -1.75050700 -0.68095300 -1.28186300

O -1.74509200 -0.34644200 1.26411700

P -1.29597700 0.49010600 -0.13959800

N -2.51955600 1.63872200 -0.06939100

C -2.20563600 3.05264100 -0.36474000

C -1.24413500 3.70441900 0.64031000

C -1.72810500 3.26095400 -1.80795300

H -3.16381900 3.58477500 -0.27573000

C -1.04323900 5.19013800 0.32717600

H -0.27487500 3.18683700 0.60260500

H -1.63357100 3.57102100 1.66097300

C -1.53505100 4.74790300 -2.11695000

H -0.77178800 2.72386700 -1.94799000

H -2.45476800 2.80221500 -2.49786800

C -0.57883900 5.40517100 -1.11684300

H -0.31485000 5.62133300 1.03178200

H -1.99504200 5.73173100 0.48482000

H -1.16432800 4.87566100 -3.14750400

H -2.51539700 5.25828200 -2.07335700

H -0.47944500 6.48223600 -1.33208400

H 0.42444700 4.96362900 -1.23807100

C -3.82956200 1.34091400 0.54906000

C -3.92392100 1.79024200 2.01472400

C -5.02337600 1.88302700 -0.24959700

H -3.92500800 0.24831000 0.54848400

C -5.23687100 1.30433000 2.63890600

H -3.87514600 2.89258200 2.06617700

H -3.05968200 1.39614700 2.56726600

C -6.33757600 1.38081900 0.35903000

H -5.02686100 2.98626600 -0.23131500

H -4.93790400 1.57637700 -1.30020000

C -6.45447600 1.77432600 1.83555700

H -5.31127000 1.64347400 3.68537600

H -5.22651800 0.19920900 2.66917900

H -7.19347400 1.77558800 -0.21320300

H -6.37747200 0.28176700 0.26296500

H -7.38243600 1.36637400 2.26998700

H -6.53220100 2.87470300 1.91029400

Pd 0.83642100 0.94098000 -0.72878300

C 2.44656700 0.99749900 -2.25901200

C 1.75071300 -0.08738300 -2.75435000

C 1.86925500 -1.41133400 -2.14046800

C 2.79327100 -1.62267400 -1.08718300

C 3.72544000 -0.48820000 -0.67396200

C 3.15997400 0.91979700 -0.97696700

H 0.34319700 -2.30747700 -3.36969400

H 2.44692900 1.94166700 -2.80859500

H 1.20051900 0.00027600 -3.69243300

C 1.06837300 -2.48171800 -2.57171300

C 2.89128500 -2.88856300 -0.50792300

C 2.09068900 -3.94714100 -0.94842600

C 1.17865000 -3.74115600 -1.98501500

H 3.60334400 -3.05449900 0.30142700

H 2.18261300 -4.92846600 -0.47820800

H 0.55144200 -4.56318300 -2.33769900

C 5.02341100 -0.52110500 -1.57088900

N 6.11459200 -0.44948000 -0.71943300

O 5.07899900 -0.55355700 -2.77703400

C 7.47988900 -0.42904700 -1.18563400

H 7.45558900 -0.39753900 -2.28260800

H 8.01241700 0.45774200 -0.80461900

H 8.02422600 -1.33200700 -0.86113400

C 5.72531400 -0.54841600 0.62072800

C 4.32464800 -0.62156900 0.70929100

C 6.53931500 -0.62900900 1.74876700

C 3.72828700 -0.85814800 1.93610100

C 5.91554400 -0.82061100 2.99084900

C 4.52769000 -0.95059200 3.08631800

H 2.65158700 -0.99928300 1.98882200

H 6.53036200 -0.88774400 3.89158500

H 4.06252200 -1.12735700 4.05870700

H 7.62559900 -0.56389900 1.67075900

H 3.95338900 1.65890000 -0.87469400

C 2.22988300 2.08999800 0.48097900

C 2.13670200 1.88512100 1.86520200

C 2.40274300 3.40819100 0.01957200

C 2.17451700 2.96574100 2.75321800

H 1.99537900 0.88571500 2.26239200

C 2.44591800 4.48761200 0.90354800

H 2.50516600 3.60086200 -1.05226900

C 2.32863700 4.27111700 2.28004900

H 2.07337900 2.77977700 3.82587500

H 2.57219300 5.50101800 0.51380700

H 2.36183900 5.11194300 2.97675800

**TS2**

0 1

C 1.65820700 1.59030800 1.60605900

C 0.71327800 1.31833600 2.60150500

H 0.71865100 0.33905700 3.07994100

C -0.25474000 2.27290100 2.92419000

H -1.00472700 2.03821300 3.68205800

C -0.30944300 3.48941400 2.23432600

H -1.10920300 4.20452300 2.43969100

C 0.64460500 3.75249400 1.25173700

C 0.75133500 4.94459700 0.32712400

H -0.22863000 5.26167800 -0.06173300

H 1.18140600 5.81462900 0.85405500

C 1.69958500 4.43219700 -0.77576500

H 2.26525600 5.22831400 -1.28123000

H 1.12114300 3.89271300 -1.54206600

C 2.62979800 3.40728300 -0.05115100

C 1.65741300 2.82350900 0.95667500

C 3.80696000 4.15381600 0.65353000

H 4.21066500 3.49781500 1.44075300

H 3.48363000 5.08979200 1.13117100

C 4.86080700 4.35083200 -0.45258100

H 5.89014700 4.40323300 -0.06503400

H 4.68391100 5.28665600 -1.01182000

C 4.63488400 3.14934900 -1.34239000

C 5.47047600 2.62883700 -2.33062900

H 6.42798100 3.10458600 -2.55607900

C 5.06306200 1.48704300 -3.03049500

H 5.69778100 1.07108300 -3.81644000

C 3.86525400 0.84805000 -2.70230000

H 3.55237200 -0.06845400 -3.20413500

C 3.04960000 1.35996000 -1.68574700

C 3.40091700 2.54720400 -1.03876600

O 1.92766700 0.65489800 -1.30916500

O 2.52244700 0.58789100 1.19266200

P 1.92773100 -0.42518300 -0.01168300

N 3.25325500 -1.43306800 -0.14995200

C 3.00922900 -2.87585000 -0.36948800

C 2.26384900 -3.55886200 0.78957100

C 2.32053100 -3.14084800 -1.71690400

H 4.00509400 -3.33937100 -0.42438700

C 2.05882000 -5.05276700 0.52557700

H 1.27875800 -3.07527400 0.91678200

H 2.81768100 -3.39375900 1.72680300

C 2.10839100 -4.63642800 -1.96396900

H 1.34086700 -2.62966500 -1.72252800

H 2.91907400 -2.68489000 -2.52190300

C 1.34264000 -5.28774800 -0.80812000

H 1.49184400 -5.50523700 1.35543600

H 3.03991100 -5.56300600 0.50384300

H 1.57618900 -4.78830900 -2.91713800

H 3.09092300 -5.13222000 -2.07234300

H 1.21325100 -6.36692900 -0.99192300

H 0.32913700 -4.85031700 -0.75682500

C 4.62314900 -0.97536200 0.16174700

C 5.05994900 -1.34621000 1.58564600

C 5.67104600 -1.43405800 -0.86038100

H 4.59496700 0.12164800 0.11273200

C 6.42332700 -0.72568300 1.91022600

H 5.12137300 -2.44590100 1.67531300

H 4.29427000 -1.00048300 2.29501000

C 7.02780700 -0.79241300 -0.54939000

H 5.78195000 -2.53155200 -0.82693400

H 5.33981800 -1.17192800 -1.87400400

C 7.48526900 -1.11743300 0.87690300

H 6.74442900 -1.01865800 2.92327800

H 6.31765500 0.37446600 1.92290300

H 7.78070300 -1.12889500 -1.28108700

H 6.93960900 0.30183000 -0.67159100

H 8.44040400 -0.61296700 1.09819600

H 7.68056400 -2.20299700 0.95367900

Pd -0.25729100 -1.14095800 0.14745200

C -6.53924500 -1.09028900 1.49081100

C -7.67771800 -0.84578200 0.75571500

C -7.70005500 0.18058300 -0.22796700

C -6.52374600 0.97458100 -0.44464800

C -5.34921500 0.67799100 0.31851500

C -5.36853300 -0.33093100 1.26290100

H -9.75218300 -0.17191000 -0.83460700

H -6.52776500 -1.87768000 2.24785400

H -8.58247800 -1.43773900 0.91659200

C -8.86159600 0.43803700 -1.00758800

C -6.56759200 1.99905600 -1.43267700

H -4.46534800 -0.54964500 1.83440900

C -7.70952700 2.21771200 -2.17415700

C -8.86869900 1.43122900 -1.96238500

H -5.68972000 2.62847300 -1.57738100

H -7.72453400 3.01059100 -2.92586200

H -9.76742600 1.61778300 -2.55511500

C -4.10176200 1.49764300 0.11729500

N -2.95509800 0.80696600 -0.23591900

O -4.10795900 2.70931800 0.27093800

C -1.71461300 1.56679800 -0.39185100

H -0.97162900 1.26938700 0.36278800

H -1.29076400 1.40081500 -1.39323200

H -1.95141200 2.62733800 -0.25303300

C -2.98600200 -0.55037400 -0.65599500

C -2.22659200 -1.56622400 -0.01701700

C -3.76986100 -0.92309600 -1.75594800

C -2.33613700 -2.91213400 -0.41578700

C -3.85947400 -2.25242300 -2.17419100

C -3.14098200 -3.24791400 -1.50685100

H -1.81383200 -3.68364900 0.15185800

H -4.49782600 -2.50807800 -3.02231400

H -3.21419600 -4.29128600 -1.82224300

H -4.32689100 -0.14357600 -2.27891400

Br -1.85616200 -1.31554700 2.18472400

**TS4**

0 1

C -1.40550300 1.65611600 -1.64572700

C -0.49776300 1.83036400 -2.69636800

H -0.14174900 0.94964400 -3.23176900

C -0.04970500 3.11436600 -3.01772900

H 0.67349300 3.23999100 -3.82615400

C -0.48989800 4.22554200 -2.28926800

H -0.10990700 5.22408100 -2.51930300

C -1.39339600 4.03470600 -1.24509200

C -1.94820800 5.05128000 -0.27315900

H -1.19845400 5.79440900 0.04163000

H -2.78115300 5.61644100 -0.72780200

C -2.44375300 4.16352300 0.88304200

H -3.24340500 4.61932500 1.48409000

H -1.60667400 3.93324400 1.56162100

C -2.89252100 2.83038300 0.19567300

C -1.87485000 2.75260700 -0.92614000

C -4.35888500 2.96308200 -0.31529900

H -4.54028100 2.17146100 -1.05933100

H -4.54902900 3.93024800 -0.80245400

C -5.22365100 2.71811300 0.93858900

H -6.20598700 2.27897900 0.70399200

H -5.42035700 3.65888300 1.48200800

C -4.36260600 1.78716200 1.76549500

C -4.71295300 1.04766900 2.89486100

H -5.72458700 1.10175800 3.30415100

C -3.74314500 0.23800100 3.49845400

H -3.98784600 -0.33144500 4.39820400

C -2.47707400 0.10395900 2.92357200

H -1.73156400 -0.56892600 3.34427500

C -2.14914000 0.81449200 1.76176100

C -3.06187400 1.73174200 1.23294900

O -0.95080300 0.55765200 1.13656100

O -1.80393100 0.37948200 -1.27933200

P -0.84632300 -0.45853100 -0.20779800

N -1.83670000 -1.77995500 0.02446800

C -1.28816400 -3.06209800 0.52638900

C -0.10337300 -3.61353900 -0.27396900

C -0.95989100 -3.00057200 2.02411800

H -2.10542500 -3.78776100 0.40005800

C 0.33760800 -4.97709700 0.26156800

H 0.74917700 -2.91723200 -0.20690400

H -0.36108100 -3.65926700 -1.34078300

C -0.47530500 -4.34950500 2.56366700

H -0.18195800 -2.23428600 2.18145400

H -1.85019900 -2.66504000 2.57463100

C 0.69771300 -4.90039100 1.74782900

H 1.19286700 -5.34581200 -0.32729700

H -0.47700400 -5.71225400 0.12274300

H -0.19931300 -4.25178700 3.62690300

H -1.31008000 -5.07348500 2.52638900

H 0.99980000 -5.89009600 2.12828500

H 1.57253500 -4.23562400 1.87248500

C -3.27319700 -1.74442000 -0.33841400

C -3.52834900 -2.46570200 -1.66754700

C -4.20639000 -2.25272200 0.76696600

H -3.52614500 -0.68848600 -0.48957900

C -4.99375300 -2.32171800 -2.09109000

H -3.27779100 -3.53648600 -1.55820500

H -2.85074800 -2.05604000 -2.43155700

C -5.66853700 -2.08492800 0.33711700

H -4.01529700 -3.31983700 0.97372700

H -4.01388900 -1.69883500 1.69507100

C -5.95000700 -2.79550700 -0.99096100

H -5.17603600 -2.87478000 -3.02691800

H -5.19740700 -1.25829200 -2.31368600

H -6.33852500 -2.46105600 1.12777300

H -5.88691800 -1.00644400 0.23184400

H -6.99746400 -2.63755300 -1.29728000

H -5.82660900 -3.88498300 -0.84837000

Pd 1.36908000 -0.53823300 -0.89861500

C 4.03579600 -1.52186900 -1.54894800

C 4.92634200 -1.83908700 -0.56736600

C 5.32902300 -0.86155700 0.41721900

C 4.69842600 0.41156900 0.45709600

C 3.54595500 0.65509600 -0.43669300

C 3.36858000 -0.24525500 -1.56101600

H 6.85171500 -2.11169800 1.30570400

H 3.80372700 -2.22006900 -2.35511800

H 5.41413000 -2.81657800 -0.55391700

C 6.37043800 -1.13079300 1.33298600

C 5.13803400 1.37410400 1.38220500

H 3.08715200 0.19116400 -2.52265900

C 6.17364600 1.09171000 2.26344600

C 6.78792600 -0.17229700 2.24439200

H 4.65544700 2.35193500 1.41449600

H 6.50750100 1.85276500 2.97210500

H 7.59739200 -0.39696700 2.94277400

C 3.13922000 2.10335200 -0.71616600

N 2.26802300 2.59888000 0.23437700

O 3.53632200 2.72980200 -1.67568600

C 1.87908400 3.99259100 0.24109100

H 2.17505600 4.42613500 -0.72088000

H 0.79065000 4.07577900 0.35531800

H 2.37768300 4.54260900 1.05758800

C 1.88744200 1.69323400 1.22806600

C 2.10892800 0.34169200 0.89940900

C 1.45889500 2.05911900 2.50728800

C 2.09157400 -0.61997000 1.92067400

C 1.31934900 1.06635000 3.47947400

C 1.66580900 -0.26327300 3.20075700

H 2.36538100 -1.65360600 1.69775200

H 0.97302200 1.33962200 4.47869200

H 1.59257100 -1.02358300 3.98160700

H 1.26081200 3.10396800 2.75044000

Br 0.74487400 -1.57980400 -3.11885900

**TS7**

0 1

C -1.57275300 0.59821600 -2.29447700

C -0.95024500 -0.01185700 -3.38980800

H -0.84670200 -1.09544700 -3.40251600

C -0.44450200 0.77848100 -4.42525500

H 0.04530300 0.29439200 -5.27307600

C -0.50422100 2.17687400 -4.34981500

H -0.05087500 2.79344100 -5.12976600

C -1.14204700 2.76889400 -3.25918100

C -1.31132300 4.23761000 -2.93588100

H -0.37941600 4.80681500 -3.07661600

H -2.06813300 4.70064800 -3.59311800

C -1.78341900 4.21195700 -1.46710700

H -2.39711300 5.07933400 -1.18393400

H -0.91204400 4.18464700 -0.79349000

C -2.55667000 2.86336700 -1.32775400

C -1.73319700 1.98301700 -2.25381900

C -4.01326300 3.02170200 -1.87514700

H -4.39744000 2.02010400 -2.12508300

H -4.05505200 3.63238400 -2.78826100

C -4.81414100 3.60556700 -0.69696700

H -5.88210400 3.33941500 -0.72681000

H -4.76149200 4.70873600 -0.68367600

C -4.10305200 3.02264400 0.50288100

C -4.55044200 2.93136500 1.82113400

H -5.52804800 3.32887300 2.10414900

C -3.73086800 2.31660200 2.77534800

H -4.06138000 2.24288800 3.81405700

C -2.50716600 1.75686400 2.40107900

H -1.87535800 1.23467100 3.12079100

C -2.07966700 1.83504900 1.07025900

C -2.85004500 2.51140300 0.12194900

O -0.92276300 1.19658900 0.69385900

O -1.98379600 -0.17237500 -1.22707800

P -0.94925400 -0.38042900 0.07673100

N -2.03470300 -1.26867000 1.00570300

C -1.59621900 -2.01930700 2.20623900

C -1.71873700 -3.54202500 2.05309700

C -0.18734800 -1.63767500 2.65529500

H -2.26708900 -1.71222400 3.02481000

C -1.33797000 -4.25651200 3.35455300

H -1.04377600 -3.87265700 1.25110300

H -2.74309100 -3.81342700 1.75982500

C 0.20271800 -2.35413400 3.95111900

H 0.54291700 -1.90956300 1.88385000

H -0.13401800 -0.54866200 2.79207100

C 0.07339400 -3.87278800 3.80670300

H -1.41496900 -5.34710700 3.21510200

H -2.06439600 -3.99080800 4.14589000

H 1.23704100 -2.08401300 4.21869900

H -0.44261100 -2.00193200 4.77794200

H 0.32306500 -4.37138300 4.75805600

H 0.79698000 -4.22069500 3.05198400

C -3.49682700 -1.19716400 0.71349500

C -3.96728700 -2.23725500 -0.31612100

C -4.38454200 -1.24385500 1.96273000

H -3.67303600 -0.21246300 0.26396300

C -5.42395200 -1.97082700 -0.70963900

H -3.87576700 -3.25045700 0.10873000

H -3.31719300 -2.19272400 -1.19864100

C -5.84116900 -0.96325300 1.57309200

H -4.32539700 -2.23433400 2.44353800

H -4.04439600 -0.49925900 2.69531200

C -6.34416100 -1.95051400 0.51500200

H -5.76581200 -2.72525300 -1.43715300

H -5.48054200 -0.99384000 -1.22422500

H -6.48095000 -1.00115900 2.47023200

H -5.91205700 0.06737300 1.18159700

H -7.37710900 -1.70253700 0.21888400

H -6.37977400 -2.96354300 0.95605600

Pd 1.21031300 -0.70969600 -0.75365400

C 2.04353800 0.91861800 -1.89105000

C 3.03103800 -0.07293000 -2.01080300

C 4.12684900 -0.17880500 -1.04523000

C 4.13022700 0.63375700 0.11082100

C 2.97761900 1.59016800 0.36665300

C 1.79821400 1.37961700 -0.57416000

H 5.13703900 -1.76006400 -2.11054200

H 1.38609100 1.17363200 -2.71990600

H 3.15522800 -0.58534100 -2.96455800

C 5.16592000 -1.10464500 -1.23812700

C 5.18395200 0.52793400 1.02019300

C 6.22225600 -0.38497700 0.81204000

C 6.20675900 -1.21000700 -0.31638700

H 5.18062100 1.15082700 1.91723300

H 7.03375300 -0.46098000 1.53902000

H 7.00196900 -1.94143200 -0.47519200

C 3.40930900 3.07404500 0.07855300

N 3.08342100 3.81993900 1.19728100

O 3.88706100 3.50152200 -0.94614700

C 3.29252900 5.24460400 1.28709200

H 3.69830200 5.58288000 0.32482000

H 2.34384100 5.76872600 1.48947300

H 4.00756500 5.49093200 2.08981800

C 2.59699000 3.01100300 2.23519400

C 2.55122100 1.67160900 1.81594700

C 2.22585800 3.38610000 3.52356200

C 2.17808700 0.67073400 2.69596900

C 1.82083800 2.36850100 4.40247100

C 1.80725500 1.02936600 4.00350700

H 2.18790200 -0.36928100 2.36793400

H 1.52547900 2.63220800 5.42089700

H 1.50806400 0.25518300 4.71232100

H 2.25606700 4.42806400 3.84573600

H 0.96320900 2.05820700 -0.40122100

B 2.12030600 -3.28219600 -0.04677200

C 0.90228900 -2.76048300 -1.64268300

C -0.44234700 -3.19040700 -1.57605300

C 1.52061000 -2.89917300 -2.90869200

C -1.13462500 -3.68735900 -2.68373800

H -0.97147400 -3.14682000 -0.62473900

C 0.84482200 -3.38642500 -4.02999000

H 2.57962100 -2.65435000 -3.01161200

C -0.49563100 -3.77508400 -3.92341700

H -2.17597500 -4.00299700 -2.57785500

H 1.36501500 -3.47236200 -4.98828200

H -1.03146800 -4.15594300 -4.79682800

O 2.53526900 -2.19402500 0.77391600

H 3.46982000 -2.00913700 0.61273400

O 3.18686200 -3.90520700 -0.70298100

H 2.88579700 -4.67882100 -1.19253500

F 1.19327100 -4.09001100 0.59472100

**TS9**

0 1

C -0.98945700 -1.18648900 2.16268400

C -0.29635500 -0.62571600 3.24348900

H -0.52463700 0.40231000 3.52924900

C 0.68465200 -1.36645800 3.90665300

H 1.21882100 -0.92226000 4.75001100

C 1.02464000 -2.65070600 3.46111900

H 1.83615100 -3.20501500 3.93894400

C 0.32436600 -3.20107800 2.38805900

C 0.54251600 -4.52604900 1.69037300

H 1.60827600 -4.72215800 1.49360400

H 0.17276600 -5.36340600 2.30824200

C -0.27943300 -4.36752800 0.39558900

H -0.64365300 -5.31996000 -0.01615500

H 0.33221100 -3.88040800 -0.37725000

C -1.44354600 -3.40357000 0.78558700

C -0.72598400 -2.49820500 1.77079200

C -2.58636500 -4.19836900 1.49689000

H -3.19416200 -3.48435200 2.07503000

H -2.20099600 -4.95323600 2.19721000

C -3.42607900 -4.78630800 0.34815300

H -4.48187700 -4.94140500 0.62007200

H -3.03395600 -5.76709100 0.02545000

C -3.25453000 -3.75680000 -0.74577200

C -3.99864700 -3.60082200 -1.91562000

H -4.82373500 -4.27977300 -2.14413300

C -3.66971200 -2.56121700 -2.79305700

H -4.23079000 -2.42974600 -3.72128900

C -2.65353200 -1.65961200 -2.46854100

H -2.41276700 -0.81694800 -3.11798100

C -1.93591300 -1.80349800 -1.27379400

C -2.18889900 -2.89325100 -0.43654500

O -0.99828200 -0.86426500 -0.94019700

O -1.86775200 -0.40587800 1.44524400

P -1.23786100 0.43905900 0.11462200

N -2.68468100 1.15893900 -0.33376100

C -2.68625600 2.51465000 -0.91418100

C -2.22413000 3.61674900 0.04741200

C -1.92112100 2.58675600 -2.24264000

H -3.73943800 2.72576600 -1.14901300

C -2.38361000 4.99767300 -0.59754500

H -1.17107800 3.45158300 0.31426200

H -2.80373900 3.55260700 0.98167900

C -2.08686800 3.96233500 -2.89432200

H -0.85179900 2.38714100 -2.05019600

H -2.28062500 1.78816000 -2.91137900

C -1.65649900 5.08394400 -1.94441700

H -2.00700500 5.77251400 0.08814500

H -3.45874700 5.20750400 -0.75347000

H -1.51182600 4.01044300 -3.83381400

H -3.14843800 4.10268000 -3.17104200

H -1.82891100 6.06851200 -2.41058700

H -0.57196500 5.00225600 -1.76928700

C -3.98692700 0.51078300 -0.06697700

C -4.67658300 1.06264500 1.18924200

C -4.95474200 0.53682500 -1.25820000

H -3.76744900 -0.54627300 0.12990300

C -5.93409700 0.24858500 1.51347700

H -4.94612300 2.12147400 1.02334600

H -3.96627300 1.03265600 2.02769600

C -6.19398300 -0.30802400 -0.94473300

H -5.27848400 1.57023100 -1.46833000

H -4.44950900 0.16560300 -2.15913100

C -6.89835700 0.18832200 0.32335900

H -6.44116200 0.66492100 2.39953600

H -5.62825100 -0.77883700 1.78318500

H -6.88829800 -0.29173300 -1.80115400

H -5.88567000 -1.36016300 -0.81219200

H -7.76182000 -0.45512300 0.56115200

H -7.30468900 1.19946700 0.13629500

Pd 0.84994700 1.17526000 0.55089400

C 2.70879100 0.62878400 1.75987100

C 3.01007600 1.91807700 1.12762700

C 3.71749100 1.85175400 -0.17761500

C 3.90727100 0.62423600 -0.84960500

C 3.52220100 -0.70694000 -0.22013300

C 2.77878100 -0.55669900 1.08859700

H 4.12733900 3.96755900 -0.21104600

H 2.36281500 0.62989600 2.79565800

H 3.42129300 2.66577700 1.80552200

C 4.23695100 3.02603600 -0.75176000

C 4.54824900 0.61730500 -2.09478000

C 5.02578000 1.79367500 -2.67030800

C 4.88278900 3.00412700 -1.98535800

H 4.68231100 -0.33424600 -2.61480700

H 5.52417500 1.76329400 -3.64192100

H 5.27699500 3.92961700 -2.41160300

C 4.82180700 -1.50731600 0.16144700

N 4.69971500 -2.77702500 -0.38112300

O 5.74081800 -1.11551900 0.84025100

C 5.68758500 -3.81192500 -0.20016500

H 6.47445400 -3.40899400 0.45076800

H 5.24333700 -4.70405300 0.27212300

H 6.13159800 -4.11137300 -1.16452100

C 3.55171500 -2.89064000 -1.17729500

C 2.83667100 -1.68090700 -1.16223000

C 3.12196700 -3.98495600 -1.92473000

C 1.69502100 -1.53579700 -1.93009300

C 1.94995800 -3.83272500 -2.68422900

C 1.24680900 -2.62555400 -2.69572400

H 1.14174900 -0.59806000 -1.92357700

H 1.58672200 -4.67600100 -3.27660500

H 0.33646700 -2.52929000 -3.28974500

H 3.67669300 -4.92451400 -1.92963500

H 2.47275300 -1.47724100 1.58660000

C 1.38444400 3.21735500 1.08280600

C 0.94605800 3.56414400 2.37365500

C 1.42713000 4.21085000 0.09639700

C 0.51830300 4.86315700 2.65601500

H 0.93443200 2.81109500 3.16701600

C 1.02336400 5.51891000 0.38810600

H 1.77028400 3.96505700 -0.91063900

C 0.56144500 5.85039300 1.66435000

H 0.16008700 5.10935400 3.65930400

H 1.05925300 6.28173600 -0.39414900

H 0.24248200 6.87105700 1.88772800
